# Domino Synthesis of Coumarins via Phosphine-Mediated Formal *Oxa*-[4 + 2] Cycloaddition: Discovering a Missing Recyclization of Maleimides with Salicylaldehydes

**DOI:** 10.1021/acs.joc.6c00595

**Published:** 2026-07-03

**Authors:** Fernando Alves Barretto, Pedro P. de Castro, Kleber T. de Oliveira, Silvio Cunha

**Affiliations:** † Instituto de Química, 130230 Universidade Federal da Bahia, Campus de Ondina, Bahia 40170-115, Brazil; ‡ Departamento de Farmácia, 28113 Universidade Federal de Juiz de Fora - Campus Governador Valadares, Minas Gerais 35010-177, Brazil; § Departamento de Química, 67828 Universidade Federal de São Carlos, São Carlos 13565-905, Brazil

## Abstract

A one-pot procedure for direct access to a hybrid of coumarin-acetamide through a tributylphosphine-mediated reaction between maleimides and salicylaldehydes under microwave irradiation is presented. The process involves the in situ ylide formation, followed by a Wittig reaction, itaconimide isomerization, and intramolecular cyclization, and represents a recyclization of maleimides via formal *oxa*-[4 + 2] cycloaddition. A broad scope of 24 derivatives was selectively formed in up to a 68% overall yield, after crystallization. This protocol was also transposed to continuous flow (up to 65% yield), allowing for the reaction scale-up to a 3 mmol scale. Control reactions and computational calculations revealed that the tributylphosphine plays a dual role, acting not only as a substrate for the phosphonium ylide formation but also as a catalyst for itaconimide isomerization. The synthesized coumarins exhibited fluorescence properties, with large Stokes shift values and fluorescence quantum yields (up to 84%), significantly influenced by substituents.

## Graphical abstract

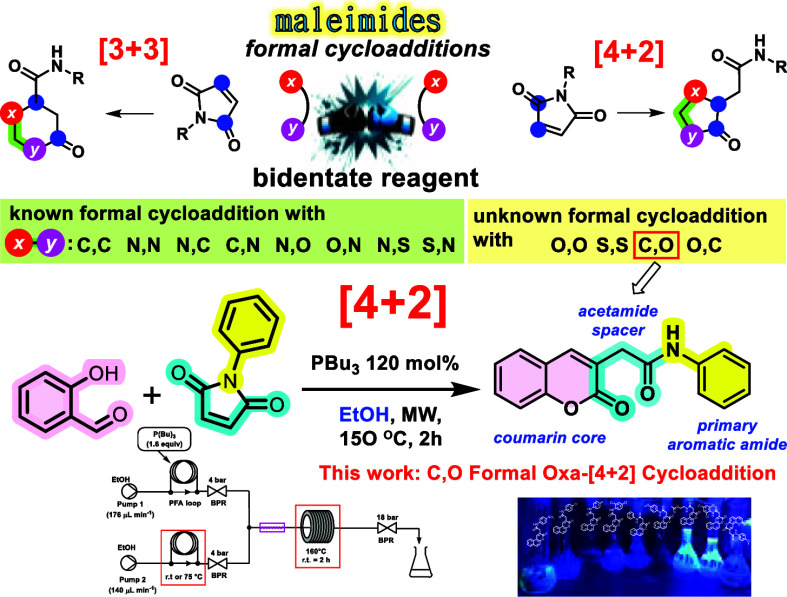

## Introduction

Maleimide is a multifaceted motif in organic synthesis with diverse applications.^1,2^ In the context of cycloadditions, the Diels–Alder (DA) reaction and 1,3-dipolar cycloadditions (1,3-DC) result in carbocycles and heterocycles, respectively, by reaction with C3–C4 sites of maleimide, Figure 1A.^3–5^ Complementary, formal cycloadditions of maleimide present a versatile reactive pattern which affords five- and six-membered heterocycles.^6,7^ To this end, there are several known combinations of maleimide and X,Y bidentate reagents (indicated as “X-spacer-Y” in Figure 1A), which form two new sigma bonds with C2–C3 or C2–C4 sites of maleimides. However, there are unknown X,Y combinations among such formal cycloadditions, and the *oxa*-[4 + 2] annulation of maleimides with a C,O bidentate reagent to form a six-membered ring by recyclization is a missing reactive pattern, Figure 1A.^8^

**Figure 1:**
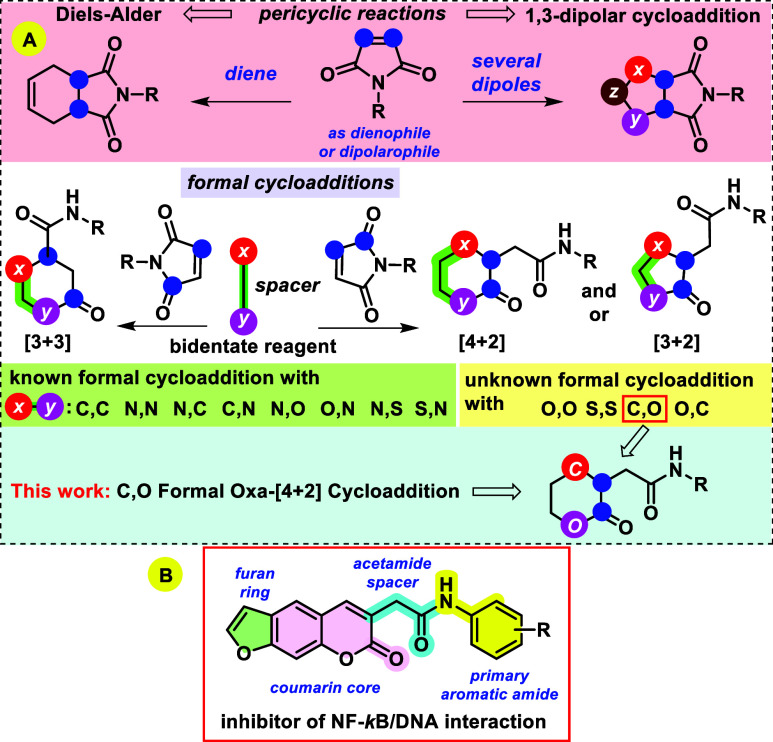
(A) Annulations of maleimide via pericyclic and formal cycloaddition reactions to form 6- and 5-membered rings; (B) representative structure of hybrid coumarin acetamide of the potential candidate against the inflammatory phenotype of cystic fibrosis^12^ (essential units highlighted).

Coumarin is a privileged structural scaffold with diverse biological applications.^9–12^ Thus, a hybrid of coumarin–acetamide was described as a potential candidate against the inflammatory phenotype of cystic fibrosis, indicated in Figure 1B with essential units highlighted.^13^ There are three known routes to this rare hybrid, and the most classical involves a stepwise approach that installs the amide function via traditional coupling reagents to a previously prepared functionalized coumarin core, Figure 2A.^14,15^ The other synthesis of the coumarin–acetamide hybrid involves an itaconimide, which undergoes a formal annulation under basic conditions, forming its enolate that reacts with a *p*-quinone methide, Figure 2B;^16^ and the oldest reported synthesis is based on an intramolecular photochemical double bond isomerization of itaconimides using UV light, Figure 2C.^17^

**Figure 2:**
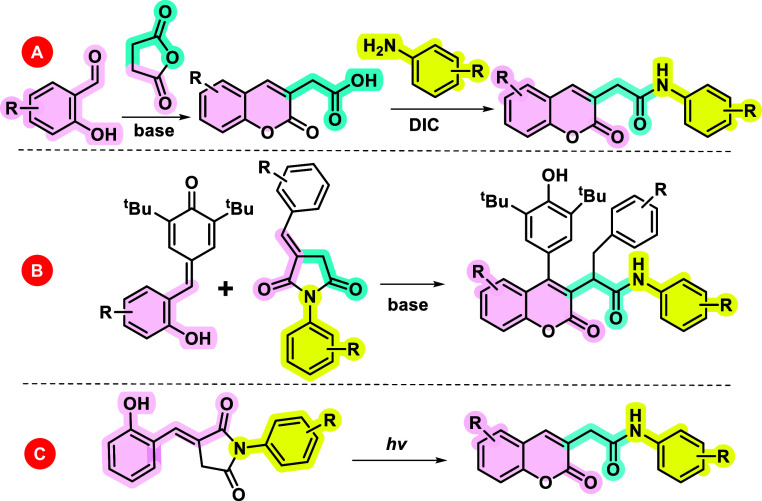
Reported routes to the hybrid of coumarin–acetamide moieties.

Inspired by these works, we developed a domino synthesis of a coumarin–acetamide hybrid under microwave and continuous flow conditions, which accesses both moieties in a single step by the combination of maleimide with salicylaldehyde as a C,O bidentate reagent, via phosphine-mediated formal *oxa*-[4 + 2] cycloaddition. Thus, this study covers the gap of this missing reactive pattern of a formal cycloaddition of maleimide (Figure 1A) and describes a domino route to potential bioactive coumarins.

## Results and Discussion

According to Figure 2, the simplest route to access the hybrid of coumarin–acetamide is route C from an E-isomer of itaconimides, which is photochemically converted to a Z-isomer, allowing the adequate proximal geometry between the phenolic OH group and maleimide α,β-unsaturated carbonyl to the intramolecular *O*-acylation, affording a coumarin core. We rationalized that the double bond of E-itaconimides is sufficiently electrophilic to be isomerized by a phosphine acting as a Lewis base.^18^ To test this initial hypothesis, some itaconimides were synthesized based on literature conditions,^19,20^ aiming at a posterior reaction to convert them into coumarins, Scheme 1.

**Scheme 1:**
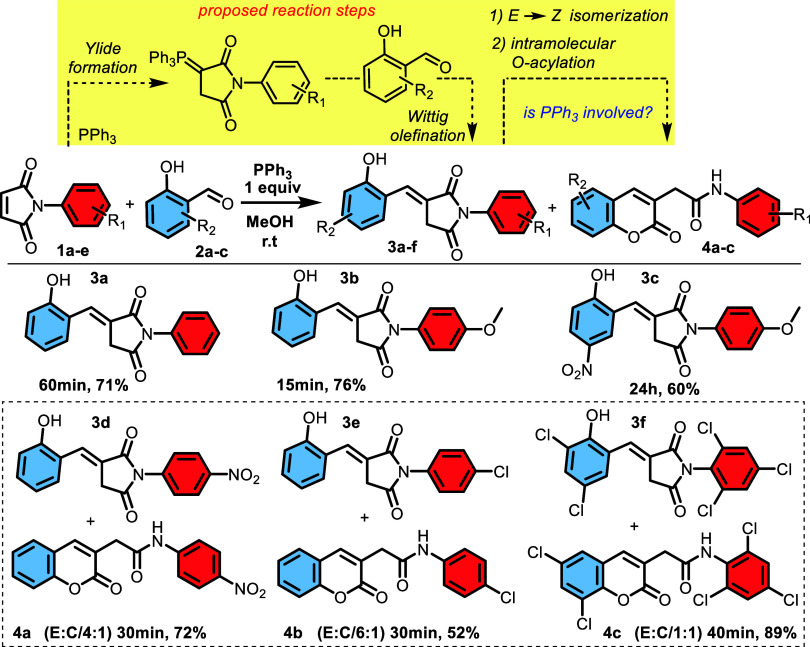
Domino Reaction with PPh_3_ to Access Itaconimides and First Domino Synthesis of Coumarins via Formal *Oxa*-[4 + 2] Cycloaddition^a^

a Reaction under literature conditions^18,19^ to the synthesis of itaconamid **3**; E: E-isomer of **3**, and C: coumarin.

We decided to combine the domino approach, reacting equimolar amounts of *N*-phenyl maleimide, triphenylphosphine (Ph_3_P), and aldehyde but using methanol as a solvent instead of ethanol, because this reaction was found to be faster in methanol. Itaconimides **3a**–**c** were exclusively formed by the described phospha-Michael addition/Wittig olefination, Scheme 1.^20–22^ To our surprise, in the reaction of *N*-aryl maleimides **1c**–**d** containing electron-withdrawing groups (EWG = 4-NO_2_, 4-Cl, and 2,4,5-Cl), **3d**–**f** were isolated as mixtures with its isomer coumarin **4a**–**c**, which correspond to a domino synthesis of coumarin via formal *oxa*-[4 + 2] cycloaddition. Interestingly, the amount of coumarin increases as the electron-withdrawing capacity of aryl-maleimide increases, from **4b** to **4a** and **4c**, Scheme 1.

The formation of **4a**–**c** suggests that PPh_3_ is not only an active component, participating exclusively in the Wittig olefination. Its nucleophilic/basic character should drive the formation of observed coumarins. Thus, the more basic tributylphosphine (Bu_3_P)^23–25^ was tested by taking **2a** and **1a,d** as model reactants under the same reaction conditions with Ph_3_P for these substrates, and the same product distribution was observed; **3a** and a mixture of **3d/4a** were isolated, albeit in low yields compared to PPh_3_ (Scheme 2, condition A; see also Table S1 to details of the optimization of reaction conditions between **1a** and **2a**). However, in EtOH (condition B), **3a** was isolated in a higher yield (85%) in relation to the previous condition (44%), and its purification was simpler because **3a** is less soluble in cold ethanol than methanol, and the byproduct tributylphosphine oxide (Bu_3_PO) remains in solution (an additional advantage of Bu_3_P is that Bu_3_PO is more soluble than Ph_3_PO in ethanol). These experimental observations make the combination of Bu_3_P and ethanol as ideal to optimize the reaction conditions and avoid purification by column chromatography. Furthermore, because maleimides are poorly soluble solids in ethanol at room temperature (ethanol being the typical solvent for their recrystallization), microwave (MW) heating was evaluated to improve yield and selectivity, Scheme 2.

**Scheme 2:**
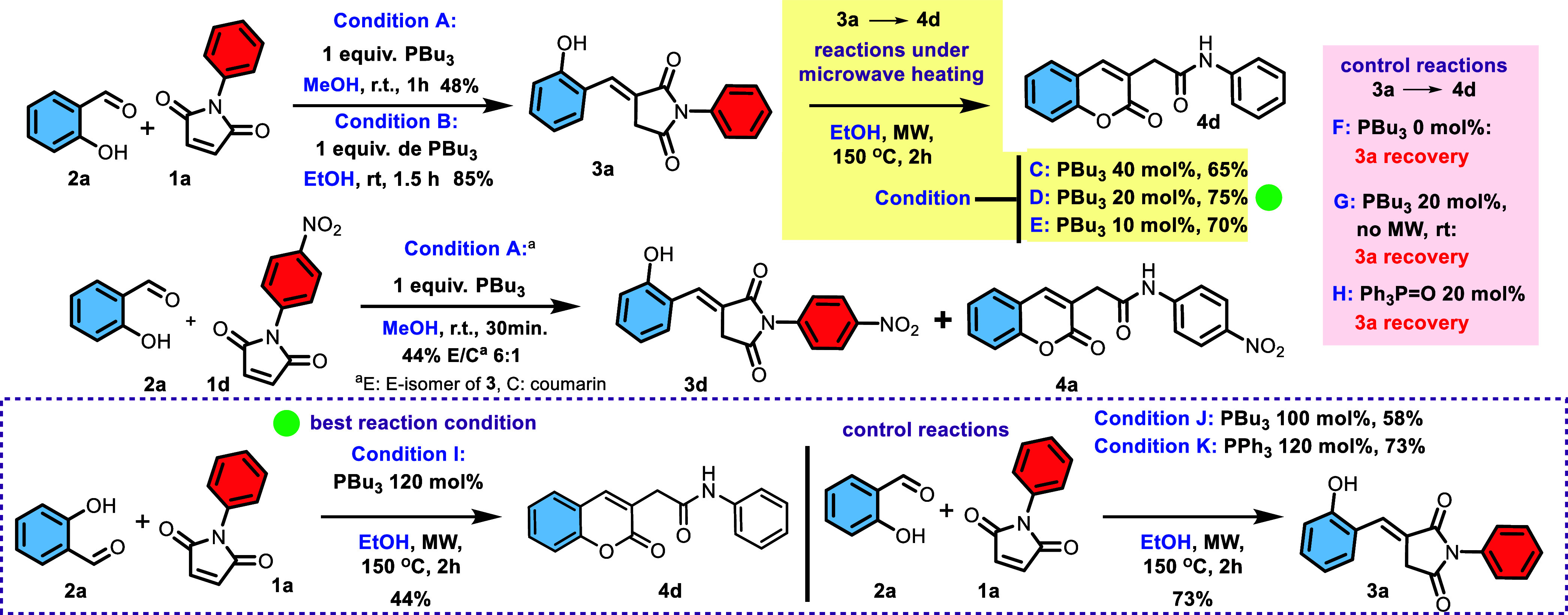
Reaction Optimization with PBu_3_ to Access Itaconimides and Coumarins via Formal *Oxa*-[4 + 2] Cycloaddition

We then turned our attention to the intramolecular cyclization of **3a** to afford **4d** under microwave irradiation. The optimal MW reaction temperature was defined in a single screening experiment using 1.2 equiv of Bu_3_P, whereby **2a** and **1a** were reacted in ethanol, initially at 100 °C and then heated to 120 °C because reactants were still detectable by TLC at these temperatures; the starting materials were completely consumed when the reaction reached 150 °C. The phosphine catalyst loading was tested using 40/20/10 mol % (conditions C–E), revealing 20 mol % as optimal for this transformation (condition D). Under a control reaction in the absence of Bu_3_P (condition F) or without heating (condition G), **3a** was recovered, suggesting that the association of phosphine and high temperatures provided by MW irradiation is necessary to afford the desired product. Moreover, the use of triphenylphosphine oxide failed to afford the product (condition H), confirming that phosphine is the catalyst for this transformation. These results indicate that a new condition to the stepwise route to coumarin was achieved, and the association of Bu_3_P and MW heating proved to be essential for the reaction, likely due to the necessity of isomerizing the itaconimide before the ring closure step, supporting the proposed reaction pathway (Scheme 1).

With these achievements in hand, a more practical synthetic route to a hybrid of coumarin–acetamide became the goal to work with. Thus, having established a protocol for domino transformations (phospha-Michael for ylide formation, Wittig reaction, double-bond isomerization, and intramolecular cyclization), the possibility of a one-pot procedure for this transformation was evaluated, in search of a direct reaction with the new reactivity pattern via phosphine-mediated formal *oxa*-[4 + 2] cycloaddition. The use of 1.2 mol equiv of tributylphosphine in the presence of salicylaldehyde and *N*-phenyl maleimide, utilizing ethanol as a solvent, for 2 h under microwave irradiation at 150 °C was assessed (Scheme 2, condition I). To our delight, the desired coumarin **4d** was isolated in a 44% yield. It is worth to mention that a simple procedure for the purification of the coumarin by precipitation was also developed, contributing to a greener synthetic protocol. Additionally, control reaction had proven that the excess and nature of phosphine are pivotal because by using the optimized reaction parameters but 100 mol % (condition J) of Bu_3_P, or 120 mol % of Ph_3_P instead of Bu_3_P (condition K), itaconimide **3a** was the sole product formed.

To test the generality of the developed methodology, a representative scope was evaluated, as shown in Scheme 3. The reaction tolerated the use of a diversity of substituted *N*-aryl maleimides. For example, the *p*-chloro- and *p*-methyl-substituted analogues **4b** and **4f** were prepared in 59 and 50% yields, respectively. Notably, even sterically hindered substituents, such as 2,4-dichloro and 2,4,6-trichloro, were well tolerated, affording **4e** and **4c** in up to a 58% yield. Strong electron-withdrawing *p*-nitro derivative **4a** was prepared in a 41% yield. The use of a maleimide bearing a pyrazolone substituent was also successfully evaluated, producing **4g** in a 22% yield. The use of other aldehydes, such as *o*-vanillin, was investigated, affording products **4h**–**4i** in yields ranging from 50 to 68%. Other aldehydes were also tolerated under optimized reaction conditions, including the eugenol derivative 5-allyl-2-hydroxy-3-methoxybenzaldehyde (compounds **4j** and in up to a 63% yield).

**Scheme 3:**
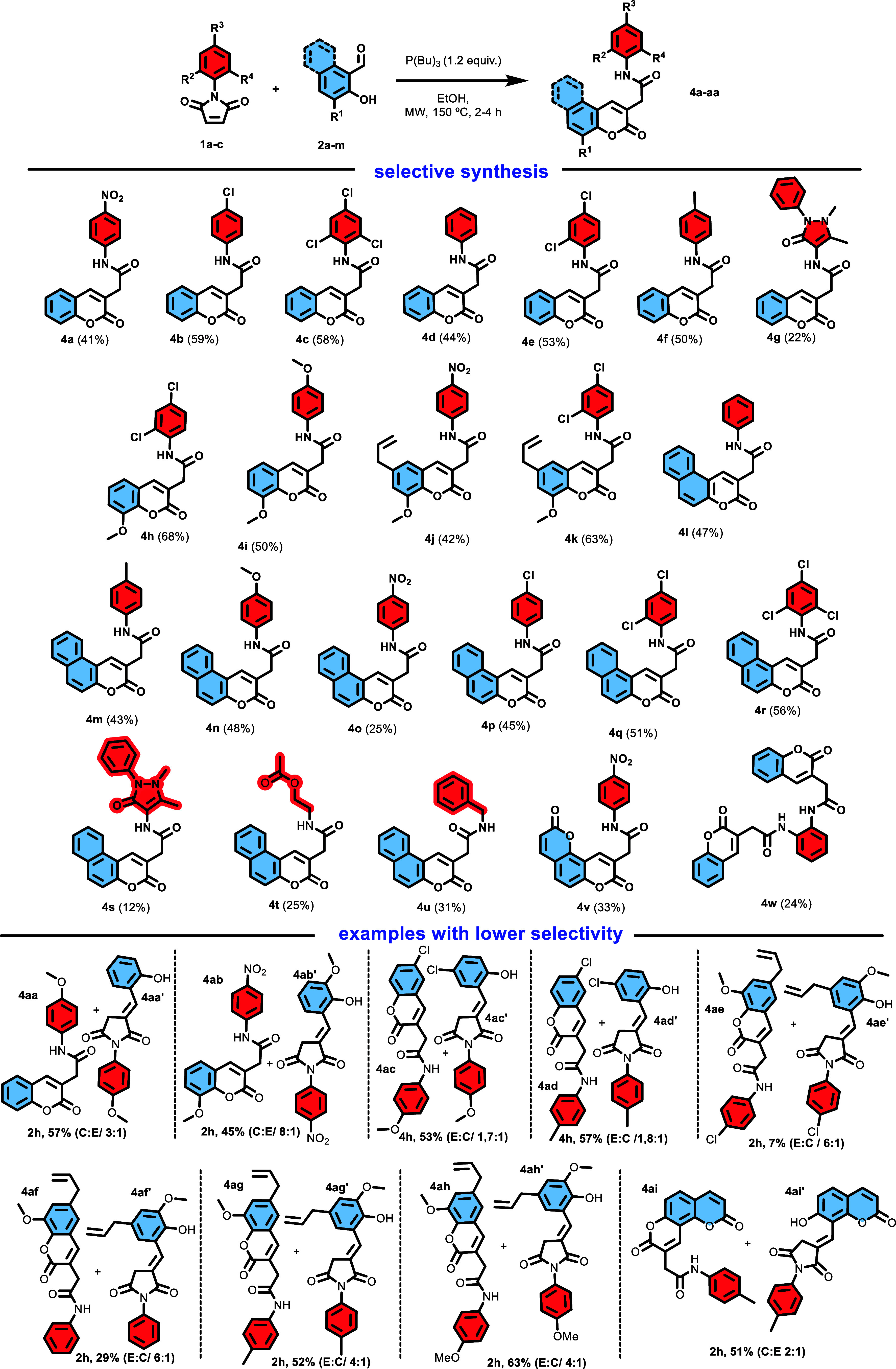
Scope of Bu_3_P-Mediated Formal *Oxa*-[4 + 2] Cycloaddition to Coumarin Synthesis^a^

a Reactions were carried out at 0.3 or 0.5 mmol scale.

The reaction between 2-hydroxy-1-naphthaldehyde and a diversity of *N*-arylmaleimides was also investigated, affording compounds **4l**–**4u** in yields ranging from 25% to 56%. Once more, the use of both electron-donating and -withdrawing substituents was well tolerated. The use of a pyrazolone-containing maleimide was also demonstrated, leading to derivative **4s** in a 12% yield. *N*-Alkyl maleimides were also successfully tested under the optimized reaction conditions, affording compounds **4t** and **4u** in up to a 31% yield.

Notably, the preparation of *bis*-coumarins was demonstrated through the reaction between *N*-aryl maleimides and 7-hydroxy-2-oxo-2*H*-chromene-8-carbaldehyde, affording analogue **4v** in a 33% yield. Last, the use of the 1,1′-(1,2-phenylene)*bis*(1*H*-pyrrole-2,5-dione) was also demonstrated, leading to product **4w** in a 24% yield, through two consecutive intramolecular cyclization reactions. Although in some cases the isolated yields may seem moderate or low at first sight, it is worth to mention that the formation of these analogues involves several steps (including ylide formation, Wittig reaction, itaconimide isomerization, and intramolecular ring-closure). This leads to an average yield per step ranging from 60% (for product **4s**) to 91% (for derivative **4h**).

As limitation, attempts to prepare other derivatives resulted in lower selectivity because the mixture of the desired coumarin **4aa–4ai** and the E-itaconimide intermediate **4aa′**–**4ai′** was formed, even when the reaction was maintained under microwave heating for 2–4 h, Scheme 3.

To provide an alternative for scaling up the synthesis of these analogues, we decided to investigate whether the microwave-assisted batch protocol could be transposed to continuous flow conditions, as shown in Scheme 4. To this purpose, several reactions were carried out in order to optimize the best conditions for this transformation. In most cases, a mixture of the desired coumarin and the itaconimide intermediate was found (for full details, see the Supporting Information).

**Scheme 4:**
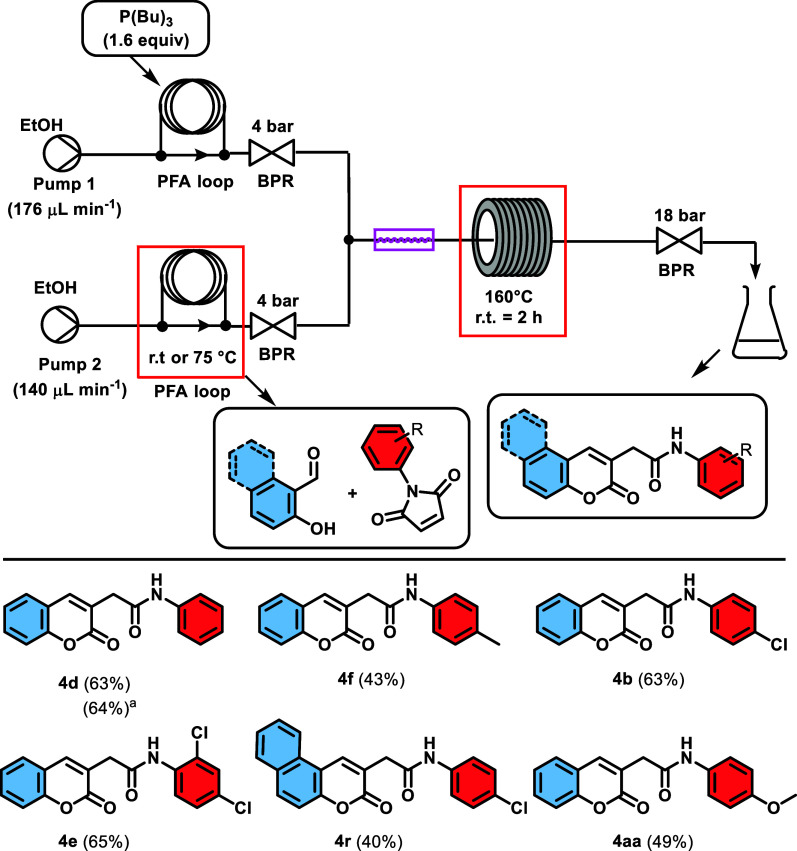
Continuous Flow Setup for the Preparation of Coumarins^a^

a Twelve hour run (3 mmol scale).

After careful analysis of the reaction setup, including the reactor temperature and the concentration of the reagents, we found the setup shown in Scheme 4 to be optimal for this transformation. Thus, the optimized reaction conditions involved the use of two pumps, the first with tributylphosphine (1.6 eq., 0.09 M in ethanol) and the second with the *N*-aryl maleimide and the aldehyde (0.07 M in ethanol). The pumps were set to 176 μL min^–1^ and 140 μL min^–1^, respectively (total flow of 316 μL min^–1^). The solutions were pumped through a Y mixer, passed through a second static mixer, and entered the reactor. After 2 h residence time at 160 °C, the crude reaction mixture was collected, cooled, and the precipitate filtered off, affording the pure coumarin **4d** in a 63% yield.

By using this condition, a small scope was prepared, including variations in both *N*-aryl maleimide and the aldehyde. The isolated yields were, in most cases, comparable to those obtained in the microwave-assisted synthesis. For example, while products **4d** (63% against 44% in MW), **4b** (63% against 59% in MW), and **4e** (65% against 53% in MW) were isolated in better yields in continuous flow than in the batch methodology, analogues **4r** (40% against 45% in MW) and **4f** (43% against 50% in MW) presented an opposite profile.

Unlike batch conditions, coumarin **4aa** was successfully selectively prepared in a 49% yield. Notably, the reaction was successfully scaled up to a 3 mmol scale (long-term experiment), affording the desired product in a 64% isolated yield (536 mg).

Next, we turned our attention to the key aspects concerning the reaction mechanism, as shown in Scheme 5. The formation of phosphorus ylides from the reaction between maleimides and phosphines is well-known in the literature.^26,27^ In this context, the development of protocols for the in situ generation of ylides, followed by the one-pot preparation of functionalized analogues, is of great interest in organic synthesis.^28,29^ It is also well-established that the Wittig reaction of these ylides with aldehydes affords E-itaconimides.^30^ The main doubt regarding the mechanism was whether the phosphine-catalyzed isomerization of E-itaconimide was viable, affording the Z-isomer, which presents an adequate molecular arrangement for the intramolecular cyclization to produce the desired coumarin. To this purpose, control experiments (Scheme 2, conditions C–H,J,K) and density functional theory (DFT) calculations were carried out.

**Scheme 5:**
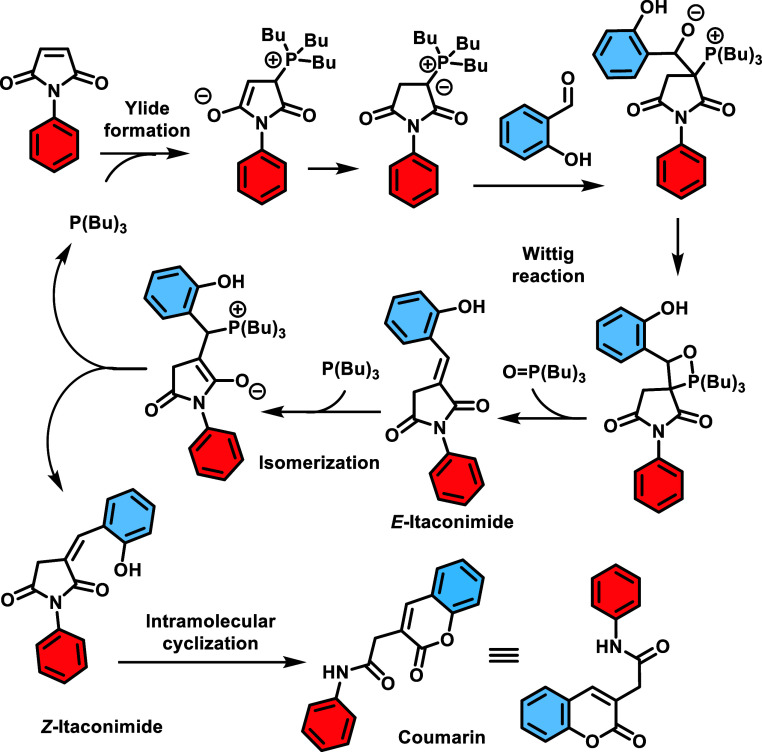
Proposed Reaction Pathway to the Formal *Oxa*-[4 + 2] Cycloaddition

It was observed that when a stoichiometric loading of tributylphosphine (1 equiv) is used, only the E-itaconimide intermediate is formed in a 58% yield (Scheme 2, condition J). Under these conditions, it is expected that all phosphine was consumed for ylide formation (forming tributylphosphine oxide), leaving no phosphine to catalyze the isomerization step. Interestingly, by using 1.2 equiv of the phosphine, the coumarin is formed in a 44% yield, suggesting that the phosphine acts as a catalyst for the E-to-Z-itaconimide isomerization step.

The isomerization step was investigated using computational calculations, Figure 3. The most stable isomer is the E-itaconimide (0.00 kcal mol^–1^, against 4.19 kcal mol^–1^ of the Z-isomer), which explains the reason for its isolation as the sole intermediate. The phosphine-mediated isomerization involves a Michael-type attack to the E-itaconimide (Δ*G* ^‡^ = 14.93 kcal mol^–1^), affording an ionic intermediate (Δ*G* ^‡^ = 5.34 kcal mol^–1^). This intermediate presents a free rotation over the C–C dihedral, allowing the access to an adequate molecular arrangement for the phosphine elimination (catalyst regeneration), with formation of the Z-itaconimide. This step presents a Δ*G* ^‡^ = 18.91 kcal mol^–1^ and a Δ*G* = −1.15 kcal mol^–1^. These values suggest the isomerization of the itaconimide is viable to occur under the experimental reaction conditions (150 °C and 250 psi). Finally, the intramolecular cyclization to form the desired coumarin is thermodynamically favorable (Δ*G* = −9.85 kcal mol^–1^). The gain in terms of Gibbs free energy explains why the Z-itaconimide proceeds to the coumarin instead of returning to the E-intermediate.

**Figure 3:**
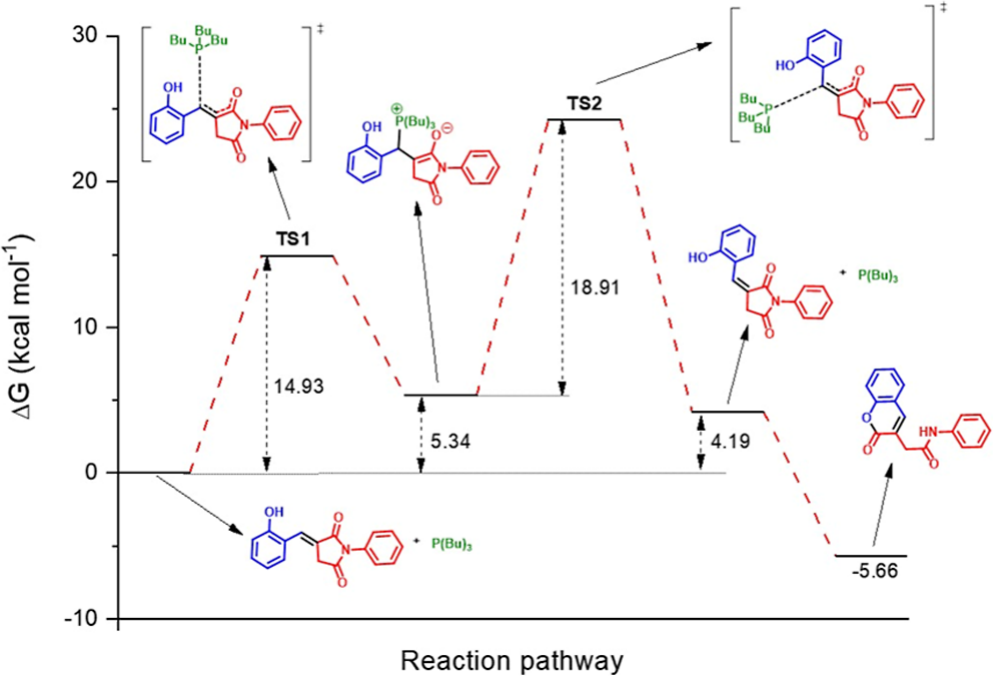
Density functional theory M06-2X/6-31G­(d)-calculated Gibbs free energy profiles for the itaconimide isomerization and intramolecular cyclization steps of the formal *oxa*-[4 + 2] cycloaddition.

The relatively high energetic demand associated with the isomerization/cyclization sequence is consistent with the experimental observations. Under room-temperature conditions, little-or-no coumarin formation was observed, with preferential formation of the corresponding itaconimide intermediate (see Scheme 2), indicating that microwave heating is essential to render the cyclization process kinetically accessible. Subtle energetic variations among different substrates likely contribute to the formation of the coumarin/itaconimide mixtures observed in some cases.

During the preparation of the reaction scope, we observed that some of the synthesized compounds were fluorescent, especially those prepared using 2-hydroxy-1-naphthaldehyde. Thus, the photophysical properties of selected coumarins were measured and the results are summarized in Table 1 and Figures S3 and S4. Stokes shifts were observed in the range of 51–114 nm and were, in general, large. Interestingly, the fluorescence quantum yields were very dependent on the coumarin substituents. For example, for *para*-substituted aryl groups (e.g., **4l**, **4m**, **4n**, **4o**, and **4p**), the values ranged from 1% to 5%. Two derivatives presented higher fluorescence quantum yield analogue **4p**, bearing a 2,4-dichloro substituent (28%), and product **4t** prepared using a *N*-alkyl maleimide (remarkably 84%).

**Table 1:** Ultraviolet–Visible and Fluorescence Data for Synthesized Coumarins^a^

coumarin	λ_max_ (abs) (nm)	absorbance (a.u.)	λ_max_ (em) (nm)^b^	emission curve area (F)	Stokes shift (nm)	fluorescence quantum yield (Φ*F*, %)
**4l**	354.97	0.1375	405.00	5783	50.03	5%
**4m**	355.07	0.0962	408.93	1251	53.93	2%
**4n**	354.03	0.1532	413.07	1425	59.04	1%
**4o**	324.93	0.2663	438.93	3847	114.00	2%
**4p**	355.07	0.1604	408.03	4657	52.96	4%
**4q**	355.01	0.2238	406.06	49,449	51.05	28%
**4t**	354.97	0.0979	406.96	58,533	51.99	84%

a Analyses were performed using 10 μM solutions in chloroform.

b Compounds were excited at the maximum absorption wavelength. The quantum yields were determined using a quinine sulfate solution as the standard (Φ*F* = 0.546 for a 0.5 M solution in H_2_SO_4_; absorbance = 0.045; area = 22,289); for details concerning these calculations, see the Supporting Information.

## Conclusions

The results of this study are in line with latest developments in phosphine-mediated processes,^34–37^ and a streamlined one-pot method for the synthesis of new 3-substituted coumarin–acetamide derivatives via a tributylphosphine-mediated reaction between maleimides and salicylaldehydes under microwave irradiation was hereby described. The method involves the sequential in situ phosphorus ylide formation, a Wittig reaction, E-itaconimide isomerization, and an intramolecular cyclization to afford the desired coumarins. The optimized conditions yielded the desired derivatives in up to a 68% yield, with no need of purification by chromatography. Additionally, the procedure was transposed to a continuous flow system, achieving yields up to 65% and enabling a scale-up to 3 mmol. Mechanistic studies and computational analysis shed light on the dual function of tributylphosphine, acting both in the phosphonium ylide formation and also as a catalyst for E-itaconimide isomerization. The resulting coumarins displayed significant fluorescence properties, with generally large Stokes shifts and variable fluorescence quantum yields (directly influenced by various substituents, reaching up to 84%). Finally, this method offers a highly direct, efficient, and scalable approach to the synthesis of 3-substituted coumarins, with potential applications in medicinal and organic chemistry and represents the first formal *oxa*-[4 + 2] cycloaddition involving maleimides.

## Experimental Section

### General Remarks

All purchased chemicals (including maleimides **1a**–**c** and aldehydes **2a**–**m**) were acquired from commercial sources and utilized as obtained, without any additional purification. Analytical Thin Layer Chromatography (TLC) was conducted on silica gel 60 F254 plates and visualized using a UV lamp. The melting points were determined using a hot-plate apparatus (Microquímica MQAPF 301). ^1^H and ^13^C­{^1^H} NMR spectra were acquired at frequencies of 500 and 125 MHz, respectively. Chemical shifts for ^1^H and ^13^C NMR are denoted as δ (parts per million), referenced to the solvent signal of CDCl_3_ (7.26 ppm—singlet for ^1^H and 77.0 ppm—triplet for ^13^C) or DMSO-*d*_6_ (2.50 ppm for ^1^H and 39.5 ppm for ^13^C). The reported chemical shifts follow these abbreviations: s for singlet, d for doublet, dd for doublet of doublets, dt for doublet of triplets, t for triplet, td for triplet of doublets, br for broad, and m for multiplet. High-resolution mass spectra (HRMS) were recorded using electron spray ionization (ESI) (hybrid linear ion trap–orbitrap FT-MS/MS and QqTOF Microtof-QII models). Microwave heating reactions were performed in a CEM Discover SP using the 10 mL Pyrex pressure vial for closed vessel reactions, under the indicated power automatically to reach and maintain the set temperature, specified in each case, with IR temperature control and medium stirring speed using cylindrical stir bars (10 × 3 mm) and a default ramp time of 10 min.

### General Procedure for the MW Synthesis of Coumarins **4a–4w**

Unless otherwise specified, the masses of 0.5 mmol of maleimide **1** (1.0 equiv) and 0.5 mmol of salicylaldehyde **2** (1.0 equiv) were added to a 10 mL microwave tube. Then, 5 mL of EtOH and 0.6 mmol of tributylphosphine (1.2 equiv) were added. The reaction was heated by microwave irradiation under conditions of 150 °C, 250 W, 250 psi, 2–4 h, and a heating ramp time of 2 min. Reaction progress was monitored by TLC, observing the consumption of maleimide and the formation of a precipitate. Upon completion, the solid product was collected by vacuum filtration and washed with 10 mL of cold ethanol to afford coumarins **4a**–**4w**. In cases where further purification was required, the solid was additionally washed with ethyl acetate. For this purpose, the mother liquor was carefully removed using a Pasteur pipet fitted with a cotton plug, and the solid was washed with approximately 5 mL portions of solvent. This washing process was repeated at least three times. Finally, the resulting solid was dried under reduced pressure to afford the desired product.

### General Procedure for Continuous-Flow Synthesis for Products **4b**, **4d**, **4e**, **4f**, **4r**, and **4aa**

A solution containing maleimide (0.5 mmol, 1.0 equiv) and salicylaldehyde (0.5 mmol, 1.0 equiv) was prepared in ethanol (7 mL, 0.07 mol L^–1^) in a 10 mL Erlenmeyer flask. The mixture was briefly sonicated to ensure complete dissolution and then transferred to a 9 mL sample loop using a syringe. For less soluble maleimides, the solution was heated to 75 °C prior to transfer and maintained at this temperature to avoid precipitation. In a separate 10 mL Erlenmeyer flask, a solution of tributylphosphine (0.8 mmol, 1.6 equiv) in ethanol (9 mL, 0.09 mol L^–1^) was prepared and transferred to a 13 mL sample loop using a syringe. After preparation, both loops were connected to the two pumps of the continuous-flow system and to a 4 bar back pressure regulator. The reaction was performed at a total flow rate of 316 μL min^–1^, with Pump A (phosphine solution) using a flow rate of 176 μL min^–1^ and pump B of 140 μL min^–1^. The streams were combined through a Y-connector equipped with a static mixer to ensure efficient mixing of the reagents and then passed through a 38 mL stainless-steel reactor maintained at 160 °C under a back pressure of 18 bar. The system was operated under continuous-flow conditions for 2 h. The outflowing reaction mixture was collected in a 120 mL Erlenmeyer flask, concentrated under reduced pressure, and subsequently placed in a freezer to induce precipitation of the product. The resulting solid was isolated by vacuum filtration and washed with 10 mL of cold ethanol to afford the coumarin derivative.

#### *N*-(4-Nitrophenyl)-2-(2-oxo-2H-chromen-3-yl)­acetamide (**4a**)

Two hours (72.3 mg, 41% yield), pearl-yellow solid, mp: 275 °C (decompose). ^1^H NMR (DMSO-*d*_6_, 500 MHz): δ 10.80 (s, NH, 1H), 8.23 (d, *J* = 9.0 Hz, Ar H, 2H), 8.04 (s, CH, 1H), 7.84 (d, *J* = 9.0 Hz, Ar H, 2H), 7.72 (d, *J* = 9.0 Hz, Ar H, 1H), 7.62–7.59 (t, *J* = 8.0 Hz, Ar H, 1H), 7.43 (d, *J* = 9.0 Hz, Ar H, 1H), 7.39–7.36 (t, *J* = 8.0 Hz, Ar H, 1H), 3.72 (s, CH_2_, 2H). ^13^C­{^1^H} NMR (DMSO-*d*_6,_ 125 MHz): δ 169.3, 161.2, 153.4, 145.7, 142.7, 142.7, 131.9, 128.6, 125.5, 125.2, 123.5, 119.5, 119.2, 116.6, 38.7. IR (KBr): *v*/cm^–1^ 3326, 3294, 3229, 3163, 3098, 1697, 1616, 1609, 1508, 1339, 1169, 1111, 1092, 864, 760. HRMS (ESI-TOF) *m*/*z*: [M + H]^+^ Calcd for C_17_H_12_N_2_O_5_, 325.0824; found, 325.0824.

#### *N*-(4-Chlorophenyl)-2-(2-oxo-2H-chromen-3-yl)­acetamide (**4b**)

Two hours (95.6 mg, 59% yield), pearl-white solid, mp: 257–258 °C (Lit.: 258–259 °C).^17^ ^1^H NMR (DMSO-*d*_6_, 500 MHz): δ 10.31 (s, NH, 1H), 8.01 (s, CH, 1H), 7.71 (d, *J* = 8.0 Hz, Ar H, 1H), 7.63–7.58 (m, Ar H, 3H), 7.42 (d, *J* = 8.0 Hz, Ar H, 1H), 7.38–7.35 (m, Ar H, 3H), 3.63 (s, CH_2_, 2H). ^13^C­{^1^H} NMR (DMSO-*d*_6_, 125 MHz): δ 167.8, 160.8, 152.9, 142.0, 138.1, 131.4, 128.7, 128.1, 126.8, 124.7, 123.4, 120.6, 119.1, 116.1, 38.0. IR (KBr): *v*/cm^–1^ 3333, 3291, 3201, 3121, 1713, 1697, 1609, 1547, 1489, 1400, 1246, 1169, 1084, 849, 818, 752.

#### 2-(2-Oxo-2H-chromen-3-yl)-*N*-(2,4,6-trichlorophenyl)­acetamide **(4c)**

0.3 mmol of **1**, 0.3 mmol of **2**, and 1.2 equiv of 0.4 mmol of tributylphosphine were mixed. Two hours (71.2 mg, 58% yield), pearl-white solid, mp: 292–294 °C. ^1^H NMR (DMSO-*d*_6_, 500 MHz): δ 10.08 (s, NH, 1H), 7.75 (s, Ar H, 2H), 7.71 (d, *J* = 8.0 Hz, Ar H, 1H), 7.61–7.58 (t, *J* = 8.0 Hz, Ar H, 1H), 7.41 (d, *J* = 6.0 Hz, Ar H, 1H), 7.38–7.35 (t, *J* = 8.0 Hz, Ar H, 1H), 3.66 (s, CH_2_, 2H). ^13^C­{^1^H} NMR (DMSO-*d*_6_, 125 MHz): δ 167.7, 160.5, 152.9, 142.1, 134.5, 132.4, 132.3, 131.4, 128.3, 128.1, 124.6, 123.0, 119.1, 116.0, 36.7. IR (KBr): *v*/cm^–1^ 3221, 3183, 3067, 3005, 1724, 1713, 1670, 1640, 1606, 1562, 1520, 1458, 1385, 1358, 1215, 1192, 1072, 952, 856, 822. HRMS (ESI-TOF) *m*/*z*: [M + H]^+^ Calcd for C_17_H_10_Cl_3_NO_3_, 381.9805; found, 381.9798.

#### 2-(2-Oxo-2H-chromen-3-yl)-*N*-phenylacetamide **(4d)**

Two hours (81.4 mg, 44% yield), pearl-white solid, mp: 205–206 °C (Lit.: 210–211 °C).^17^ ^1^H NMR (DMSO-*d*_6_, 500 MHz): δ 10.17 (s, NH, 1H), 8.01 (s, CH, 1H), 7.72 (d, *J* = 8.0 Hz, Ar H, 1H), 7.61–7.59 (m, Ar H, 3H), 7.42 (d, *J* = 8.0 Hz, Ar H, 1H), 7.38–7.35 (t, *J* = 8.0 Hz, Ar H, 1H), 7.32–7.29 (m, Ar H, 2H), 7.06–7.03 (t, *J* = 7.0 Hz, Ar H, 1H), 3.64 (s, CH_2_, 2H). ^13^C­{^1^H} NMR (DMSO-*d*_6_, 125 MHz): δ 167.6, 160.8, 152.9, 141.9, 139.1, 131.3, 128.7, 128.0, 124.6, 123.6, 123.2, 119.1, 119.1, 38.0. IR (KBr): *v*/cm^–1^ 3337, 1717, 1694, 1601, 1547, 1493, 1443, 1346, 1250, 1076, 752, 702.

#### *N*-(2,4-Dichlorophenyl)-2-(2-oxo-2H-chromen-3-yl)­acetamide **(4e)**

Two hours (101.1 mg, 53% yield), pearl-white solid, mp: 201–203 °C. ^1^H NMR (DMSO-*d*_6_, 500 MHz): δ 9.82 (s, NH, 1H), 8.02 (s, CH, 1H), 7.76 (d, *J* = 9.0 Hz, Ar H, 1H), 7.72–7.70 (d, *J* = 8.0 Hz e *J* = 3.0 Hz, Ar H, 1H), 7.66 (d, *J* = 3.0 Hz, Ar H, 1H), 7.61–7.58 (m, Ar H, 1H), 7.43–7.35 (m, Ar H, 3H), 3.72 (s, CH_2_, 2H). ^13^C­{^1^H} NMR (DMSO-*d*_6_, 125 MHz): δ 168.3, 160.8, 152.9, 142.0, 134.1, 131.4, 129.4, 129.0, 128.1, 127.5, 124.6, 123.3, 123.3, 37.6. IR (KBr): *v*/cm^–1^ 3237, 3090, 3024, 1724, 1709, 1659, 1609, 1578, 1524, 1474, 1385, 1281, 1204, 1192, 1072, 860, 826, 760. HRMS (ESI-TOF) *m*/*z*: [M + H]^+^ Calcd for C_17_H_11_Cl_2_NO_3_, 348.0194; found, 348.0189.

#### 2-(2-Oxo-2H-chromen-3-yl)-*N*-(*p*-tolyl)­acetamide **(4f)**

Two hours and 30 min (76.1 mg, 50% yield), white solid, mp: 210–211 °C. ^1^H NMR (DMSO-*d*_6_, 500 MHz): δ 10.07 (s, NH, 1H), 7.99 (s, CH, 1H), 7.71 (d, *J* = 8.0 Hz, Ar H, 1H), 7.61–7.58 (t, *J* = 8.0 Hz, Ar H, 1H), 7.48 (d, *J* = 9.0 Hz, Ar H, 2H), 7.41 (d, *J* = 8.0 Hz, Ar H, 1H), 7.37–7.34 (t, *J* = 8.0 Hz, Ar H, 1H), 7.10 (d, *J* = 9.0 Hz, Ar H, 2H), 3.61 (s, CH_2_, 2H), 2.24 (s, CH_3_, 3H). ^13^C­{^1^H} NMR (DMSO-*d*_6_, 125 MHz): δ 167.4, 160.8, 152.9, 141.9, 136.7, 132.1, 131.3, 129.1, 128.0, 124.6, 123.6, 119.1, 116.0, 38.0, 20.4. IR (KBr): *v*/cm^–1^ 3333, 1713, 1690, 1609, 1547, 1516, 1458, 1345, 1246, 1192, 1177, 1092, 822, 760. HRMS (ESI-TOF) *m*/*z*: [M + H]^+^ Calcd for C_18_H_15_NO_3_, 294.1130; found, 294.1126.

#### *N*-(1,5-Dimethyl-3-oxo-2-phenyl-2,3-dihydro-1H-pyrazol-4-yl)-2-(2-oxo-2H-chromen-3-yl)­acetamide **(4g)**

Two hours (48.6 mg, 22% yield), white solid, mp: 231–233 °C. ^1^H NMR (DMSO-*d*_6_, 500 MHz): δ 9.30 (s, NH, 1H), 8.01 (s, CH, 1H), 7.70–7.65 (m, Ar H, 8H), 3.58 (s, CH_2_, 2H), 3.04 (s, CH_3_, 3H), 2.12 (s, CH_3_, 3H). ^13^C­{^1^H} NMR (DMSO-*d*_6_, 125 MHz): δ 168.0, 161.7, 160.5, 152.7, 152.3, 141.4, 135.0, 131.0, 128.9, 127.8, 126.0, 124.4, 123.6, 123.4, 119.0, 118.8, 107.4, 36.9, 35.9, 10.9. IR (KBr): *v*/cm^–1^ 3410, 3183, 3005, 1709, 1674, 1651, 1608, 1589, 1493, 1296, 1223, 1076, 760. HRMS (ESI-TOF) *m*/*z*: [M + H]^+^ Calcd for C_22_H_19_N_3_O_4_, 390.1454; found, 390.1455.

#### *N*-(2,4-Dichlorophenyl)-2-(8-methoxy-2-oxo-2H-chromen-3-yl)­acetamide **(4h)**

Two hours (128.2 mg 68% yield), white solid, mp: 215–217 °C. ^1^H NMR (DMSO-*d*_6_, 500 MHz): δ 9.81 (s, NH, 1H), 7.99 (s, CH, 1H), 7.75 (d, *J* = 9.0 Hz, Ar H, 1H), 7.65 (d, *J* = 2.5 Hz, Ar H, 1H), 7.42–7.39 (d, *J* = 9.0 Hz, *J* = 2.5 Hz, Ar H, 1H), 7.29–7.23 (m, Ar H, 3H), 3.91 (s, OMe, 3H), 3.71 (s, CH_2_, 2H). ^13^C­{^1^H} NMR (DMSO-*d*_6_, 125 MHz): δ 168.3, 160.5, 164.4, 142.2, 142.2, 134.1, 129.4, 128.9, 127.5, 127.2, 127.0, 124.6, 123.5, 119.6, 119.2, 56.1, 37.6. IR (KBr): *v*/cm^–1^ 3294, 1724, 1667, 1582, 1528, 1477, 1385, 1281, 1200, 1107, 976, 868, 783. HRMS (ESI-TOF) *m*/*z*: [M + H]^+^ Calcd for C_18_H_13_Cl_2_NO_4_, 378.0294; found, 378.0290.

#### 2-(8-Methoxy-2-oxo-2H-chromen-3-yl)-*N*-(4-methoxyphenyl)­acetamide **(4i)**

0.3 mmol of **1**, 0.3 mmol of **2**, and 1.2 equiv of 0.4 mmol of tributylphosphine were mixed. 2.5 h (51.1 mg, 50% yield), white solid, mp: 229 °C (decompose). ^1^H NMR (DMSO-*d*_6_, 500 MHz): δ 10.01 (s, NH, 1H), 7.96 (s, CH, 1H), 7.50 (d, *J* = 9.0 Hz, Ar H, 2H), 7.31–7.24 (m, Ar H, 3H), 6.87 (d, *J* = 9.0 Hz, Ar H, 2H), 3.91 (s, OMe, 3H), 3.71 (s, OMe, 3H), 3.59 (s, CH_2_, 2H). ^13^C­{^1^H} NMR (DMSO-*d*_6_, 125 MHz): δ 167.0, 160.5, 155.2, 146.4, 142.2, 142.1, 132.3, 124.5, 123.8, 120.6, 119.7, 119.2, 113.8, 113.6, 56.1, 55.1, 37.8. IR (KBr): *v*/cm^–1^ 3283, 3127, 3057, 2936, 2837, 1724, 1610, 1541, 1512, 1481, 1458, 1273, 1244, 1107, 1061, 1028, 829, 734. HRMS (ESI-TOF) *m*/*z*: [M + H]^+^ Calcd for C_19_H_17_NO_5_, 340.1185; found, 340.1185.

#### 2-(6-Allyl-8-methoxy-2-oxo-2H-chromen-3-yl)-*N*-(4-nitrophenyl)­acetamide **(4j)**

0.4 mmol of **1**, 0.4 mmol of **2**, and 1.2 equiv of 0.5 mmol of tributylphosphine were mixed. Two hours (67.3 mg, 42% yield), pearl-yellow solid, mp: 270–273 °C. ^1^H NMR (DMSO-*d*_6_, 500 MHz): δ 10.79 (s, NH, 1H), 8.22 (d, *J* = 9.0 Hz, Ar H, 2H), 7.96 (s, CH, 1H), 7.83 (d, *J* = 9.0 Hz, Ar H, 2H), 7.12 (s, Ar H, 1H), 7.05 (s, Ar H, 1H), 6.04–5.96 (m, CH, 1H), 5.14–5.08 (m, =CH_2_, 2H), 3.90 (s, OMe, 3H), 3.69 (s, CH_2_, 2H), 3.43 (d, *J* = 7.0 Hz, CH_2_, 2H). ^13^C­{^1^H} NMR (DMSO-*d*_6_, 125 MHz): δ 168.8, 160.6, 146.3, 145.2, 142.4, 142.2, 140.7, 137.2, 136.5, 125.0, 123.3, 119.4, 118.8, 118.4, 116.3, 114.2, 56.1, 39.0, 38.3. IR (KBr): *v*/cm^–1^ 3323, 3293, 3226, 3161, 3096, 3011, 2974, 2938, 1701, 1618, 1589, 1570, 1558, 1508, 1494, 1485, 1463, 1437, 1339, 1256, 1168, 1130, 1109, 1003, 860, 750, 694. HRMS (ESI-TOF) *m*/*z*: [M + H]^+^ Calcd for C_21_H_18_N_2_O_6_, 395.1243; found, 395.1241.

#### 2-(6-Allyl-8-methoxy-2-oxo-2H-chromen-3-yl)-*N*-(2,4-dichlorophenyl)­acetamide **(4k)**

Two hours (121.2 mg, 63% yield), pearl-yellow solid, mp: 203–204 °C. ^1^H NMR (DMSO-*d*_6_, 500 MHz): δ 9.71 (s, NH, 1H), 7.94 (s, CH, 1H), 7.76 (d, *J* = 9.0 Hz, Ar H, 1H), 7.63 (d, *J* = 2.0 Hz, Ar H, 1H), 7.41–7.39 (dd, *J* = 9.0 Hz, *J* = 2.0 Hz, Ar H, 1H), 7.11 (s, Ar H, 1H), 7.05 (s, Ar H, 1H), 6.03–5.98 (m, CH, 1H), 5.15–5.08 (m, =CH_2_, 2H), 3.91 (s, OMe, 3H), 3.7 (s, CH_2_, 2H), 3.43 (d, *J* = 7.0 Hz, CH_2_, 2H). ^13^C­{^1^H} NMR (DMSO-*d*_6_, 125 MHz): δ 168.1, 160.4, 146.2, 141.9, 140.7, 137.0, 136.4, 134.0, 129.2, 128.7, 127.4, 126.9, 123.3, 119.4, 118.3, 116.1, 114.2, 56.0, 38.8, 37.4. IR (KBr): *v*/cm^–1^ 3237, 3071, 2967, 2943, 2847, 1728, 1713, 1667, 1589, 1524, 1385, 1130, 1199, 907. HRMS (ESI-TOF) *m*/*z*: [M + H]^+^ Calcd for C_21_H_17_Cl_2_NO_4_, 418.0613; found, 418.0613.

#### 2-(3-Oxo-3H-benzo­[f]­chromen-2-yl)-*N*-phenylacetamide **(4l)**

Washing with ethyl acetate is required, 2 h (77.6 mg, 47% yield), light-yellow solid, mp: 274–276 °C. ^1^H NMR (DMSO-*d*_6_, 500 MHz): δ 10.21 (s, NH, 1H), 8.90 (s, CH, 1H), 8.49 (d, *J* = 9.0 Hz, Ar H, 1H), 8.15 (d, *J* = 9.0 Hz, Ar H, 1H), 8.05 (d, *J* = 9.0 Hz, Ar H, 1H), 7.76–7.732 (t, *J* = 7.0 Hz, Ar H, 1H), 7.64–7.56 (m, Ar H, 4H), 7.32–7.29 (t, *J* = 8.0 Hz, Ar H, 2H), 7.06–7.03 (t, *J* = 7.0 Hz, Ar H, 1H), 3.75 (s, CH_2_, 2H). ^13^C­{^1^H} NMR (DMSO-*d*_6_, 125 MHz): δ 167.8, 160.9, 152.4, 139.2, 138.5, 132.5, 130.0, 128.9, 128.8, 128.6, 128.3, 126.1, 123.3, 122.9, 122.1, 119.1, 116.6, 113.1, 38.4. ^135^DEPT NMR (DMSO-*d*_6_, 125 MHz): δ 138.3, 132.3, 128.7, 128.5, 128.0, 125.8, 123.0, 121.9, 118.8, 116.3, 38.1. IR (KBr): *v*/cm^–1^ 3271, 3132, 3063, 1721, 1655, 1639, 1597, 1578, 1531, 1443, 1354, 1258, 1207, 1076, 995, 818, 752. HRMS (ESI-TOF) *m*/*z*: [M + H]^+^ Calcd for C_21_H_15_NO_3_, 330.1130; found, 330.1129.

#### 2-(3-Oxo-3H-benzo­[f]­chromen-2-yl)-*N*-(*p*-tolyl)­acetamide **(4m)**

Washing with ethyl acetate is required, 2 h (76.2 mg, 43% yield), white-pearl solid, mp: 255–256.7 °C. ^1^H NMR (DMSO-*d*_6_, 500 MHz): δ 10.11 (s, NH, 1H), 8.89 (s, CH, 1H), 8.49 (d, *J* = 9.0 Hz, Ar H, 1H), 8.15 (d, *J* = 9.0 Hz, Ar H, 1H), 8.05 (d, *J* = 8.0 Hz, Ar H, 1H), 7.76–7.73 (t, *J* = 8.0 Hz, Ar H, 1H), 7.63–7.60 (t, *J* = 8.0 Hz, Ar H, 1H), 7.57 (d, *J* = 9.0 Hz, Ar H, 1H), 7.50 (d, *J* = 8.0 Hz, Ar H, 2H), 7.10 (d, *J* = 8.0 Hz, Ar H, 2H), 3.73 (s, CH_2_, 2H), 2.24 (s, CH_3_, 3H). ^13^C­{^1^H} NMR (DMSO-*d*_6_, 125 MHz): δ 167.5, 160.8, 152.4, 138.5, 136.7, 132.5, 132.1, 130.0, 129.1, 128.9, 128.6, 128.2, 126.0, 123.0, 122.1, 119.1, 116.5, 113.1, 38.3, 20.4. IR (KBr): *v*/cm^–1^ 3279, 3125, 3036, 2920, 2866, 1721, 1709, 1655, 1574, 1524, 1404, 1350, 1254, 1207, 1076, 995, 953, 934, 818, 779. HRMS (ESI-TOF) *m*/*z*: [M + H]^+^ Calcd for C_22_H_17_NO_3_, 344.1287; found, 344.1282.

#### *N*-(4-Methoxyphenyl)-2-(3-oxo-3H-benzo­[f]­chromen-2-yl)­acetamide **(4n)**

Washing with ethyl acetate is required, 2 h (92.8 mg, 48% yield), white-pearl solid, mp: 241–244.4 °C. ^1^H NMR (DMSO-*d*_6_, 500 MHz): δ 10.05 (s, NH, 1H), 8.90 (s, CH, 1H), 8.49 (d, *J* = 8.0 Hz, Ar H, 1H), 8.15 (d, *J* = 9.0 Hz, Ar H, 1H), 8.05 (d, *J* = 8.0 Hz, Ar H, 1H), 7.76–7.73 (t, *J* = 8.0 Hz, Ar H, 1H), 7.64–7.61 (t, *J* = 8.0 Hz, Ar H, 1H), 7.57 (d, *J* = 9.0 Hz, Ar H, 1H), 7.52 (d, *J* = 9.0 Hz, Ar H, 2H), 6.88 (d, *J* = 9.0 Hz, Ar H, 2H), 3.71 (s, OMe/CH_2_, 5H). ^13^C­{^1^H} NMR (DMSO-*d*_6_, 125 MHz): δ 167.2, 160.8, 155.2, 152.4, 138.4, 132.4, 132.3, 130.0, 128.9, 128.6, 128.2, 126.0, 123.0, 122.1, 120.7, 116.5, 113.8, 113.1, 55.1, 38.2. IR (KBr): *v*/cm^–1^ 3275, 3075, 2924, 1721, 1713, 1655, 1577, 1513, 1466, 1439, 1408, 1250, 1223, 1076, 1034, 833, 818, 783. HRMS (ESI-TOF) *m*/*z*: [M + H]^+^ Calcd for C_22_H_17_NO_4_, 360.1236; found, 360.1224.

#### *N*-(4-Nitrophenyl)-2-(3-oxo-3H-benzo­[f]­chromen-2-yl)­acetamide **(4o)**

Two hours (75.6 mg, 25% yield), yellow solid, mp: 257 °C (decompose). ^1^H NMR (DMSO-*d*_6_, 500 MHz): δ 10.84 (s, NH, 1H), 8.95 (s, CH, 1H), 8.50 (d, *J* = 9.0 Hz, Ar H, 1H), 8.23 (d, *J* = 9.0 Hz, Ar H, 2H), 8.17 (d, *J* = 9.0 Hz, Ar H, 1H), 8.07 (d, *J* = 8.0 Hz, Ar H, 1H), 7.85 (d, *J* = 9.0 Hz, Ar H, 2H), 7.77–7.74 (t, *J* = 8.0 Hz, Ar H, 1H), 7.65–7.62 (t, *J* = 8.0 Hz, Ar H, 1H), 7.59 (d, *J* = 9.0 Hz, Ar H, 1H), 3.83 (s, CH_2_, 2H). ^13^C­{^1^H} NMR (DMSO-*d*_6_, 125 MHz): δ 168.9, 160.8, 152.5, 145.2, 142.2, 138.8, 132.6, 130.0, 128.9, 128.6, 128.3, 126.1, 125.0, 122.4, 122.1, 118.8, 116.6, 113.1, 38.6. IR (KBr): *v*/cm^–1^ 3321, 3291, 3224, 3159, 3094, 1717, 1686, 1616, 1570, 1508, 1408, 1335, 1300, 1261, 1207, 1165, 1099, 856, 810. HRMS (ESI-TOF) *m*/*z*: [M + H]^+^ Calcd for C_21_H_14_N_2_O_5_, 375.0981; found, 375.0975.

#### *N*-(4-Chlorophenyl)-2-(3-oxo-3H-benzo­[f]­chromen-2-yl)­acetamide **(4p)**

Washing with ethyl acetate is required, 2 h (84.1 mg, 45% yield), light-yellow solid, mp: 258.5 °C (decompose). ^1^H NMR (DMSO-*d*_6_, 500 MHz): δ 10.35 (s, NH, 1H), 8.91 (s, CH, 1H), 8.48 (d, *J* = 9.0 Hz, Ar H, 1H), 8.32 (d, *J* = 9.0 Hz, Ar H, 1H), 8.06 (d, *J* = 8.0 Hz, Ar H, 1H), 7.76–7.73 (t, *J* = 8.0 Hz, Ar H, 1H), 7.65–7.61 (m, Ar H, 3H), 7.57 (d, *J* = 9.0 Hz, Ar H, 1H), 7.36 (d, *J* = 9.0 Hz, Ar H, 2H), 3.75 (s, CH_2_, 2H). ^13^C­{^1^H} NMR (DMSO-*d*_6_, 125 MHz): δ 167.9, 160.8, 152.4, 138.6, 138.1, 132.5, 130.0, 128.9, 128.7, 128.6, 128.3, 126.8, 126.1, 122.7, 122.1, 120.7, 116.6, 113.1, 38.4. IR (KBr): *v*/cm^–1^ 3275, 3113, 3067, 1721, 1709, 1593, 1574, 1524, 1493, 1400, 1342, 1211, 1092, 1076, 1015, 995, 818, 779. HRMS (ESI-TOF) *m*/*z*: [M + H]^+^ Calcd for C_21_H_14_ClNO_3_, 364.0740; found, 364.0731.

#### *N*-(2,4-Dichlorophenyl)-2-(3-oxo-3H-benzo­[f]­chromen-2-yl)­acetamide **(4q)**

Partially soluble in DMSO-*d*_6_. Two hours (102.7 mg, 51% yield), pearl-yellow solid, mp: 260–262 °C. ^1^H NMR (DMSO-*d*_6_, 500 MHz): δ 9.85 (s, NH, 1H), 8.95 (s, Ar H, 1H), 8.51 (s, Ar H, 1H), 8.18 (s, Ar H, 1H), 8.08 (s, Ar H, 1H), 7.77–7.42 (m, Ar H, 6H), 3.83 (s, CH_2_, 2H). IR (KBr): *v*/cm^–1^ 3221, 1705, 1655, 1578, 1524, 1473, 1381, 1285, 1215, 1103, 1072, 880, 822. HRMS (ESI-TOF) *m*/*z*: [M + H]^+^ Calcd for C_21_H_13_Cl_2_NO_3_, 398.0351; found, 398.0347.

#### 2-(3-Oxo-3H-benzo­[f]­chromen-2-yl)-*N*-(2,4,6-trichlorophenyl)­acetamide **(4r)**

0.3 mmol of **1**, 0.3 mmol of **2**, and 1.2 equiv of 0.4 mmol of tributylphosphine were mixed. Two hours (71.2 mg, 56% yield), pearl-white solid, mp: 265–269.7 °C. ^1^H NMR (DMSO-*d*_6_, 500 MHz): δ 10.11 (s, NH, 1H), 8.94 (s, C–H, 1H), 8.49 (d, *J* = 8.0 Hz, Ar H, 1H), 8.16 (d, *J* = 9.0 Hz, Ar H, 1H), 8.06 (d, *J* = 8.0 Hz, Ar H, 1H), 7.77–7.75 (m, Ar H, 3H), 7.64–7.11 (t, *J* = 8.0 Hz, Ar H, 1H), 7.59 (d, *J* = 9.0 Hz, Ar H, 1H), 3.78 (s, CH_2_, 2H). ^13^C­{^1^H} NMR (DMSO-*d*_6_, 125 MHz): δ 167.8, 160.5, 152.5, 138.4, 134.5, 132.5, 132.4, 129.9, 128.6, 128.2, 126.0, 122.4, 122.0, 116.6, 113.1, 37.13. IR (KBr): *v*/cm^–1^ 3225, 3063, 3005, 1721, 1670, 1636, 1574, 1516, 1451, 1369, 1273, 1215, 1072, 995, 856, 818. HRMS (ESI-TOF) *m*/*z*: [M + H]^+^ Calcd for C_21_H_12_Cl_3_NO_3_, 431.9961; found, 431.9959.

#### *N*-(1,5-Dimethyl-3-oxo-2-phenyl-2,3-dihydro-1H-pyrazol-4-yl)-2-(3-oxo-3H-benzo­[f]­chromen-2-yl)­acetamide **(4s)**

Two hours (26.9 mg, 12% yield), pearl-yellow solid, mp: 267–268.9 °C. ^1^H NMR (DMSO-*d*_6_, 500 MHz): δ 9.21 (s, NH, 1H), 8.91 (s, CH, 1H), 8.53 (d, *J* = 9.0 Hz, Ar H, 1H), 8.16 (d, *J* = 9.0 Hz, Ar H, 1H), 8.06 (d, *J* = 8.0 Hz, Ar H, 1H), 7.76–7.73 (t, *J* = 8.0 Hz, Ar H, 1H), 7.64–7.61 (t, *J* = 8.0 Hz, Ar H, 1H), 7.58 (d, *J* = 9.0 Hz, Ar H, 1H), 7.52–7.48 (t, *J* = 8.0 Hz, Ar H, 1H), 7.36 (d, *J* = 8.0 Hz, Ar H, 1H), 7.33–7.30 (t, *J* = 7.0 Hz, Ar H, 1H), 3.70 (s, CH_2_, 2H), 3.05 (s, CH_3_, 3H), 2.15 (s, CH_3_, 3H). ^13^C­{^1^H} NMR (DMSO-*d*_6_, 125 MHz): δ 168.2, 161.7, 160.5, 152.3, 152.2, 137.8, 135.1, 132.2, 129.9, 129.8, 128.9, 128.7, 128.0, 126.0, 125.8, 123.3, 123.0, 122.0, 122.2, 116.4, 113.0, 107.4, 37.3, 36.0, 11.0. IR (KBr): *v*/cm^–1^ 3260, 3055, 1717, 1667, 1636, 1535, 1342, 1300, 1223, 1080, 814. HRMS (ESI-TOF) *m*/*z*: [M + H]^+^ Calcd for C_26_H_21_N_3_O_4_, 440.1610; found, 440.1612.

#### 2-(2-(3-Oxo-3H-benzo­[f]­chromen-2-yl)­acetamido)­ethyl acetate **(4t)**

Washing with ethyl acetate is required, 2 h (35.3 mg, 25% yield), yellow-pearl solid, mp: 188–189.5 °C. ^1^H NMR (DMSO-*d*_6_, 500 MHz): δ 8.82 (s, NH, 1H), 8.48 (d, *J* = 8.5 Hz, Ar H, 1H), 8.20–8.18 (t, *J* = 6.0 Hz, CH, 1H), 8.15 (d, *J* = 9.0 Hz, Ar H, 1H), 8.05 (d, *J* = 8.0 Hz, Ar H, 1H), 7.76–7.73 (t, *J* = 8.0 Hz, Ar H, 1H), 7.63–7.60 (t, *J* = 8.0 Hz, Ar H, 1H), 7.56 (d, *J* = 9.0 Hz, Ar H, 1H), 4.04–4.02 (t, *J* = 6.0 Hz, CH_2_, 2H), 3.50 (s, CH_2_, 2H), 3.35–3.31 (m, 2H/H_2_O and 2H/CH_2_, 4H), 2.00 (s, CH_3_, 3H). ^13^C­{^1^H} NMR (DMSO-*d*_6_, 125 MHz): δ 170.8, 169.5, 161.2, 152.8, 138.6, 138.5, 132.9, 130.4, 129.3, 129.1, 128.7, 126.5, 123.5, 122.6, 117.0, 113.6, 62.9, 38.3, 37.9, 21.2. IR (KBr): *v*/cm^–1^ 3279, 3078, 2955, 1721, 1709, 1640, 1578, 1558, 1439, 1416, 1358, 1246, 1227, 1096, 1076, 1049, 995, 968, 822. HRMS (ESI-TOF) *m*/*z*: [M + H]^+^ Calcd for C_19_H_17_NO_5_, 340.1185; found, 340.1175.

#### *N*-Benzyl-2-(3-oxo-3H-benzo­[f]­chromen-2-yl)­acetamide **(4u)**

Two hours (49.5 mg, 31% yield), light-yellow solid, mp: 254–256.9 °C. ^1^H NMR (DMSO-*d*_6_, 500 MHz): δ 8.81 (s, NH, 1H), 8.50–8.48 (t, *J* = 6.0 Hz, CH, 1H), 8.44 (d, *J* = 9.0 Hz, Ar H, 1H), 8.14 (d, *J* = 9.0 Hz, Ar H, 1H), 8.04 (d, *J* = 9.0 Hz, Ar H, 1H), 7.76–7.73 (t, *J* = 8.0 Hz, Ar H, 1H), 7.63–7.60 (t, *J* = 8.0 Hz, Ar H, 1H), 7.56 (d, *J* = 9.0 Hz, Ar H, 1H), 7.34 (m, Ar H, 5H), 4.34 (d, *J* = 6.0 Hz, CH_2_, 2H), 3.58 (s, CH_2_, 2H). ^13^C­{^1^H} NMR (DMSO-*d*_6_, 125 MHz): δ 168.6, 160.6, 152.3, 139.4, 137.9, 132.2, 129.8, 128.7, 128.5, 128.1, 128.0, 127.1, 126.6, 125.8, 123.0, 121.9, 116.4, 113.1, 42.2, 37.4. IR (KBr): *v*/cm^–1^ 3294, 3075, 3028, 2916, 1717, 1643, 1574, 1547, 1354, 1219, 1076, 818, 733. HRMS (ESI-TOF) *m*/*z*: [M + H]^+^ Calcd for C_22_H_17_NO_3_, 344.1287; found, 344.1282.

#### 2-(2,8-Dioxo-2H,8H-pyrano­[2,3-*f*]­chromen-9-yl)-*N*-(4-nitrophenyl)­acetamide **(4v)**

A short column silica gel chromatography was performed using ethyl acetate as the mobile phase. The eluate was concentrated and stored in a freezer to induce product precipitation; subsequently, the resulting solid was isolated and dried by rotary evaporation. o Two hours (66.9 mg, 33% yield), orange solid, mp: >300 °C. ^1^H NMR (DMSO-*d*_6_, 500 MHz): δ 10.82 (s, NH, 1H), 8.38 (s, CH, 1H), 8.22 (d, *J* = 10.0 Hz, Ar H, 2H), 8.13 (d, *J* = 10.0 Hz, Ar H, 1H), 7.91 (d, *J* = 9.0 Hz, Ar H, 1H), 7.83 (d, *J* = 10.0 Hz, Ar H, 2H), 7.40 (d, *J* = 9.0 Hz, Ar H, 1H), 6.54 (d, *J* = 10.0 Hz, Ar H, 1H), 3.84 (s, CH_2_, 2H). ^13^C­{^1^H} NMR (DMSO-*d*_6_, 125 MHz): δ 169.1, 160.4, 159.6, 155.2, 150.2, 145.6, 144.7, 142.7, 135.4, 131.2, 125.5, 124.3, 119.2, 115.6, 115.3, 113.2, 108.5, 38.6. IR (KBr): *v*/cm^–1^ 3310, 3159, 3086, 1732, 1717, 1624, 1597, 1570, 1558, 1504, 1408, 1335, 1300, 1258, 1165, 1115, 1015, 853, 779. HRMS (ESI-TOF) *m*/*z*: [M + H]^+^ Calcd for C_20_H_12_N_2_O_7_, 393.0723; found, 393.0723.

#### *N*,*N*′-(1,2-Phenylene)­bis­(2-(2-oxo-2H-chromen-3-yl)­acetamide) **(4w)**

0.3 mmol of **1**, 0.8 mmol of **2**, and 1.2 equiv of 0.9 mmol of tributylphosphine were mixed. Two hours (45.7 mg, 24% yield), beige solid, mp: 266 °C (decompose). ^1^H NMR (DMSO-*d*_6_, 500 MHz): δ 9.46 (s, NH, 1H), 8.01 (s, CH, 1H), 7.68 (d, *J* = 8.0 Hz, Ar H, 1H), 7.60–7.55 (m, Ar H, 2H), 7.40 (d, *J* = 8.0 Hz, Ar H, 1H), 7.36–7.32 (t, *J* = 8.0 Hz, Ar H, 1H), 7.18–7.16 (m, Ar H, 1H), 3.65 (s, CH_2_, 2H). ^13^C­{^1^H} NMR (DMSO-*d*_6_, 125 MHz): δ 168.1, 160.9, 152.9, 142.0, 131.4, 130.5, 128.0, 125.2, 124.8, 124.6, 123.3, 119.1, 116.0, 38.0. IR (KBr): *v*/cm^–1^ 3300, 6035, 1717, 1686, 1641, 1608, 1516, 1456, 1063, 754. HRMS (ESI-TOF) *m*/*z*: [M + H]^+^ Calcd for C_28_H_20_N_2_O_6_, 481.1400; found, 481.1398.

#### Description to the Mixture of **(4aa + 4aa′)–(4ai + 4ai′)**

##### *N*-(4-Methoxyphenyl)-2-(2-oxo-2H-chromen-3-yl)­acetamide **(4aa)** Compound with (E)-3-(2-hydroxybenzylidene)-1-(4-methoxyphenyl)­pyrrolidine-2,5-dione **(4aa′)** (3:1)

2 h (88.6 mg, 57% Yield), pearl-white solid, mp: 186–187 °C. ^1^H NMR (DMSO-*d*_6_, 500 MHz): δ major isomer 10.01 (s, NH coumarin, 1H), 7.99 (s, CH coumarin, 1H), 7.72–7.71 (d, *J* = 8.0 Hz, *J* = 1.0 Hz, Ar H coumarin, 1H), 7.61–7.49 (m, Ar H, 4H), 7.42–7.42 (d, *J* = 8.0 Hz coumarin, 1H), 7.38–7.34 (t, *J* = 8.0 Hz, Ar H coumarin, 1H), 6.97–6.87 (m, Ar H, 4H), 3.71 (s, OMe, 3H), 3.59 (s, CH_2_ coumarin, 2H). ^13^C­{^1^H} NMR (DMSO-*d*_6_, 125 MHz): δ 167.6, 161.3, 155.7, 153.3, 142.3, 132.8, 131.9, 128.5, 124.1, 123.9, 121.0, 119.6, 116.4, 114.3, 55.6, 38.4. IR (KBr): *v*/cm^–1^ 3291, 3198, 3075, 2997, 2954, 2913, 2832, 1771 (C=O of itaconimide), 1713 (C=O of coumarin), 1686, 1651, 1605, 1543, 1516, 1454, 1412, 1238, 1188, 1072, 1030, 960, 822, 756.

##### 2-(8-Methoxy-2-oxo-2H-chromen-3-yl)-*N*-(4-nitrophenyl)­acetamide **(4ab)** Compound with (E)-3-(2-hydroxy-3-methoxybenzylidene)-1-(4-nitrophenyl)­pyrrolidine-2,5-dione **(4ab′)** (8:1)

2 h (91.0 mg, 45% Yield), dark-yellow solid, mp: 290 °C (decompose). ^1^H NMR (DMSO-*d*_6_, 500 MHz): δ major isomer 10.80 (s, NH, 1H), 8.22 (d, *J* = 9.0 Hz, Ar H, 2H), 8.01 (s, CH, 1H), 7.84 (d, *J* = 9.0 Hz, Ar H, 2H), 7.36–7.25 (m, Ar H, 3H), 3.92 (s, OMe, 3H), 3.71 (s, CH_2_, coumarin, 2H). ^13^C­{^1^H} NMR (DMSO-*d*_6_, 125 MHz): δ 168.7, 160.5, 146.4, 145.2, 142.5, 142.2, 127.9, 125.0, 124.1, 123.2, 119.6, 118.8, 113.8, 56.1, 38.2. IR (KBr): *v*/cm^–1^ 3325, 3294, 3229, 3159, 3003, 2943, 2843, 1701 (C=O coumarin), 1616, 1574, 1504, 1481, 1438, 1339, 1277, 1258, 1169, 1111, 880, 741.

##### 2-(6-Chloro-2-oxo-2H-chromen-3-yl)-*N*-(4-methoxyphenyl)­acetamide **(4ac)** Compound with (E)-3-(5-Chloro-2-hydroxybenzylidene)-1-(4-methoxyphenyl)­pyrrolidine-2,5-dione **(4ac′)** (1.7:1)

Four hours (90.9 mg, 53% yield), pearl-yellow solid, mp: 199–201 °C. ^1^H NMR (DMSO-*d*_6_, 500 MHz): δ major isomer 9.44 (s, OH, 1H), 7.98 (s, Ar H, 1H), 7.89–7.84 (m, Ar H, 1H), 7.78 (s, CH, 1H), 7.32–7.26 (m, Ar H, 3H), 7.05 (d, *J* = 9.0 Hz, Ar H, 2H), 6.87 (d, *J* = 9.0 Hz, Ar H, 2H), 3.83–3.81 (m, OMe, 3H), 3.72 (s, CH_2_, itaconimide, 2H). ^13^C­{^1^H} NMR (DMSO-*d*_6_, 125 MHz): δ 173.3, 173.2, 169.9, 159.3, 140.1, 132.0, 130.7, 128.2, 127.2, 126.2, 125.7, 123.8, 121.4, 120.7, 113.8, 55.3, 37.7, 33.4. IR (KBr): *v*/cm^–1^ 3360, 3079, 2955, 2835, 1775 (C=O of itaconimide), 1701 (C=O coumarin), 1686, 1640, 1512, 1250, 1177, 1029, 880, 829, 671.

##### 2-(6-Chloro-2-oxo-2H-chromen-3-yl)-*N*-(*p*-tolyl)­acetamide **(4ad)** Compound with (E)-3-(5-Chloro-2-hydroxybenzylidene)-1-(p-tolyl)­pyrrolidine-2,5-dione **(4ad′)** (1.8:1)

4 h (73.0 mg, 57% Yield), white solid, mp: 227–228 °C. ^1^H NMR (DMSO-*d*_6_, 500 MHz): δ major isomer 10.0 (s, OH, 1H), 7.98 (s, Ar H, 1H), 7.79 (s, CH, 1H), 7.31 (d, *J* = 8.0 Hz, Ar H, 2H), 7.23 (d, *J* = 8.0 Hz, Ar H, 2H), 7.10 (d, *J* = 8.0 Hz, Ar H, 2H), 3.84 (m, CH_2_, itaconimide, 2H), 2.25 (s, CH_3_, 3H). ^13^C­{^1^H} NMR (DMSO-*d*_6_, 125 MHz): δ 173.2, 173.1, 169.5, 159.3, 140.4, 132.1, 130.7, 128.3, 127.1, 126.2, 125.6, 123.8, 121.4, 120.6, 117.5, 37.8, 33.5, 20.6. IR (KBr): *v*/cm^–1^ 3352, 1767 (C=O itaconimide); 1724 (C=O coumarin), 1701, 1516, 1458, 1400, 1180, 822.

##### 2-(6-Allyl-8-methoxy-2-oxo-2H-chromen-3-yl)-*N*-(4-chlorophenyl)­acetamide **(4ae)** Compound with (E)-3-(5-Allyl-2-hydroxy-3-methoxybenzylidene)-1-(4-chlorophenyl)­pyrrolidine-2,5-dione **(4ae′)** (6:1)

2 h (13.3 mg, 7% Yield), pale-yellow solid, mp: 244–245 °C. ^1^H NMR (DMSO-*d*_6_, 500 MHz): δ major isomer 9.17 (s, OH, 1H), 7.92 (s, CH, 1H), 7.57 (d, *J* = 9.0 Hz, Ar H, 2H), 7.41 (d, *J* = 9.0 Hz, Ar H, 2H), 7.35 (d, *J* = 9.0 Hz, Ar H, 2H), 6.95 (s, Ar H, 1H), 6.89 (s, Ar H, 1H), 6.05–5.97 (m, CH, 2H), 5.15–5.11 and 5.08–5.06 (d, *J* = 18.0 Hz and *J* = 10.0 Hz, CH_2_, 4H), 3.83 (s, OMe, 3H), 3.75 (d, *J* = 2.0 Hz, CH_2_, itaconimide, 2H), 3.36 (d, *J* = 7.0 Hz, CH_2_, 2H). ^13^C­{^1^H} NMR (DMSO-*d*_6_, 125 MHz): δ 173.1, 169.7, 147.7, 146.2, 144.7, 137.8, 130.6, 127.9, 123.3, 121.0, 120.6, 119.9, 119.4, 118.3, 116.1, 115.5, 114.2, 113.8, 55.9, 38.,9, 34.0. IR (KBr): *v*/cm^–1^ 3283, 2997, 2974, 1767 (C=O itaconimide), 1697, 1639, 1589, 1493, 1385, 1254, 1172, 1088, 914, 837, 733.

##### 2-(6-Allyl-8-methoxy-2-oxo-2H-chromen-3-yl)-*N*-phenylacetamide **(4af)** Compound with (E)-3-(5-Allyl-2-hydroxy-3-methoxybenzylidene)-1-phenylpyrrolidine-2,5-dione **(4af′)** (6:1)

2 h (51.3 mg, 29% Yield), pale-yellow solid, mp: 220 °C (decompose). ^1^H NMR (DMSO-*d*_6_, 500 MHz): δ major isomer 9.16 (s, OH, 1H), 7.91 (s, CH, 1H), 7.60–7.28 (m, Ar H, 5H), 6.96 (s, Ar H, 1H), 6.89 (s, Ar H, 1H), 5.15–5.11 and 5.09–5.07 (d, *J* = 18.0 Hz and *J* = 10.0 Hz, CH_2_, 4H), 3.83 (s, OMe, 3H), 3.76 (d, *J* = 2.0 Hz, CH_2_, itaconimide, 2H), 3.36 (d, *J* = 7.0 Hz, CH_2_, 2H). ^13^C­{^1^H} NMR (DMSO-*d*_6_, 125 MHz): δ 173.3, 169.9, 147.8, 144.7, 137.8, 130.6, 127.0, 123.6, 123.5, 123.1, 122.2, 121.0. 119.9, 119.4, 119.1, 118.3. 116.1, 115.5, 114.1, 113.7, 110.6, 56.0, 55.9, 38.9, 34.0. IR (KBr): *v*/cm^–1^ 3075, 2997, 1767 (C=O itaconimide), 1697, 1640, 1589, 1489, 1389, 1254, 1192, 1177, 1126, 910.

##### 2-(6-Allyl-8-methoxy-2-oxo-2H-chromen-3-yl)-*N*-(*p*-tolyl)­acetamide **(4ag)** Compound with (E)-3-(5-Allyl-2-hydroxy-3-methoxybenzylidene)-1-(*p*-tolyl)­pyrrolidine-2,5-dione **(4ag′)** (4:1)

2 h (95.4 mg, 52% Yield), yellow-pearl solid, mp: 240–246 °C. ^1^H NMR (DMSO-*d*_6_, 500 MHz): δ major isomer 9.14 (s, OH, 1H), 7.90 (sl, CH, 1H), 7.30 (d *J* = 8.0 Hz, Ar H, 2H), 7.23 (d, *J* = 9.0 Hz, Ar H, 2H), 6.95 (s, Ar H, 1H), 6.89 (s, Ar H, 1H), 6.05–5.97 (m, CH, 2H), 5.15–5.11 and 5.08–5.06 (d, *J* = 17.0 Hz and *J* = 10.0 Hz, CH_2_, 4H), 3.91 (s, OMe, 3H), 3.83 (s, OMe, 3H), 3.74 (d, *J* = 2.0 Hz, CH_2_, itaconimide, 2H), 3.35 (d, *J* = 7.0 Hz, CH_2_, 2H), 2.37 (s, CH_3_, 3H). ^13^C­{^1^H} NMR (DMSO-*d*_6_, 125 MHz): δ 173.4, 170.0, 147.7, 137.8, 137.5, 130.6, 127.6, 123.6, 123.5, 121.1, 119.9, 119.4, 119.1, 118.3, 116.1, 115.5, 114.1, 113.7, 55.9, 38.9, 33.9, 20.6. IR (KBr): *v*/cm^–1^ 3279, 3078, 2997, 1767 (C=O itaconimide), 1697, 1639, 1516, 1389, 1296, 1254, 1196, 1172, 1126, 907.

##### 2-(6-Allyl-8-methoxy-2-oxo-2H-chromen-3-yl)-*N*-(4-methoxyphenyl)­acetamide **(4ah)** Compound with (E)-3-(5-Allyl-2-hydroxy-3-methoxybenzylidene)-1-(4-methoxyphenyl)­pyrrolidine-2,5-dione **(4ah′)** (4:1)

2 h (121.2 mg, 63% Yield), pearl-yellow solid, mp: 203–204.5 °C. ^1^H NMR (DMSO-*d*_6_, 500 MHz): δ major isomer 9.14 (s, OH, 1H), 7.90 (sl, CH, 1H), 7.26 (d *J* = 9.0 Hz, Ar H, 2H), 7.04 (d, *J* = 9.0 Hz, Ar H, 2H), 6.95 (s, Ar H, 1H), 6.88 (s, Ar H, 1H), 6.05–5.97 (m, CH, 2H), 5.15–5.11 and 5.06–5.06 (d, *J* = 17.0 Hz and *J* = 10.0 Hz, CH_2_, 4H), 3.83 (s, OMe, 3H), 3.81 (s, OMe, 3H), 3.73 (d, *J* = 2.0 Hz, CH_2_, itaconimide, 2H), 3.35 (d, *J* = 7.0 Hz, CH_2_, 2H). ^13^C­{^1^H} NMR (DMSO-*d*_6_, 125 MHz): δ 173.5, 170.1, 147.8, 144.7, 141.8, 130.6, 128.1, 127.5, 125.2, 123.5, 121.1, 120.7, 119.9, 118.3, 116.1, 115.5, 113.8, 113.7, 55.9, 55.3, 38.9, 33.9. IR (KBr): *v*/cm^–1^ 3267, 3071, 2997, 2955, 2831, 1762 (C=O itaconimide), 1728, 1716, 1694, 1639, 1512, 1385, 1300, 1250, 1172, 1126, 837.

##### 2-(2,8-Dioxo-2H,8H-pyrano­[2,3-*f*]­chromen-9-yl)-*N*-(*p*-tolyl)­acetamide **(4ai)** Compound with (E)-3-((7-Hydroxy-2-oxo-2H-chromen-8-yl)­methylene)-1-(*p*-tolyl)­pyrrolidine-2,5-dione **(4ai′)** (2:1)

2 h (134.7 mg, 51% Yield), orange solid, mp: 228–230 °C. ^1^H NMR (DMSO-*d*_6_, 500 MHz): δ 10.00 (s, NH, 1H), 8.33 (s, Ar H, 1H), 8.12 (d, *J* = 10.0 Hz, Ar H, 2H), 7.98 (d, *J* = 10.0 Hz, Ar H, 1H), 7.91 (d, *J* = 9.0 Hz, Ar H, 2H), 7.70 (sl, CH, 1H), 7.61 (d, *J* = 9.0 Hz, Ar H, 1H), 7.47 (d, *J* = 9.0 Hz, Ar H, 2H), 7.38 (d, *J* = 9.0 Hz, Ar H, 1H), 7.31 (d, *J* = 8.0 Hz, Ar H, 1H), 7.26 (d, *J* = 9.0 Hz, Ar H, 1H), 7.10 (d, *J* = 8.0 Hz, Ar H, 2H), 6.95 (d, *J* = 9.0 Hz, Ar H, 1H), 6.52 (d, *J* = 10.0 Hz, Ar H, 2H), 6.27 (d, *J* = 10.0 Hz, Ar H, 1H), 3.73 (s, CH_2_, itaconimide 2H), 3.62 (d, *J* = 2.0 Hz, CH_2_, coumarin 2H), 2.37 (s, CH_3_, 3H), 2.25 (s, CH_3_, 3H). ^13^C­{^1^H} NMR (DMSO-*d*_6_, 125 MHz): δ 173.2, 169.4, 167.0, 159.8, 159.8, 159.0, 154.6, 153.0, 149.6, 144.7, 144.1, 137.6, 136.4, 134.2, 132.0, 130.5, 130.4, 130.0, 129.1, 128.9, 128.4, 126.8, 124.3, 123.9, 119.1, 114.9, 114.6, 112.9, 112.5, 111.2, 111.1, 109.1, 108.0, 37.7, 35.0, 27.3, 26.8, 23.3, 23.3, 20.6, 20.2, 13.3. IR (KBr): *v*/cm^–1^ 2924, 1721, 1674, 1600, 1516, 1404, 1169, 1114, 1015, 841, 818 cm^–1^.

##### *N*-(4-Methoxyphenyl)-2-(2-oxo-2H-chromen-3-yl)­acetamide **(4aa)** [Obtained by Continuous Flow]

0.5 mmol of **1**, 0.5 mmol of **2**, and 1.6 equiv of 0.8 mmol of tributylphosphine were mixed. Heat the maleimide solution to 75 °C. Two hours (76.6 mg, 49% yield), pale-pink solid, mp: 187–188 °C. ^1^H NMR (DMSO-*d*_6_, 500 MHz): δ 10.02 (s, NH, 1H), 7.99 (s, CH, 1H), 7.71 (d, *J* = 10.0 Hz, Ar H, 1H), 7.61–7.58 (t, *J* = 10.0 Hz, Ar H, 1H), 7.49 (d, *J* = 11.0 Hz, Ar H, 2H), 7.41 (d, *J* = 11.0 Hz, Ar H, 1H), 7.38–7.34 (t, *J* = 10.0 Hz, Ar H, 1H), 6.87 (d, *J* = 11.0 Hz, Ar H, 2H), 3.71 (s, OMe, 3H), 3.59 (s, CH_2_, 2H). ^13^C­{^1^H} NMR (DMSO-*d*_6_, 125 MHz): δ 167.2, 160.9, 155.3, 152.9, 142.0, 132.3, 131.4, 128.1, 124.7, 123.7, 120.7, 119.2, 116.1, 113.9, 55.2, 37.9.

### Determination of Photophysical Properties

UV–visible absorption spectra were recorded using a Varian Cary 50 spectrophotometer equipped with a quartz cuvette (10 mm optical path length). Fluorescence measurements were performed on a Varian Cary Eclipse spectrofluorimeter. All measurements were carried out in chloroform at room temperature using solutions with a concentration of 10 μM. The samples were excited at their respective absorption maxima (405–438 nm). Fluorescence quantum yields (Φ*F*) were determined using quinine sulfate as a reference standard (Φ*F* = 0.546 in 0.5 mol L^–1^ H_2_SO_4_; absorbance = 0.045; integrated emission area = 22,289).

## Computational Methods

The calculations were carried out employing the Gaussian 09 package (revision D.01). Density Functional Theory (DFT) was used in the optimization of the structures, transition states (TSs), and molecular complexes (MCs). The chosen theory level involved the use of the M06-2X functional, the 6-31G­(d) basis set (grid = ultrafine), and the solvation model based on density (SMD) for ethanol and was based on previous studies.^31–33^ The Berny algorithm was used in the optimization of the TSs, which presented a single imaginary frequency. In addition, intrinsic reaction coordinate (IRC) calculations were performed to verify the connectivity between the corresponding minima along the reaction pathway. The thermodynamic properties were determined using a temperature of 423.15 K (150 °C) and a pressure of 17 atm (250 psi), in order to precisely reproduce the experimental conditions. The vibrational analysis was carried out to confirm the identity of all stationary points and in the determination of the thermal corrections to enthalpy and Gibbs free energy.

## Supplementary Material



## Data Availability

The data underlying this study are available in the published article and its Supporting Information.

## Notes

Experimental detail for reaction optimization, control reactions, synthetic details involving the preparation of **4aa–4ai/4aa′–4ai′**, pictures of selected experimental procedures, photophysical data, copies of NMR, HRMS, IV spectra, and DFT computational details (PDF)
