# Supplementary material for: Domino Synthesis of Coumarins via Phosphine-Mediated Formal Oxa-[4 + 2] Cycloaddition: Discovering a Missing Recyclization of Maleimides with Salicylaldehydes
Source: J Org Chem. 2026 Jul 3;91(28):9739–51. doi: 10.1021/acs.joc.6c00595 (PMC13386536; doi:10.1021/acs.joc.6c00595)
Supplement: Supplementary file 1 [file jo6c00595_si_001.pdf]

Supporting Information

Domino Synthesis of Coumarins via Phosphine-Mediated Formal *Oxa*-[4+2] Cycloaddition: Discovering a Missing Recyclization of Maleimides with Salicylaldehydes

Fernando Alves Barretto,[1] Pedro P. de Castro,[2,3] Kleber T. de Oliveira,[3] and Silvio Cunha[1*]

[1] Instituto de Química, Universidade Federal da Bahia, Campus de Ondina, Bahia, 40170-115, Brazil

[2] Departamento de Farmácia. Universidade Federal de Juiz de Fora - Campus Governador Valadares, Minas Gerais 35010-177, Brazil

[3] Departamento de Química, Universidade Federal de São Carlos, São Carlos 13565-905, Brazil

silviodc@ufba.br

Table of Contents

1. **Additional batch optimization data** S2
2. **Additional data concerning the reaction scope** S6
3. **Optimization of coumarin synthesis in continuous flow** S7
4. **Additional fluorescence data** S9
5. **Spectral data of compounds 4a-4ai and 4aa'-4ai'** .... S**Erro! Indicador não definido.**

6 Computational data .... S**Erro! Indicador não definido.**

6.1. Energy profile (ΔG) of the evaluated pathway .... S**Erro! Indicador não definido.**

6.2. Electronic energies (E), enthalpies (H) and Gibbs free energies (G) of all optimized structures .... S**Erro! Indicador não definido.**

6.3. Electronic energies (ΔE), enthalpies (ΔH) and Gibbs free energies (ΔG) variation along the reaction pathway .... S**Erro! Indicador não definido.**

6.4. Intrinsic reaction coordinates (IRC) .... S**Erro! Indicador não definido.**

6.5. Images of all optimized structures .... S**Erro! Indicador não definido.**

6.6. Coordinates of optimized structures .... S**Erro! Indicador não definido.**

1. Additional batch optimization data

Phosphine catalyzed the formation of itaconimide **3a-f** and coumarin **4a-c**: Preliminary studies employing triphenylphosphine as catalyst revealed that the reaction between maleimides and salicylaldehyde derivatives in methanol at room temperature leads to the formation of itaconimides together with mixtures of coumarin isomers. However, under these conditions the reaction displayed a limited substrate scope.

Table S1. Optimization of the Reaction Conditions between **1a** and **2a**.

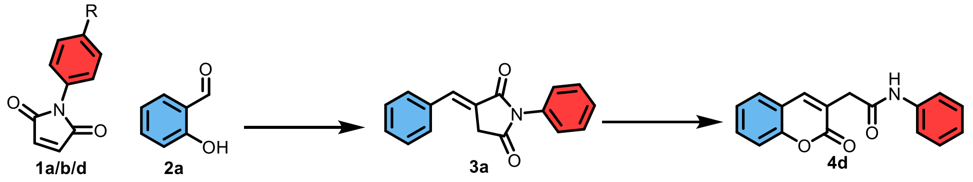

Entry	Reagents	Phophine ratio	MW power (W)	Solvent	T (°C)	Time (h)	Product Yield(%)
1	1d/2a	$P(Bu)_3$ 1 equiv.	-	MeOH	r.t	30 min.	(3d)/(4a) E/C 6:1[a], 44
2	1a/2a	$P(Bu)_3$ 1 equiv.	-	MeOH	r.t	1	(3a) 48
3	1a/2a	$P(Bu)_3$ 1 equiv.	-	EtOH	r.t	1.5	(3a) 85
4	1a/2a	PPh_3 1 equiv.	-	MeOH	r.t	1	(3a) 71
5	1b/2a	PPh_3 1 equiv.	-	MeOH	r.t	0.5	(3d)/(4a) E/C 4:1[a],72
6	1a/2a	PPh_3 1.2 equiv.	250W	EtOH	150	2	(3a) 73
7	1a/2a	$P(Bu)_3$ 1 equiv.	250 W	EtOH	150	2	(3a) 58
8	1a/2a	PPh_3 1.2 equiv.	250W	EtOH	150	2	(3a) 73
9	3a	$P(Bu)_3$ 0.4 equiv.	250W	EtOH	150	2	(4d) 65
10	3a	$P(Bu)_3$ 0.2 equiv.	250W	EtOH	150	2	(4d) 75
11	3a	$P(Bu)_3$ 0.1 equiv.	250W	EtOH	150	2	(4d) 70
12	3a	none	250W	EtOH	150	2	(3a) recovery
13	3a	$P(Bu)_3$ 0.2 equiv.	---	EtOH	r.t	2	(3a) recovery
14	3a	Ph3P=O 0.2 equiv.	250W	EtOH	150	2	(3a) recovery
**15**	**1a/2a**	**$P(Bu)_3$ 1.2 equiv.**	**250W**	**EtOH**	**150**	**2**	**(4d) 44[b]**

[a]E: E-isomer of 3, C: Coumarin; [b]cascate reaction.

General Procedure for the Synthesis of itaconimides and mixtures of isomers: 0.5 mmol of maleimide **2a-c** (1.0 equivalent), 0.5-1.0 mmol of the salicylaldehyde derivative **1a-c** (1.0-2.0 equivalents)and 0.5 mmol of triphenylphosphine (1.0 equivalent) were added to a 10 mL flask. Next, 5 mL of methanol was added to the flask. The reaction was left to proceed under magnetic stirring at room temperature for 15 minutes to 24 hours. At the end of the reaction, after analysis by TLC, the products **3a-f** and **4a-c** were vacuum filtered and washed with cold methanol.

Scheme S1. Initial study of the synthesis of itaconimides and coumarins using triphenylphosphine

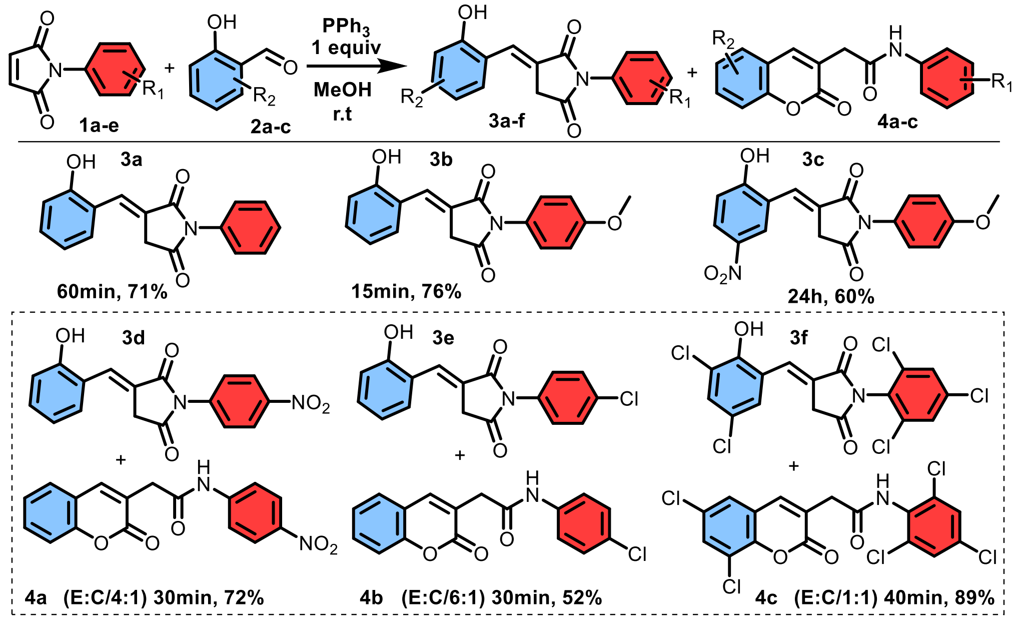

[a]E: E-isomer of **3**, and C: coumarin

The selectivity of different phosphines was investigated through a series of control experiments. These studies revealed that microwave irradiation provides the most effective conditions for the selective formation of coumarin derivatives.

Scheme S2. Study of the selectivity of $P(Bu)_3$ and PPh_3

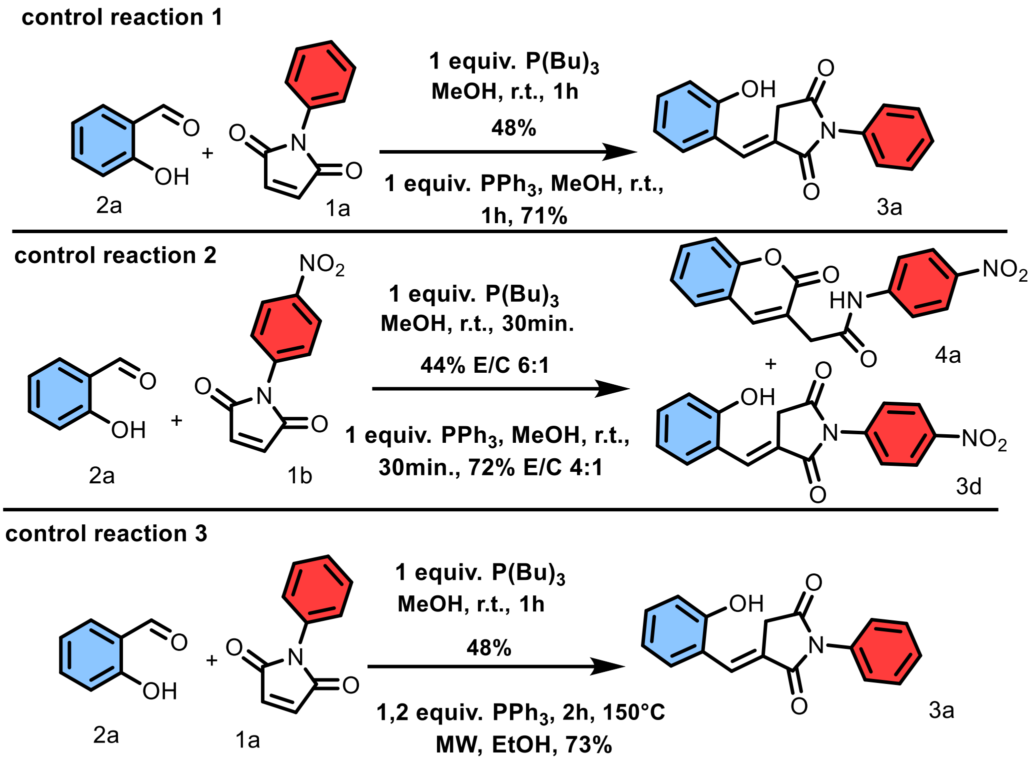

*General Procedure for the Synthesis of itaconimide **3a***: 0.25 mmol of maleimide **2a** (1.0 equivalent), 0.25 mmol of salicylaldehyde (1.0 equivalent) and 0.25 mmol of tributylphosphine (1.0 equivalent) were added to a 10 mL flask. Next, 3 mL of ethanol was added to the flask. The reaction was left to proceed under magnetic stirring at room temperature for 1.5 hours. At the end of the reaction, after analysis by TLC, the product **3a** was vacuum filtered and washed with cold ethanol.

*(E)-3-(2-hydroxybenzylidene)-1-phenylpyrrolidine-2,5-dione (**3a**)*: white solid, 85% yield, m.p. 262.5-263.2 °C, ^{1}H NMR (500 MHz, DMSO-d_6) δ: 10.24(s, OH, 1H), 7.91(t, J=2.5Hz, C=CH, 1H), 7.50-7.56(m, ArH, 3H), 7.42-7.45(m, ArH, 1H), 7.36(d, J=8.5Hz, ArH, 2H), 7.27-7.30(m, ArH, 1H), 6.96(d, J=8.0Hz, ArH, 1H), 6.92(t, J=8.0Hz, ArH, 1H), 3.80(d, J=3.5Hz, CH_2, 2H). ^{13}C{^{1}H} NMR (DMSO-d_6, 125 MHz) δ: 173.6, 170.1, 157.1, 132.7, 131.5, 129.2, 128.8, 128.1, 127.5, 127.1, 123.4, 121.0, 119.5, 115.9, 34.0. IR (KBr): v/cm^{-1} :3449, 3055, 2951, 1771, 1686, 1647, 1601.

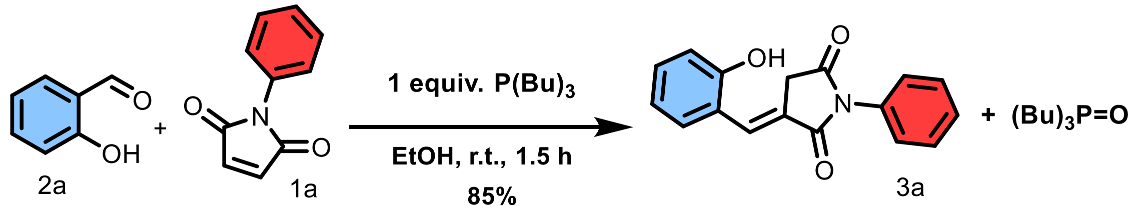

Scheme S3. Optimization of the tributylphosphine catalyzed formation of itaconimide **3a**

Itaconimide **3a** was used in the optimization of tributylphosphine catalyzed formation of coumarin **4d**. The optimization study using tributylphosphine catalysis showed that coumarin isomer **4d** can be obtained under ethanol and MW conditions as only one isomer.

*General Procedure for the Synthesis of coumarin **4d***: 0,5 mmol of itaconimide **3a** (1.0 equivalent), 5 mL of ethanol was added to the 10 mL flask and 0.05-0.2 mmol of triphenylphosphine (10-40 mol%) were added. The reaction was left to proceed under magnetic stirring at MW conditions (150 °C, 2 hours, 250 W, 250 psi and a heating ramp time of 2 minutes). At the end of the reaction, after analysis by TLC, the product **4d** was vacuum filtered and washed with cold ethanol.

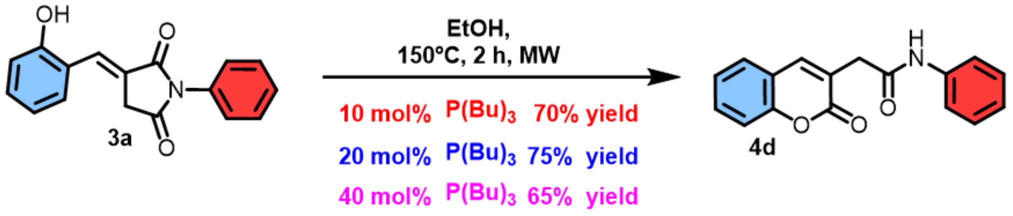

Scheme S4. Influence of tributylphosphine loading on the synthesis of coumarin **4d**

2. Additional data concerning the reaction scope

When maleimides **2a**, **2i**, and **2j** were employed with salicylaldehyde **1a**, complex reaction mixtures were obtained. It was also observed that both electron-donating and electron-withdrawing substituents on the aromatic ring of salicylaldehydes **1a-c** or maleimides **2a-m** led to the formation of coumarins **4aa-ai** and itaconimides **4aa'-ai'** as enriched mixtures. In most cases, the reactions preferentially afforded itaconimide derivatives; however, the mixtures obtained for **4aa** and **4ab** were enriched in the corresponding coumarin products.

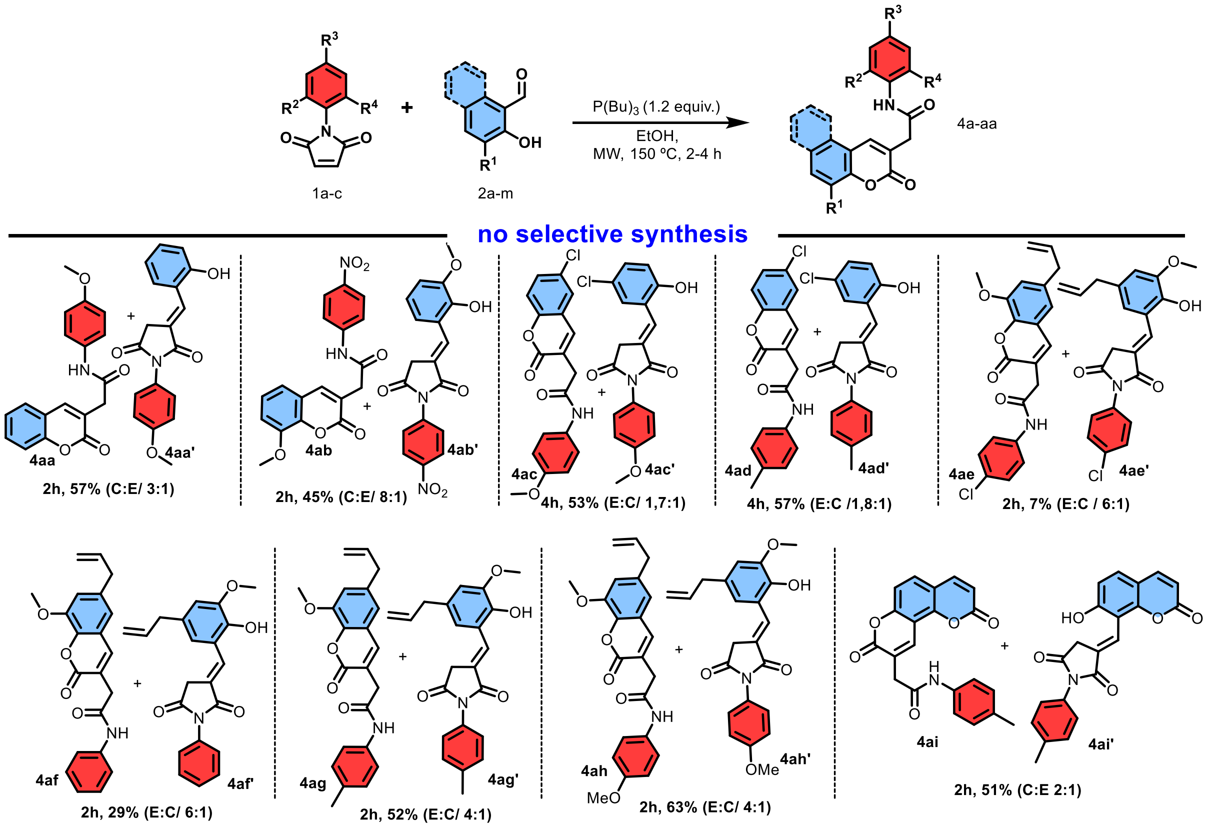

Scheme S5. Scope of coumarin and itaconimide mixtures

3. Optimization of coumarin synthesis in continuous flow

Some attempts to optimize the synthesis of coumarin **4d** under continuous flow conditions were investigated. A single-pump setup proved to be unfeasible due to the risk of precipitation of the intermediate before reaching the heated reactor zone, which could lead to clogging of the system. Therefore, the use of a mixer and two independent pumps in the continuous-flow setup was required to ensure proper mixing only upon entry into the reactor. As shown in **Table S1**, several parameters were evaluated, including reactant concentration, temperature, stainless-steel reactor configuration, and residence time; however, the best conditions were obtained in entry 1.

Table S2. Optimization Studies of Coumarin **4d** Under Continuous Flow.

Entry	Phosphine concentration (mol/L)	Maleimide/aldehyde concentration (mol/L)	Total Flow (µl/min)	Temperature (°C)	Residence time (h)	Yield (%)[*]
**1**	**0.09**	**0.07**	**316**	**160**	**2**	**63[a]**
**2**	0.06	0.06	316	160	2	52
**3**	0.1	0.1	316	160	2	38[b]
**4**	0.09	0.07	632	160	1	---[c]
**5**	0.1	0.1	632	160	1	---[c]
**6**	0.11	0.07	316	160	2	62[d]
**7**	0.09	0.07	316	180	2	23[e]
**8**	0.09	0.07	316	140	2	---[b]
**9****	0.1	0.07	316	160	1	36[b]
**10****	0.07	0.07	133	160	2	39
**11****	0.1	0.1	133	160	2	46

[*] Treatment similar to the use MW conditions; [a] Temperature (160 °C); Total flow (316 µl/min); time (2 h); [b] Coumarins and intermediate mixture; [c] There is a maleimide spot; [d] tributylphosphine 2 equiv.; [e] Begins to decompose into several spots; [**] Syrris Asia Premium Systems stainless steel reactor with HPLC55 thermal jacket (~13 mL).

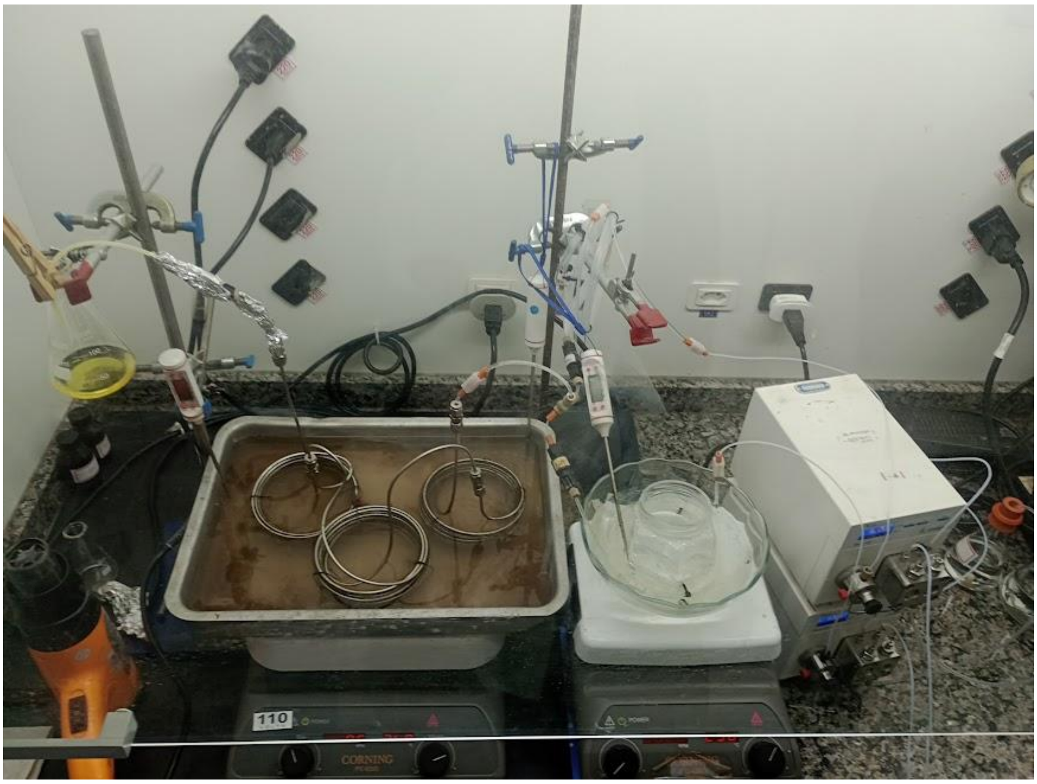

Figure S1. Continuous flow system setup

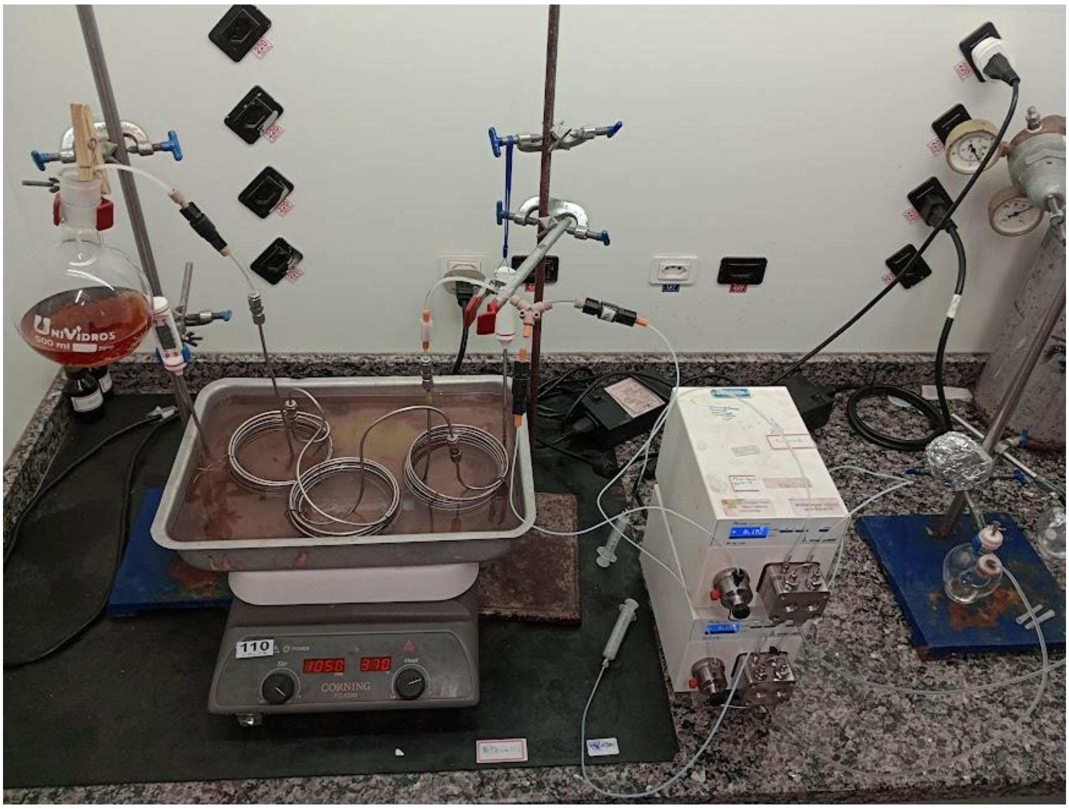

Figure S2. Continuous flow system setup for the 3 mmol scale reaction

4. Additional fluorescence data

Measurement of fluorescence quantum yield $\mathbf{\Phi}_{\boldsymbol{F(x)}}$ Fluorescence quantum yields $\mathbf{\Phi}_{\boldsymbol{F(x)}}$ were evaluated using quinine sulfate (0.546 at 0.5 mol/L H_2SO_4; Absorbance=0,045; area=22289) and the quantum yield was calculated from Equation 1. Emission spectra of 8 solutions were recorded from 405 to 438 nm with excitation at 355 to 299 nm.

$$\mathbf{\Phi}_{\boldsymbol{F(x)}} = \left(\frac{\boldsymbol{A_s}}{\boldsymbol{A_x}}\right)\left(\frac{\boldsymbol{F_x}}{\boldsymbol{F_s}}\right)\left(\frac{\boldsymbol{n_x}}{\boldsymbol{n_s}}\right)^{\mathbf{2}} \mathbf{\Phi}_{\boldsymbol{F(s)}} \qquad \textbf{(Equation 1)}$$

Onde:

$\mathbf{\Phi}_{\boldsymbol{F(x)}}$ = Quantum yield of the sample (x refers to the sample and s to the standard);

$\boldsymbol{A}$ =Absorbance at the excitation wavelength;

$\boldsymbol{F}$ = Area of the emission curve;

$\boldsymbol{n}$ = Refractive index of the solvents used;

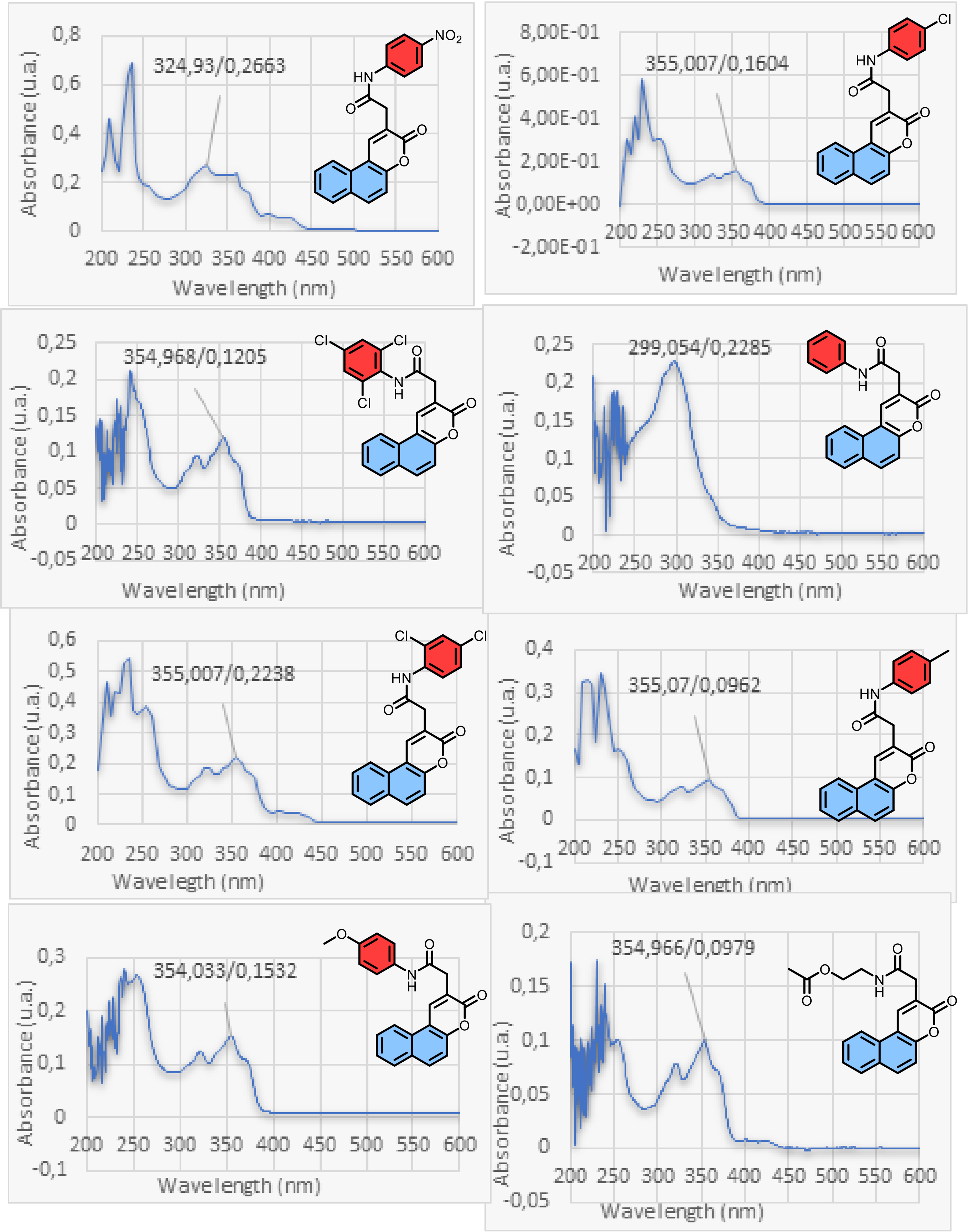

Figure S3. Absorption spectra of coumarins at a concentration of $5x10^{-6}$ M

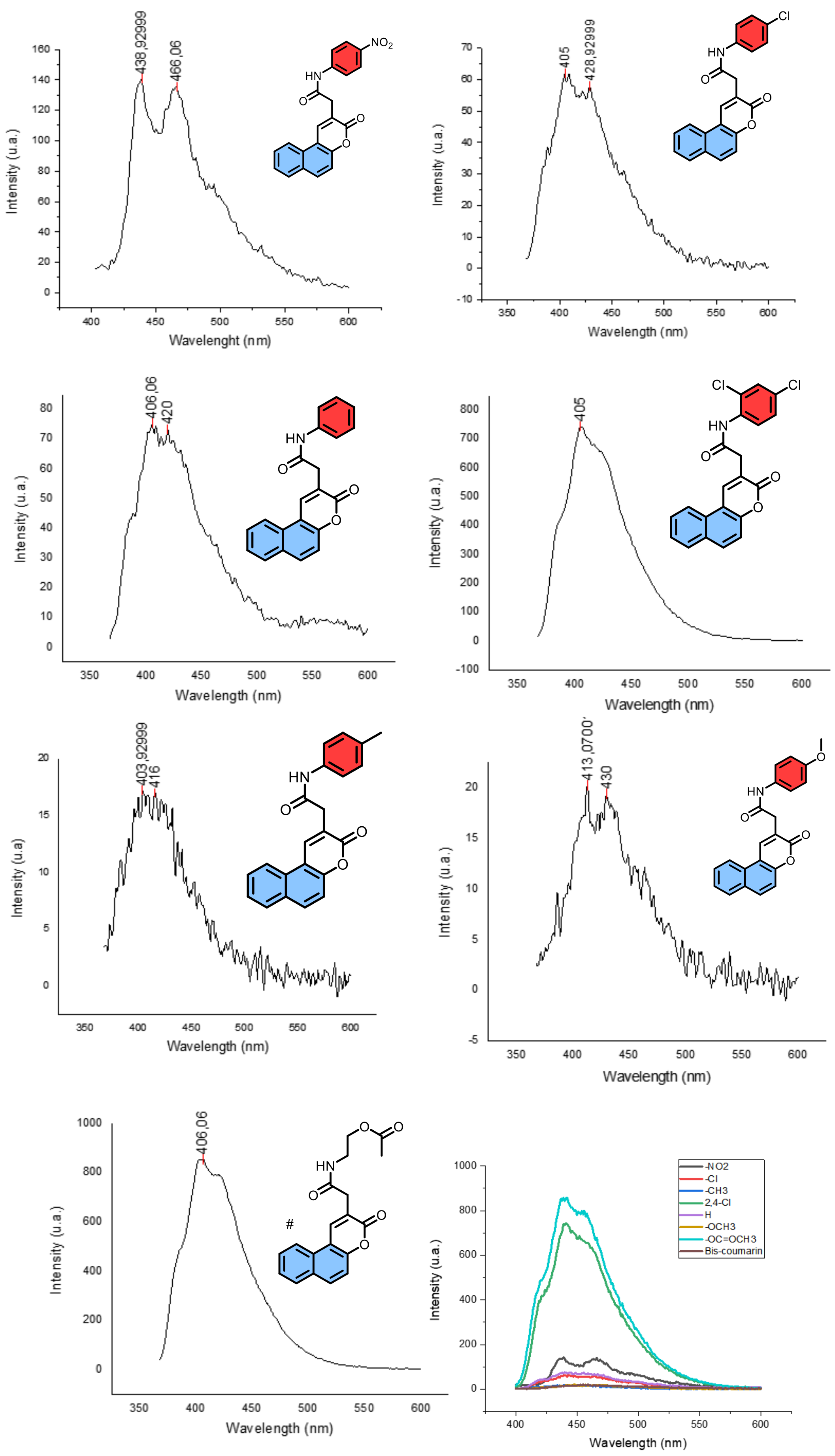

Figure S4. Emission spectra of coumarins at a concentration of *5x10*$^{-6}$ M

4. Spectral data of compounds 4a-4ai and 4aa'-4ai'

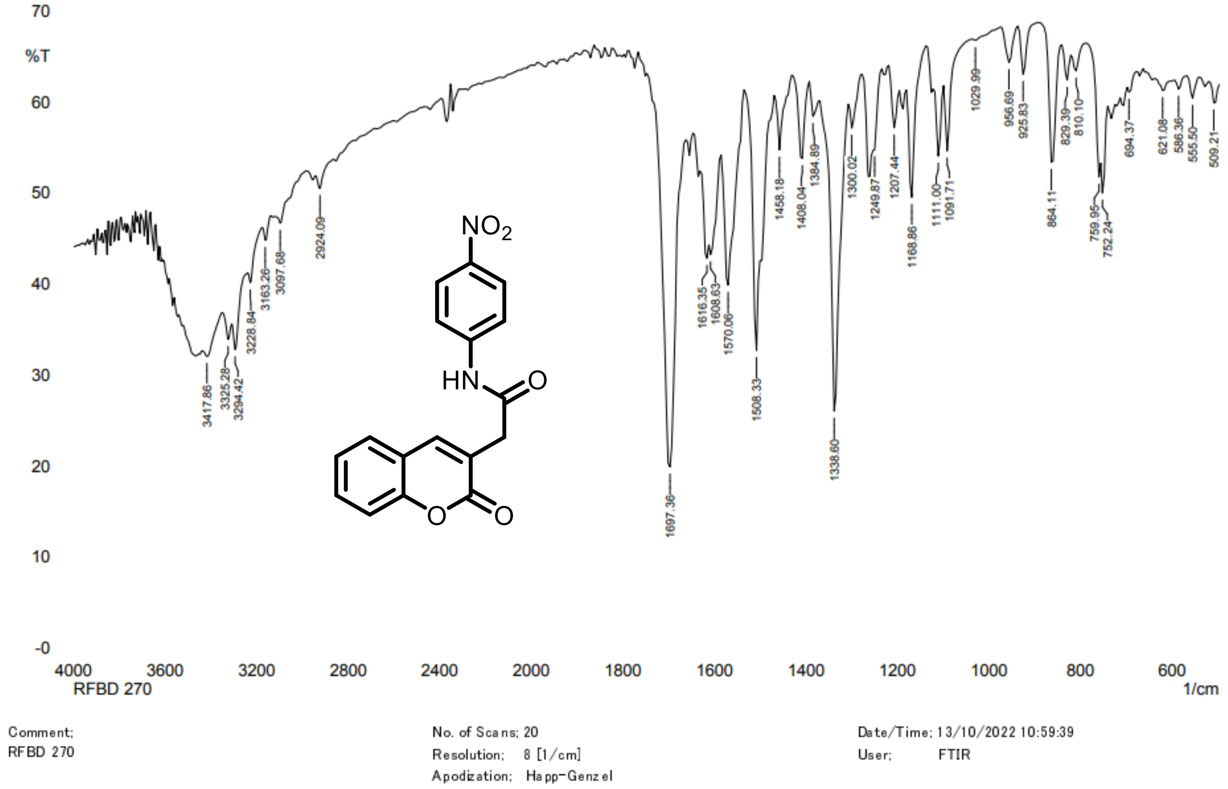

Figure S5. IR (KBr) of **4a**

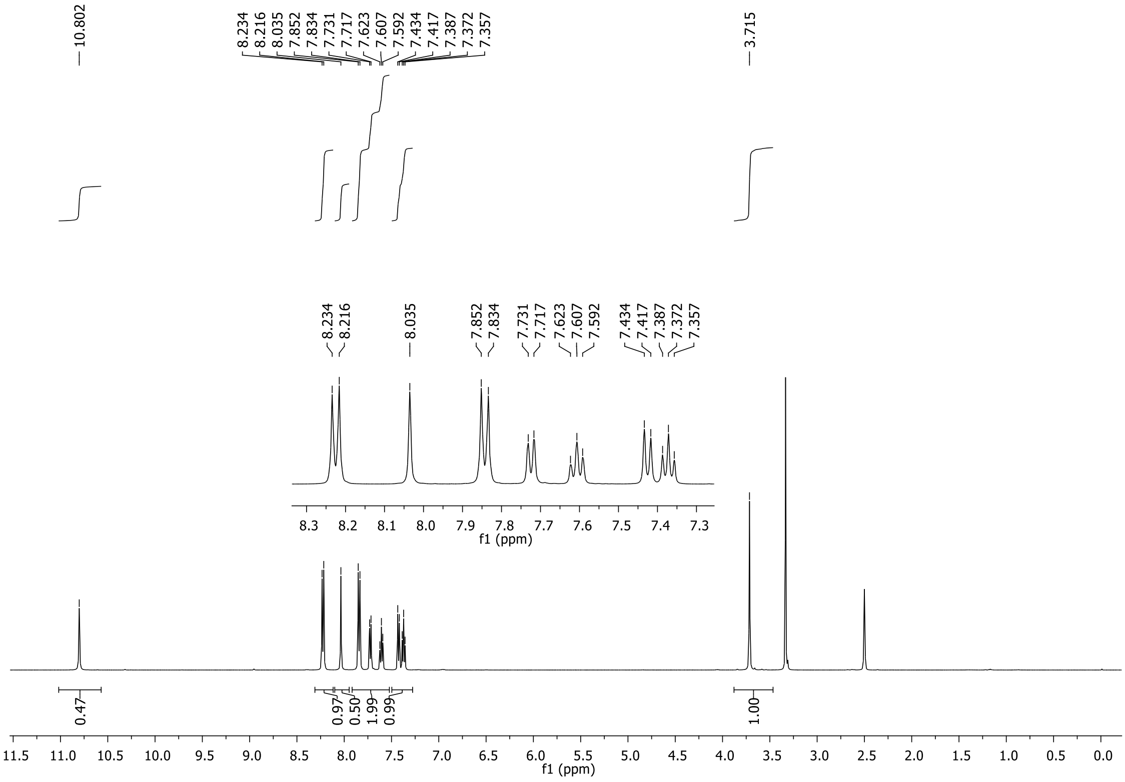

Figure S6. ^{1}H NMR (DMSO-d$_6$, 500 MHz) of **4a**

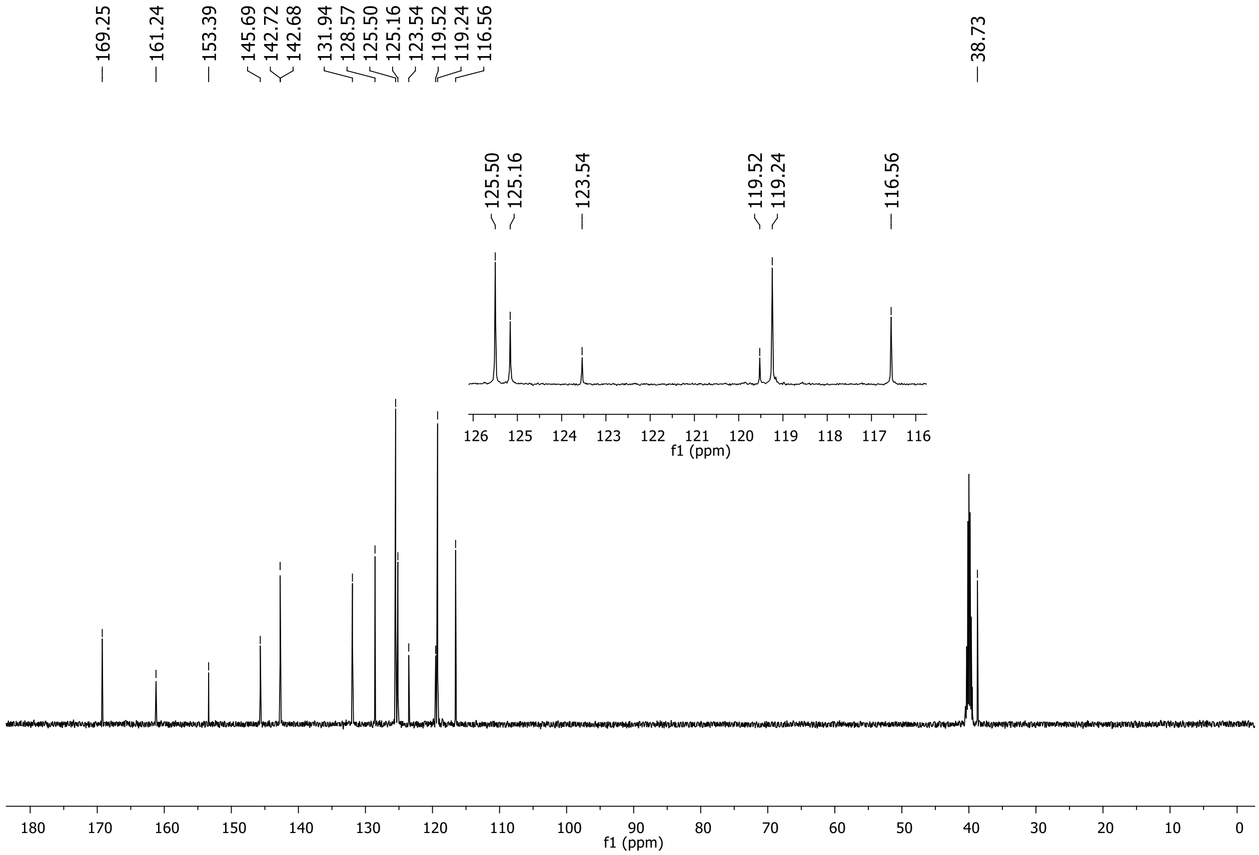

Figure S7. $^{13}C\{^{1}H\}$ NMR (DMSO-d$_6$, 125 MHz) **of 4a**

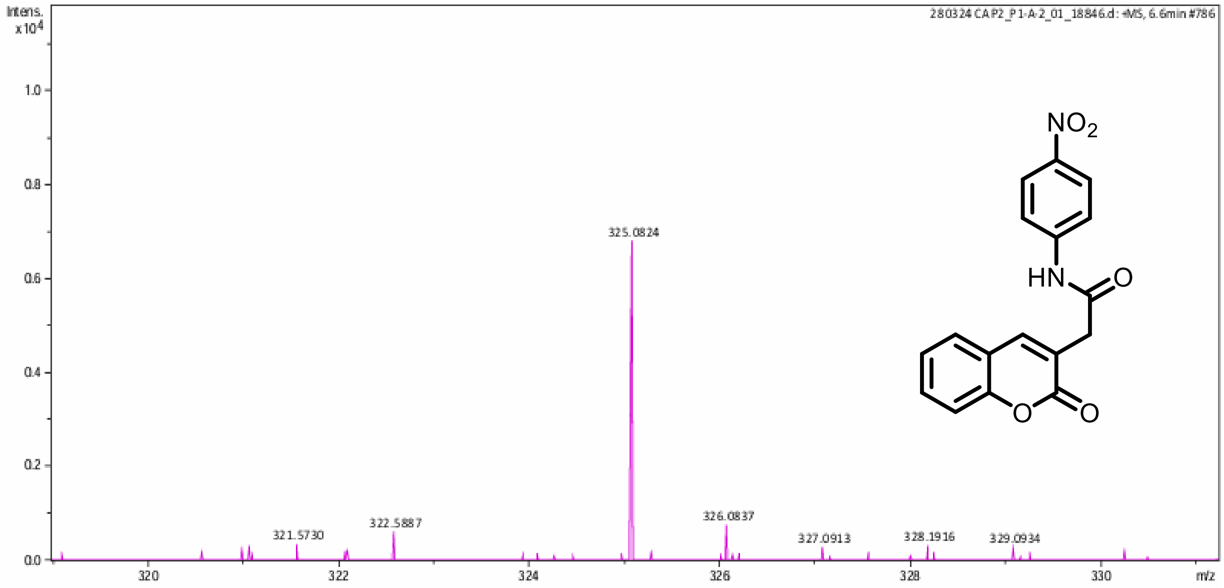

Figure S8. HRMS of **4a**

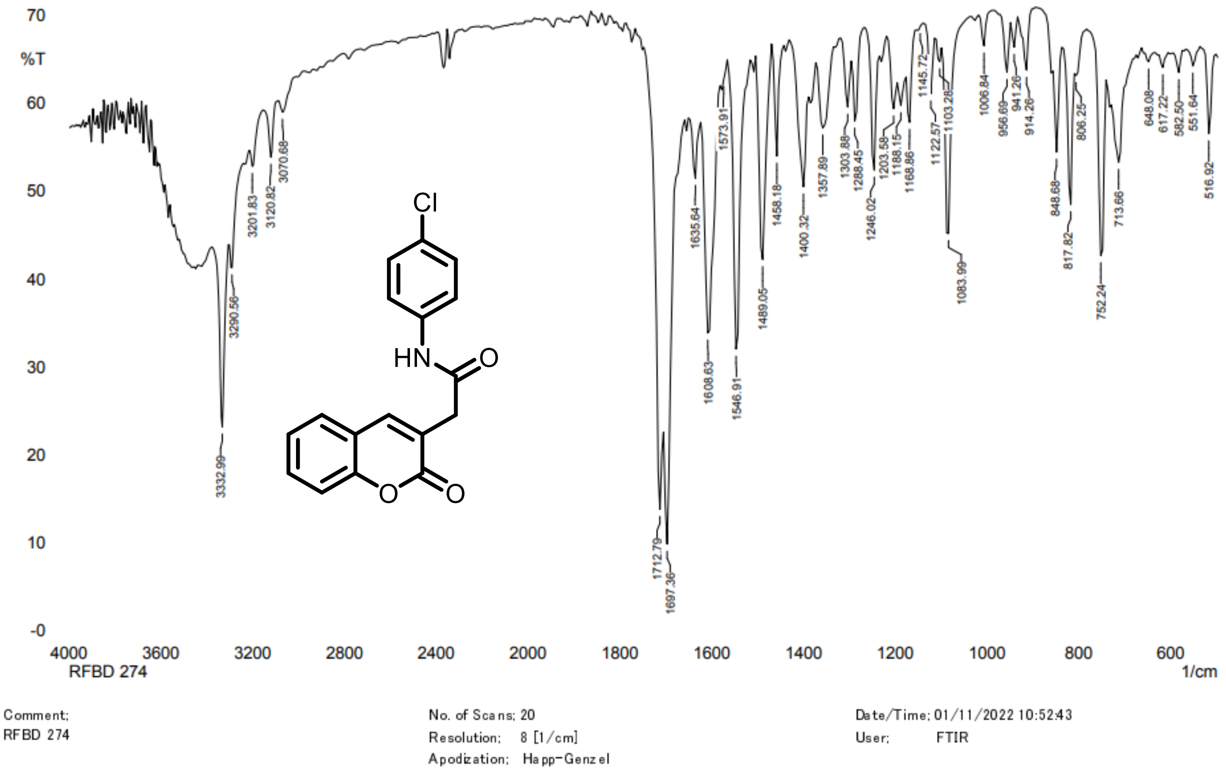

Figure S9. IR (KBr) of **4b**

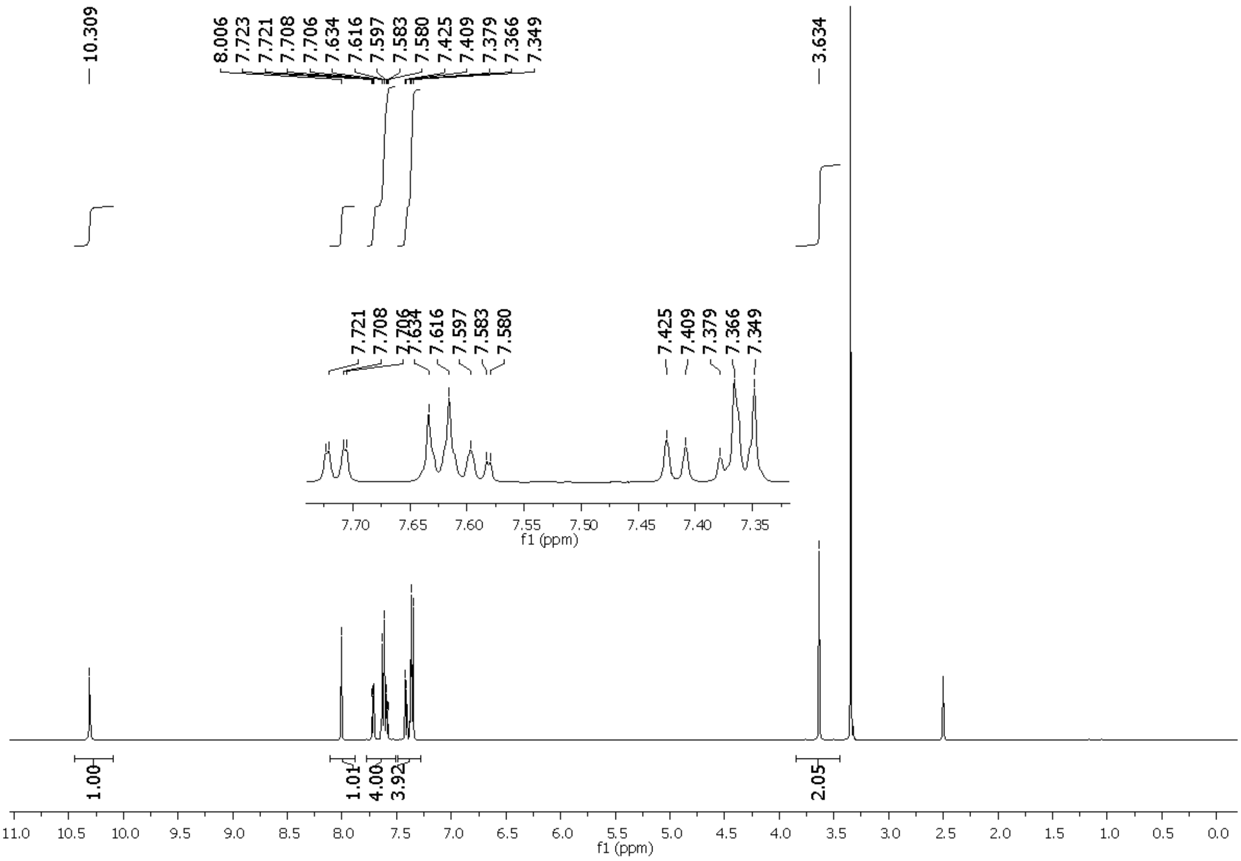

Figure S10. ^{1}H NMR (DMSO-d_6, 500 MHz) of **4b**

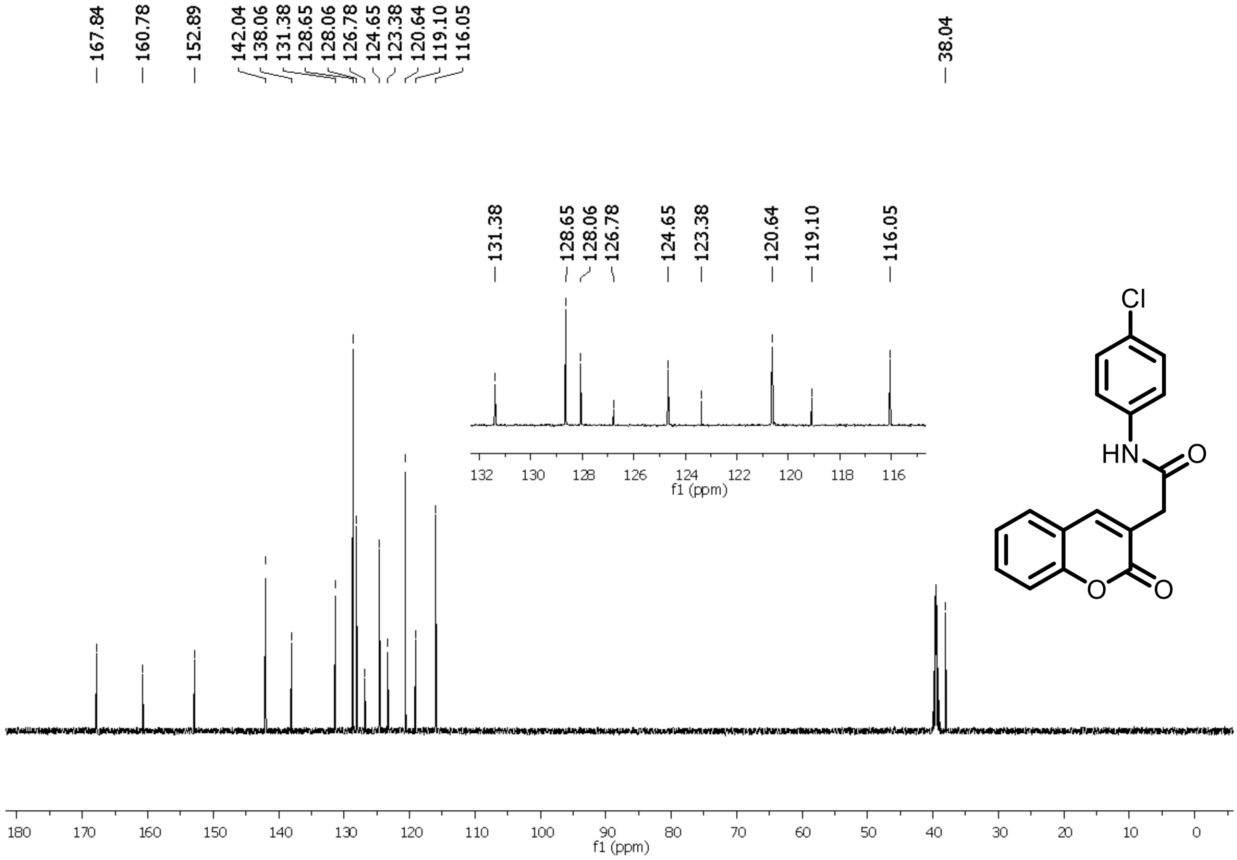

Figure S11. $^{13}C\{^{1}H\}$ NMR (DMSO-d$_6$, 125 MHz) **of 4b**

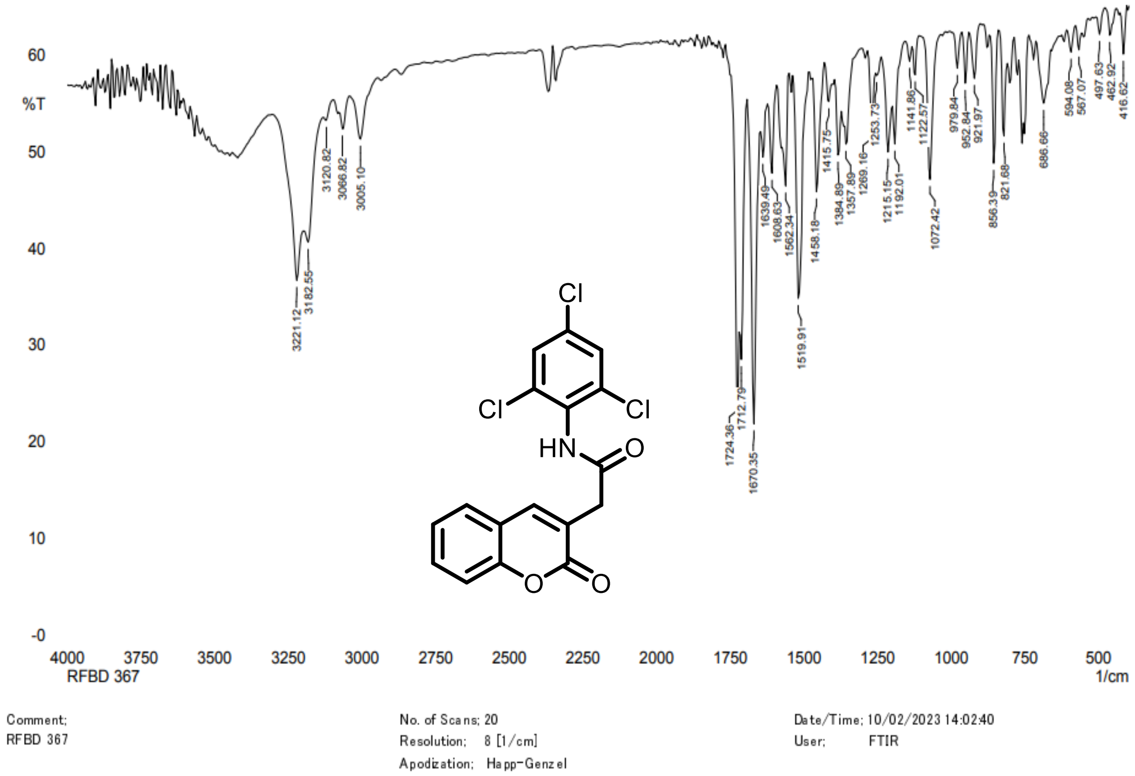

Figure S12. IR (KBr) of **4c**

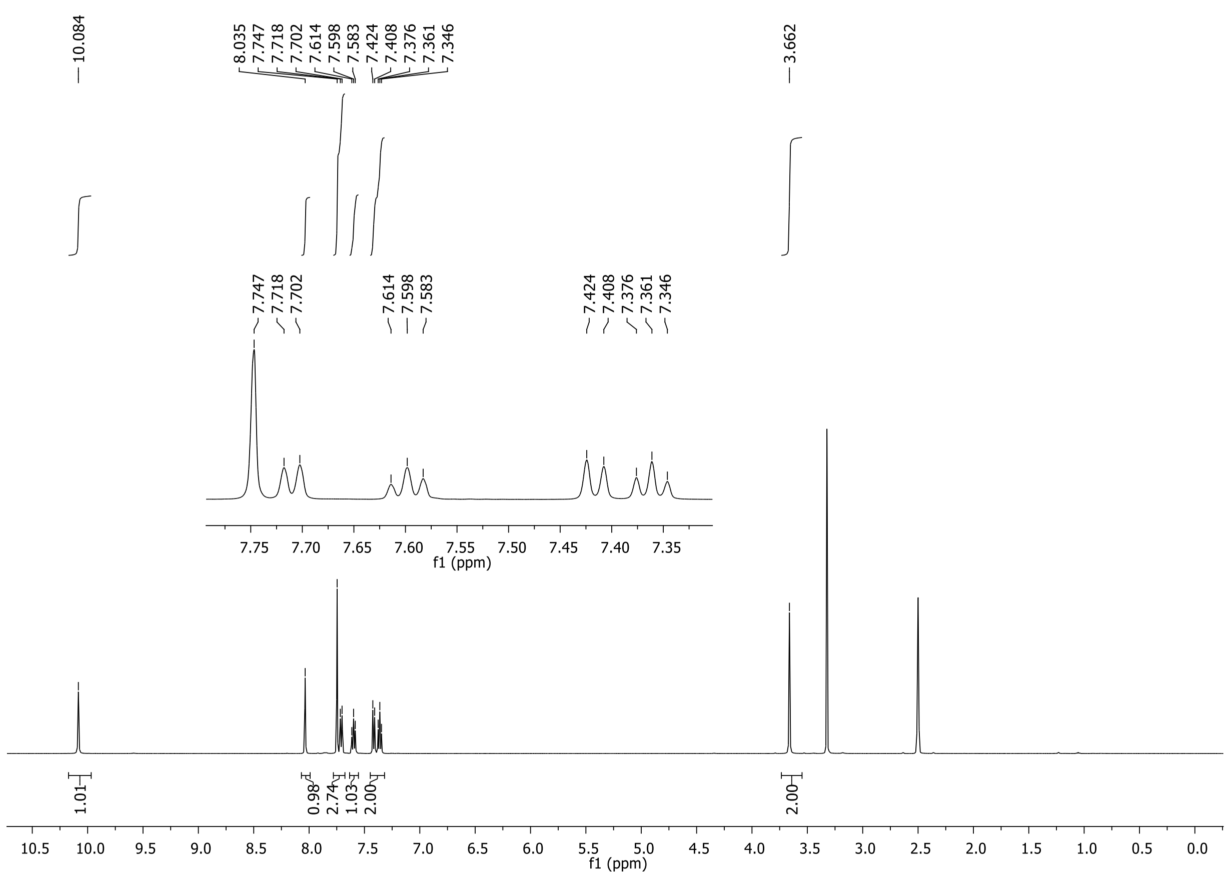

Figure S13. ^{1}H NMR (DMSO-d$_6$, 500 MHz) of **4c**

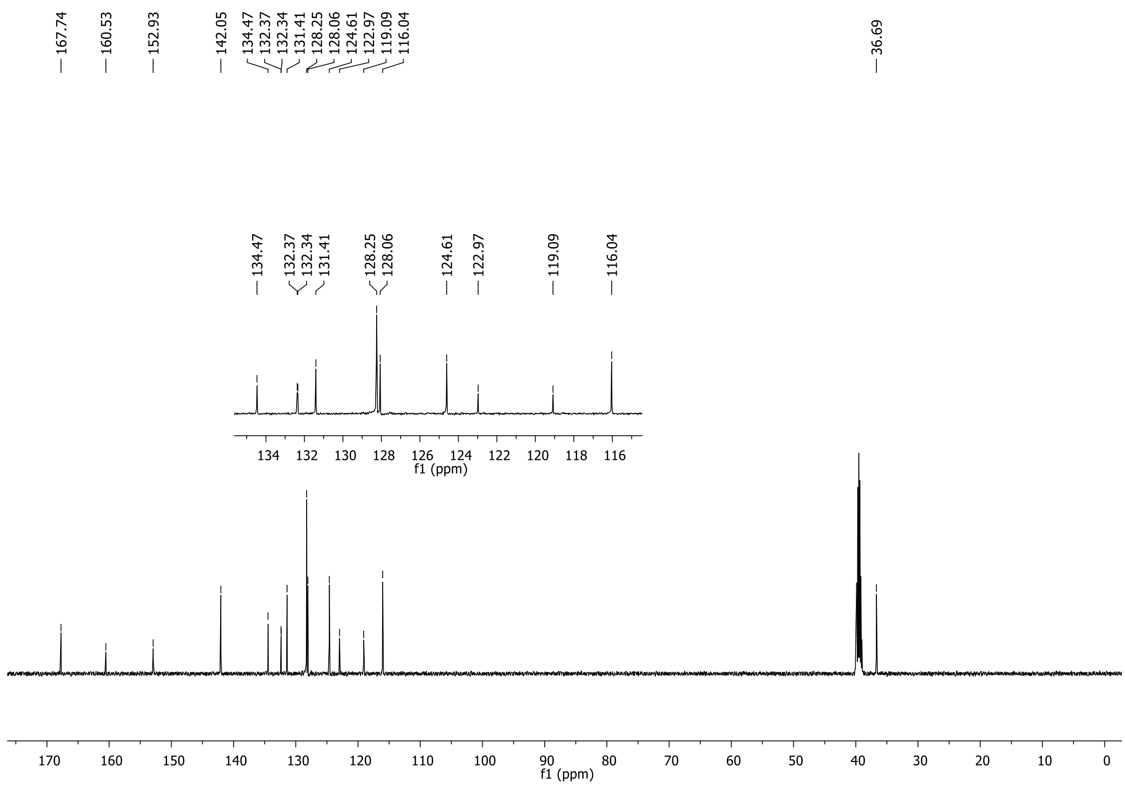

Figure S14. $^{13}C\{^{1}H\}$ NMR (DMSO-d$_6$, 125 MHz) of **4c**

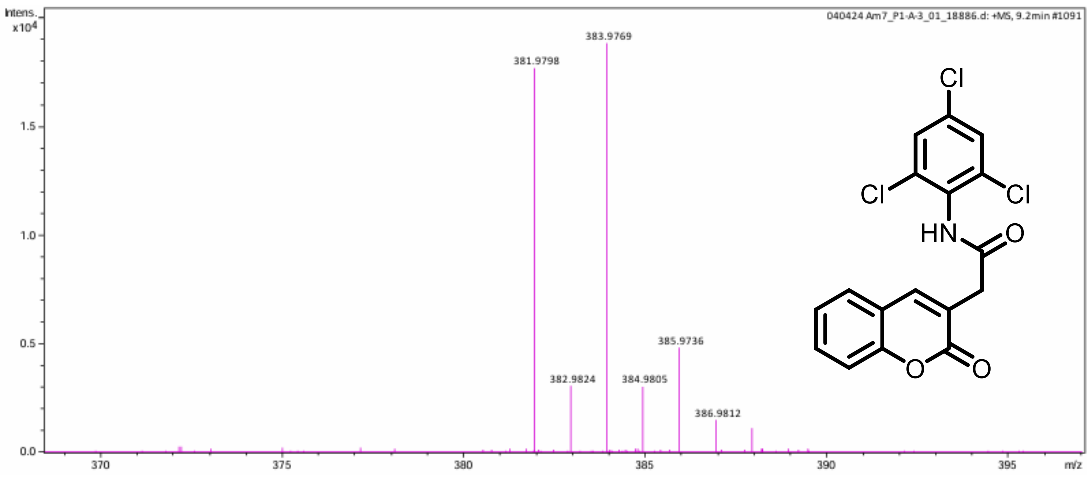

Figure S15. HRMS of **4c**

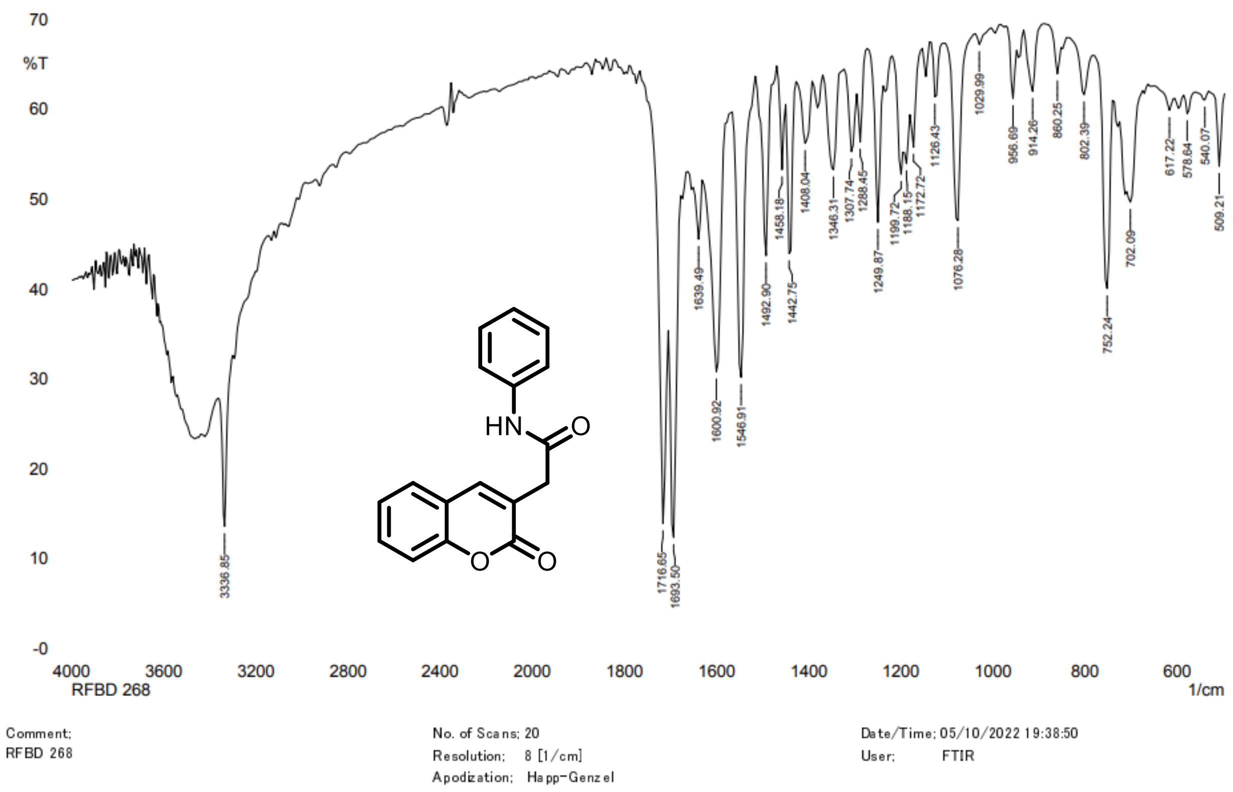

Figure S16. IR (KBr) of **4d**

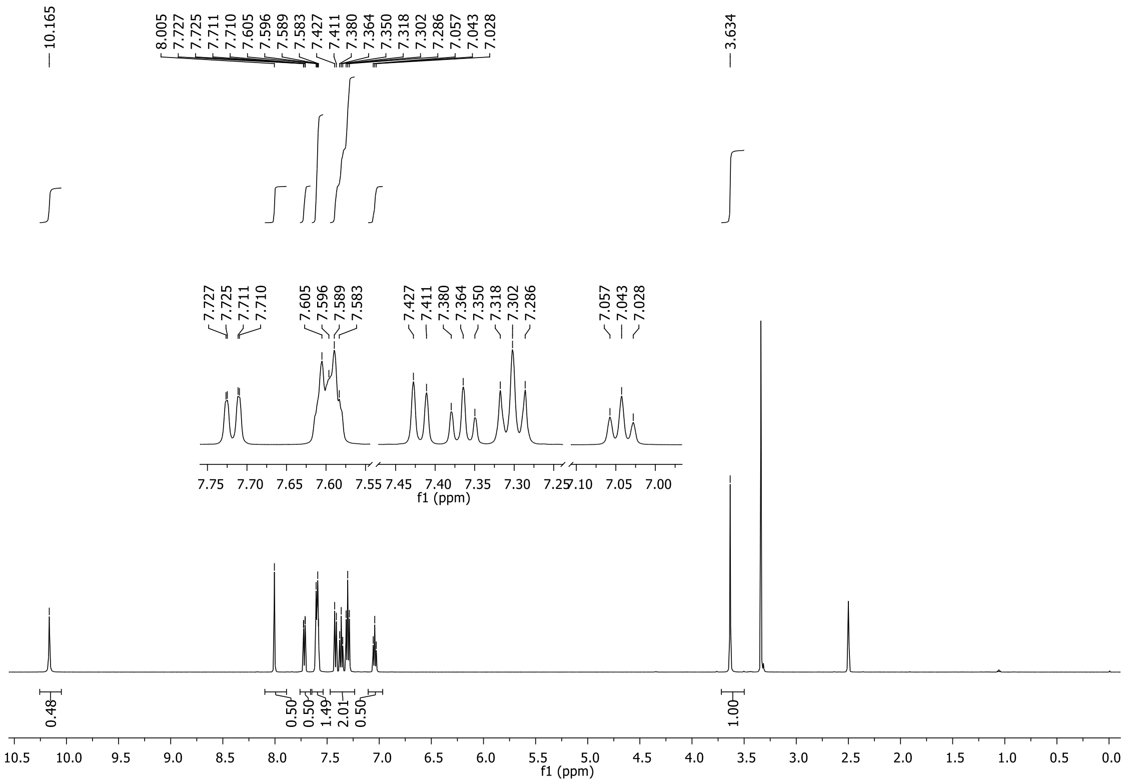

Figure S17. ^{1}H NMR (DMSO-d$_6$, 500 MHz) of **4d**

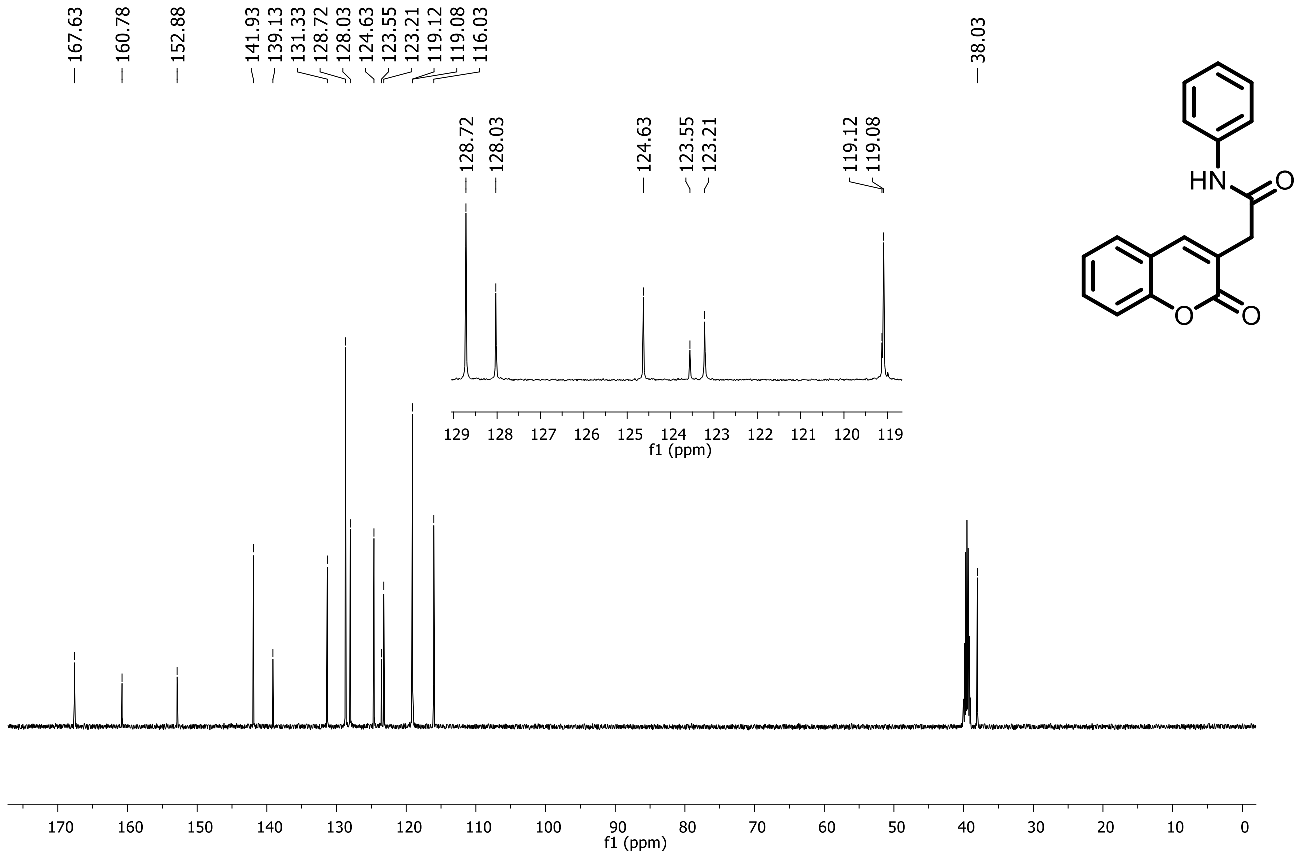

Figure S18. $^{13}C\{^{1}H\}$ NMR (DMSO-d$_6$, 125 MHz) of 4**d**

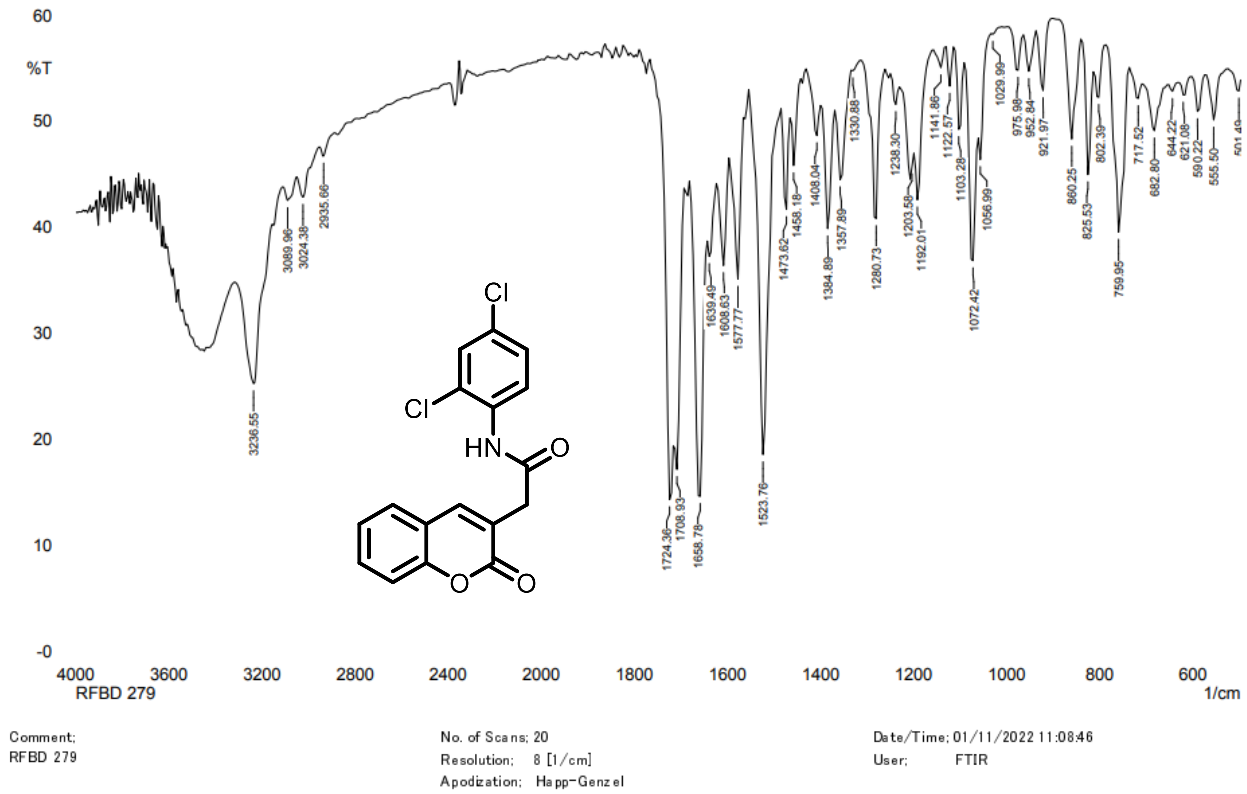

Figure S19. IR (KBr) of **4e**

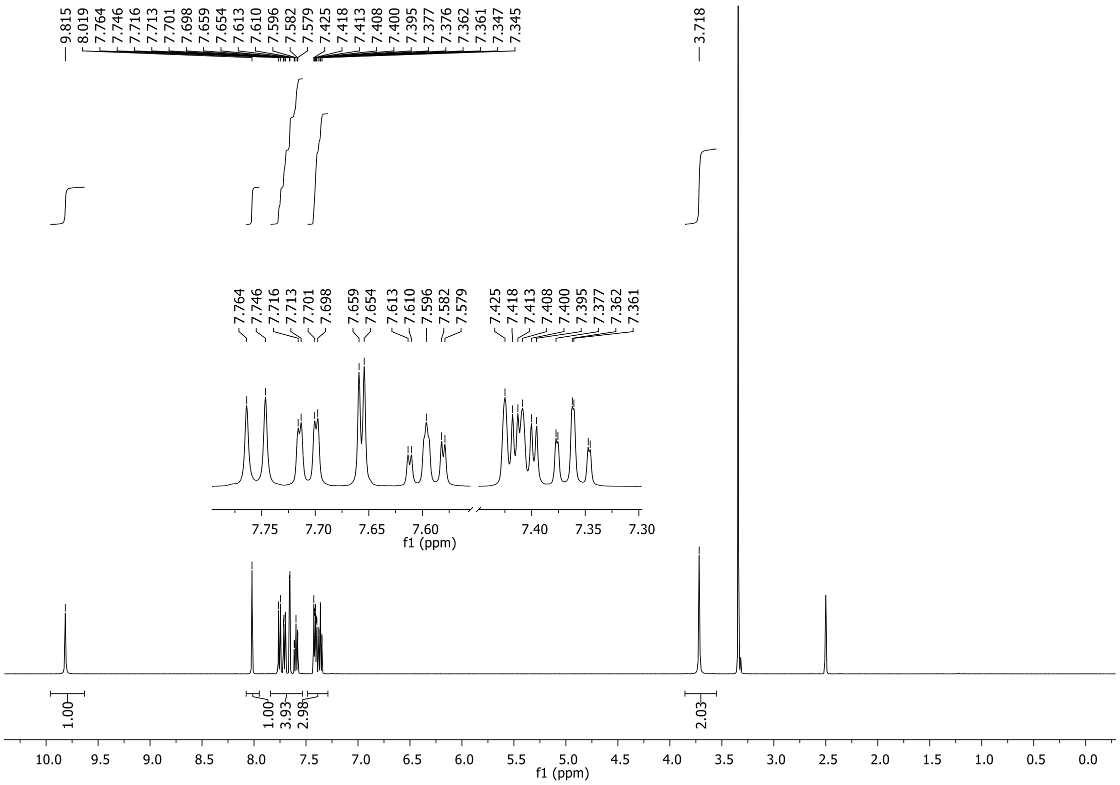

Figure S20. ^{1}H NMR (DMSO-d$_6$, 500 MHz) of **4e**

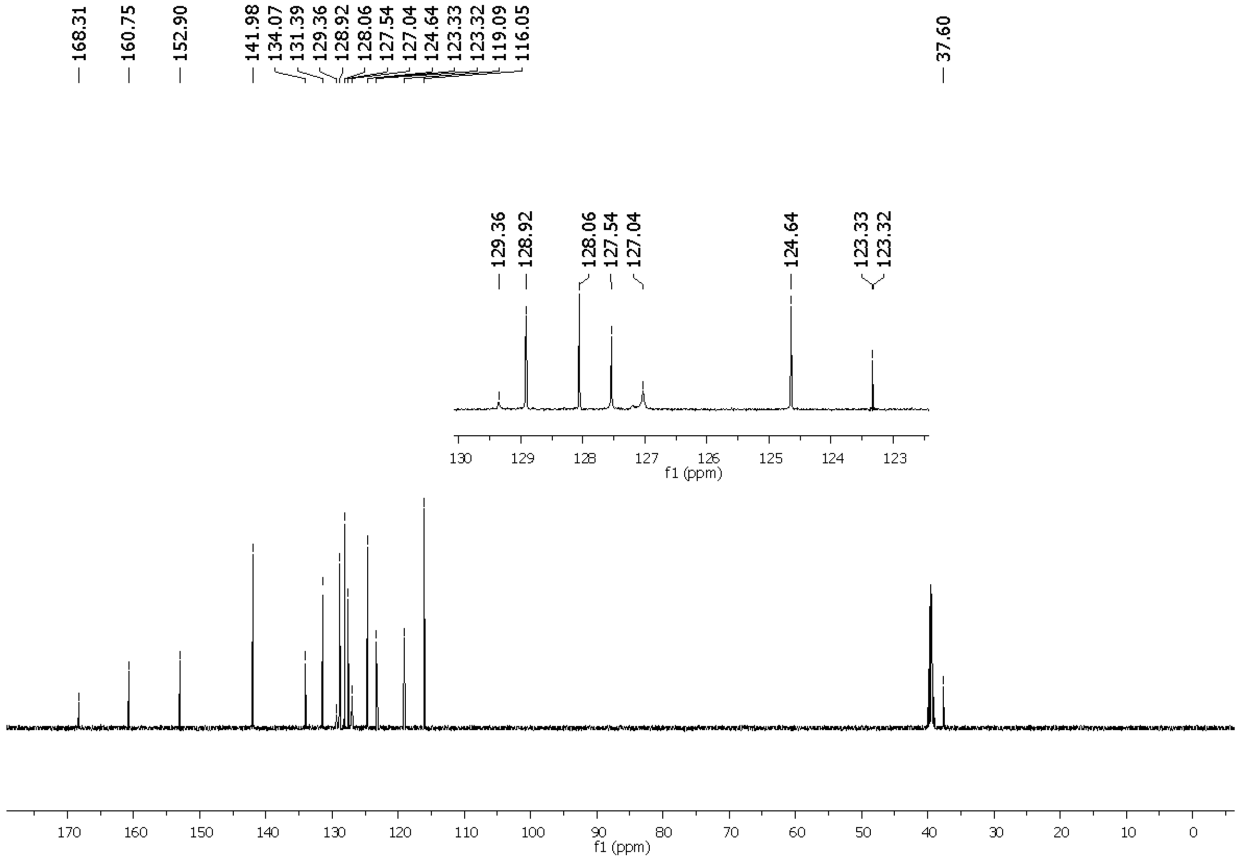

Figure S21. $^{13}C\{^{1}H\}$ NMR (DMSO-d$_6$, 125 MHz) **of 4e**

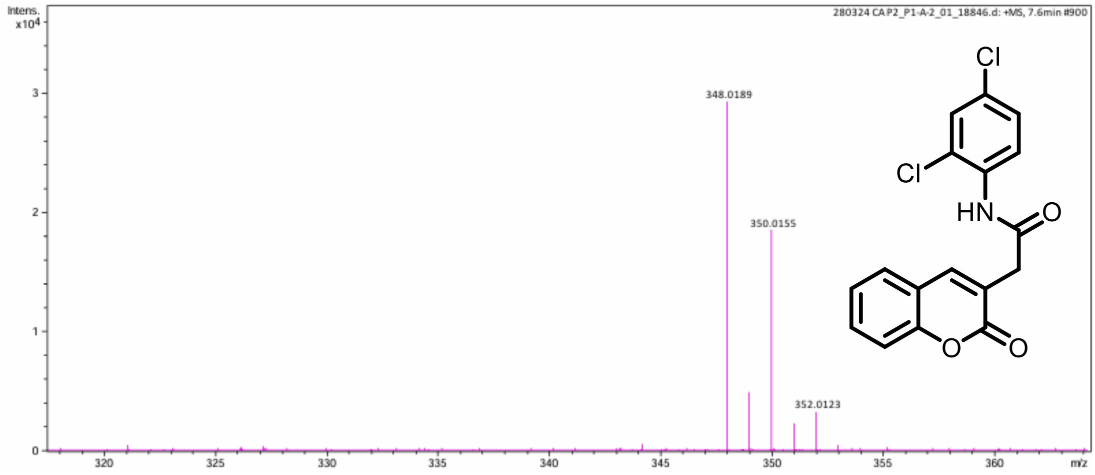

Figure S22. HRMS of **4e**

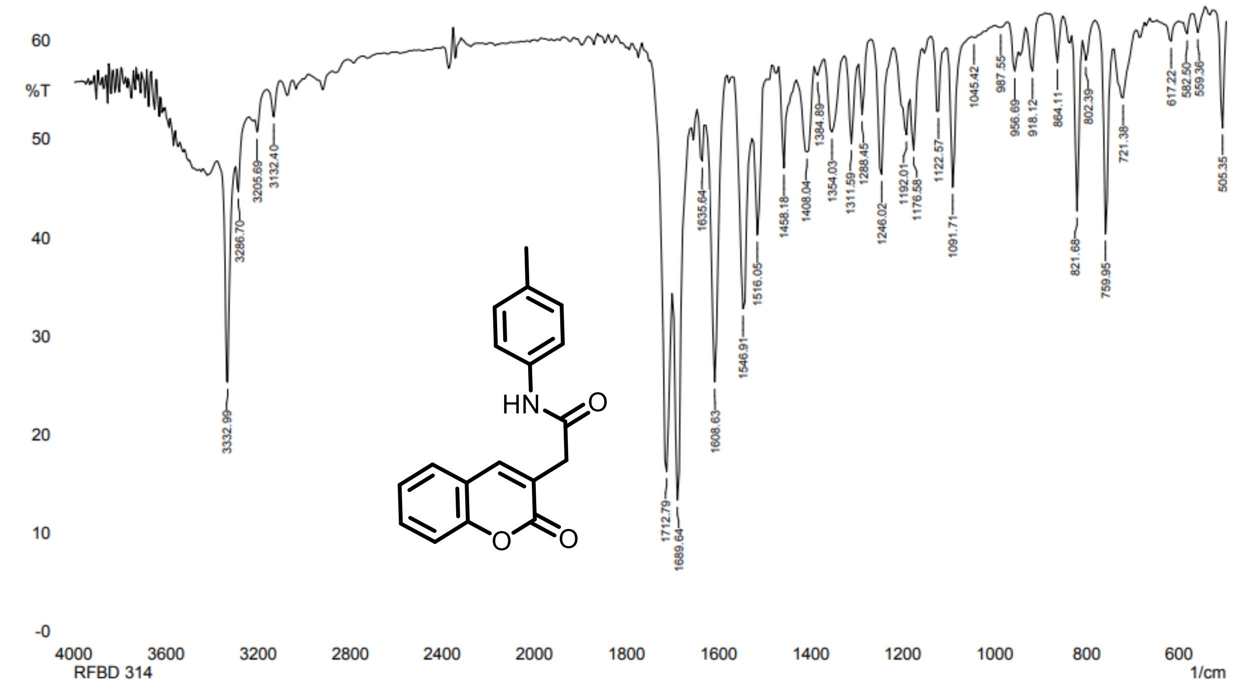

Comment;
RFBD 314

No. of Scans; 20
Resolution; 8 [1/cm]
Apodization; Happ-Genzel

Date/Time; 15/12/2022 18:11:19
User; FTIR

Figure S23. IR (KBr) of **4f**

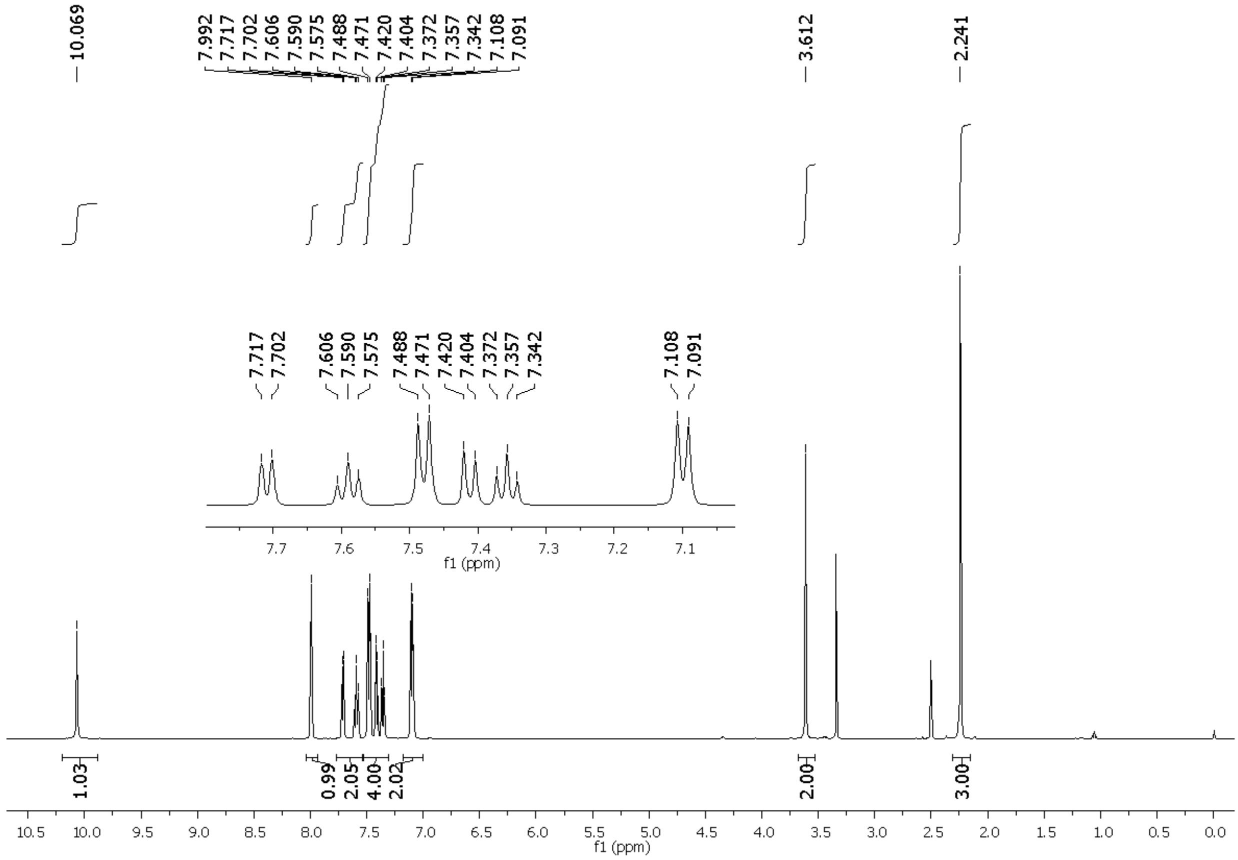

Figure S24. ^{1}H NMR (DMSO-d_6, 500 MHz) of **4f**

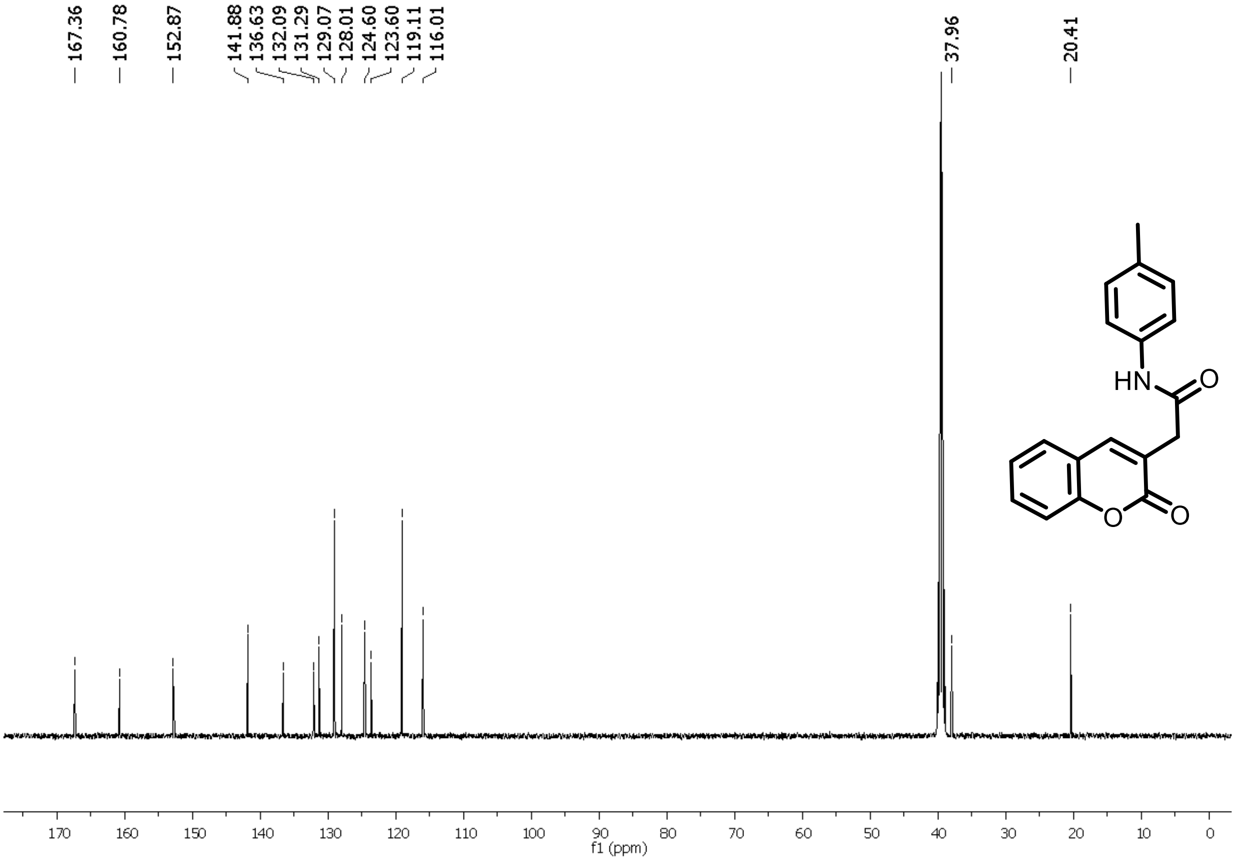

Figure S25. $^{13}C\{^1H\}$ NMR (DMSO-d$_6$, 125 MHz) **of 4f**

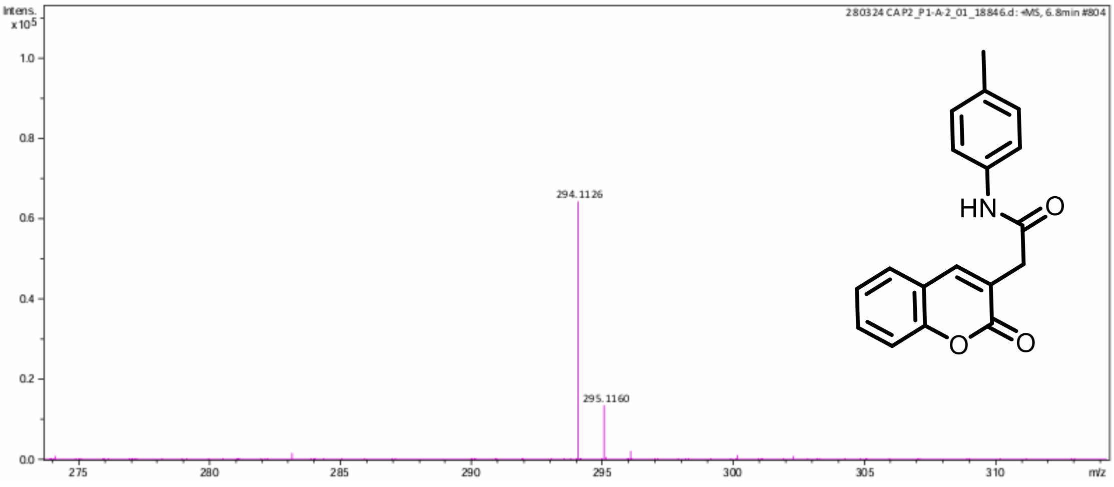

Figure S26. HRMS of **4f**

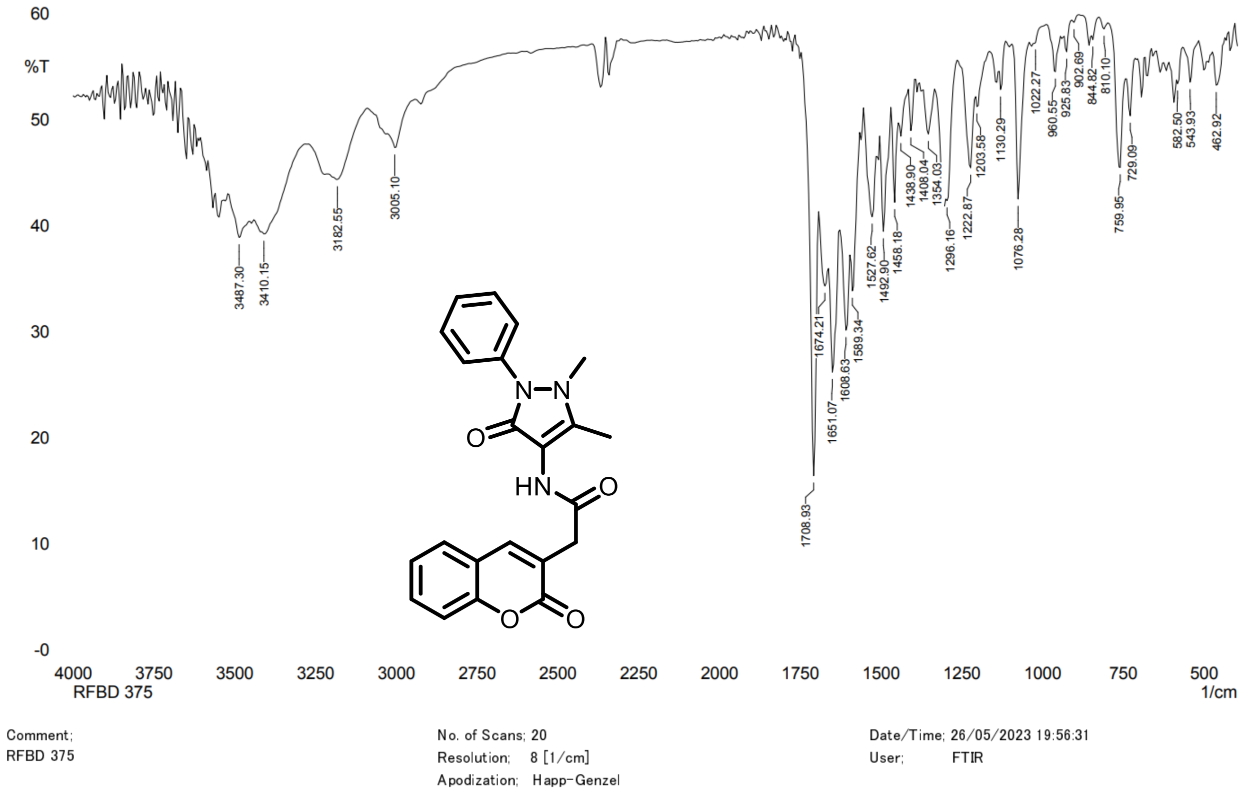

Figure S27. IR (KBr) of **4g**

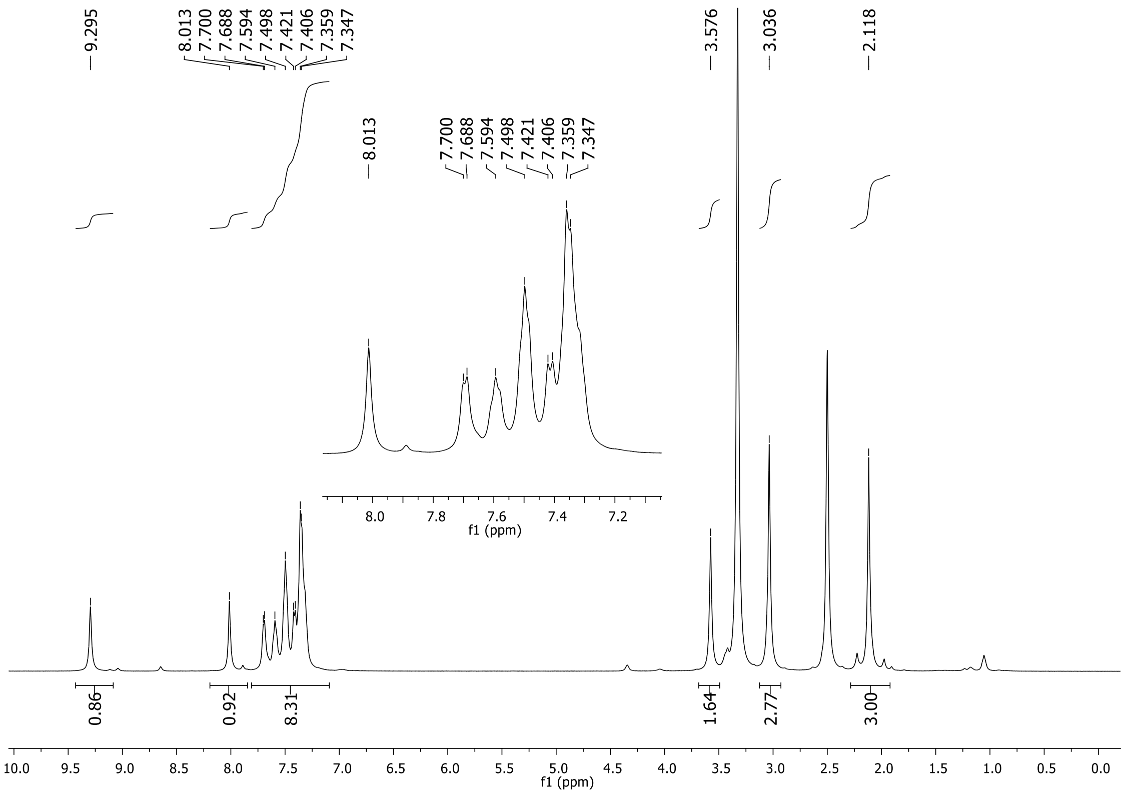

Figure S28. ^{1}H NMR (DMSO-d$_6$, 500 MHz) of **4g**

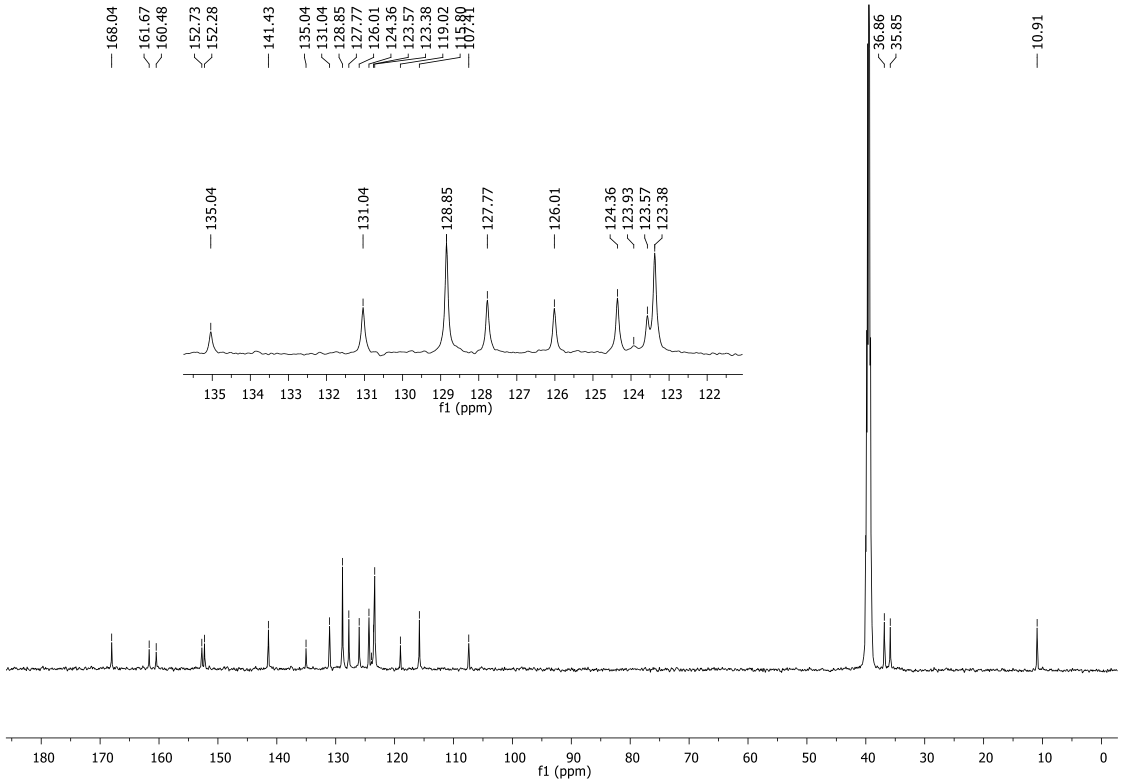

Figure S29. $^{13}C\{^{1}H\}$ NMR (DMSO-d$_6$, 125 MHz) **of 4g**

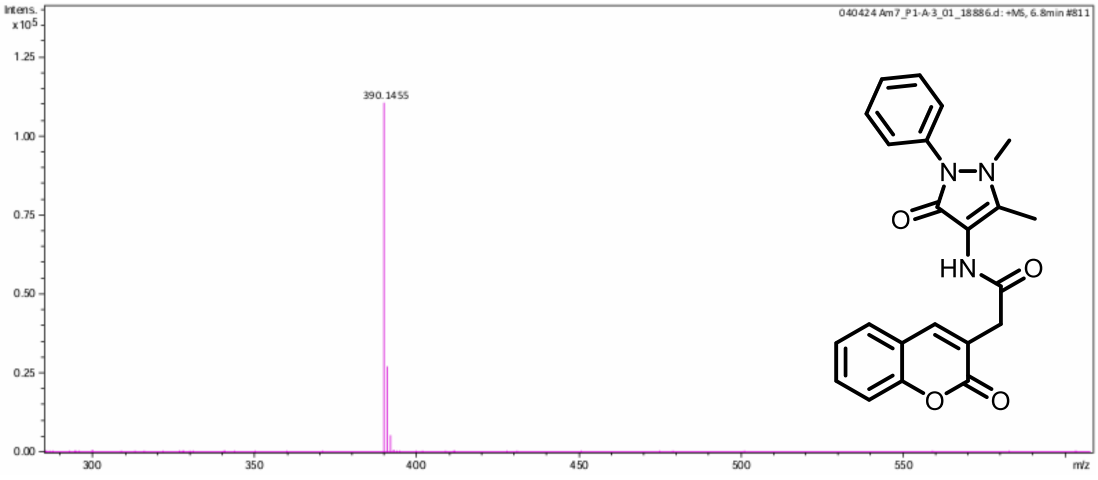

Figure S30. HRMS of **4g**

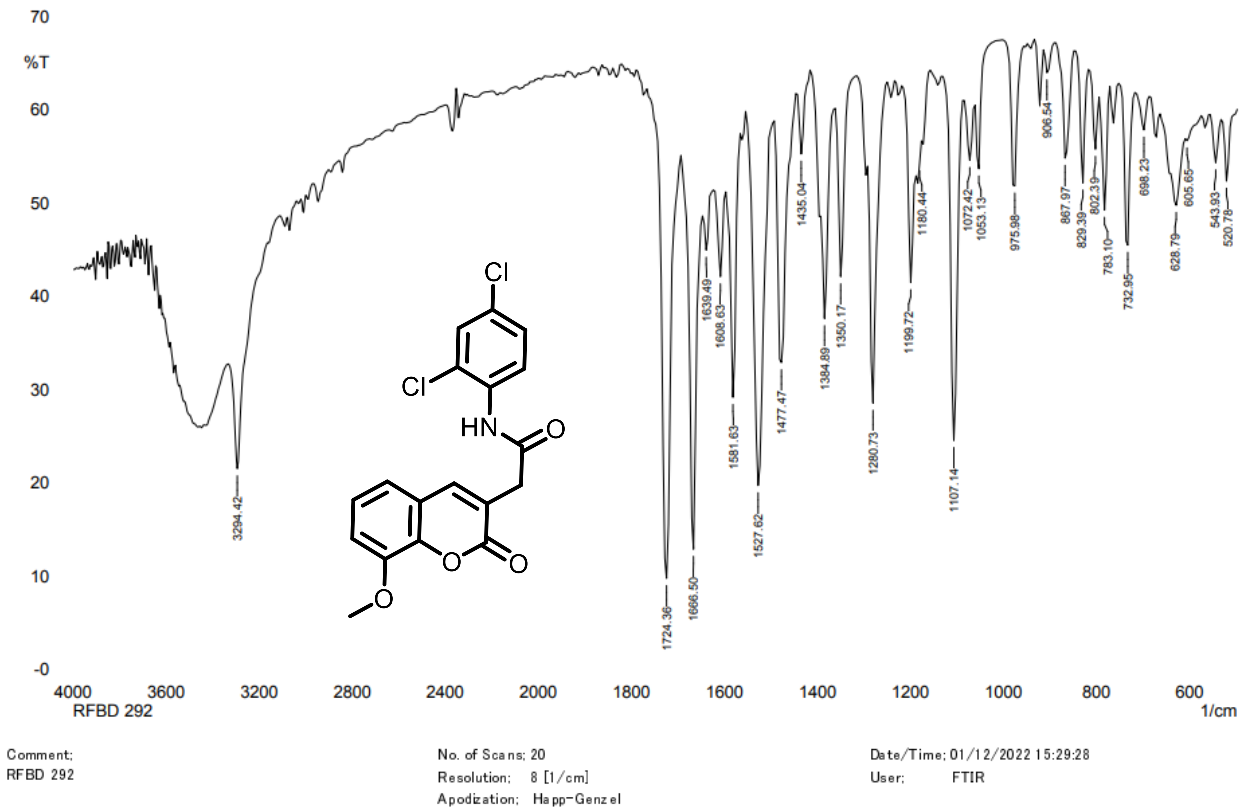

Figure S31. IR (KBr) of **4h**

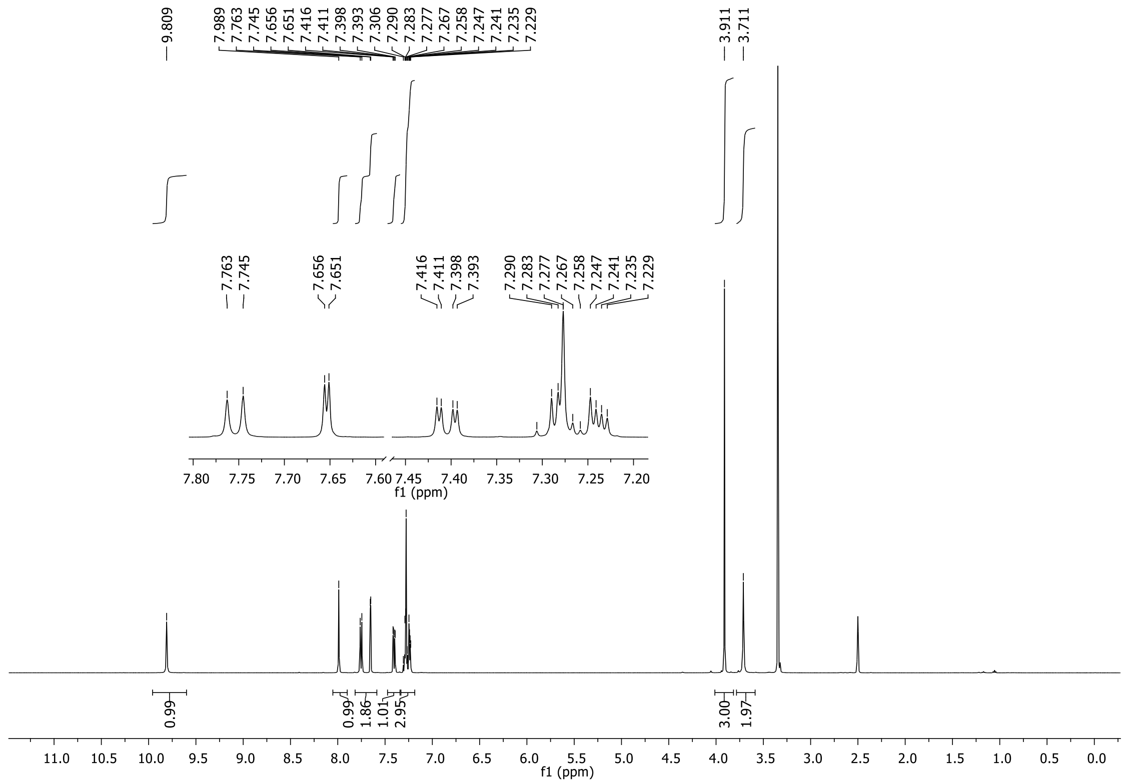

Figure S32. ^{1}H NMR (DMSO-d$_6$, 500 MHz) of **4h**

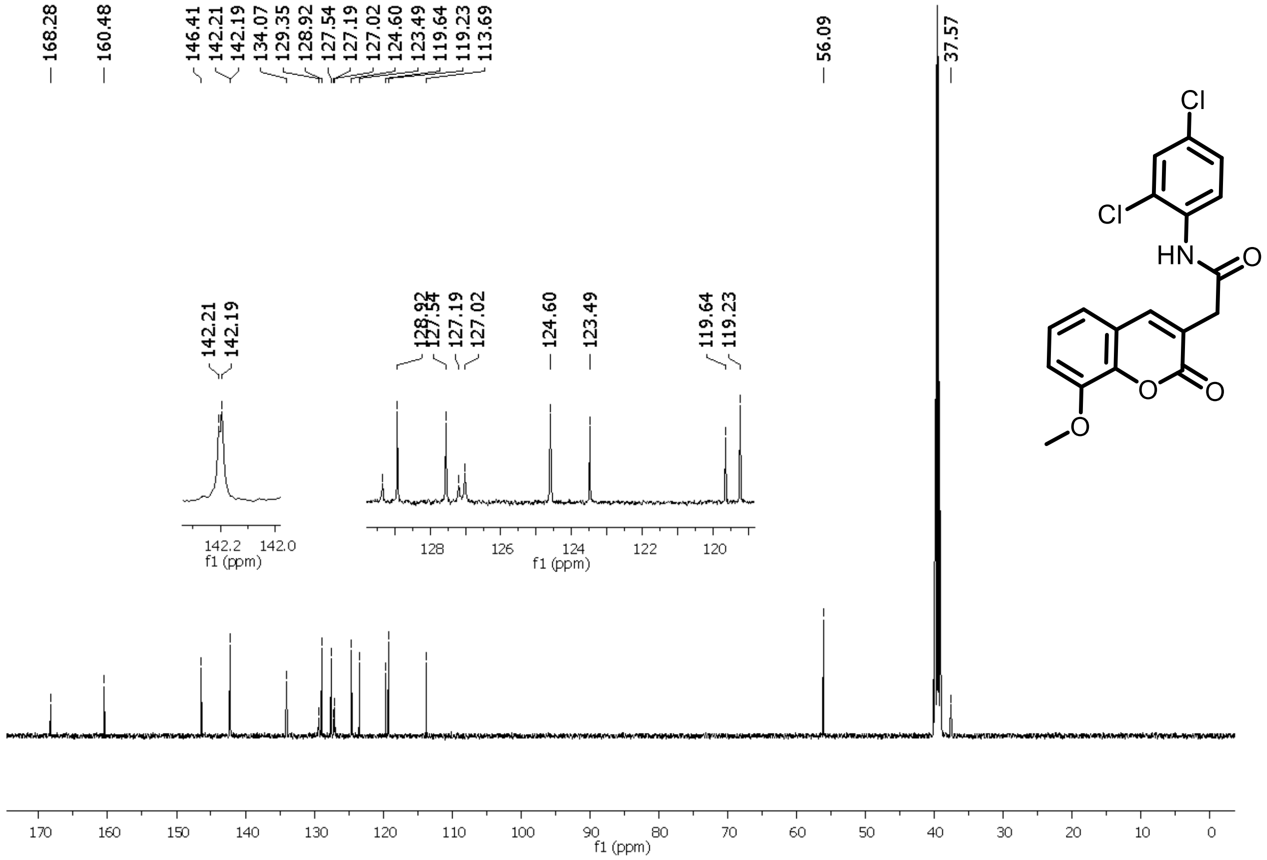

Figure S33. $^{13}C\{^{1}H\}$ NMR (DMSO-d_6, 125 MHz) **of 4h**

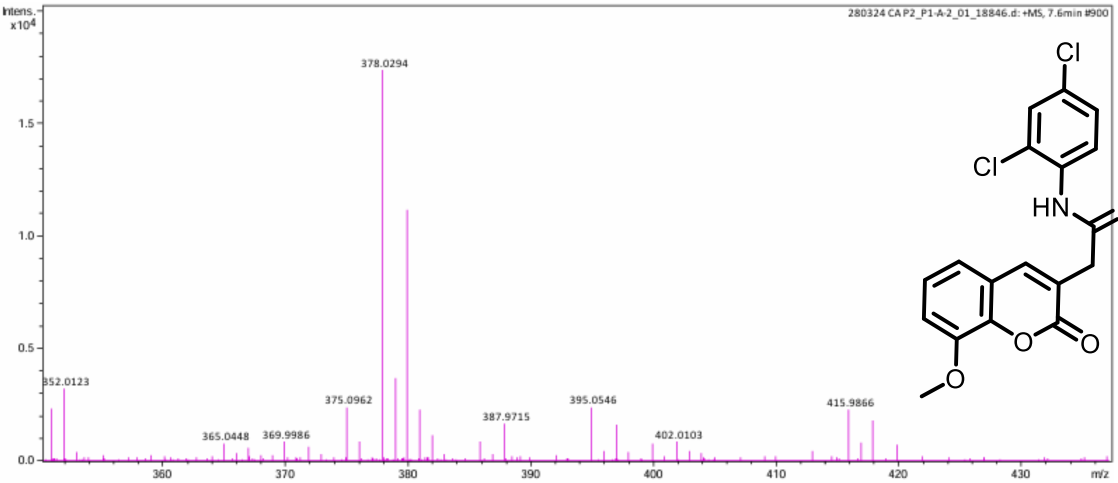

Figure S34. HRMS of **4h**

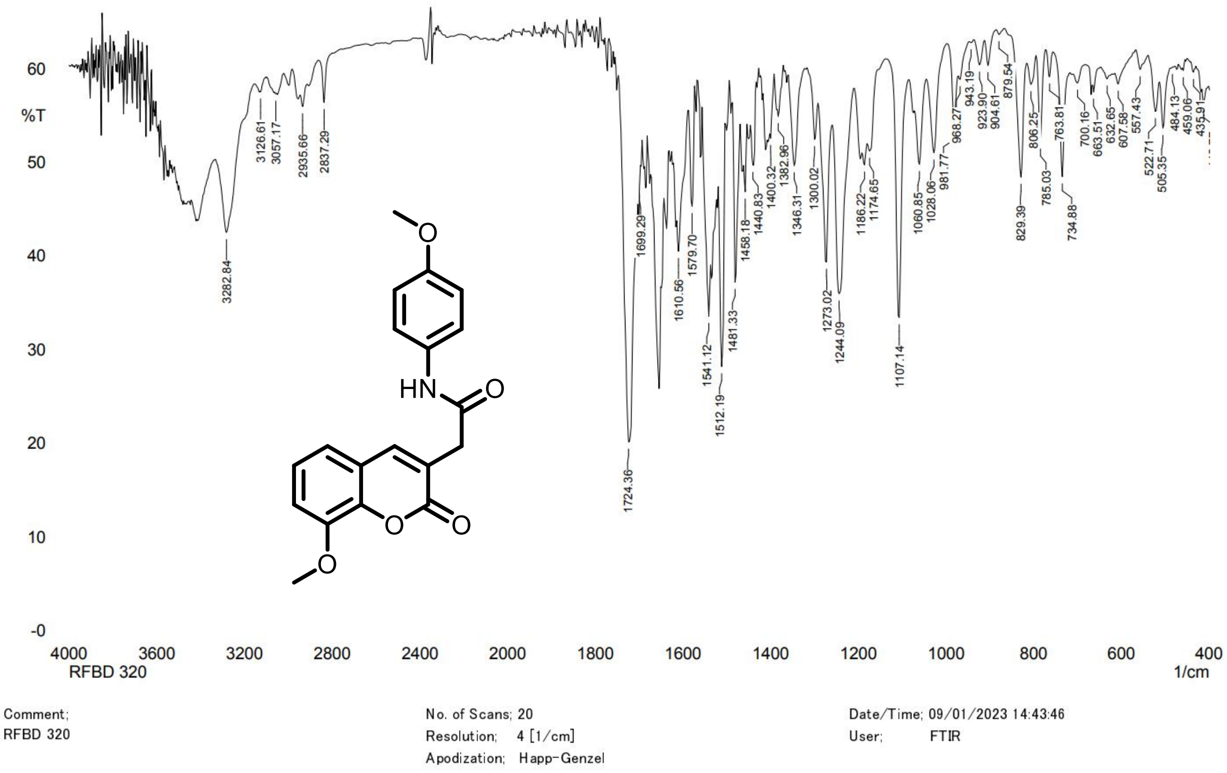

Figure S35. IR (KBr) of **4i**

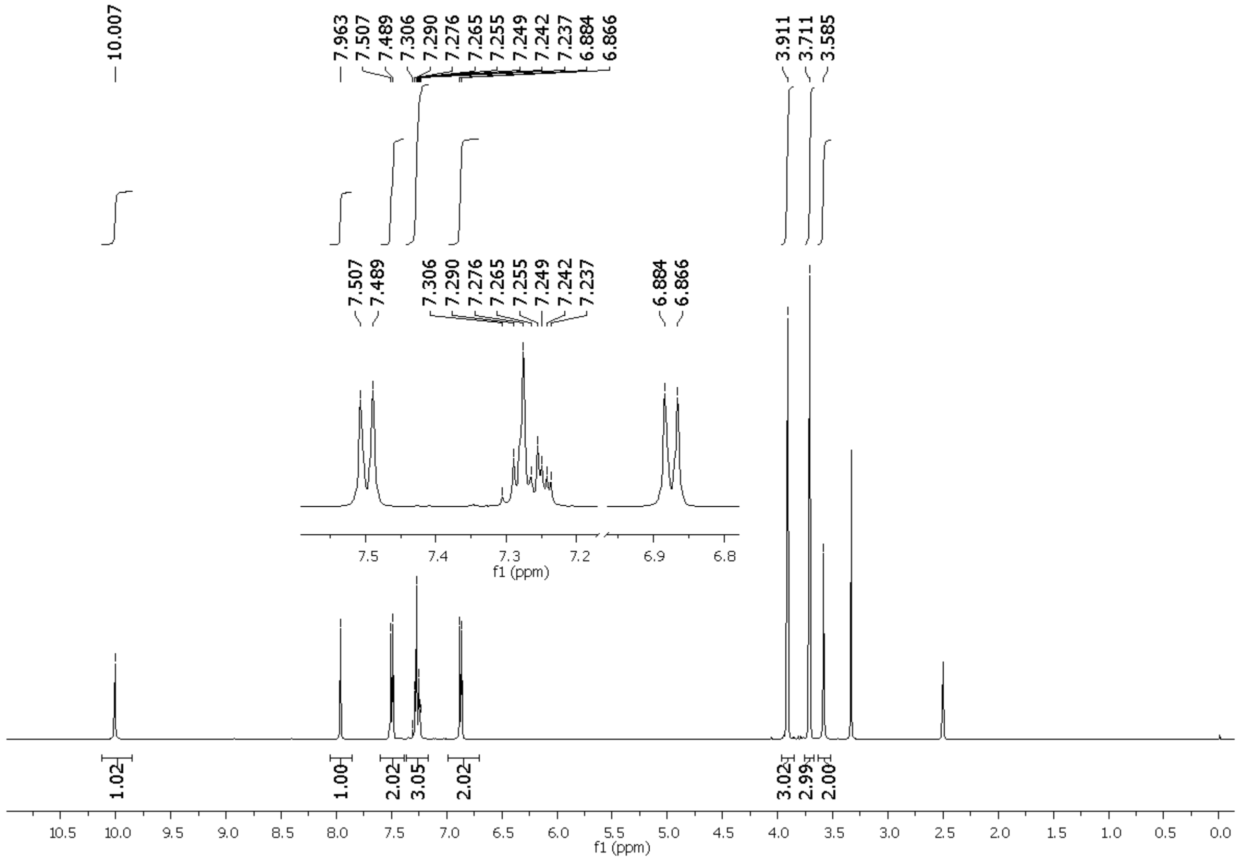

Figure S36. ^{1}H NMR (DMSO-d$_6$, 500 MHz) of **4i**

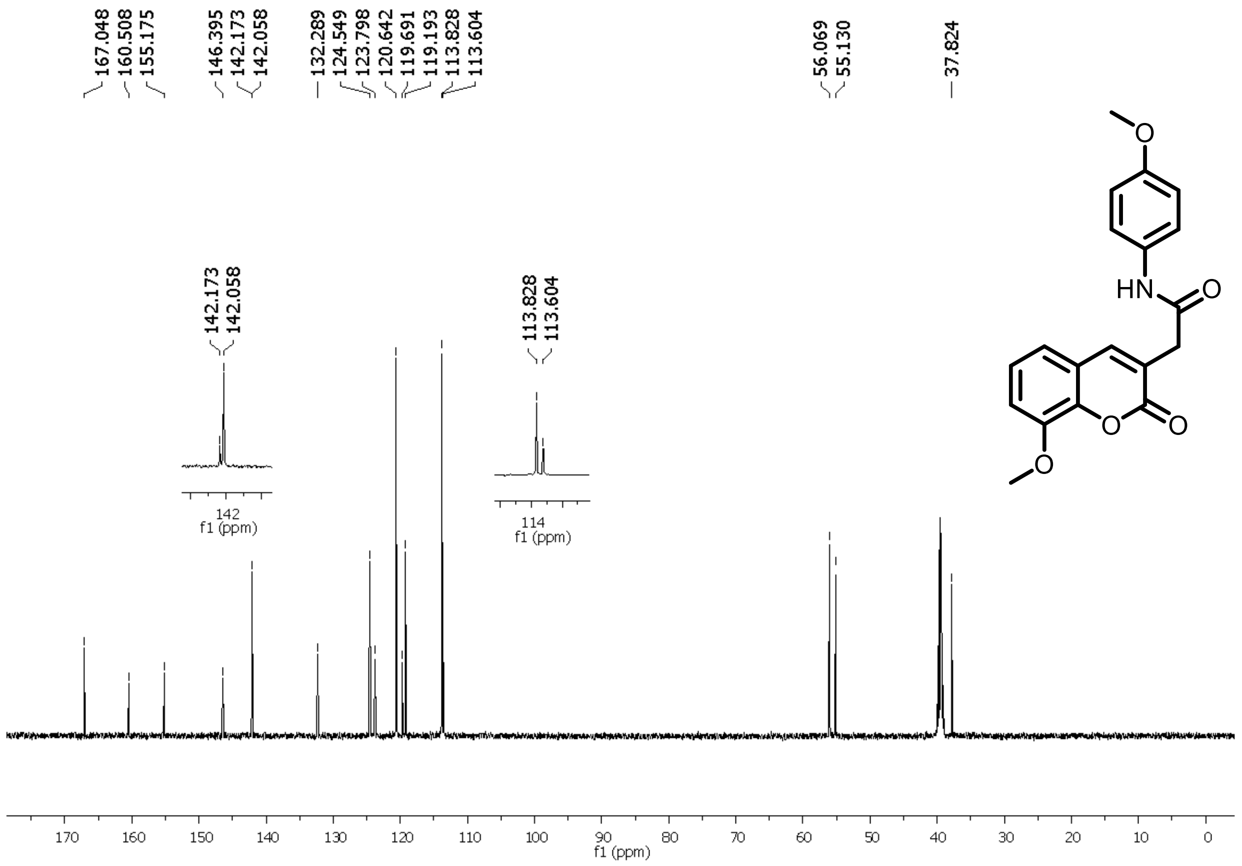

Figure S37. $^{13}C\{^{1}H\}$ NMR (DMSO-d_6, 125 MHz) **of 4i**

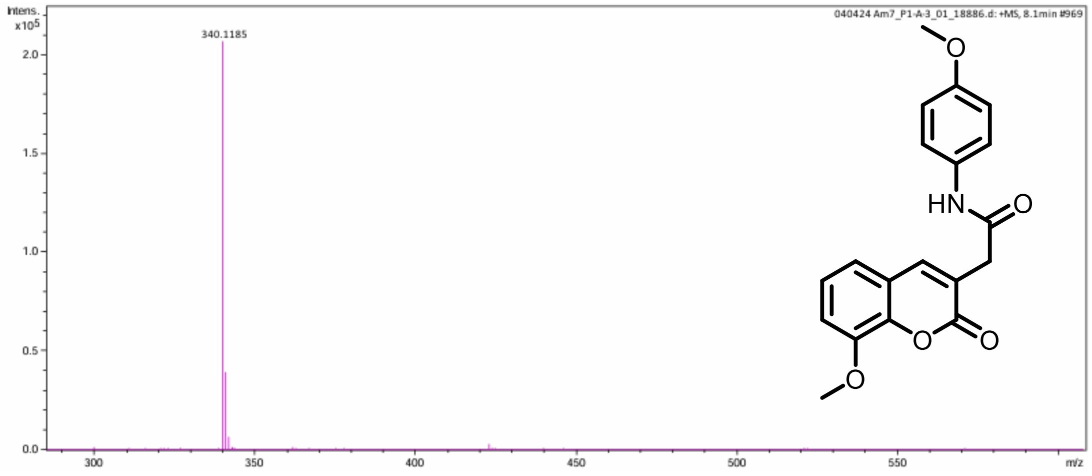

Figure S38. HRMS of **4i**

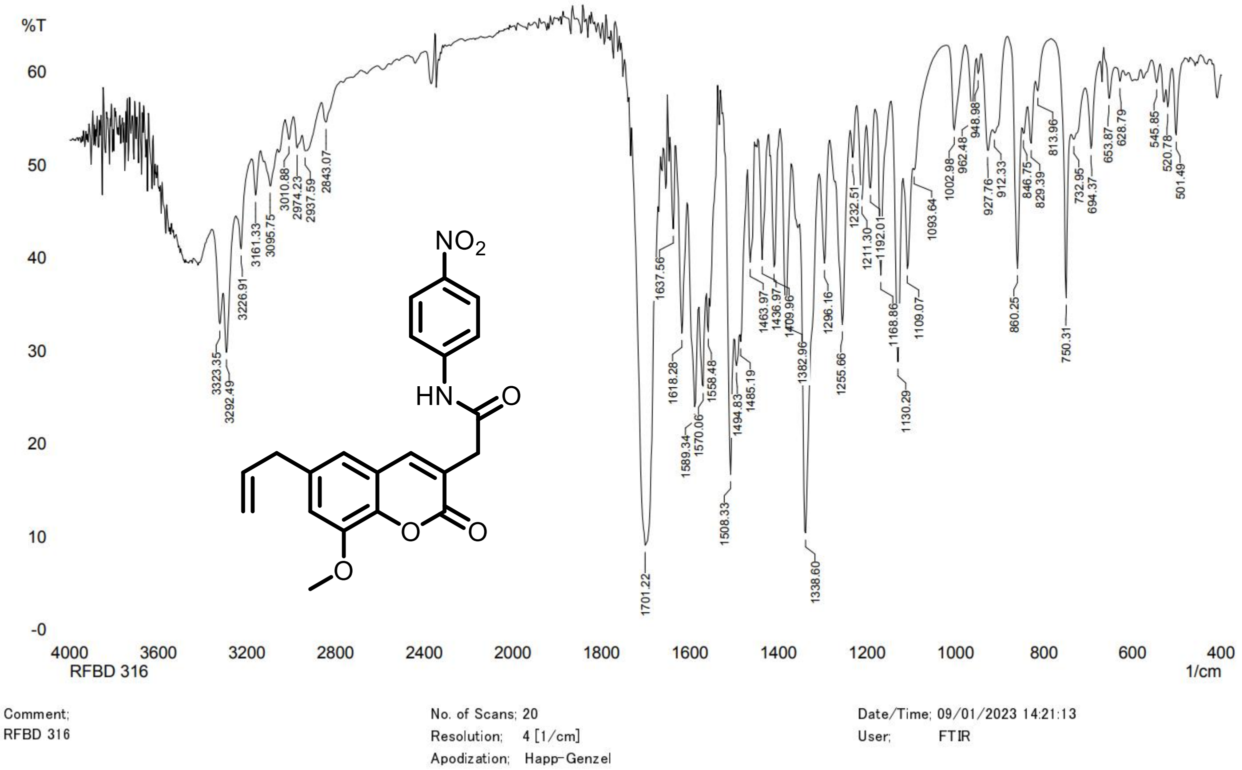

Figure S39. IR (KBr) of **4j**

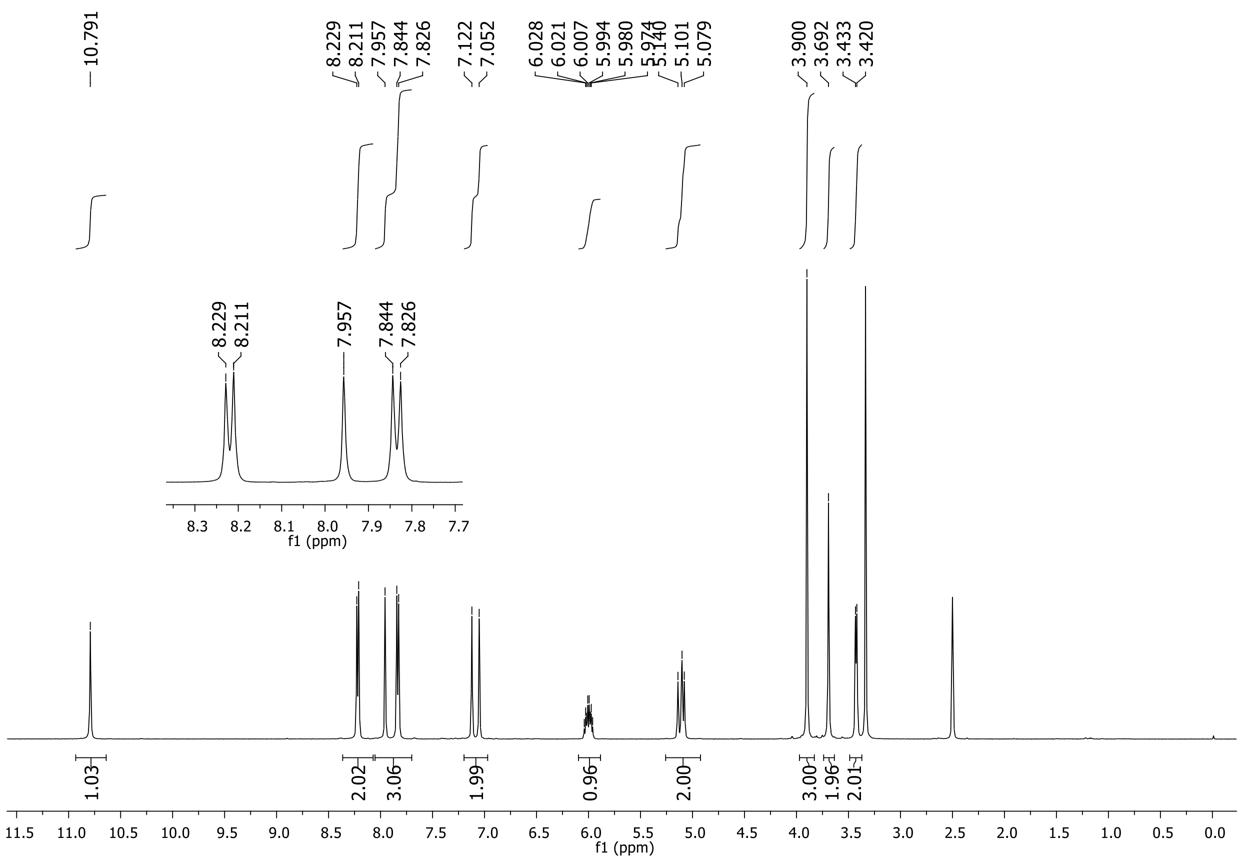

Figure S40. ^{1}H NMR (DMSO-d$_6$, 500 MHz) of **4j**

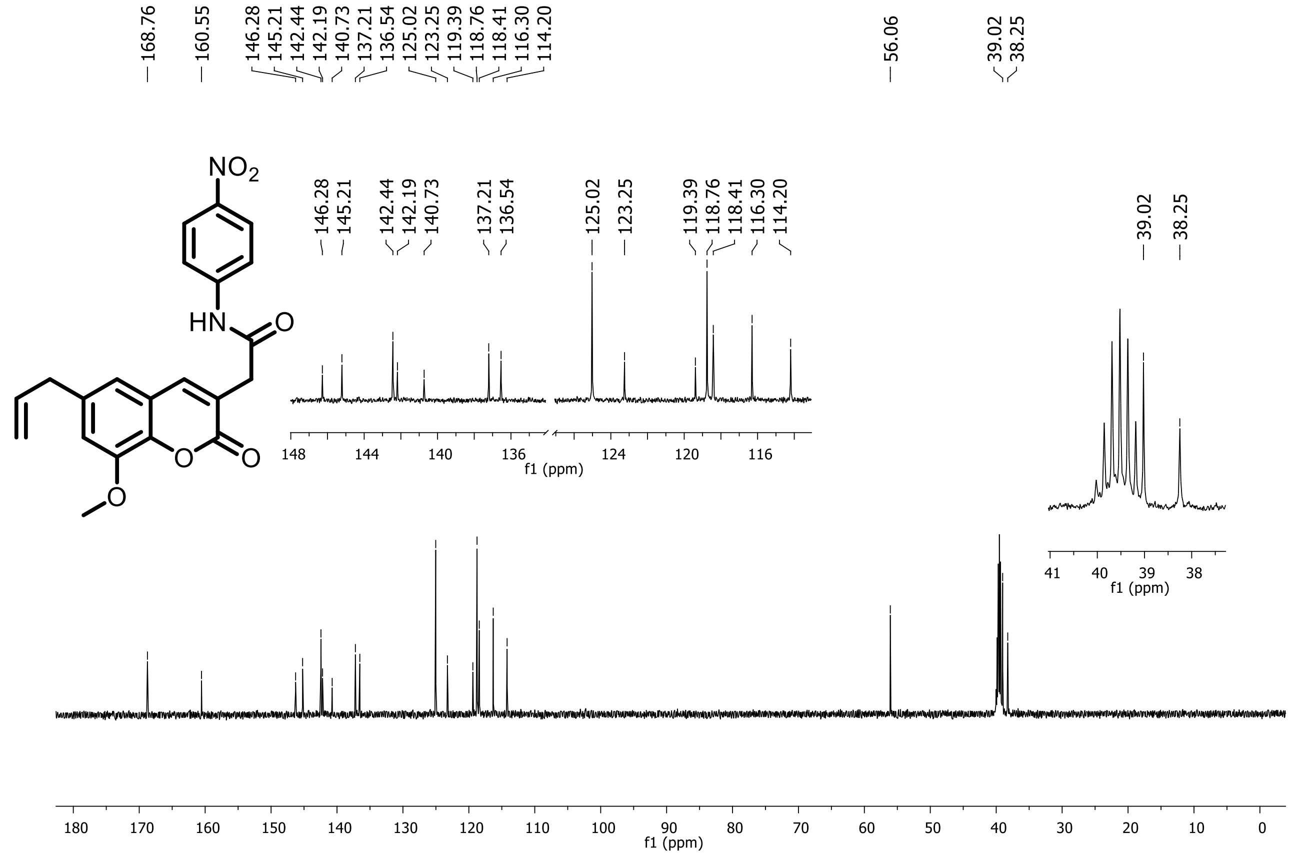

Figure S41. $^{13}C\{^{1}H\}$ NMR (DMSO-d$_6$, 125 MHz) of **4j**

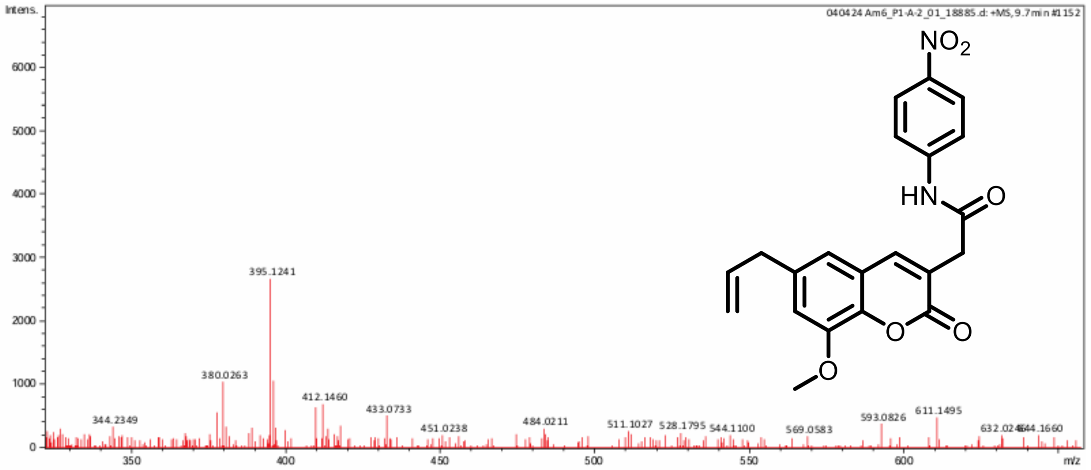

Figure S42. HRMS of **4j**

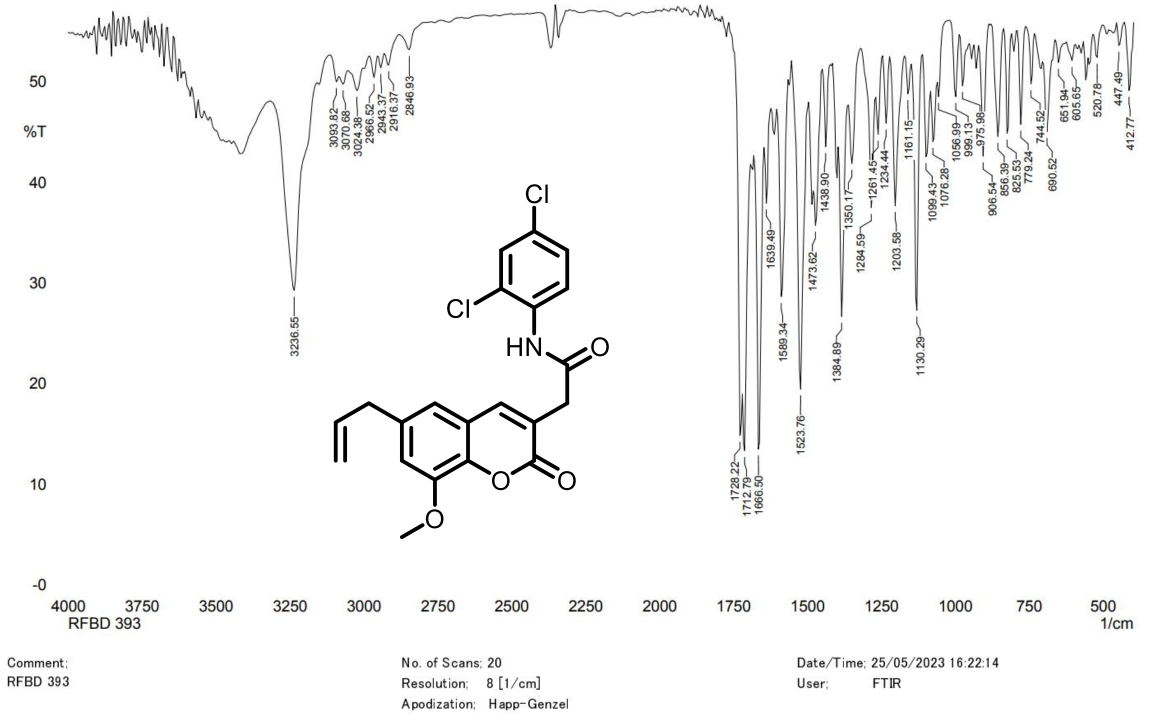

Figure S43. IR (KBr) of **4k**

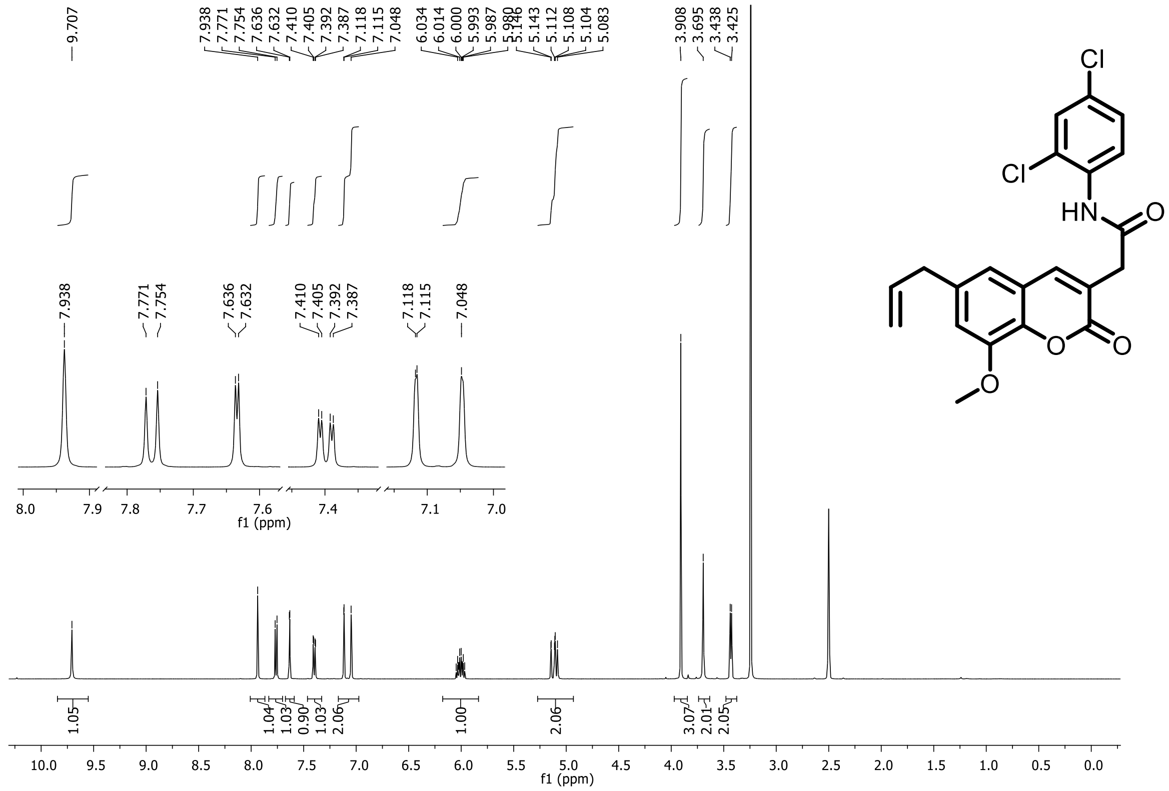

Figure S44. ^{1}H NMR (DMSO-d$_6$, 500 MHz) of **4k**

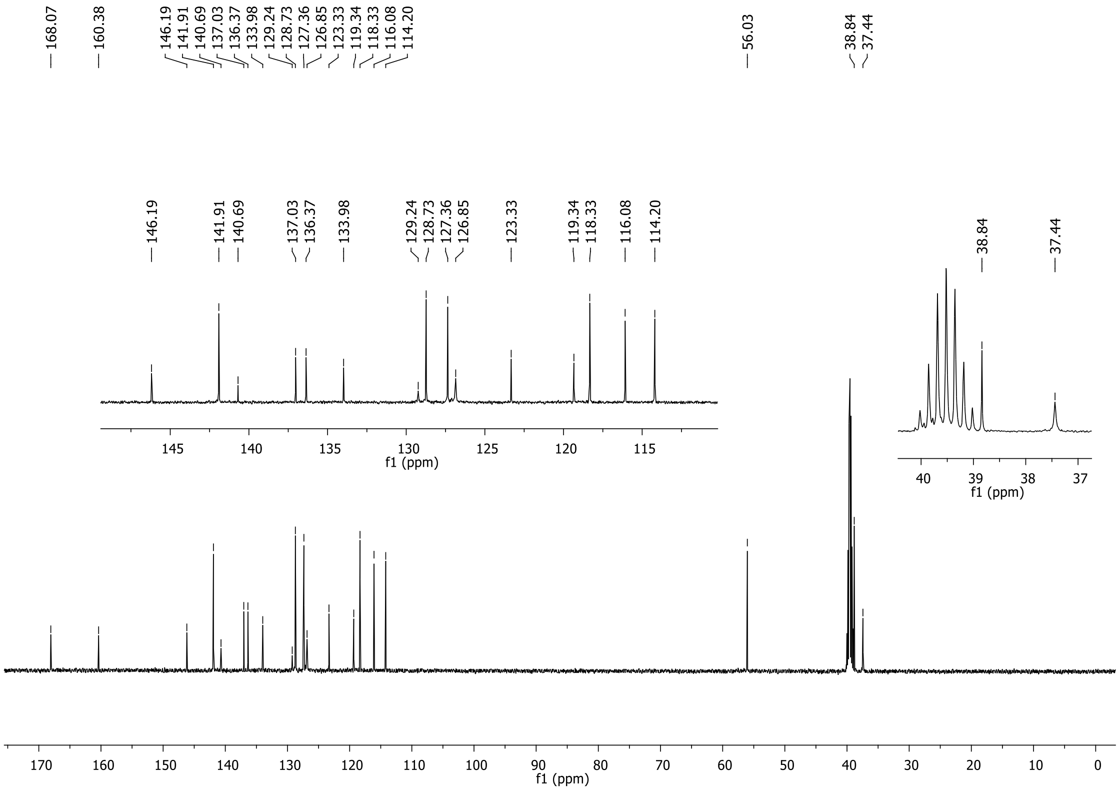

Figure S45. $^{13}C\{^{1}H\}$ NMR (DMSO-d$_6$, 125 MHz) of **4k**

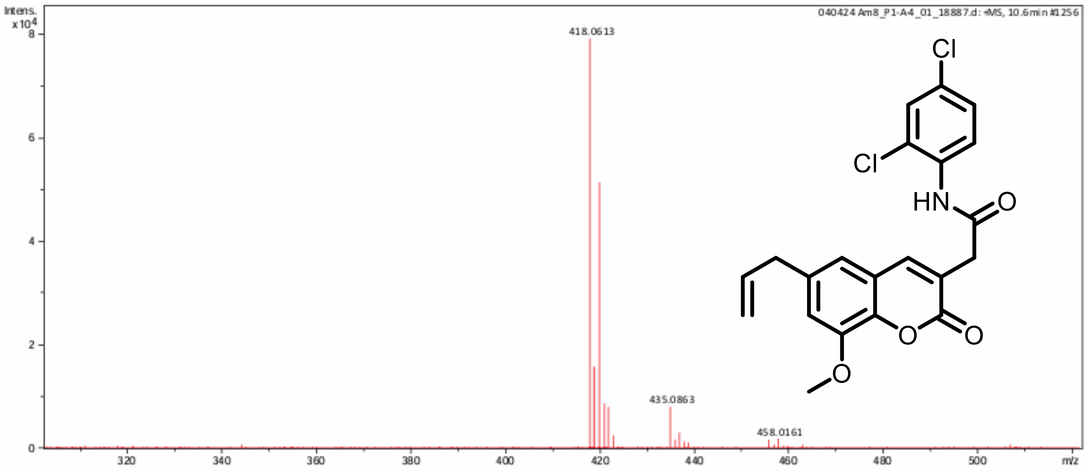

Figure S46. HRMS of **4k**

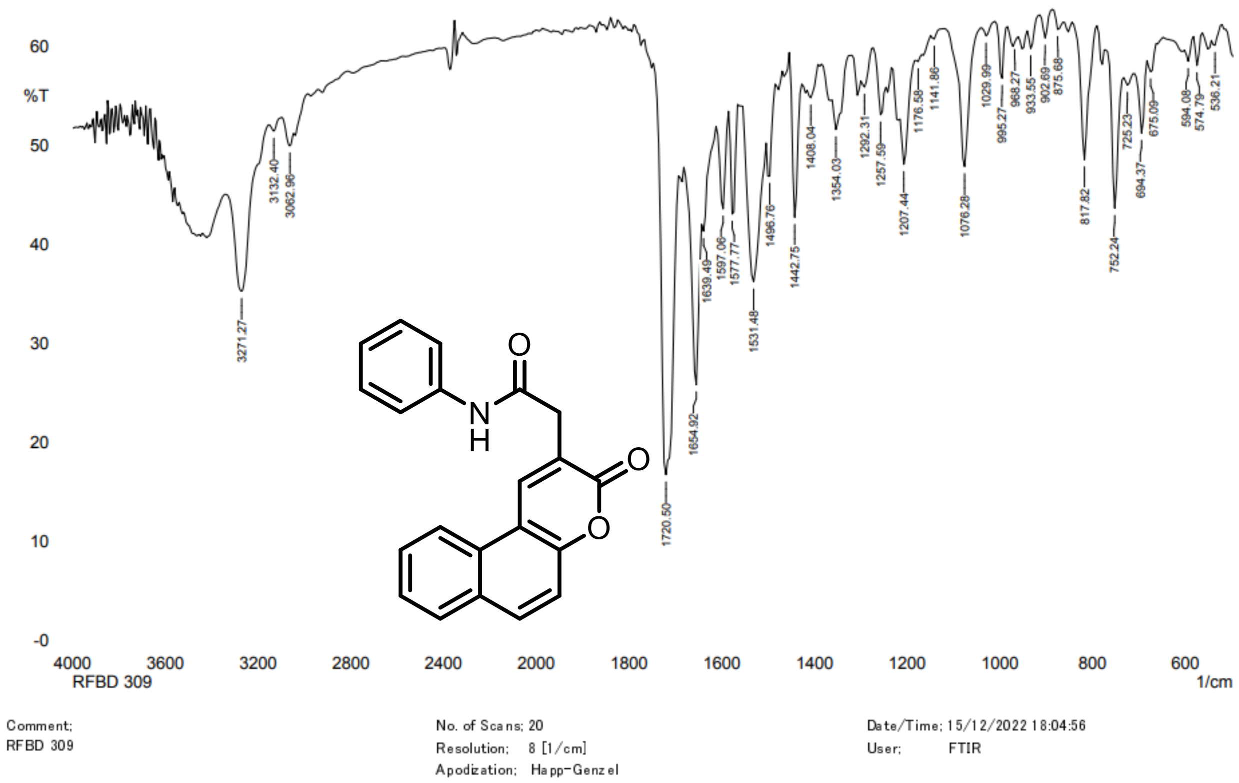

Figure S47. IR (KBr) of **4l**

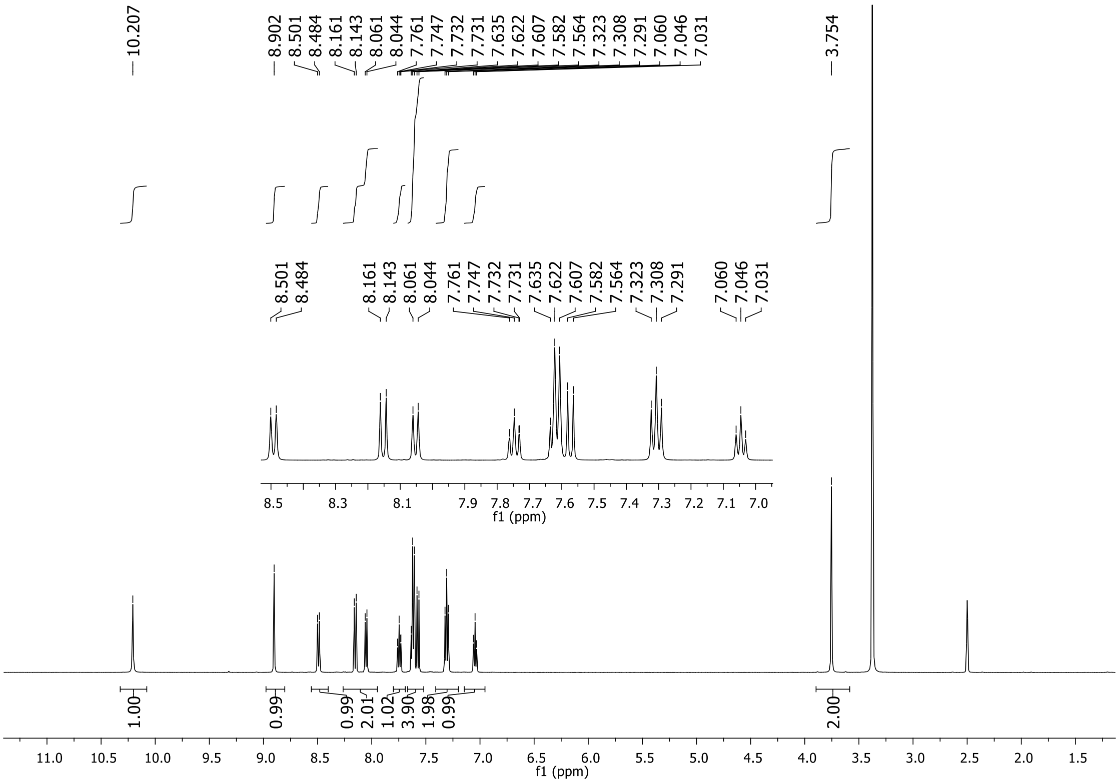

Figure S48. ^{1}H NMR (DMSO-d_6, 500 MHz) of **4l**

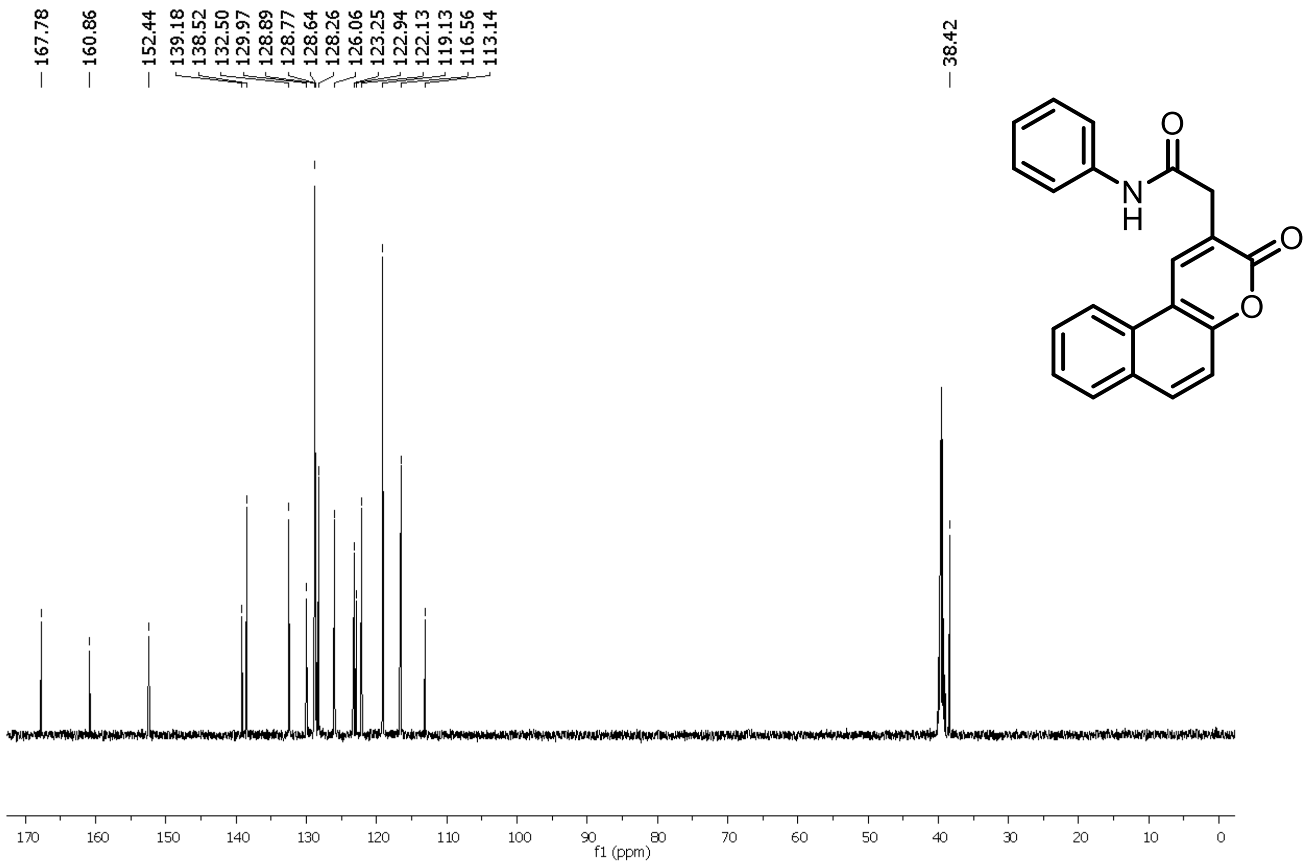

Figure S49. $^{13}C\{^1H\}$ NMR (DMSO-d$_6$, 125 MHz) of **4l**

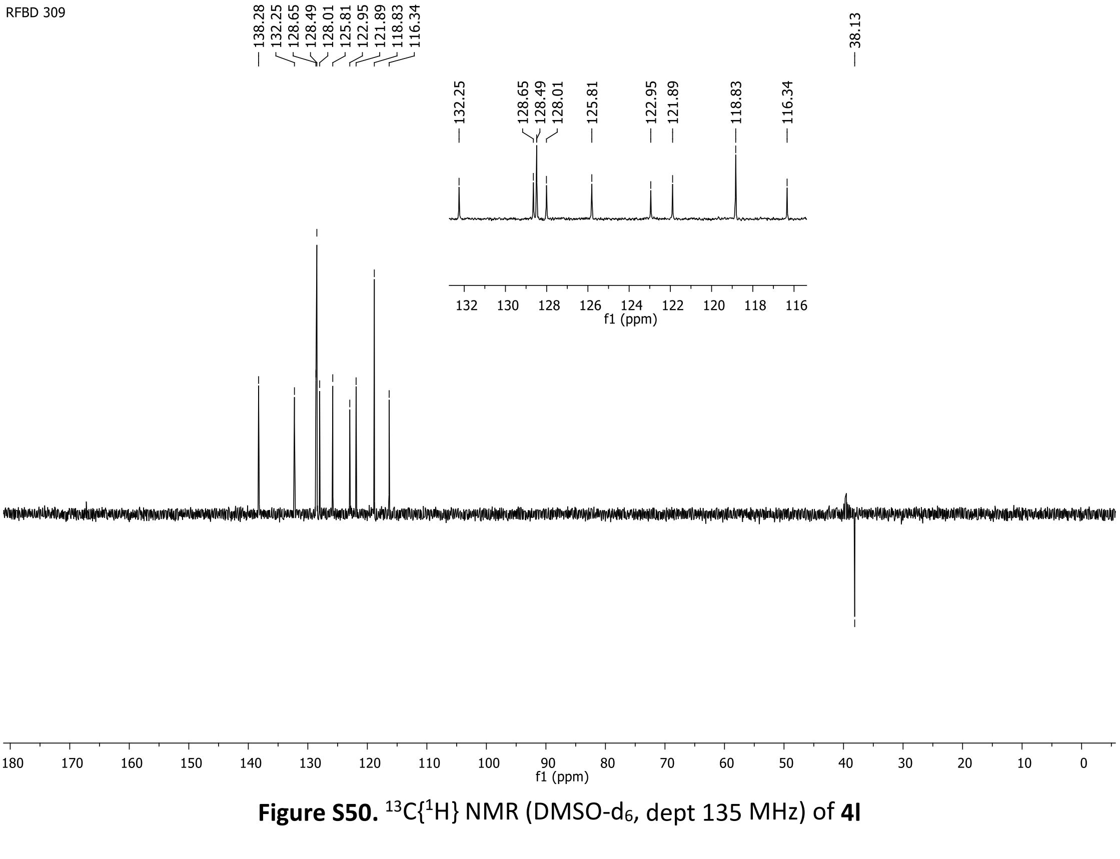

Figure S50. $^{13}C\{^1H\}$ NMR (DMSO-d$_6$, dept 135 MHz) of **4l**

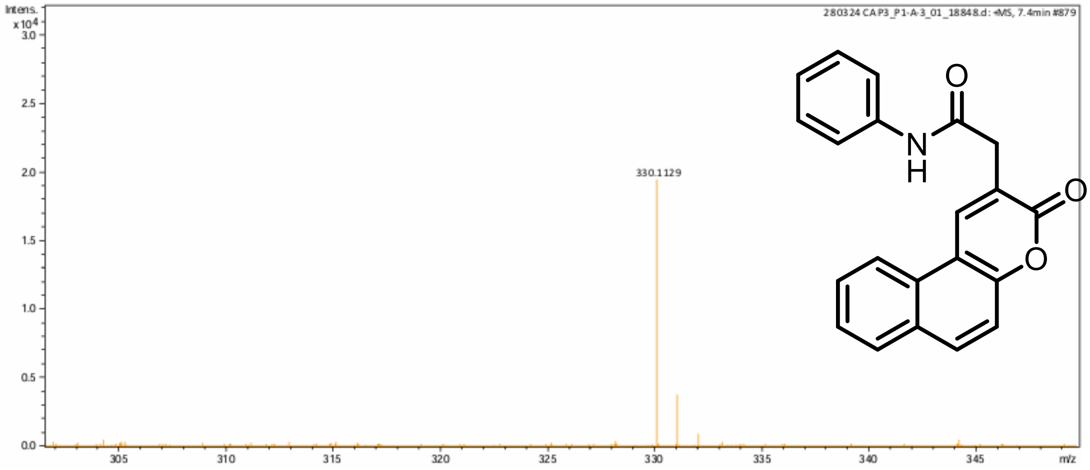

Figure S51. HRMS of **4l**

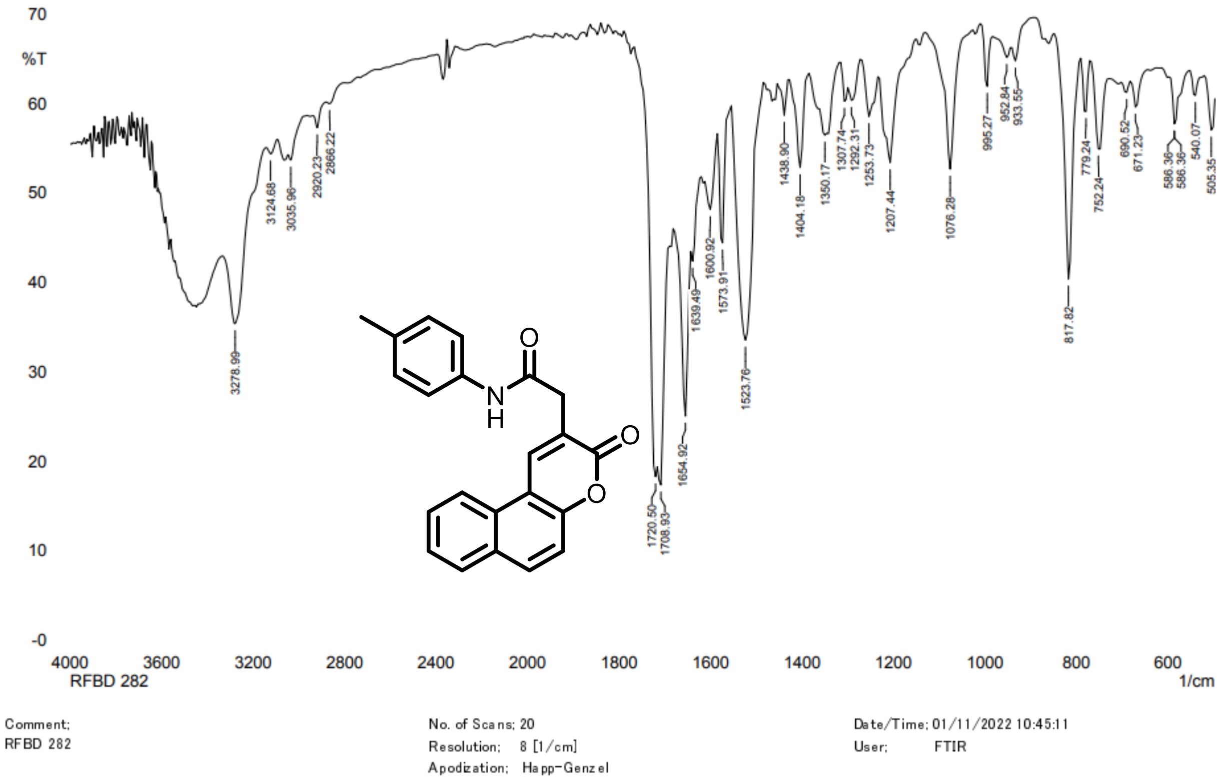

Figure S52. IR (KBr) of **4m**

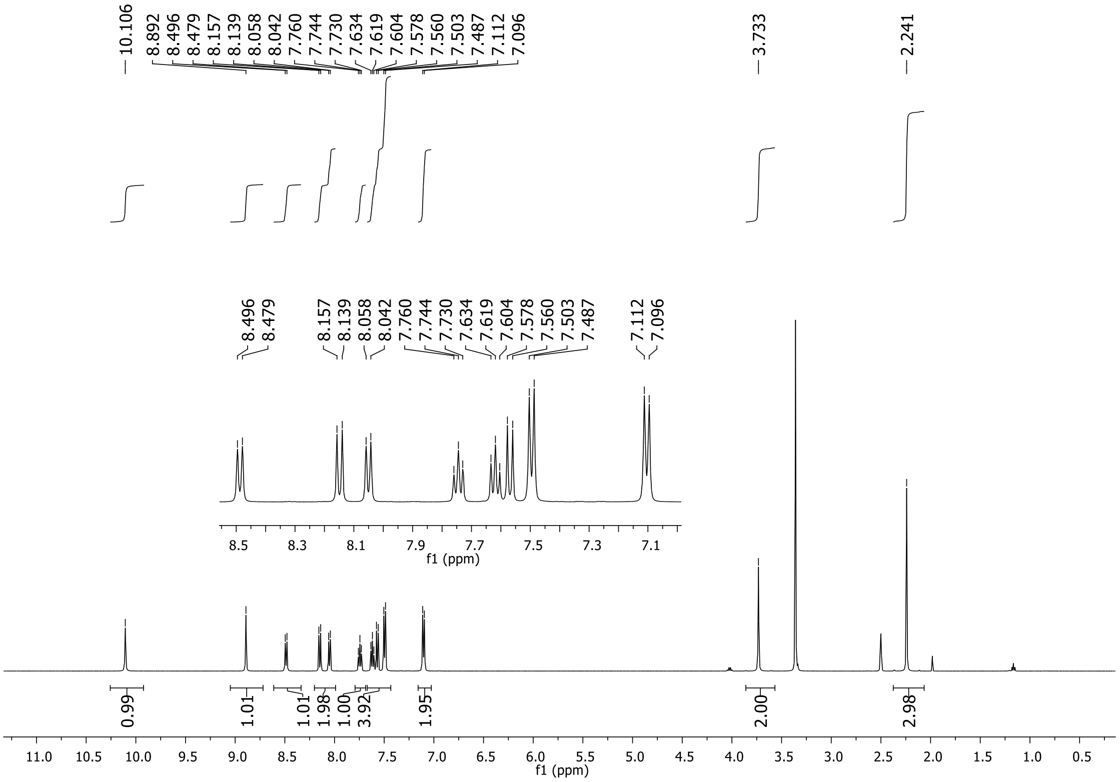

Figure S53. ^{1}H NMR (DMSO-d₆, 500 MHz) of **4m**

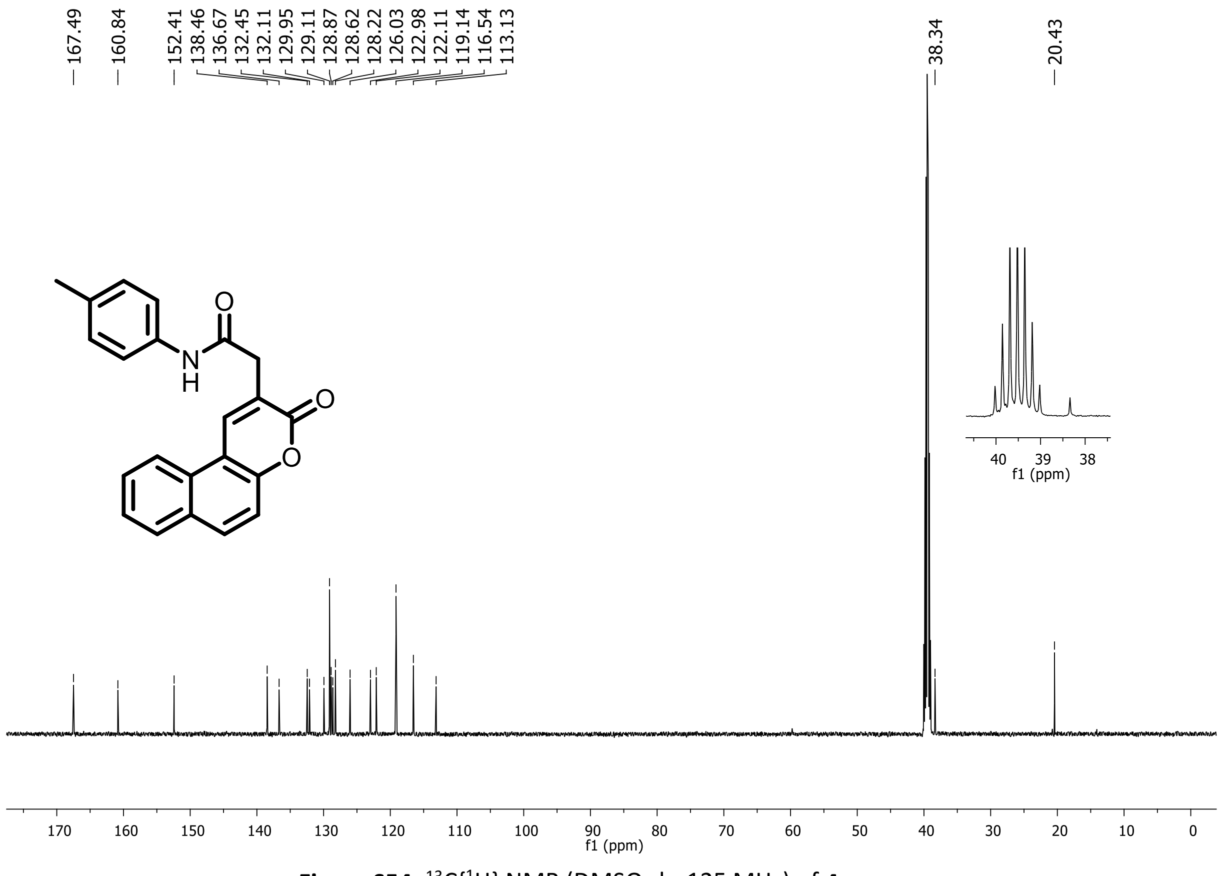

Figure S54. $^{13}C\{^{1}H\}$ NMR (DMSO-d₆, 125 MHz) of **4m**

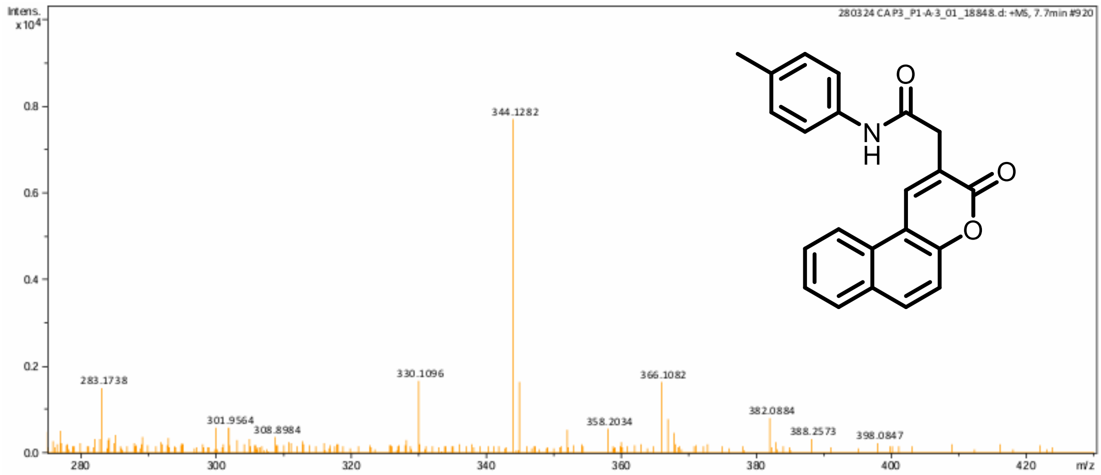

Figure S55. HRMS of **4m**

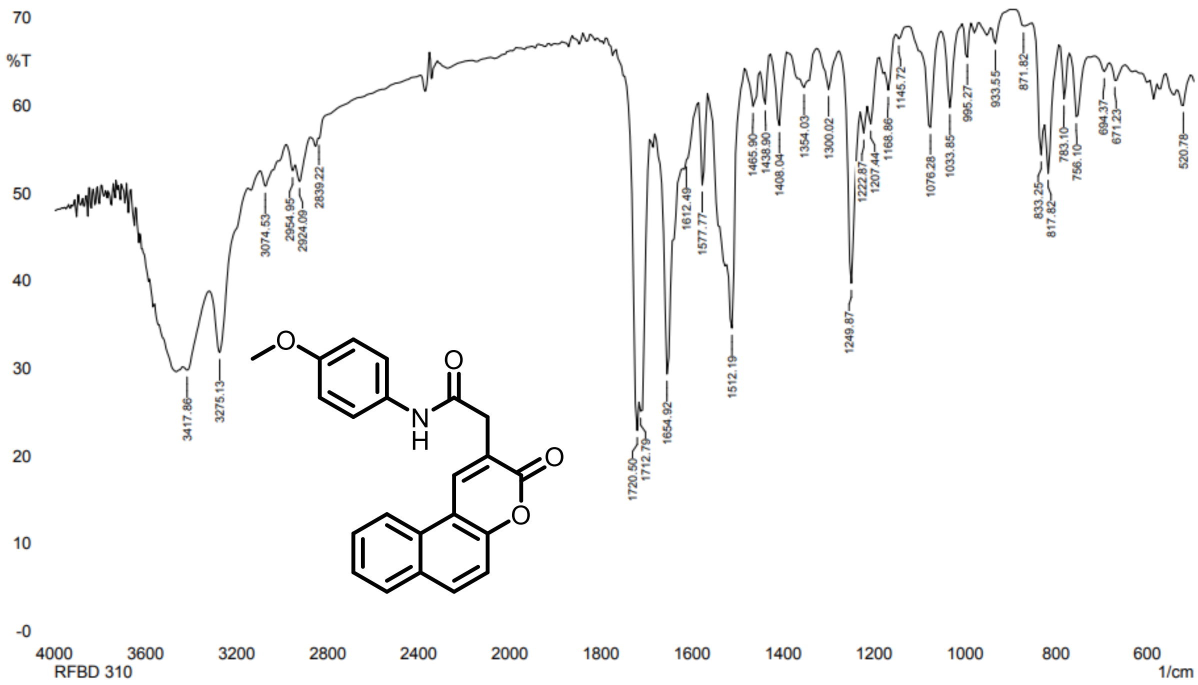

Comment;
RFBD 310

No. of Scans; 20
Resolution; 8 [1/cm]
Apodization; Happ-Genzel

Date/Time; 15/12/2022 17:41:27
User; FTIR

Figure S56. IR (KBr) of **4n**

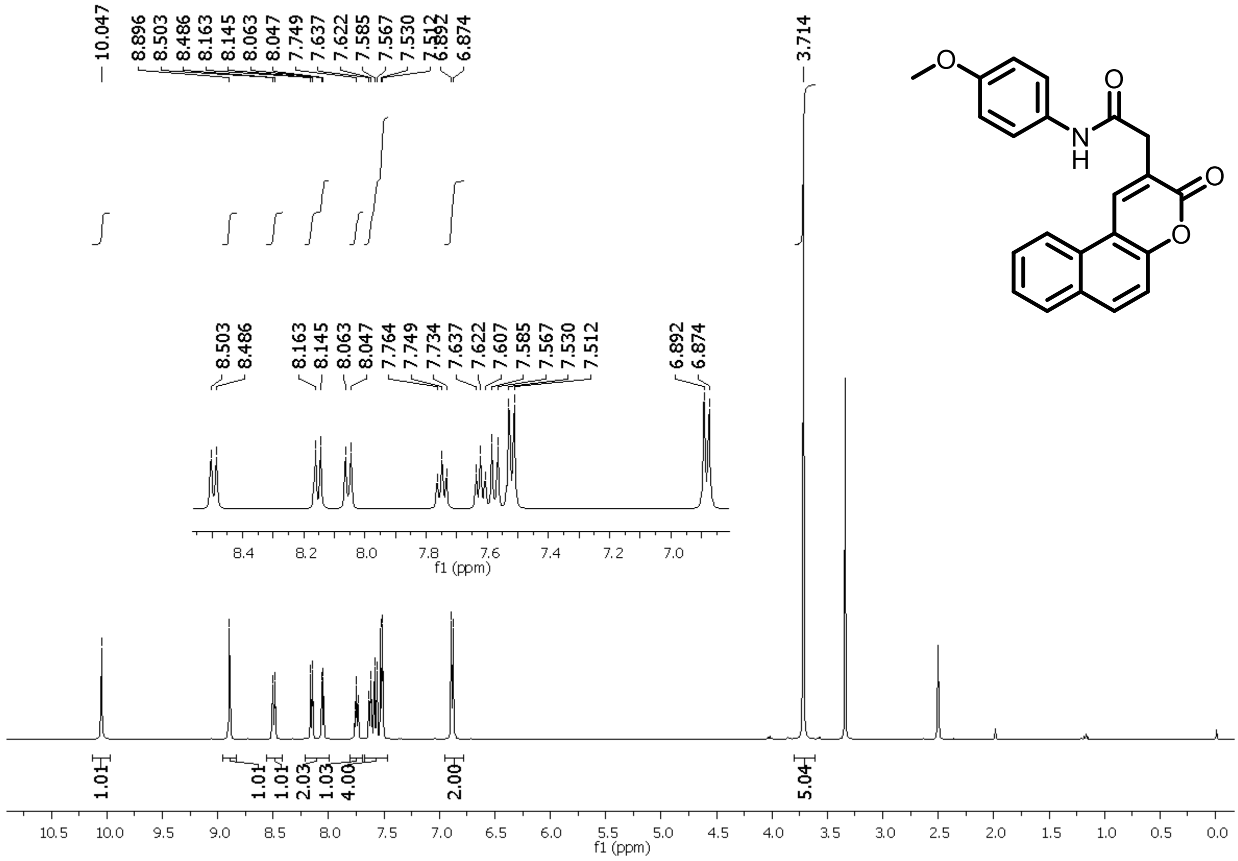

Figure S57. ^{1}H NMR (DMSO-d$_6$, 500 MHz) of **4n**

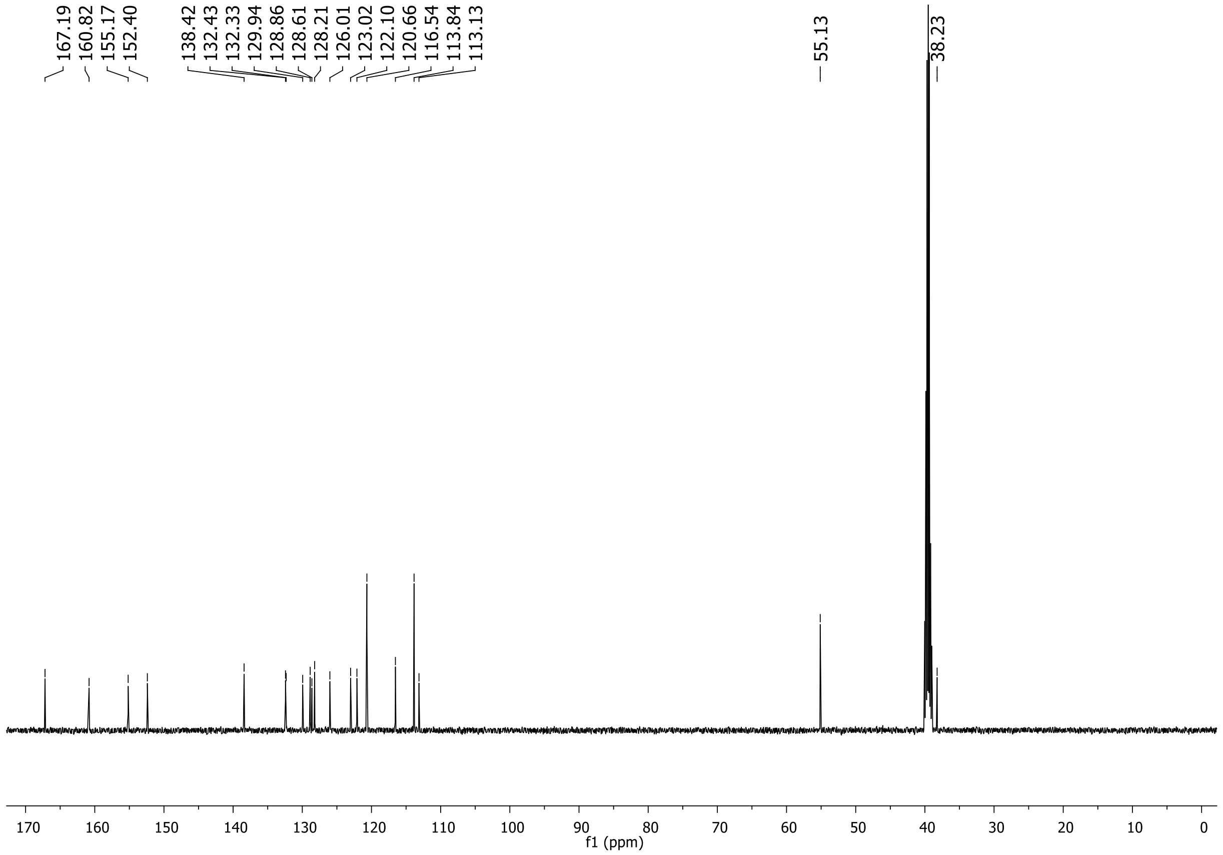

Figure S58. $^{13}C\{^{1}H\}$ NMR (DMSO-d$_6$, 125 MHz) of **4n**

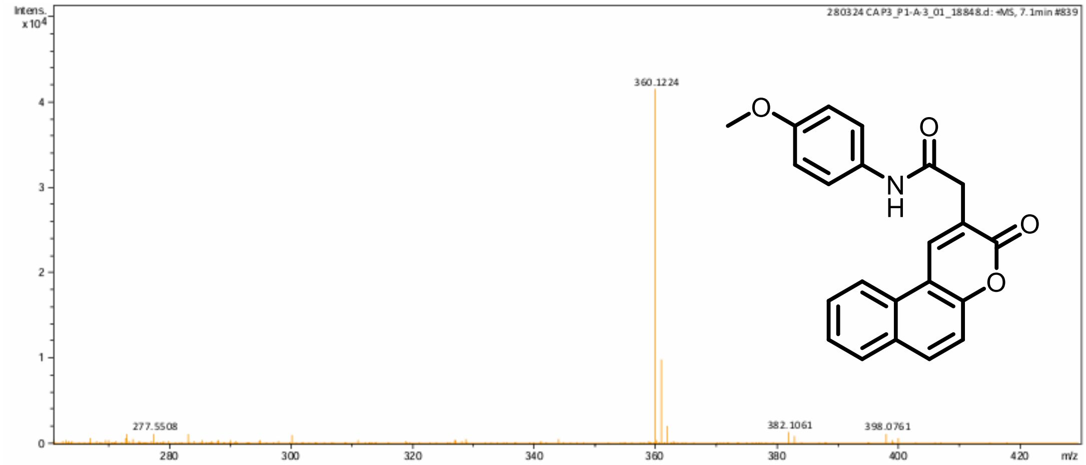

Figure S59. HRMS of **4n**

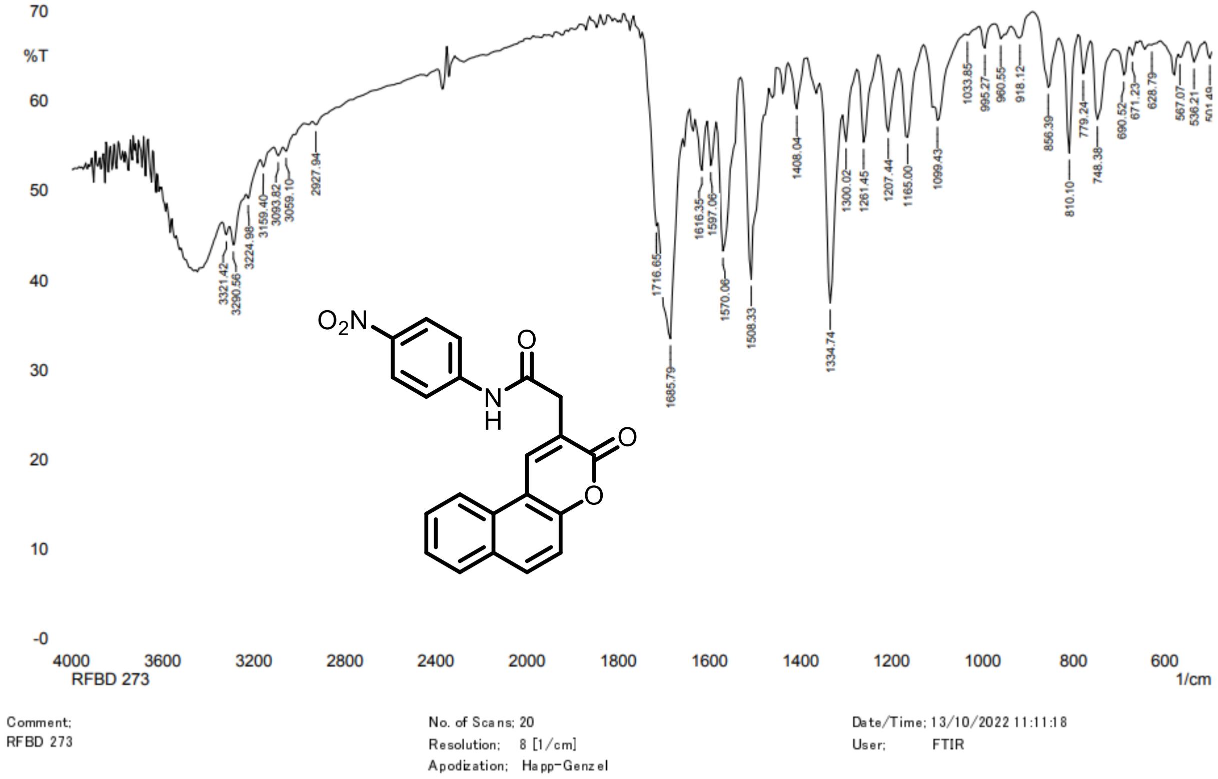

Figure S60. IR (KBr) of **4o**

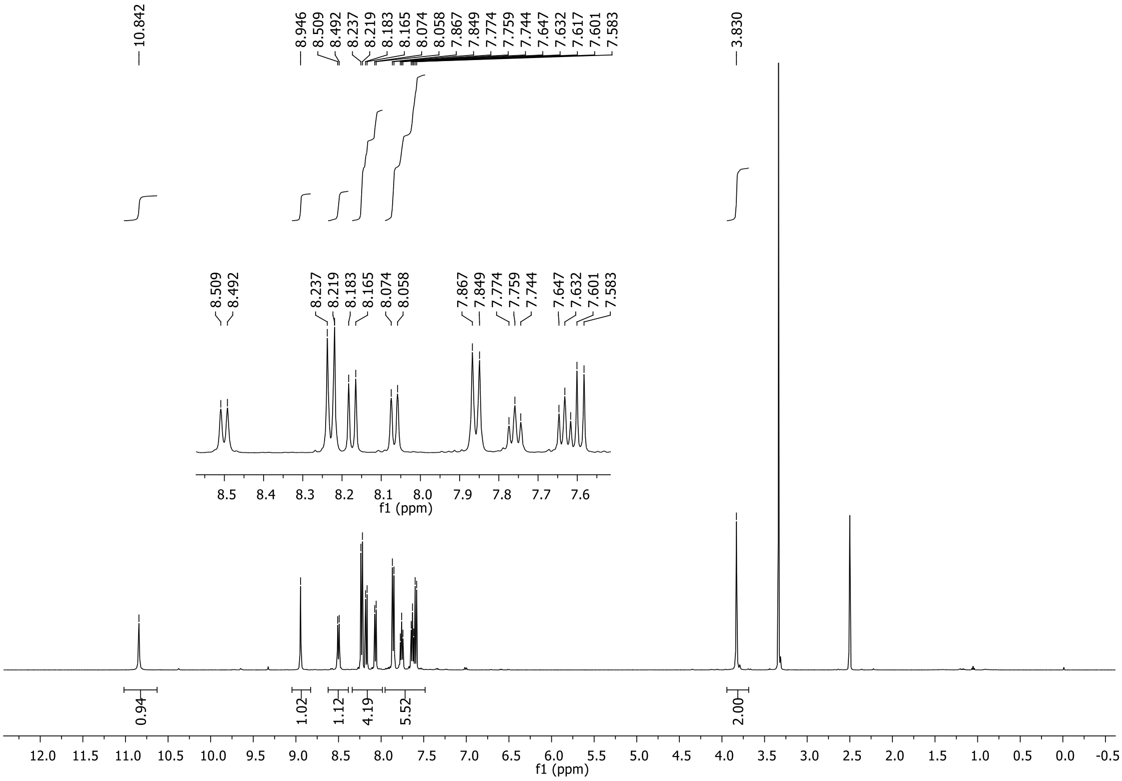

Figure S61. ^{1}H NMR (DMSO-d$_6$, 500 MHz) of **4o**

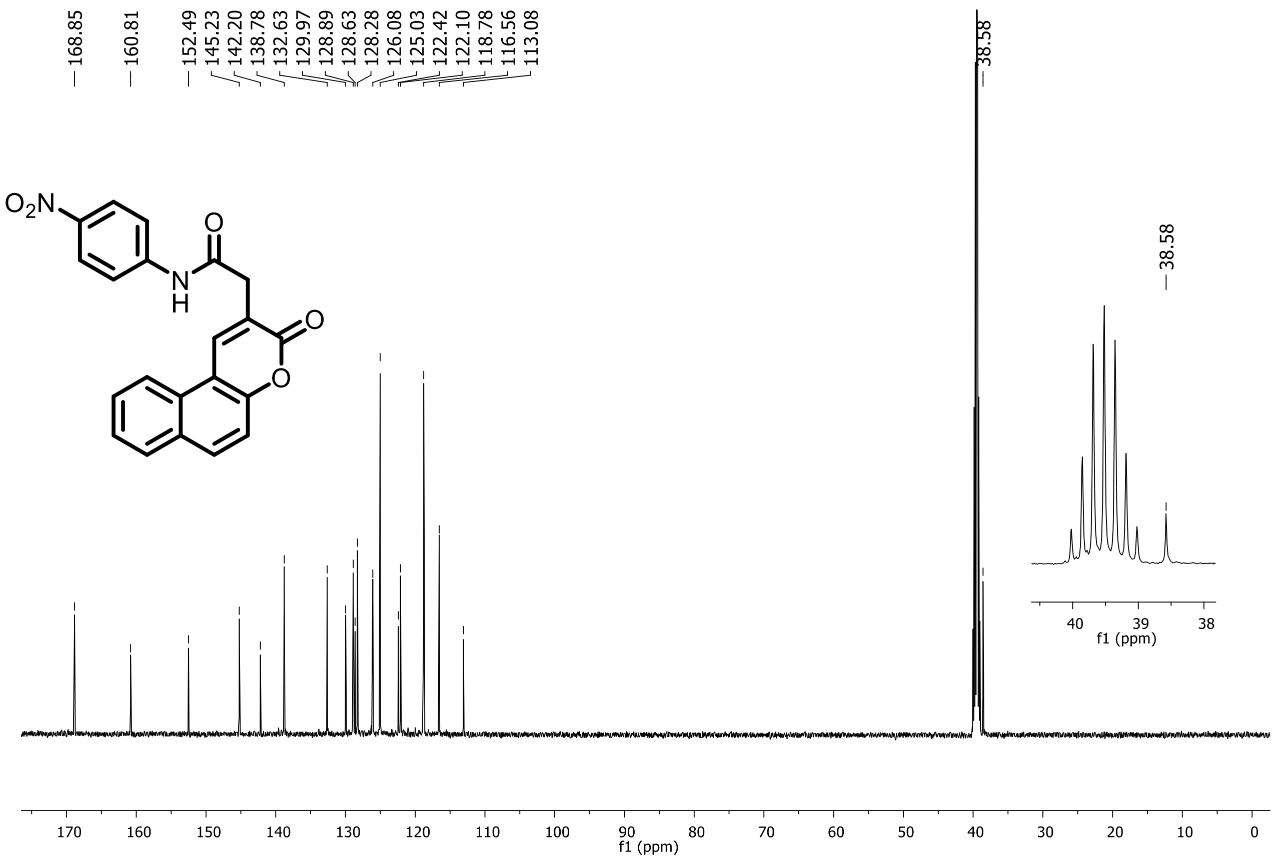

Figure S62. $^{13}C\{^{1}H\}$ NMR (DMSO-d$_6$, 125 MHz) of **4o**

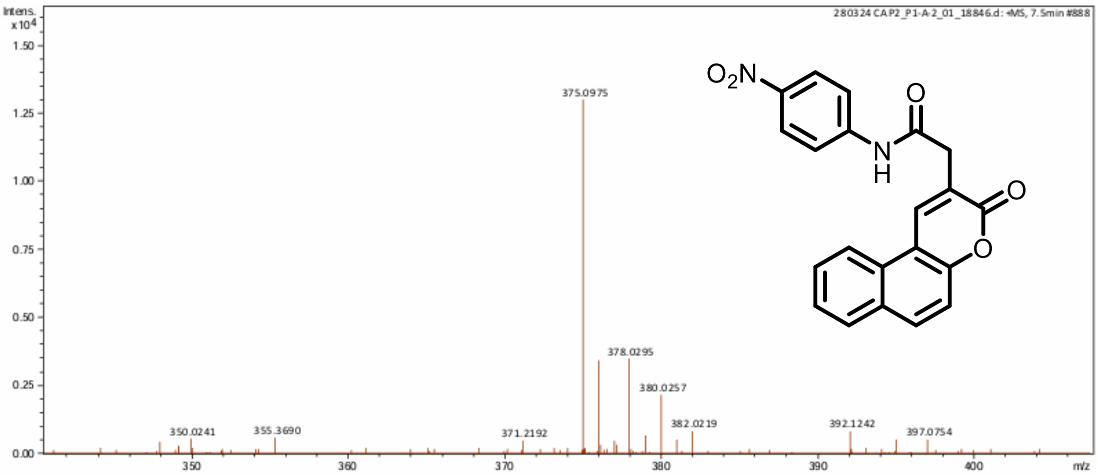

Figure S63. HRMS of **4o**

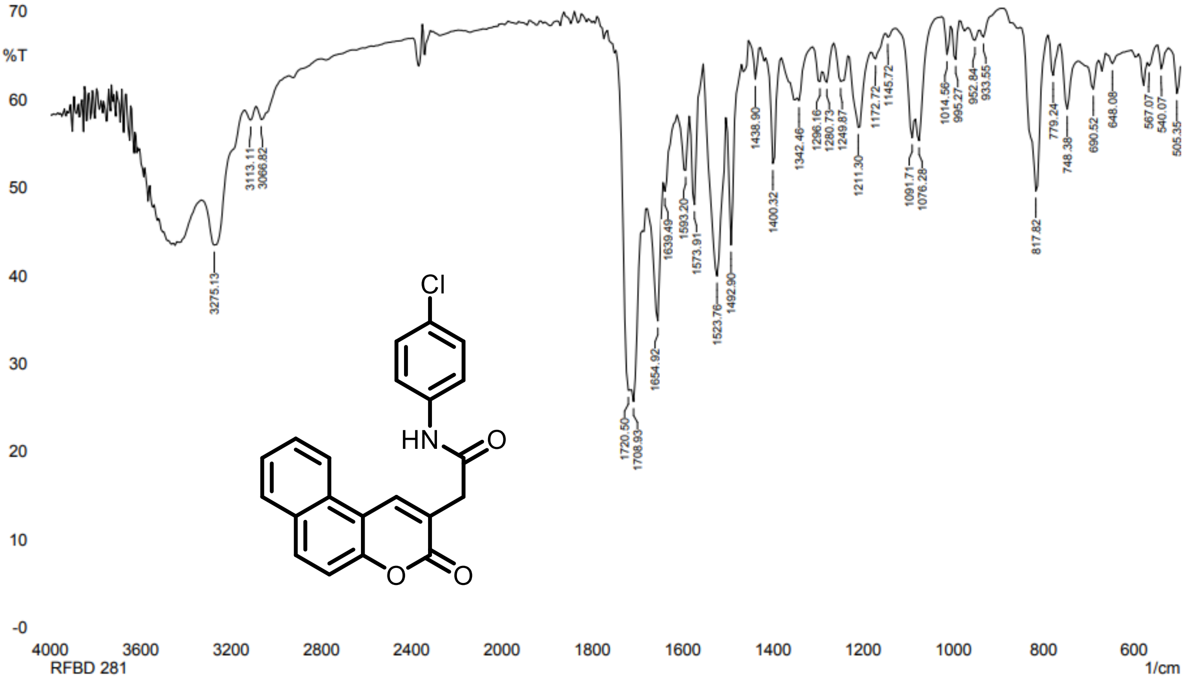

Comment;
RFBD 281

No. of Scans; 20
Resolution; 8 [1/cm]
Apodization; Happ-Genzel

Date/Time; 01/11/2022 11:21:31
User; FTIR

Figure S64. IR (KBr) of **4p**

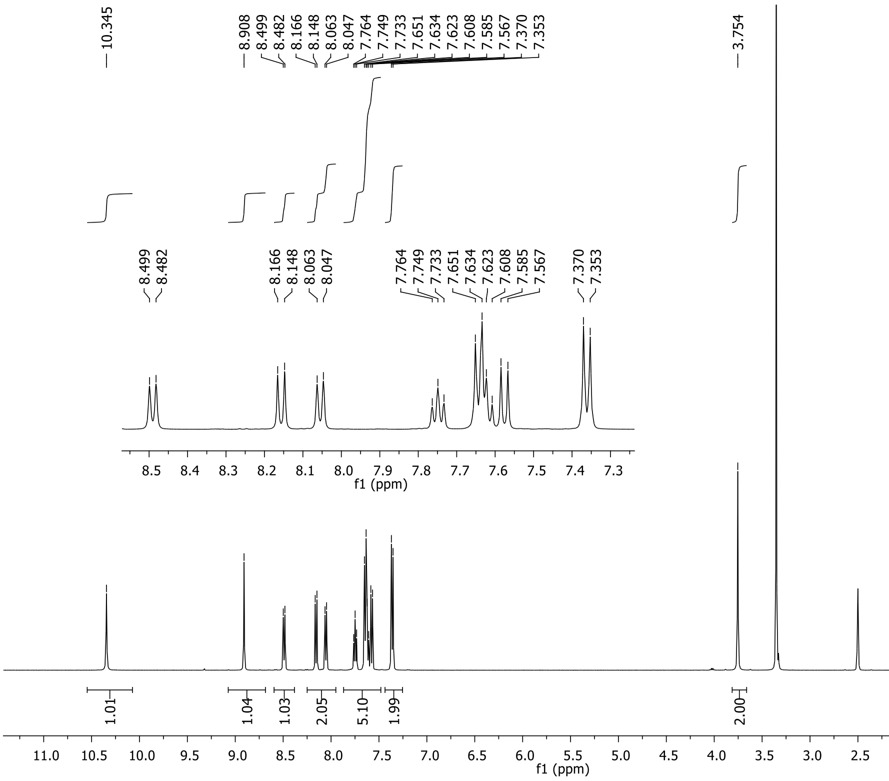

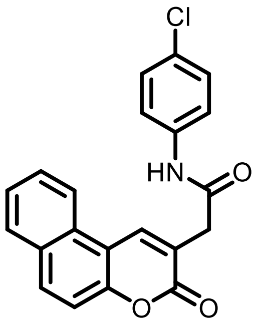

Figure S65. ^{1}H NMR (DMSO-d$_6$, 500 MHz) of **4p**

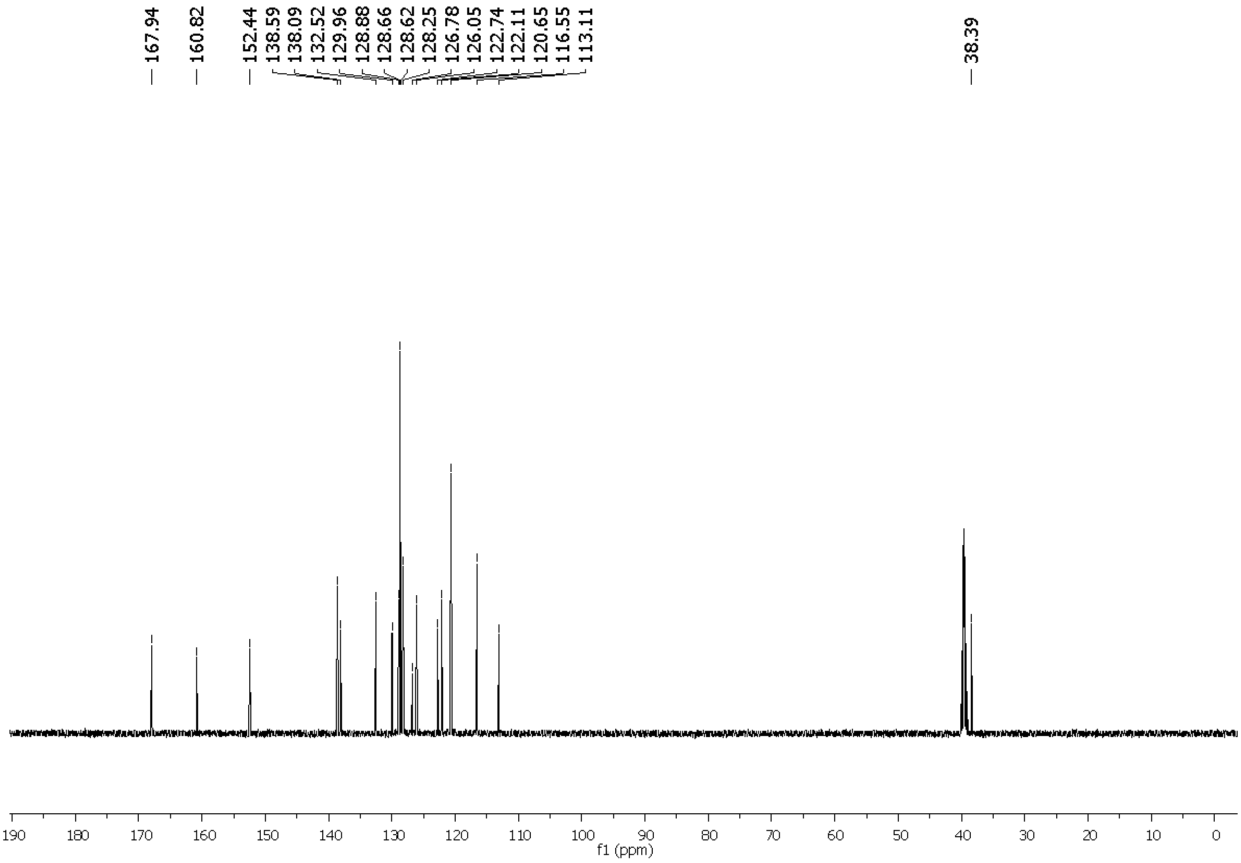

Figure S66. $^{13}C\{^{1}H\}$ NMR (DMSO-d$_6$, 125 MHz) of **4p**

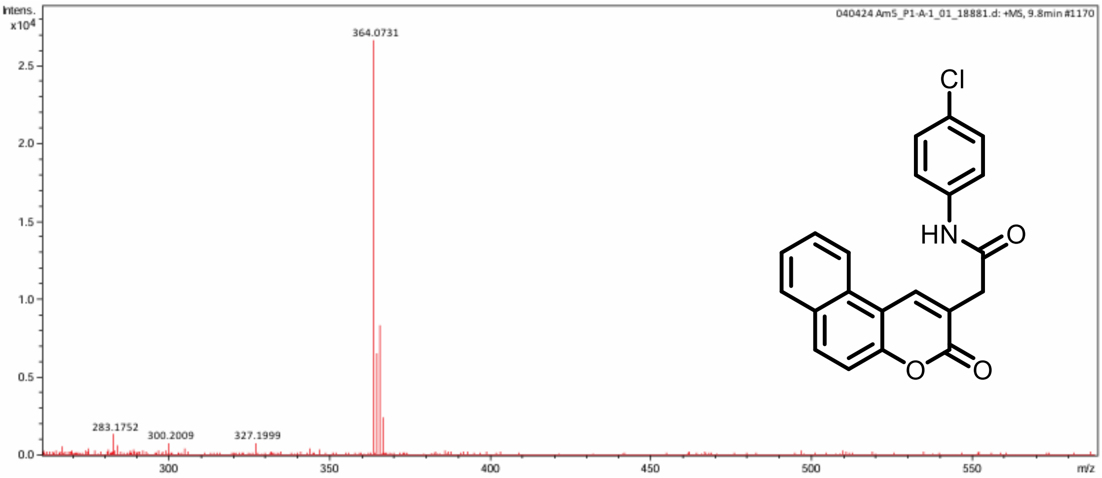

Figure S67. HRMS of **4p**

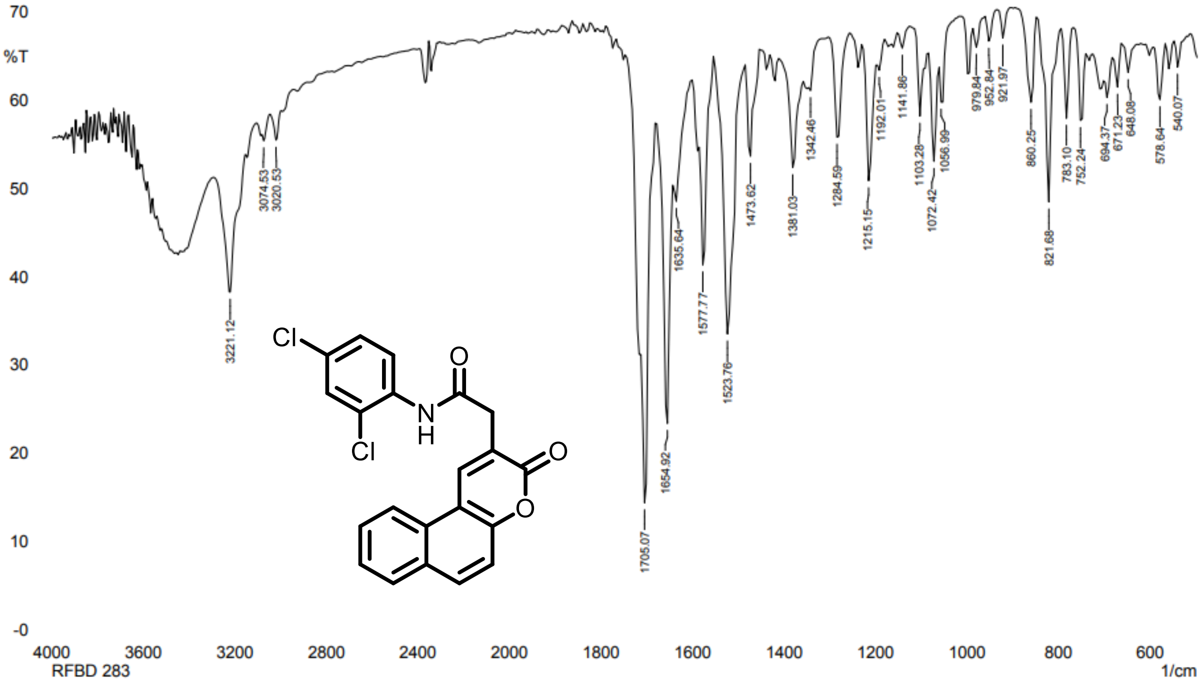

Comment;
RFBD 283

No. of Scans; 20
Resolution; 8 [1/cm]
Apodization; Happ-Genzel

Date/Time; 01/12/2022 15:53:40
User; FTIR

Figure S68. IR (KBr) of **4q**

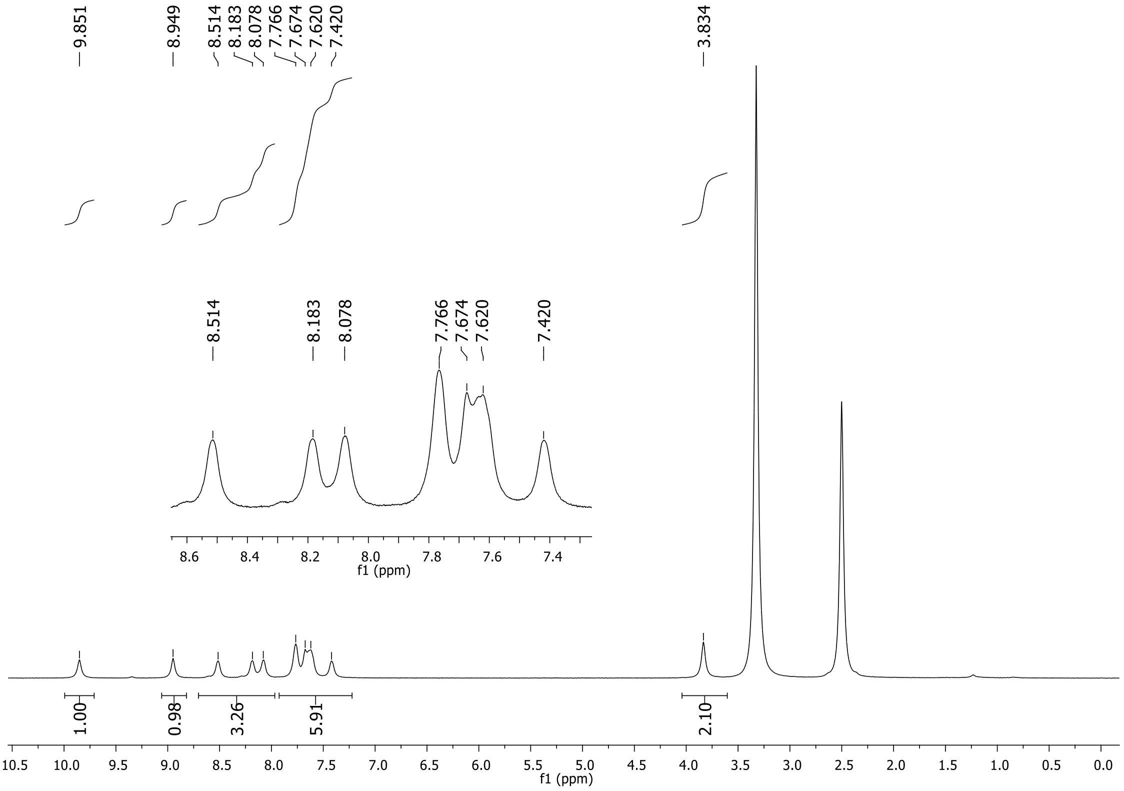

Figure S69. ^{1}H NMR (DMSO-d$_6$, 500 MHz) of **4q**

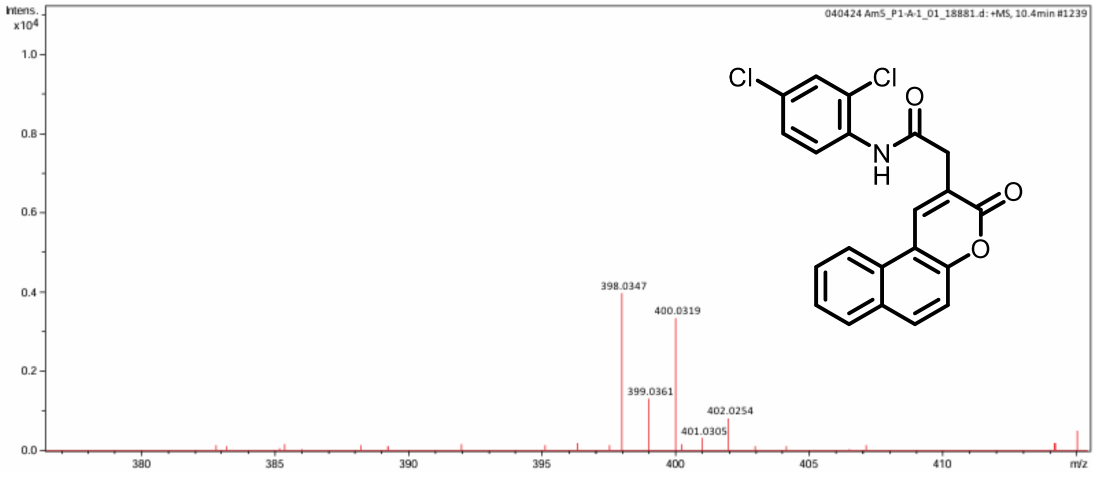

Figure S70. HRMS of **4q**

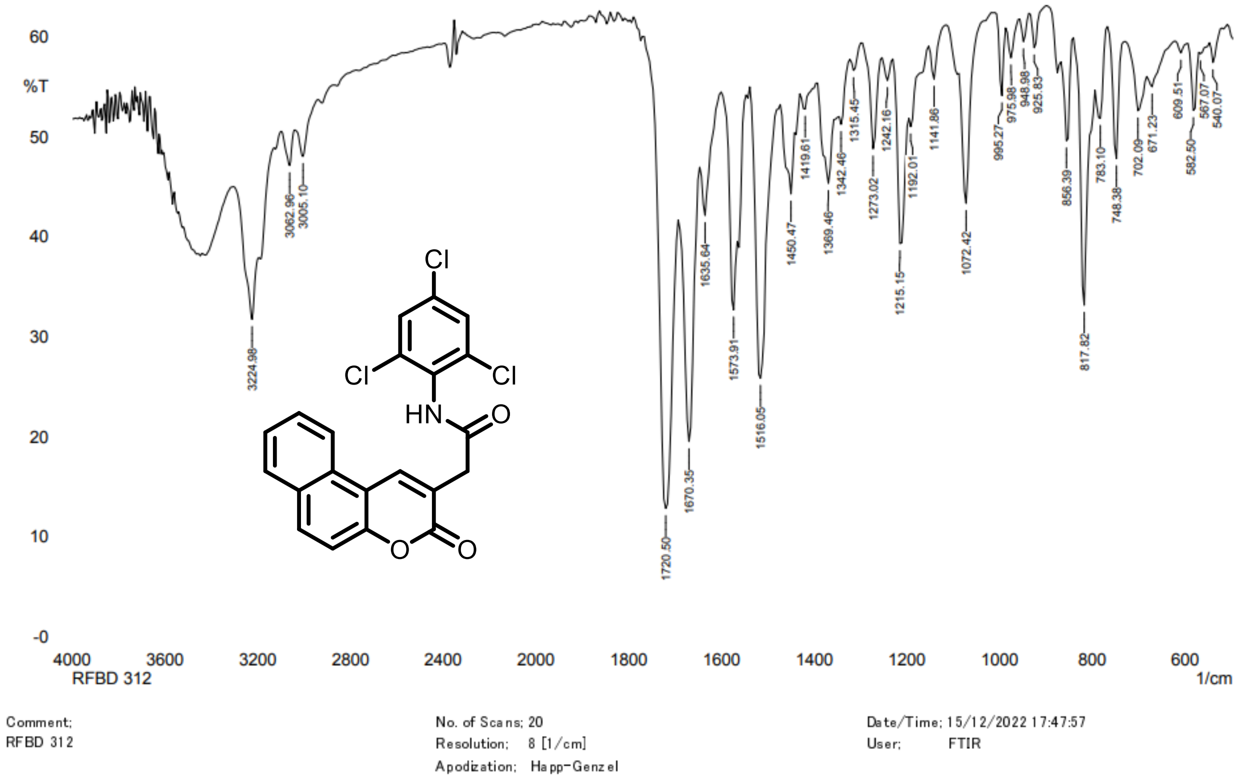

Figure S71. IR (KBr) of **4r**

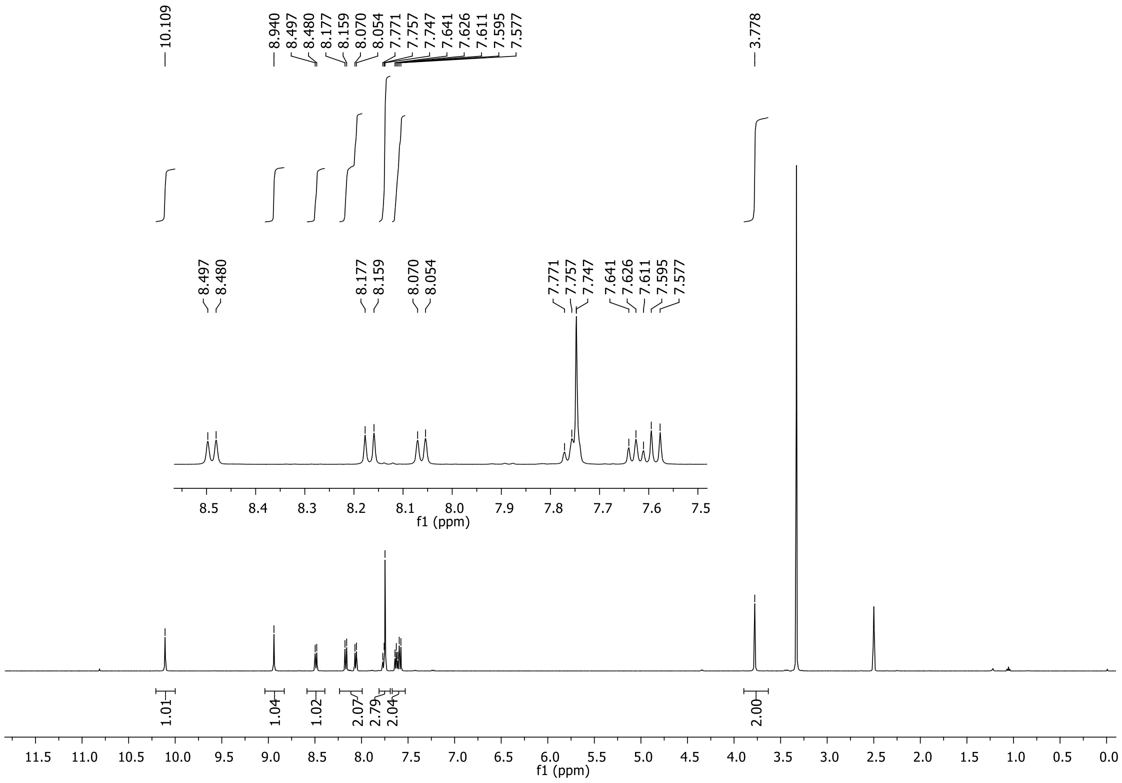

Figure S72. ^{1}H NMR (DMSO-d$_6$, 500 MHz) of **4r**

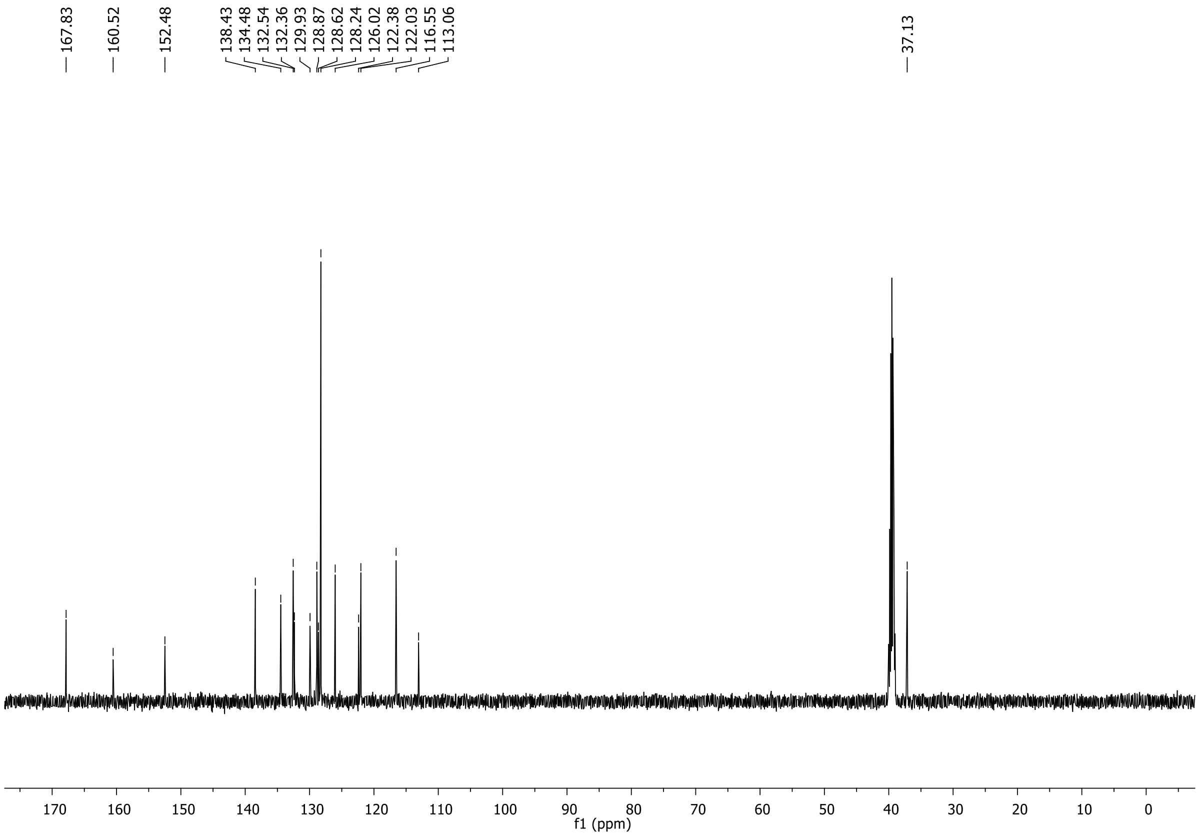

Figure S73. $^{13}C\{^{1}H\}$ NMR (DMSO-d$_6$, 125 MHz) of **4r**

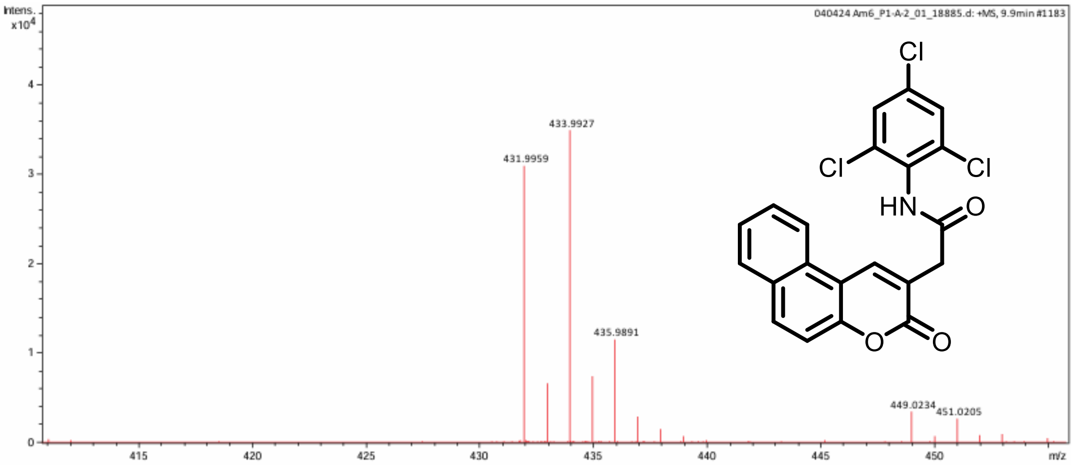

Figure S74. HRMS of **4r**

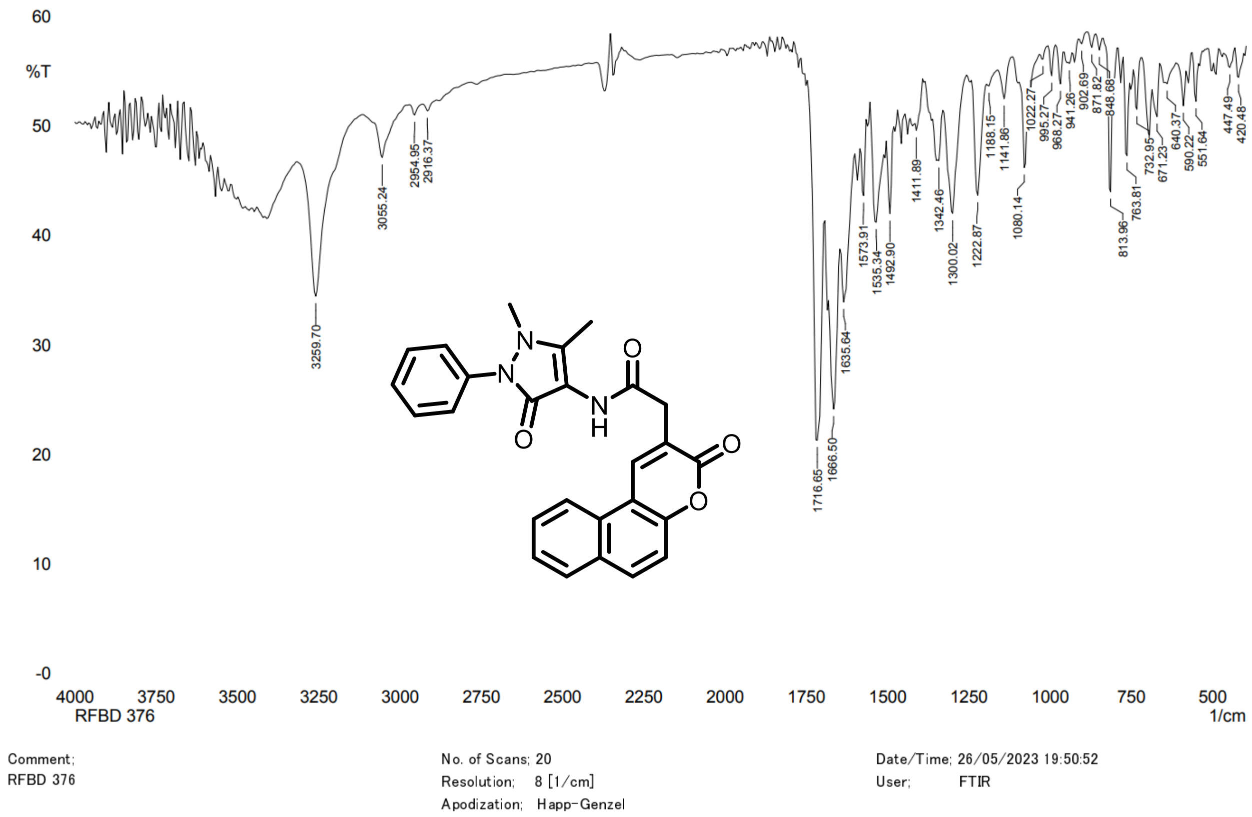

Figure S75. IR (KBr) of **4s**

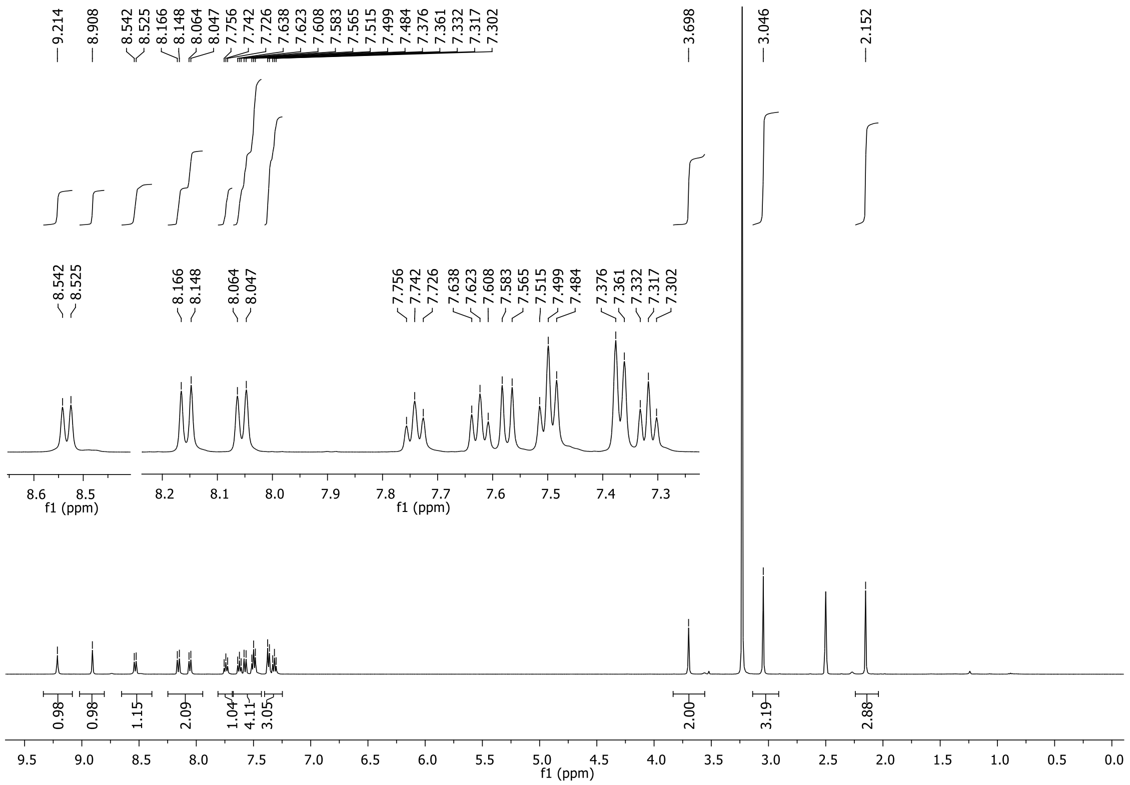

Figure S76. ^{1}H NMR (DMSO-d_6, 500 MHz) of **4s**

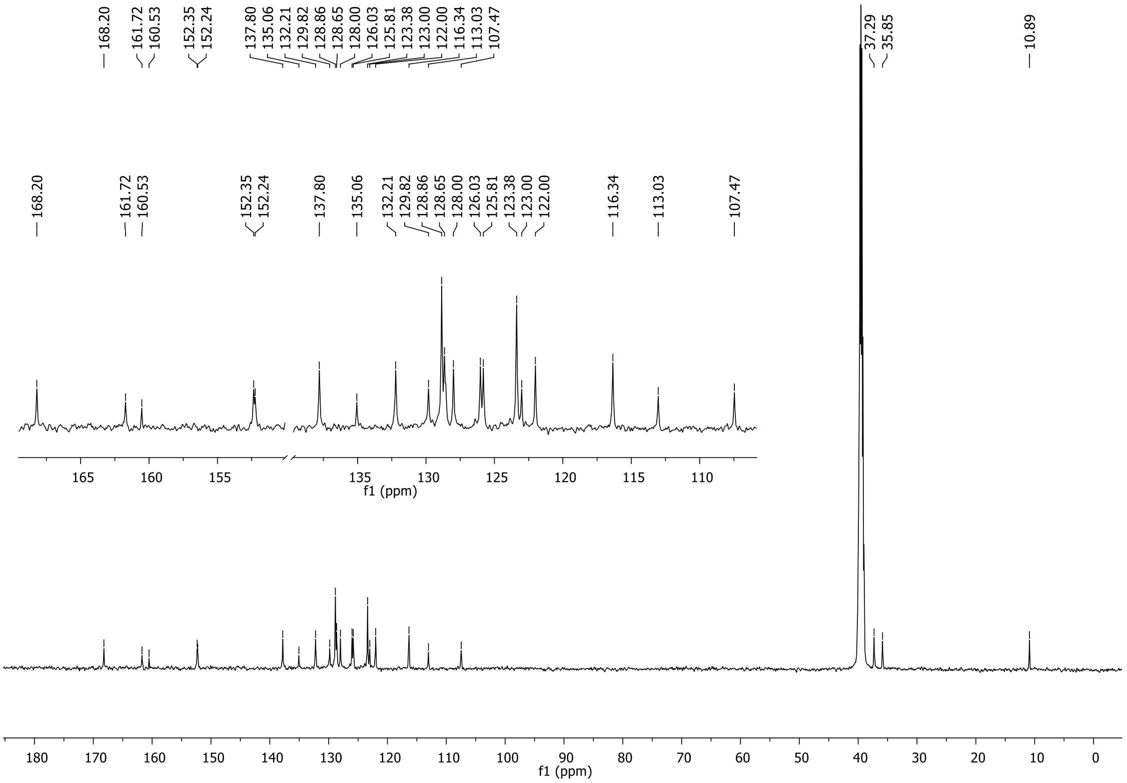

Figure S77. $^{13}C\{^{1}H\}$ NMR (DMSO-d$_6$, 125 MHz) of **4s**

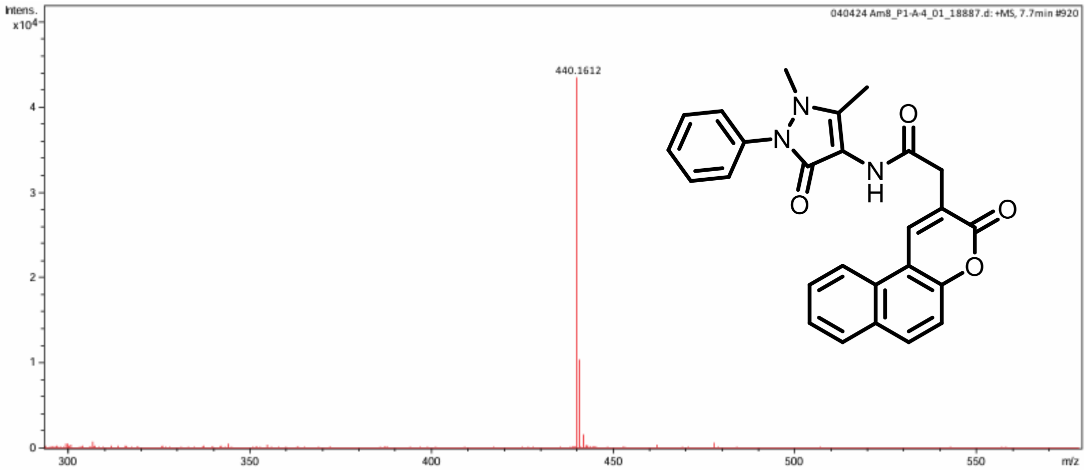

Figure S78. HRMS of **4s**

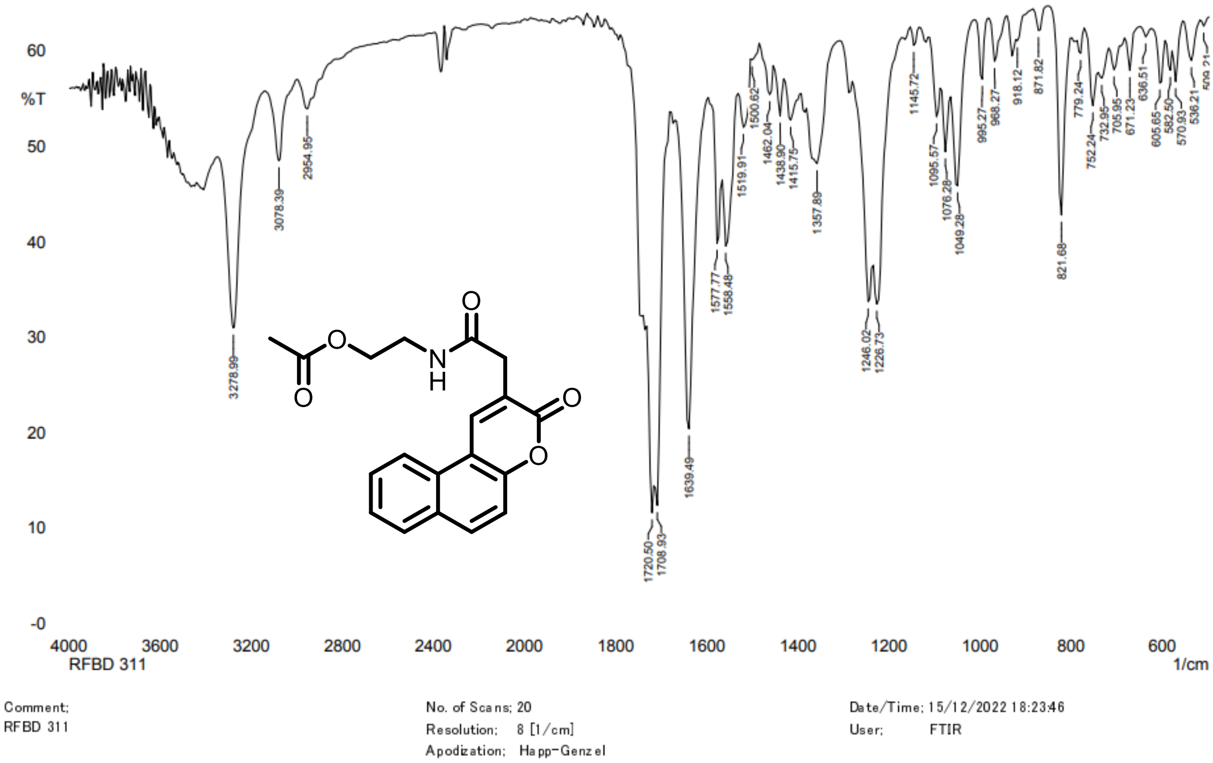

Figure S79. IR (KBr) of **4t**

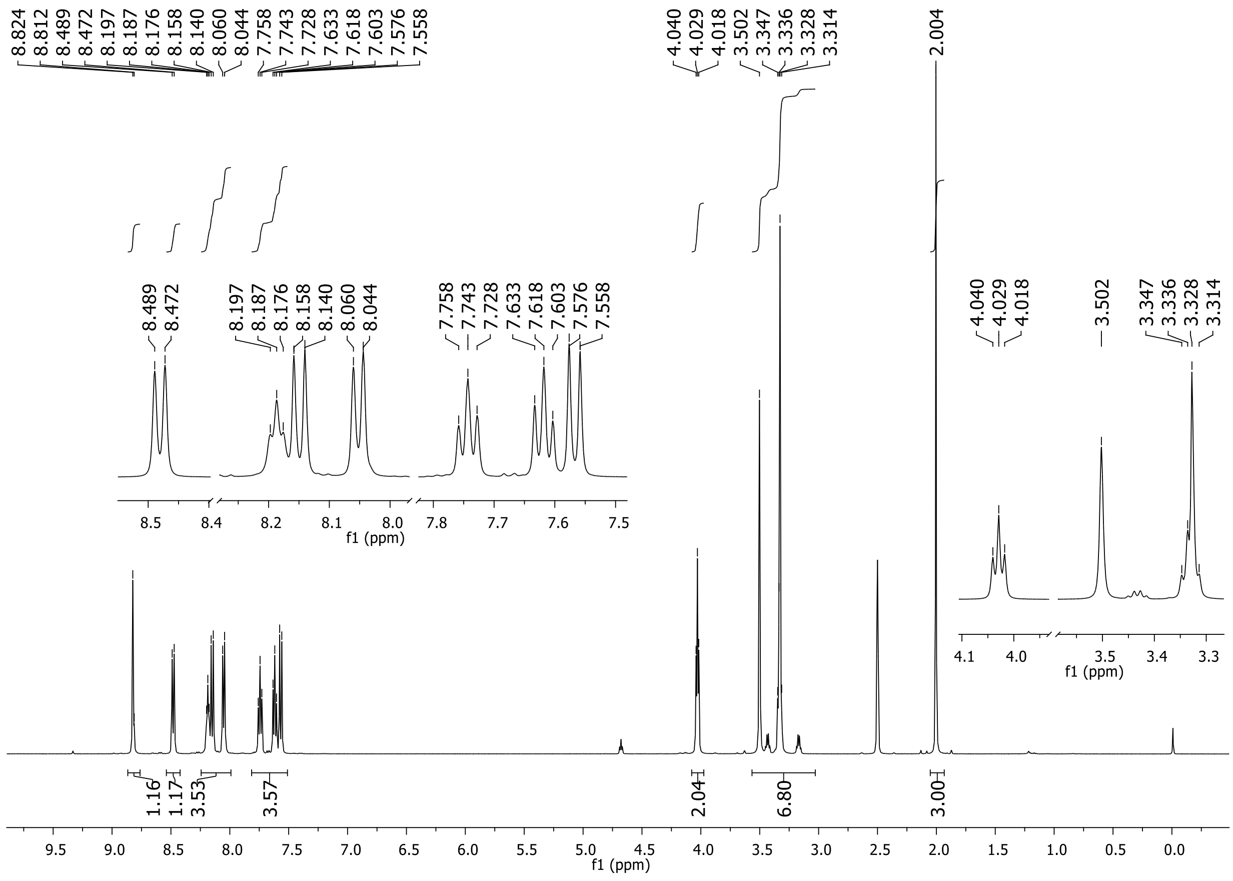

Figure S80. ^{1}H NMR (DMSO-d_6, 500 MHz) of **4t**

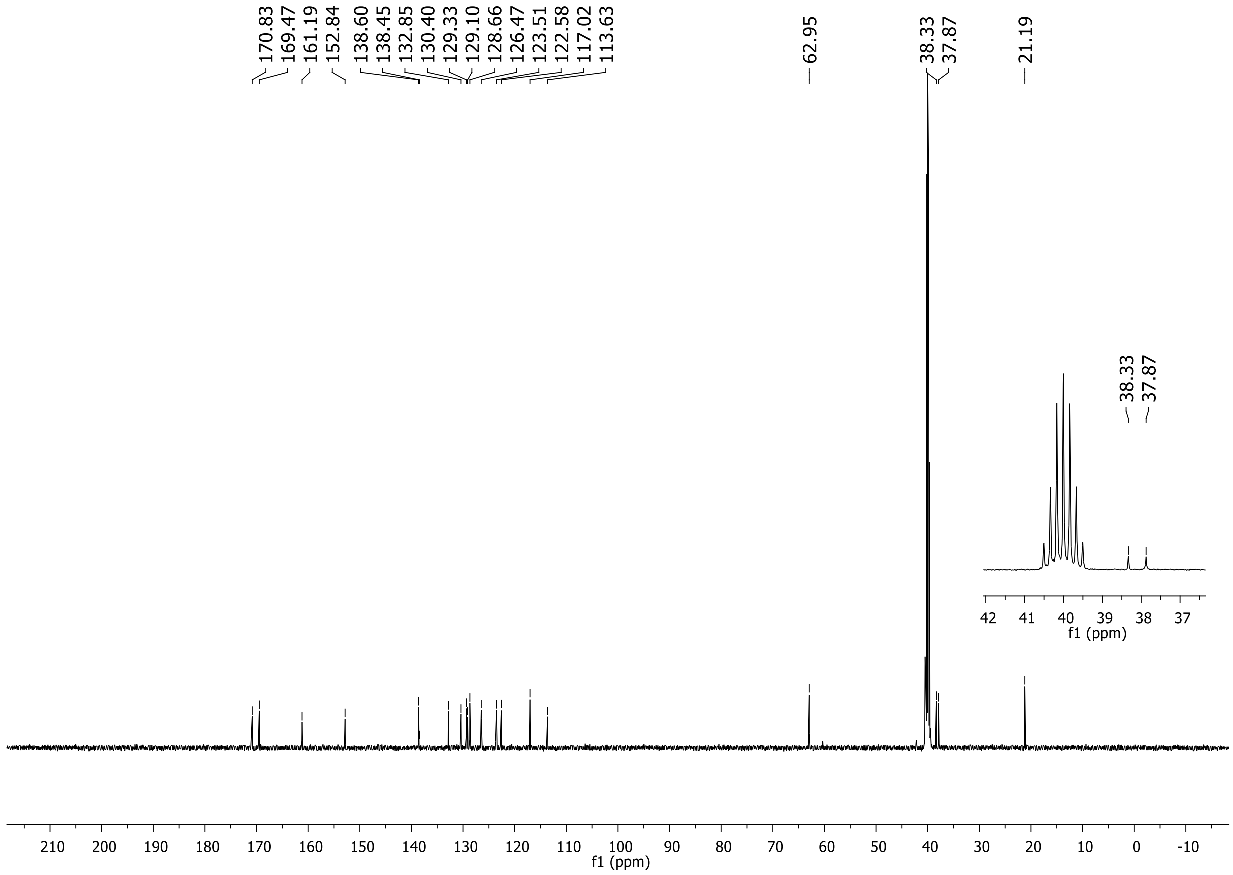

Figure S81. $^{13}C\{^{1}H\}$ NMR (DMSO-d$_6$, 125 MHz) of **4t**

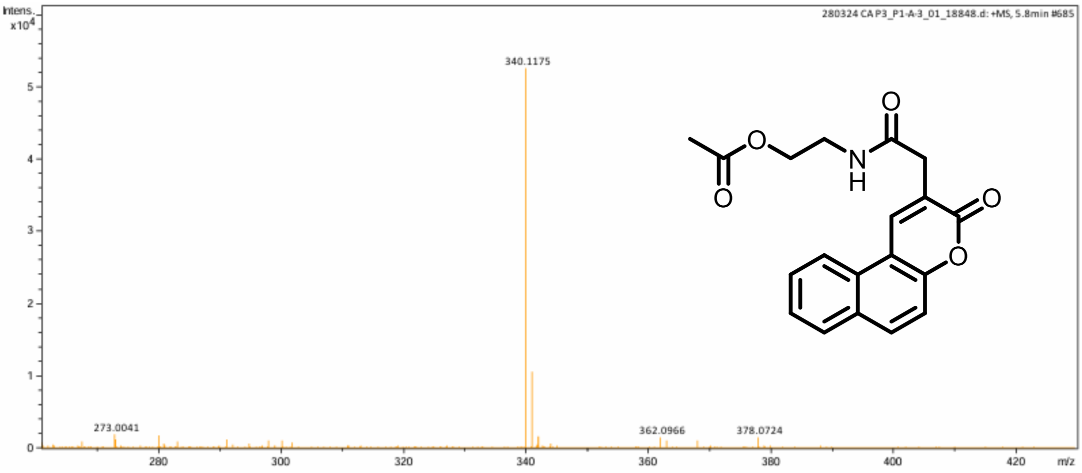

Figure S82. HRMS of **4t**

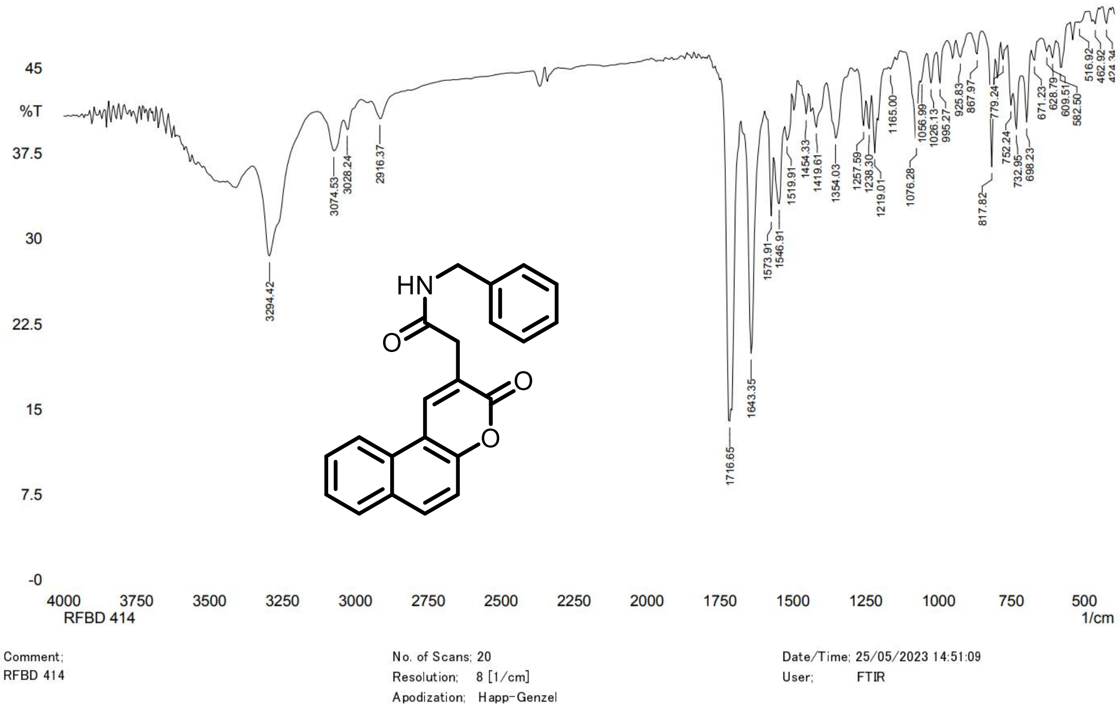

Figure S83. IR (KBr) of **4u**

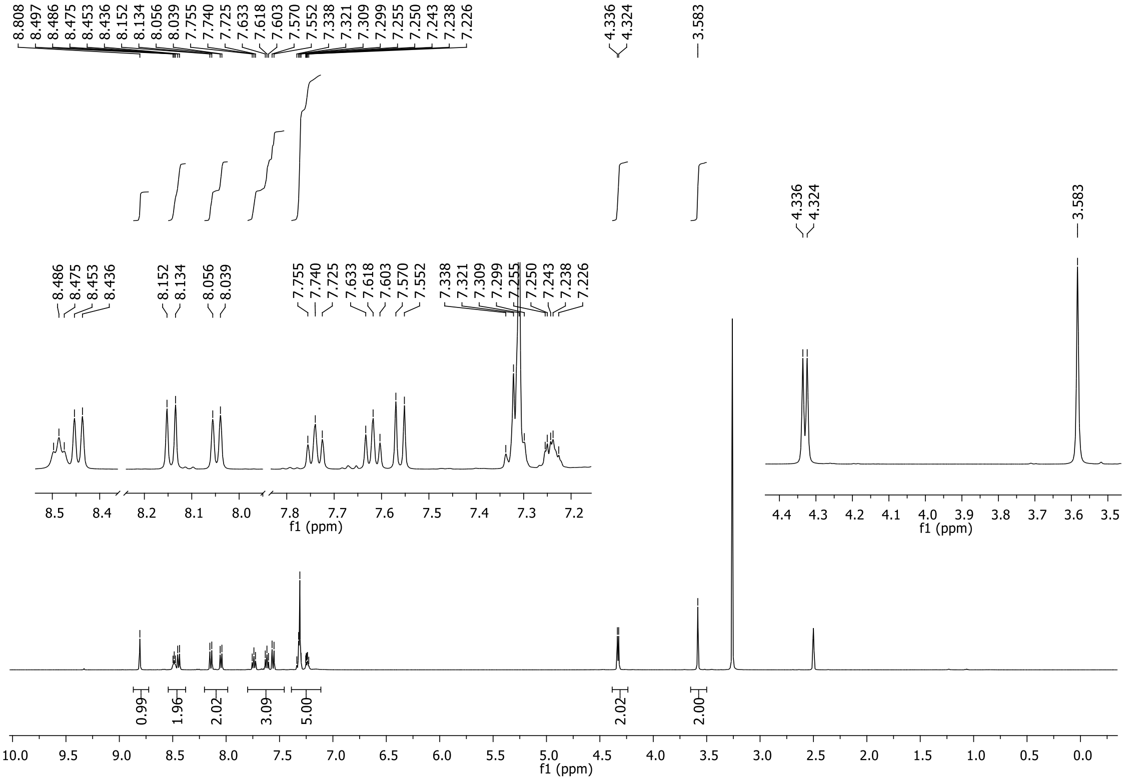

Figure S84. ^{1}H NMR (DMSO-d$_6$, 500 MHz) of **4u**

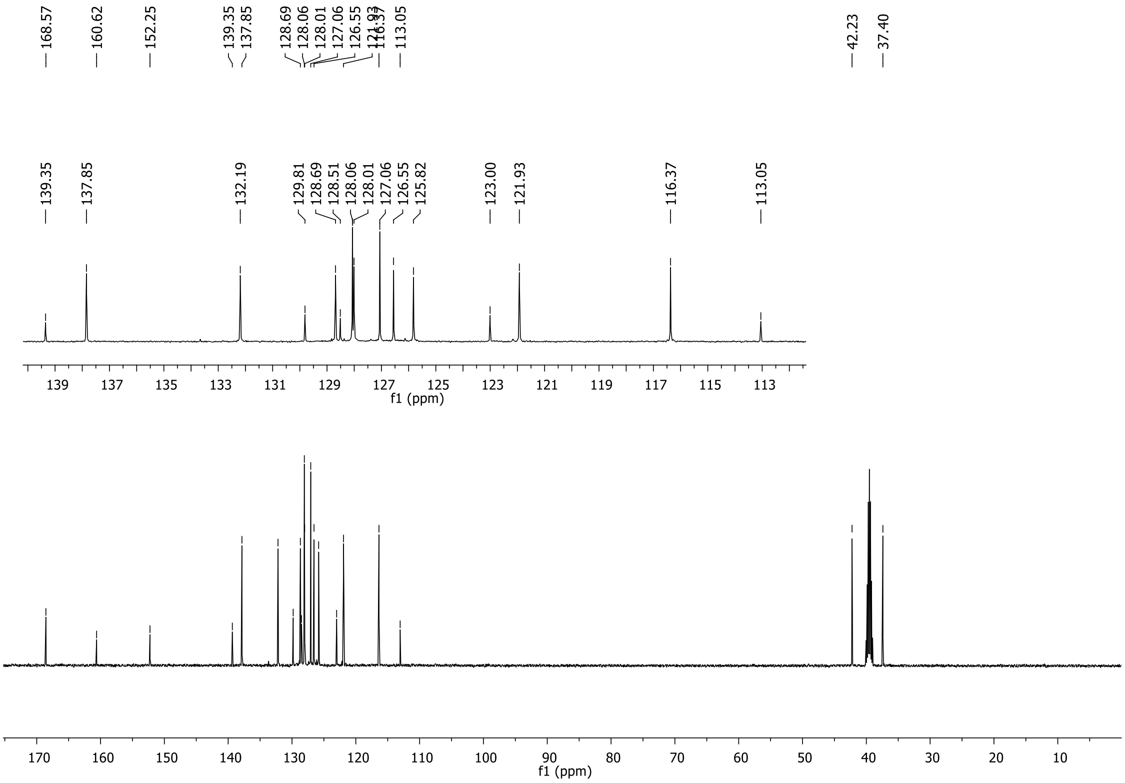

Figure S85. $^{13}C\{^{1}H\}$ NMR (DMSO-d$_6$, 125 MHz) of **4u**

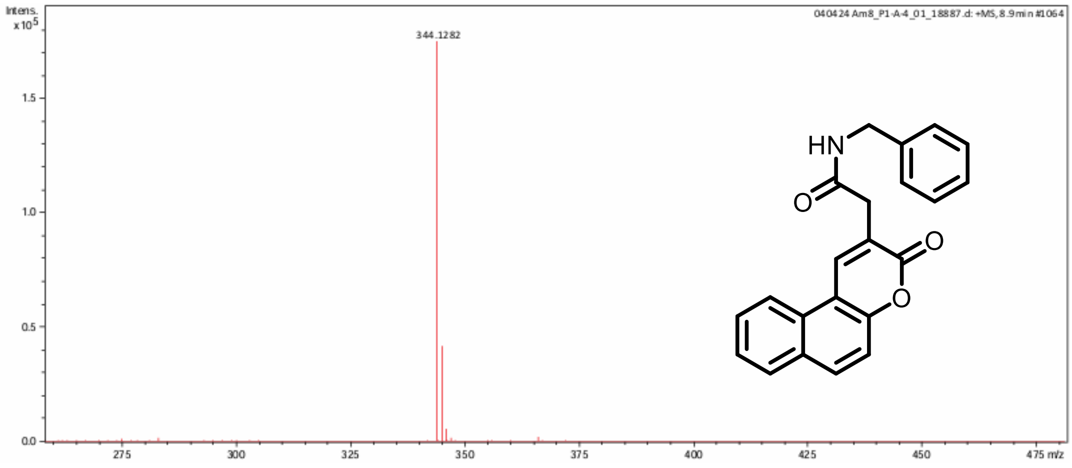

Figure S86. HRMS of **4u**

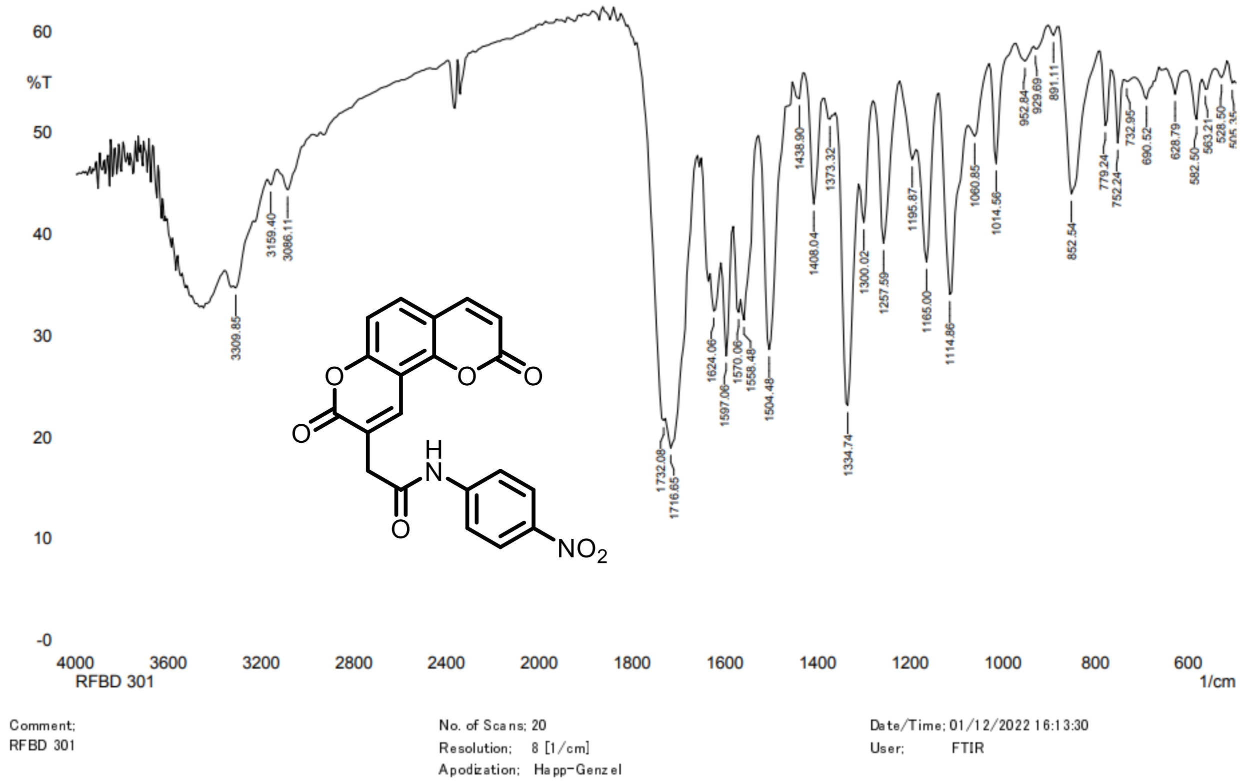

Figure S87. IR (KBr) of **4v**

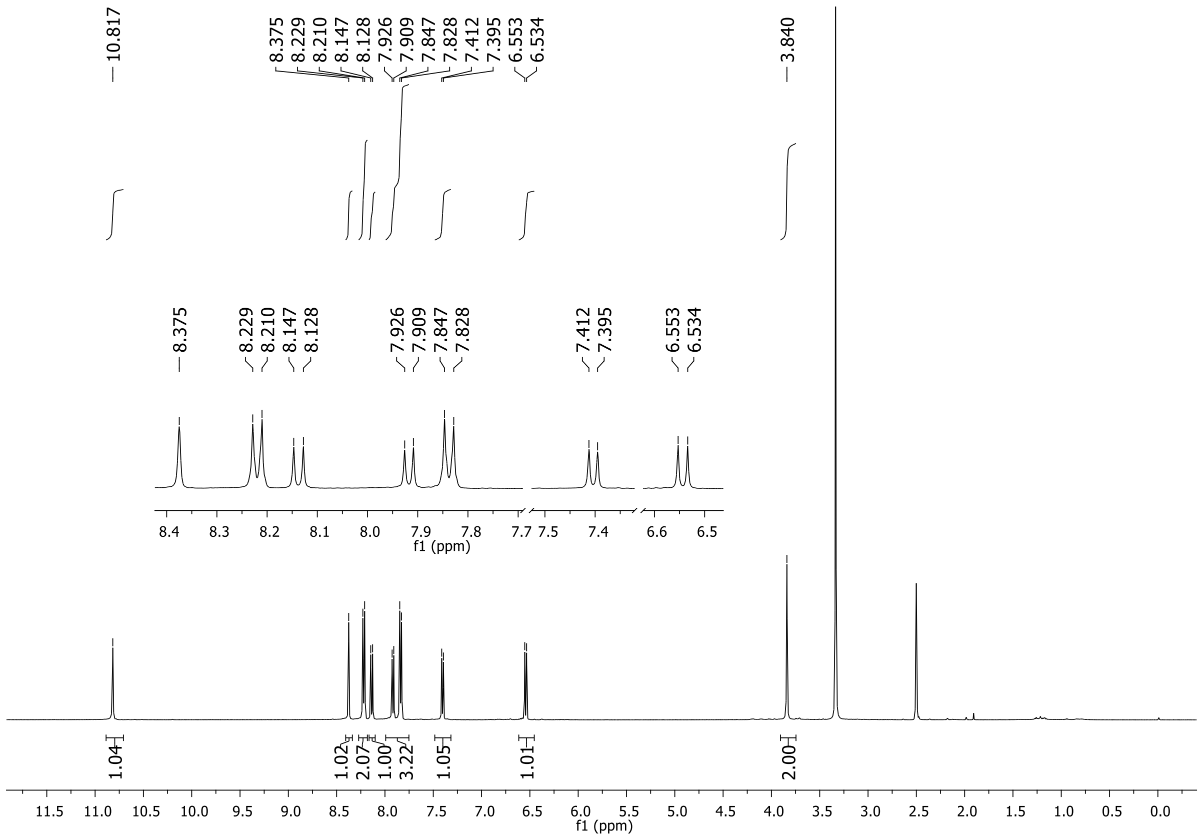

Figure S88. ^{1}H NMR (DMSO-d_6, 500 MHz) of **4v**

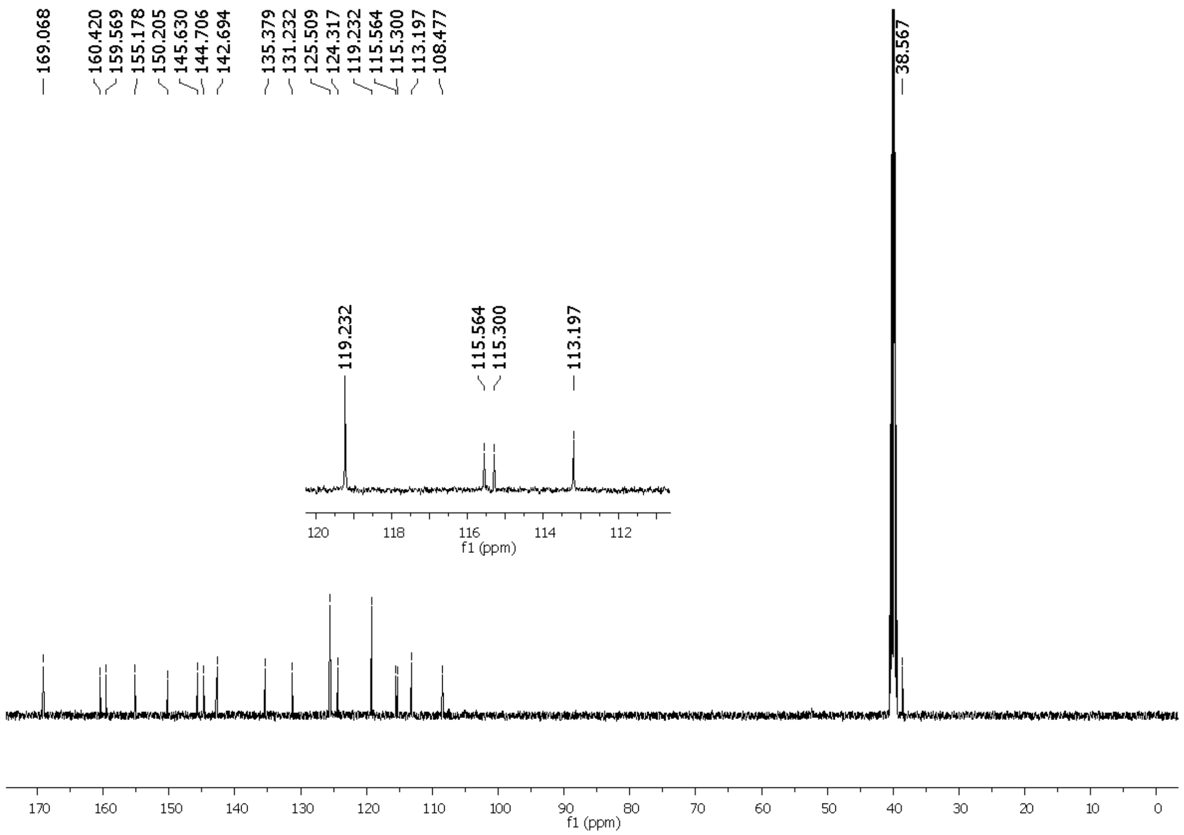

Figure S89. $^{13}C\{^{1}H\}$ NMR (DMSO-d₆, 125 MHz) of **4v**

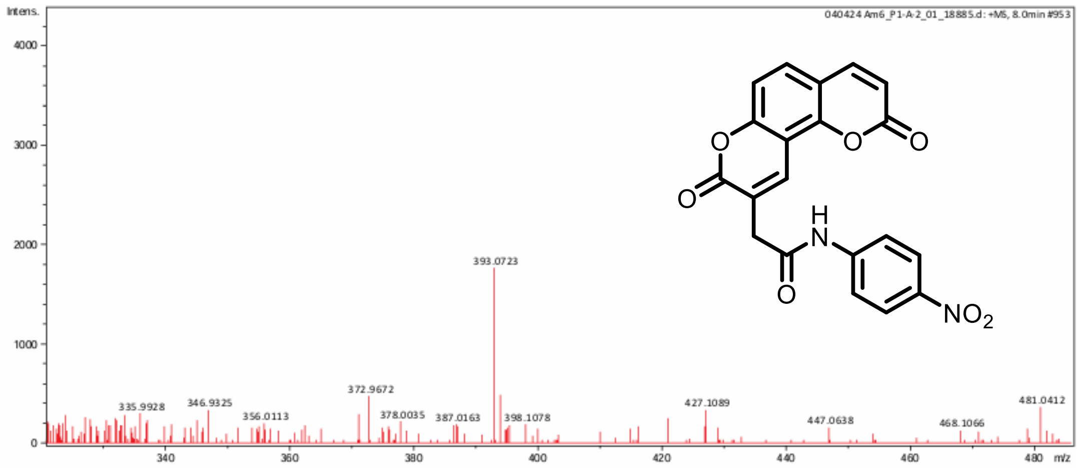

Figure S90. HRMS of **4v**

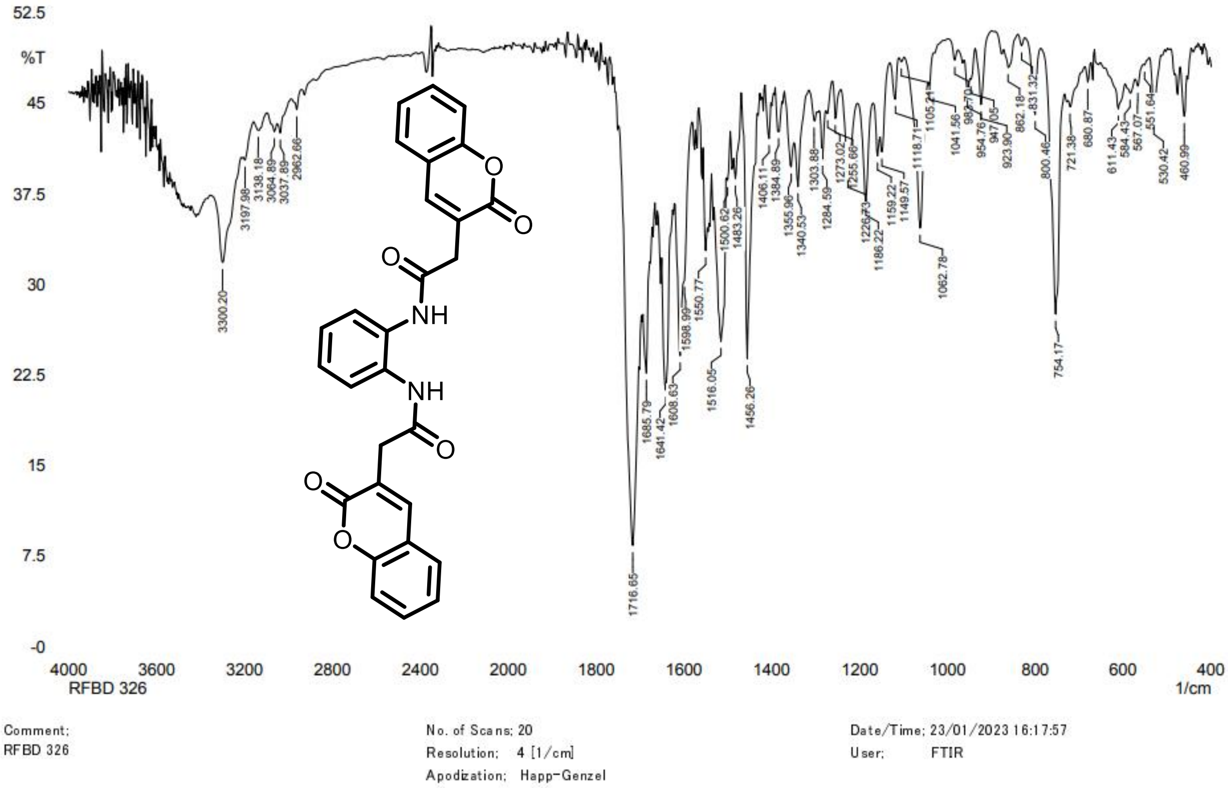

Figure S91. IR (KBr) of **4w**

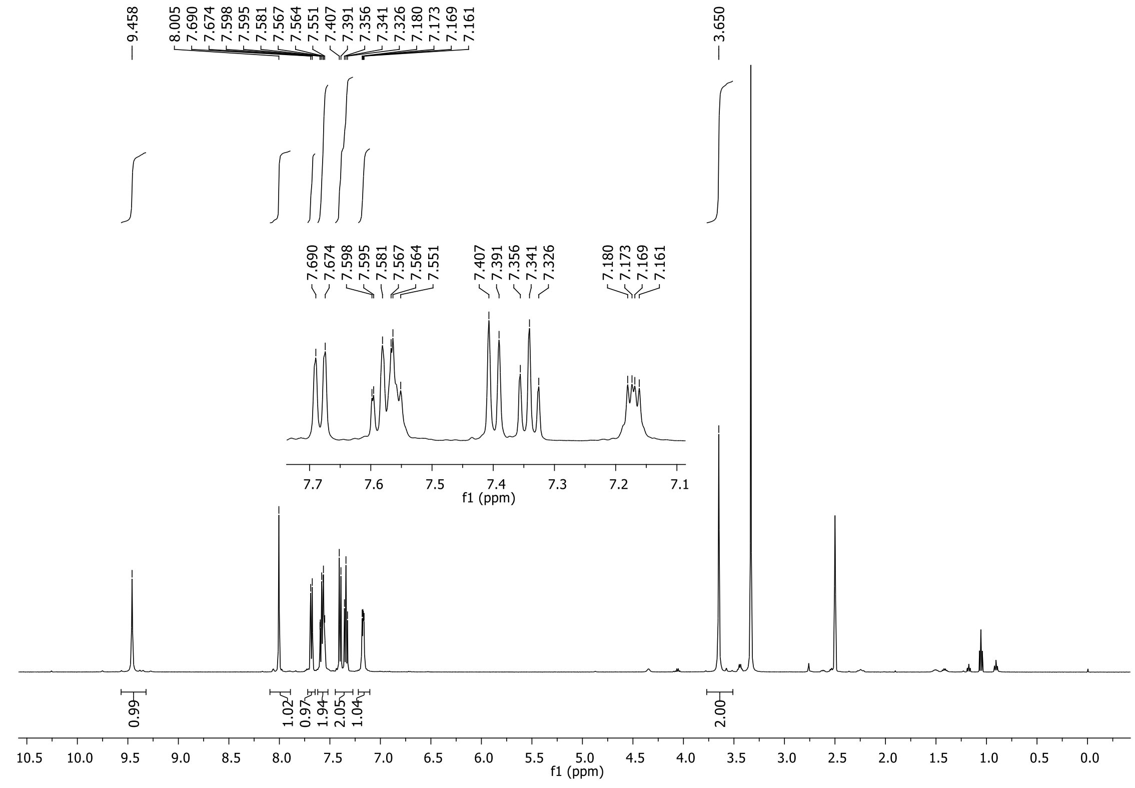

Figure S92. ^{1}H NMR (DMSO-d_6, 500 MHz) of **4w**

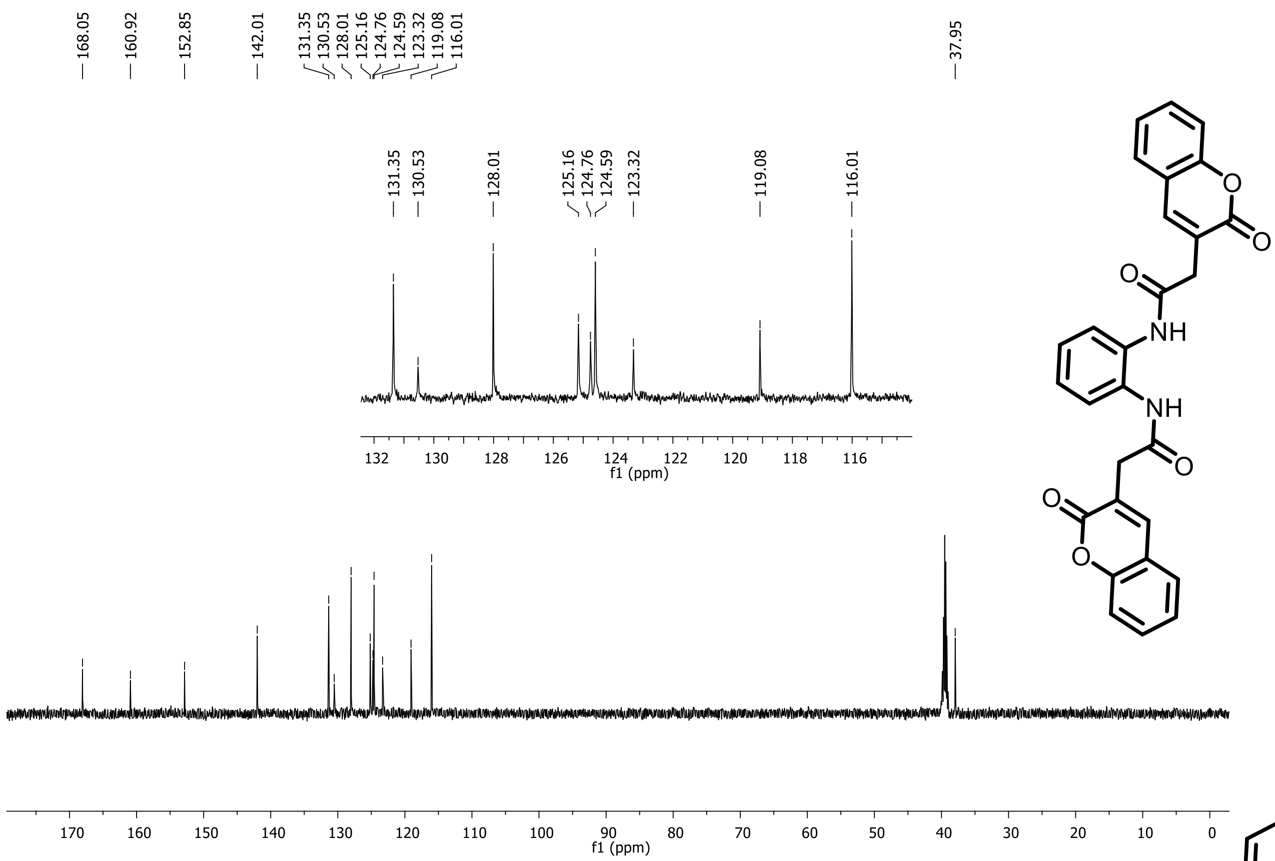

Figure S93. $^{13}C\{^{1}H\}$ NMR (DMSO-d$_6$, 125 MHz) **of 4w**

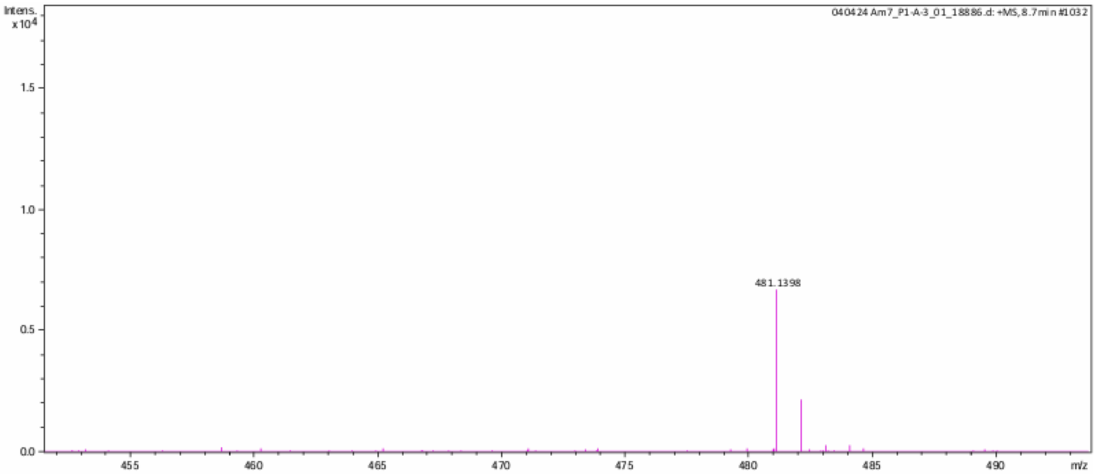

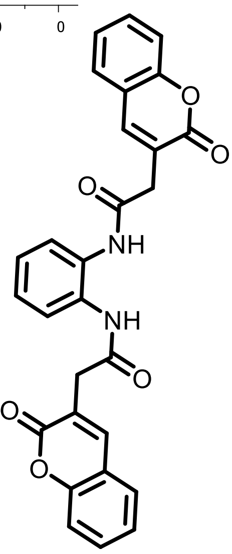

Figure S94. HRMS of **4w**

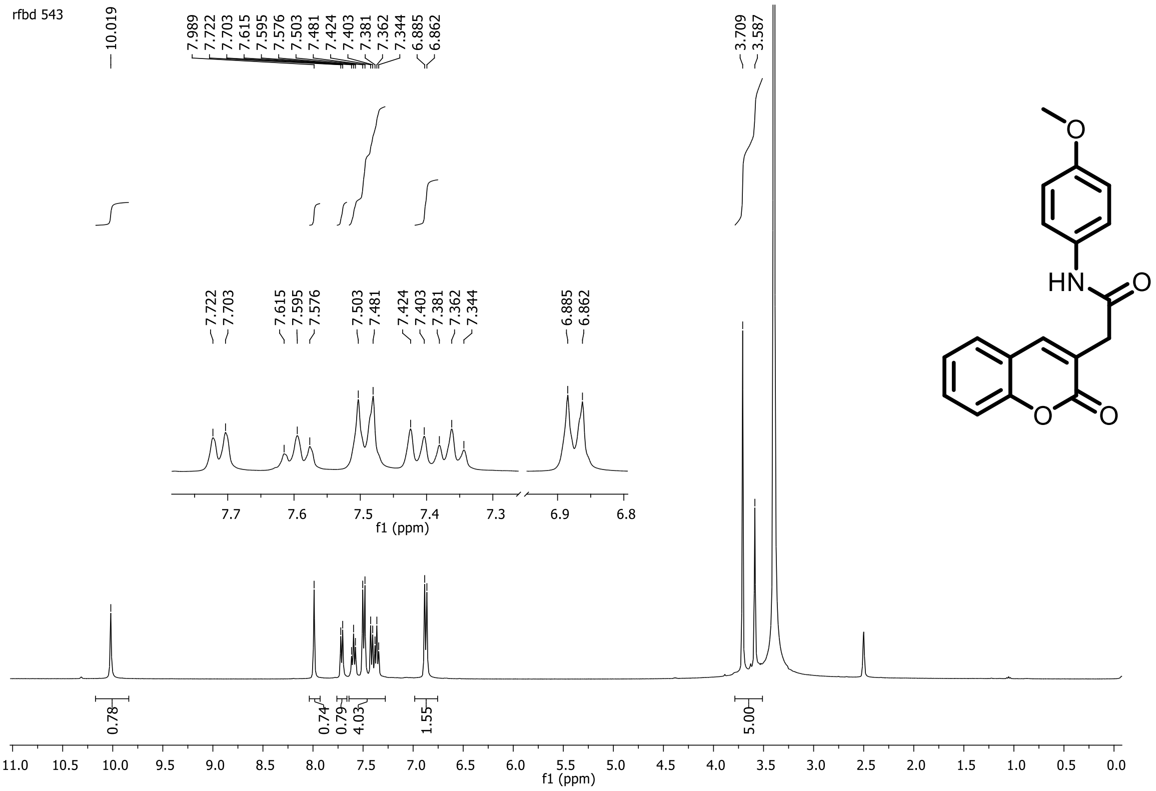

Figure S95. ^{1}H NMR (DMSO-d6, 500 MHz) of **4aa**

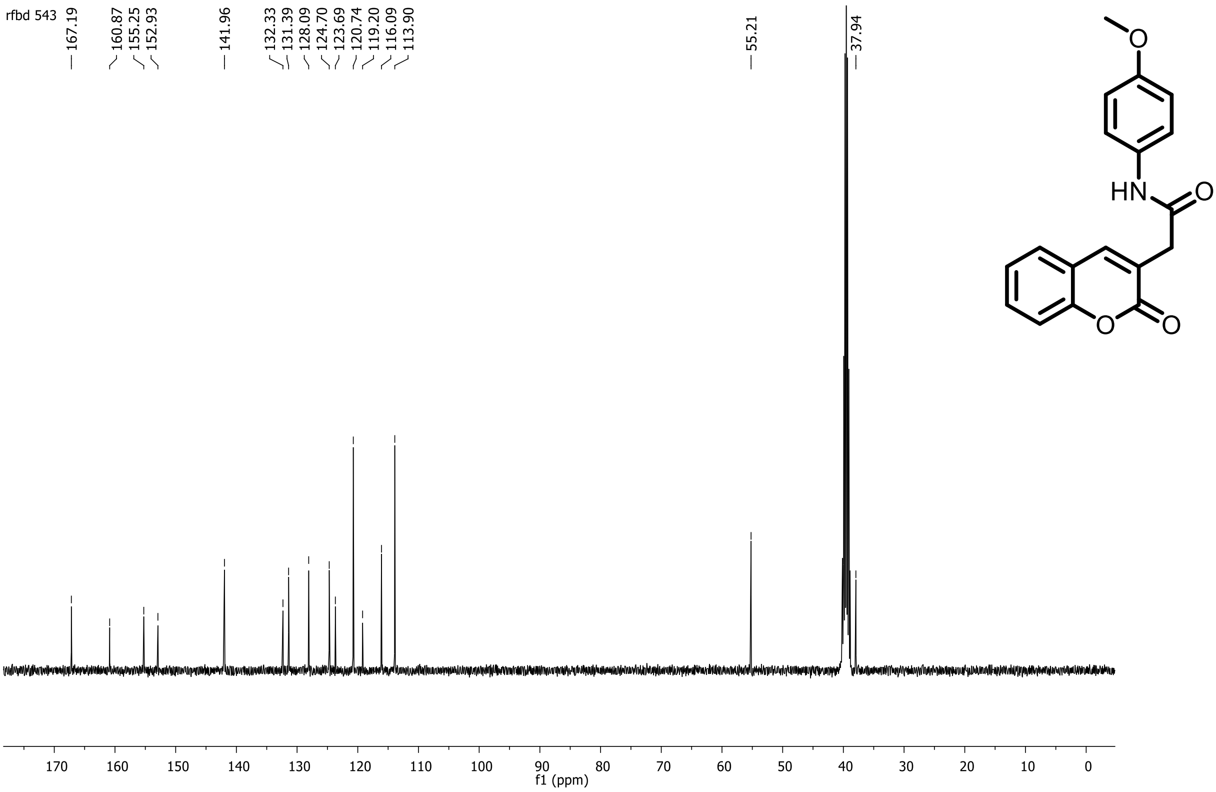

Figure S96. $^{13}C\{^{1}H\}$ NMR (DMSO-d6, 125 MHz) **of 4aa**

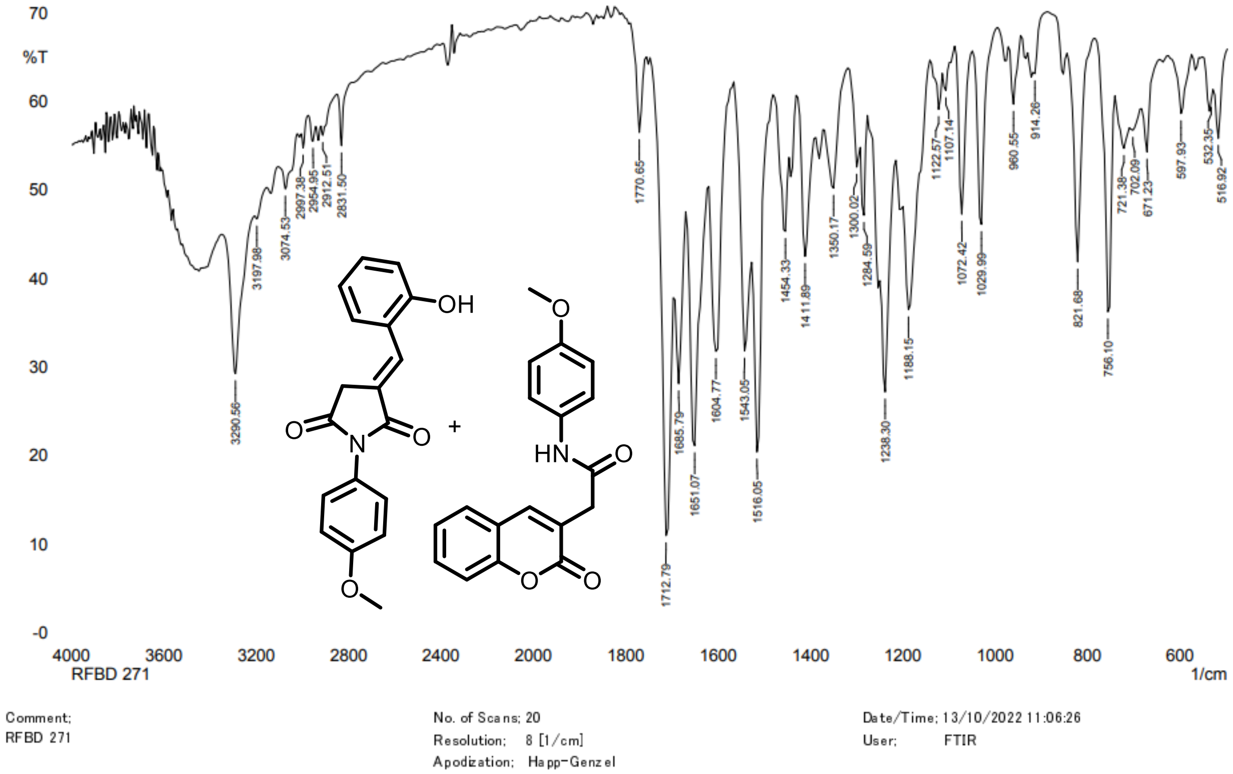

Figure S97. IR (KBr) of **4aa/4aa'**

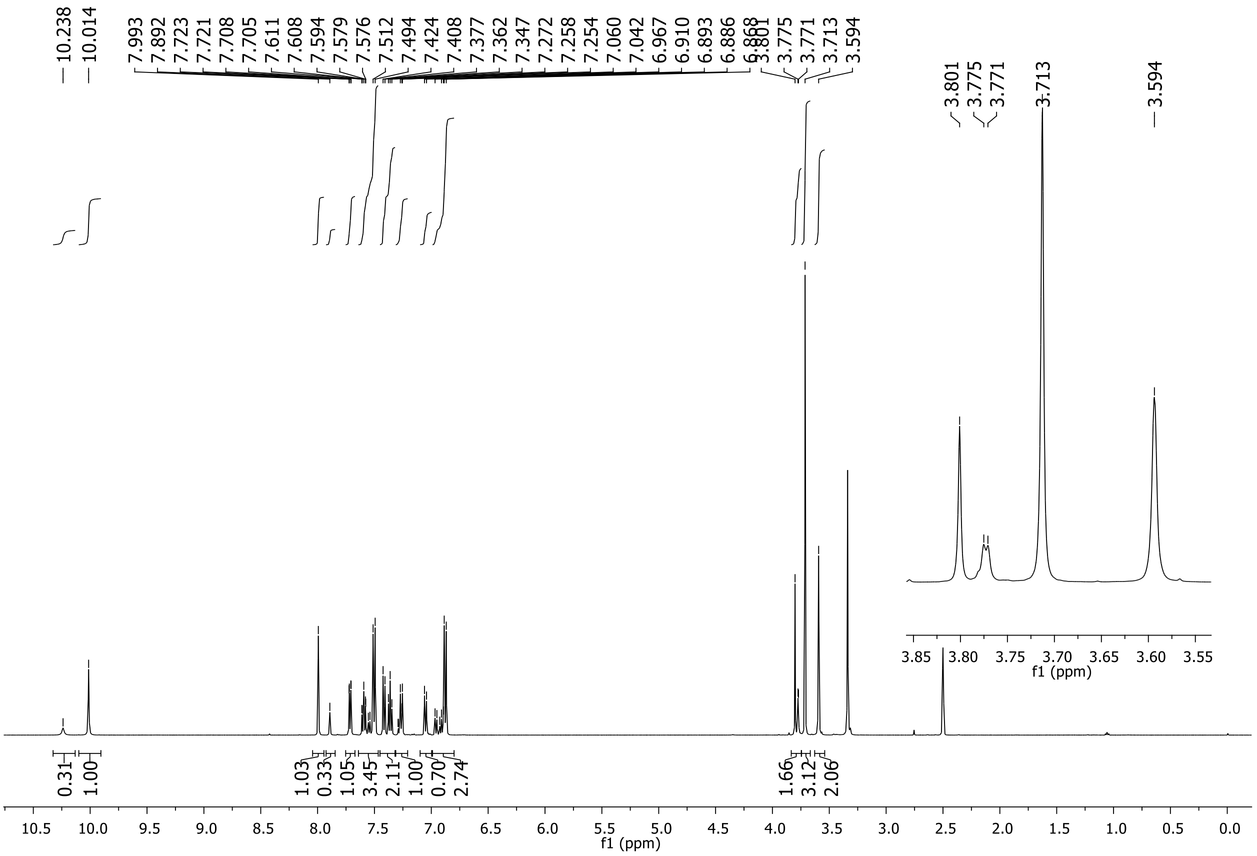

Figure S98. ^{1}H NMR (DMSO-d$_6$, 500 MHz) of **4aa/4aa'**

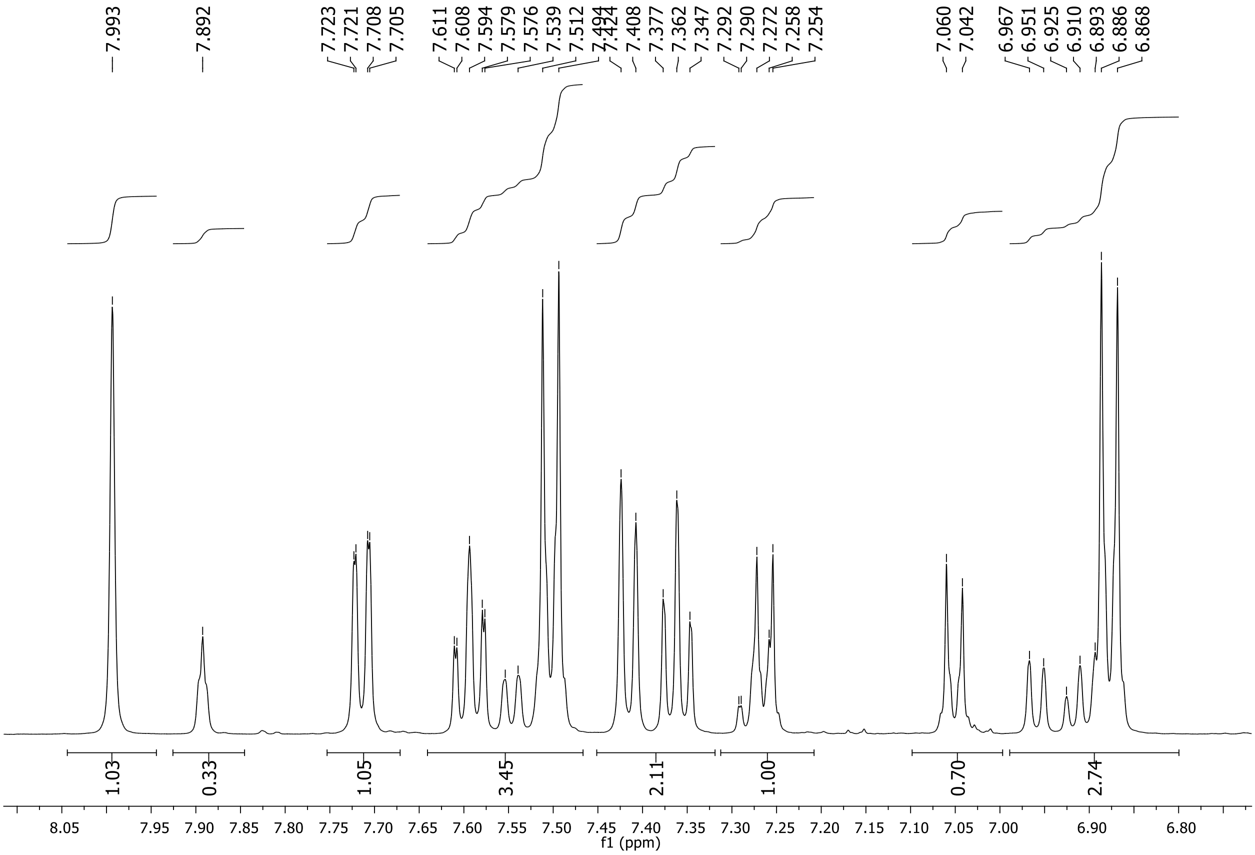

Figure S99. ^{1}H NMR (DMSO-d$_6$, 500 MHz) of **4aa/4aa'**

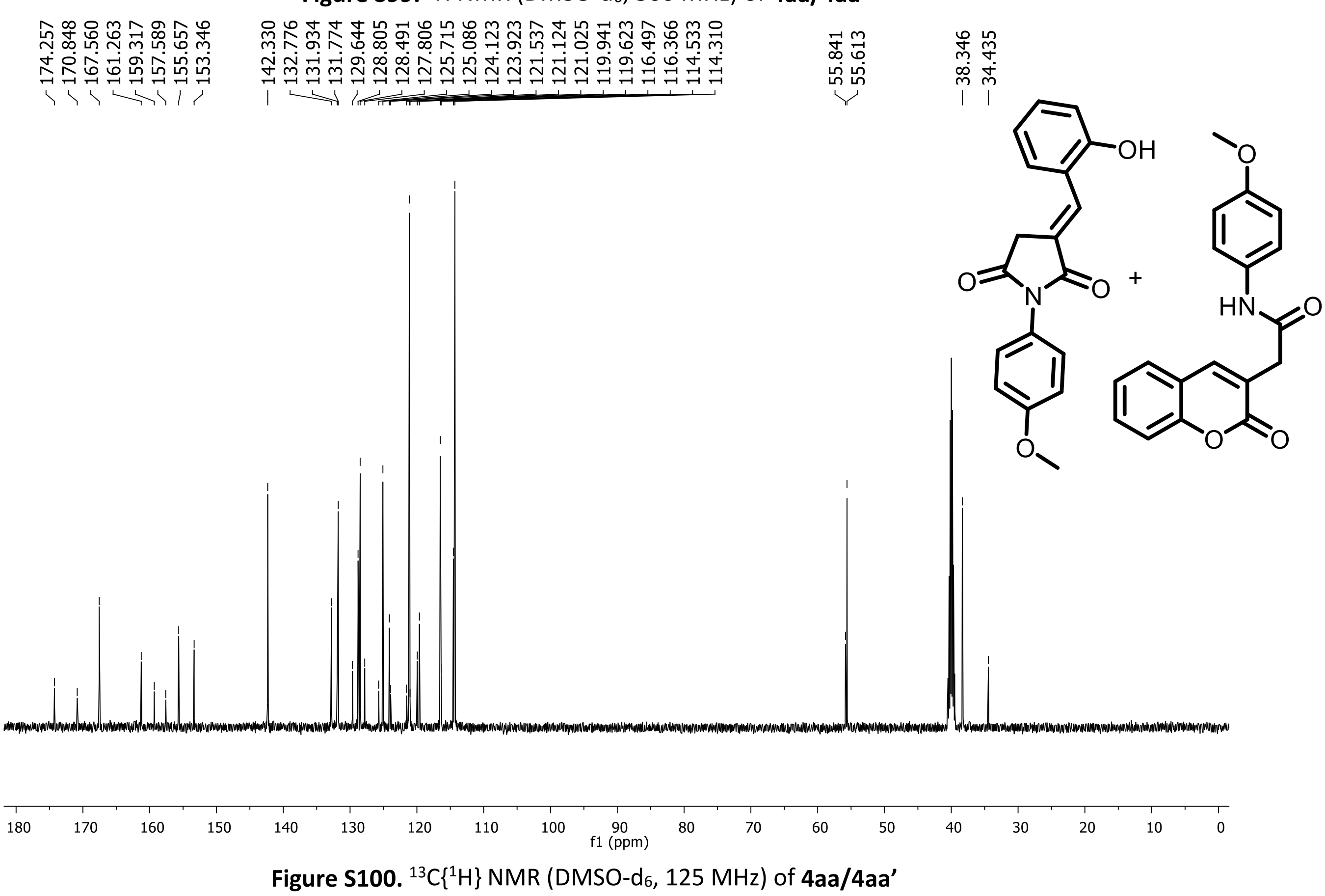

Figure S100. ^{13}C{^{1}H} NMR (DMSO-d$_6$, 125 MHz) of **4aa/4aa'**

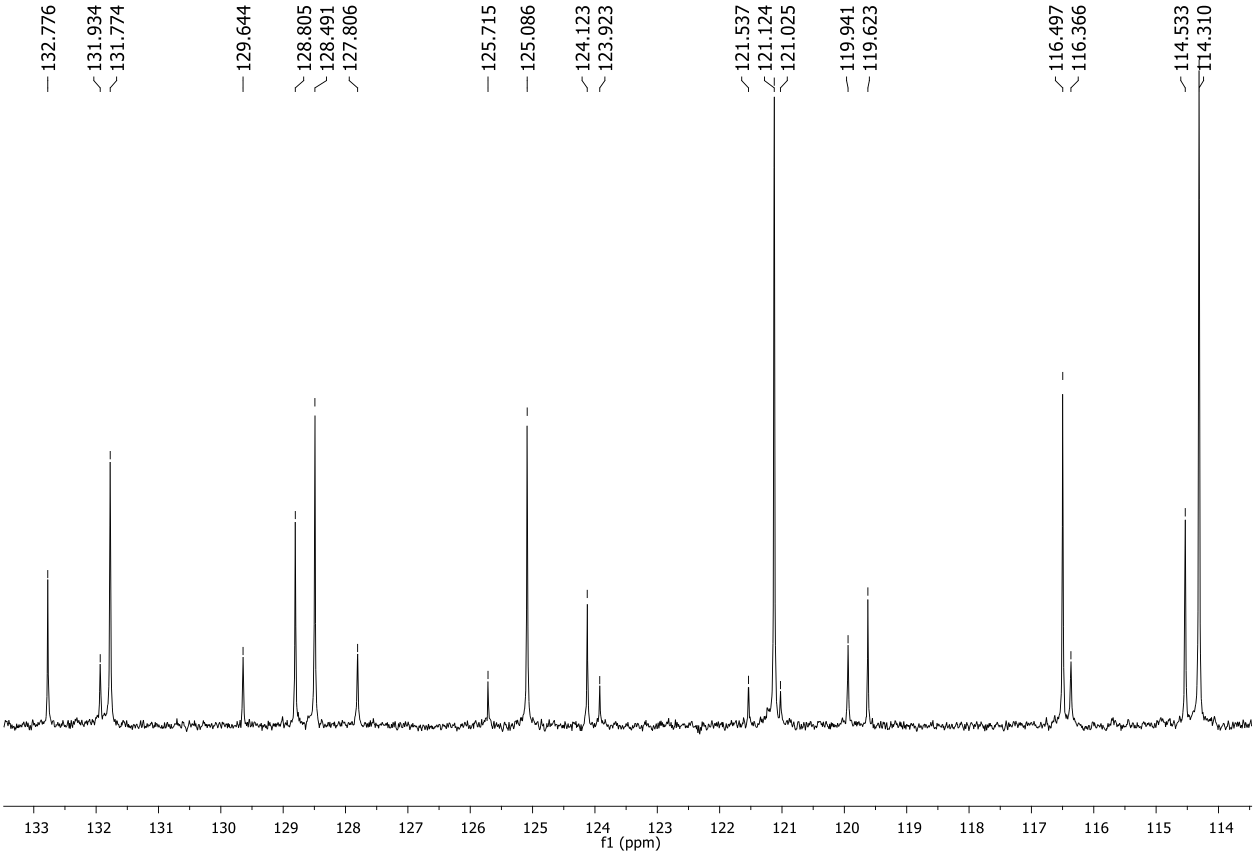

Figure S101. $^{13}C\{^1H\}$ NMR (DMSO-d$_6$, 125 MHz) of **4aa/4aa'**

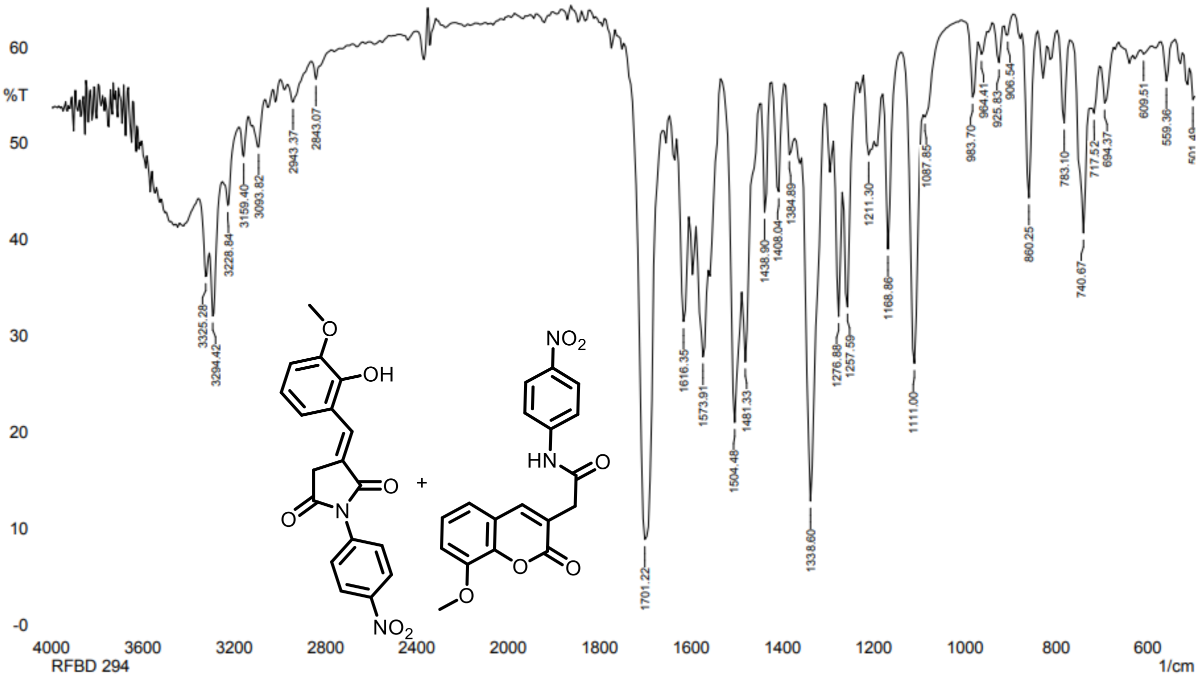

Comment;
RFBD 294

No. of Scans; 20
Resolution; 8 [1/cm]
Apodization; Happ-Genzel

Date/Time; 01/12/2022 16:06:47
User; FTIR

Figure S102. IR (KBr) of **4ab/4ab'**

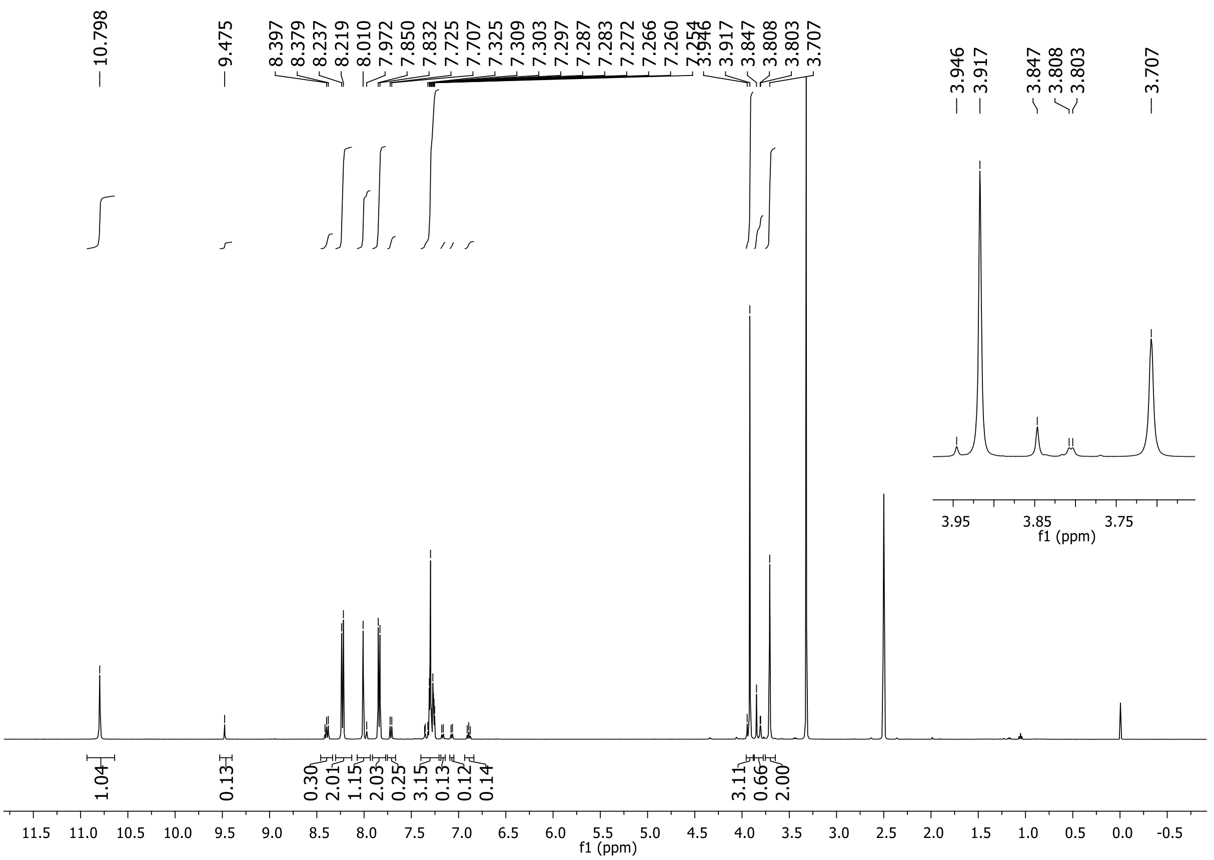

Figure S103. ^{1}H NMR (DMSO-d_6, 500 MHz) of **4ab/4ab**

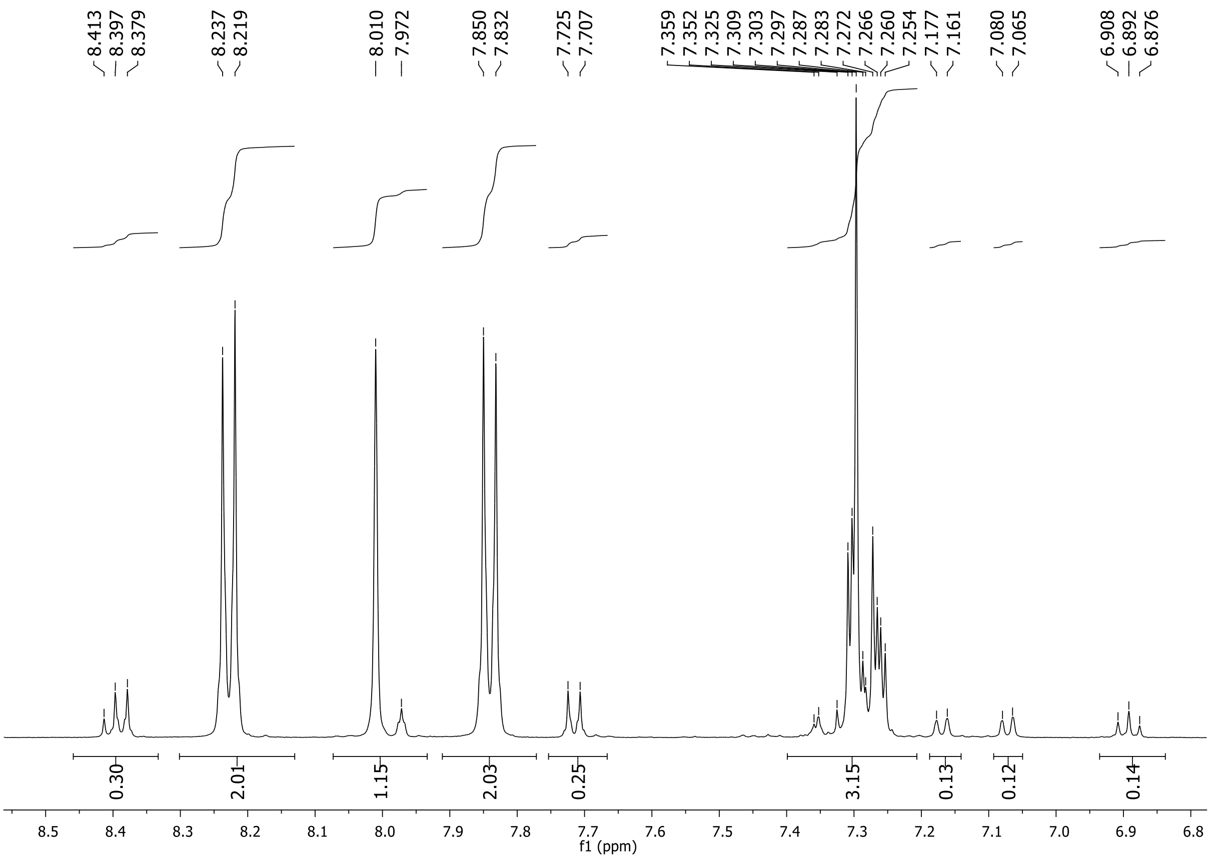

Figure S104. ^{1}H NMR (DMSO-d_6, 500 MHz) of **4ab/4ab**

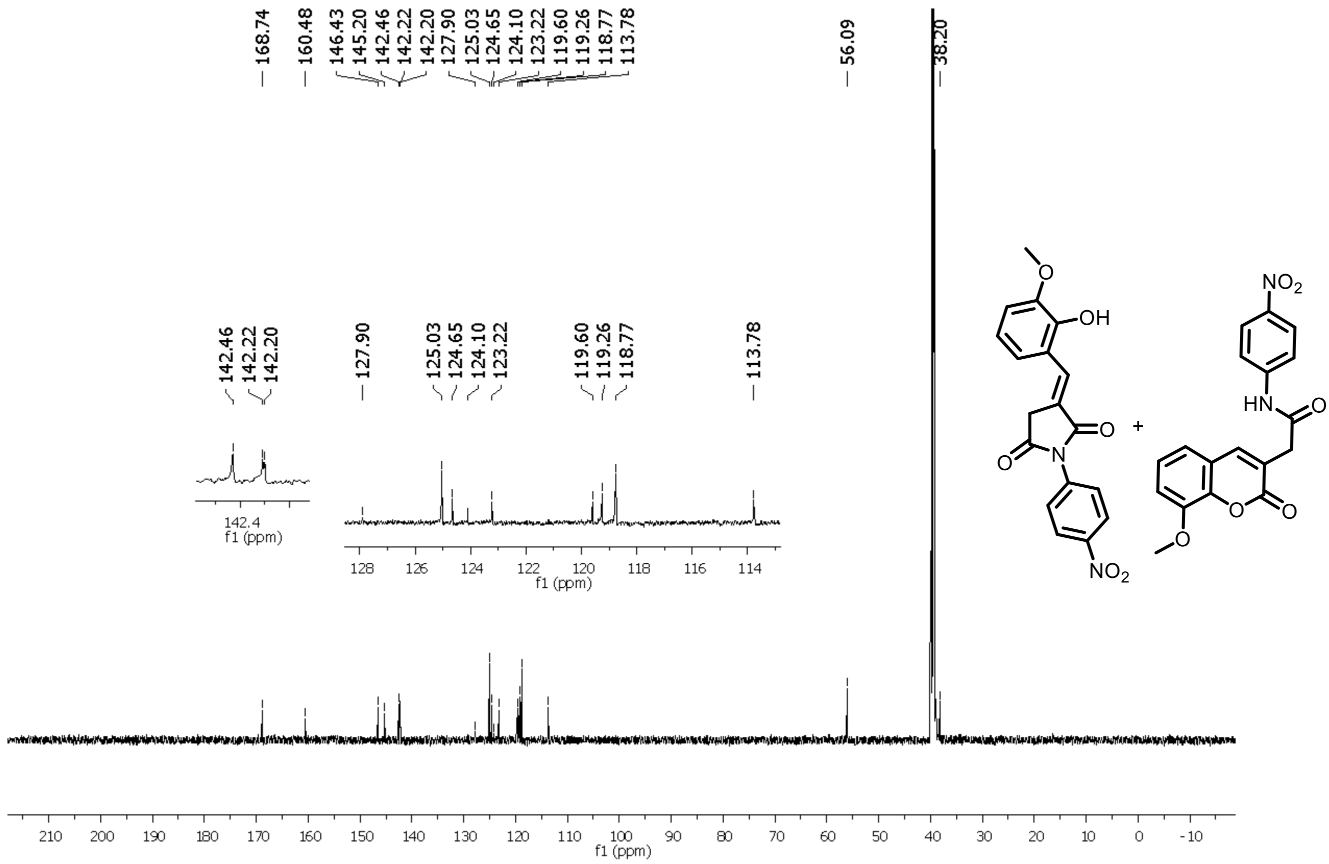

Figure S105. $^{13}C\{^{1}H\}$ NMR (DMSO-d$_6$, 125 MHz) of **4ab/4ab**

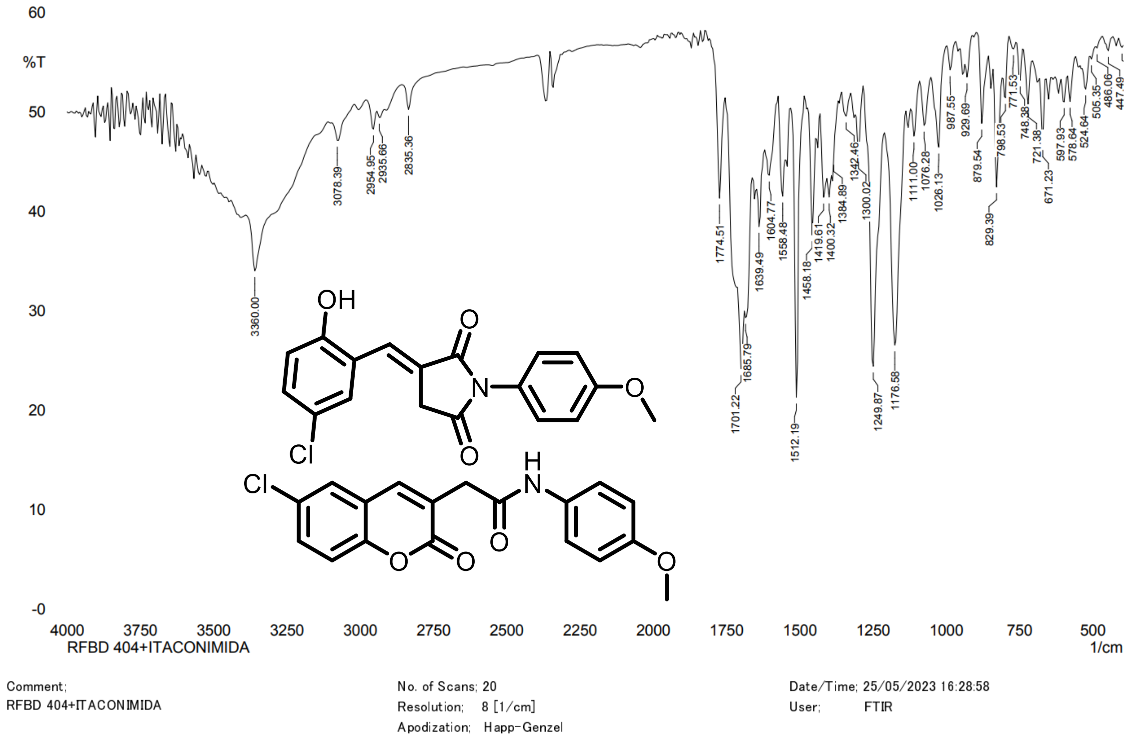

Figure S106. IR (KBr) of **4ac/4ac’**

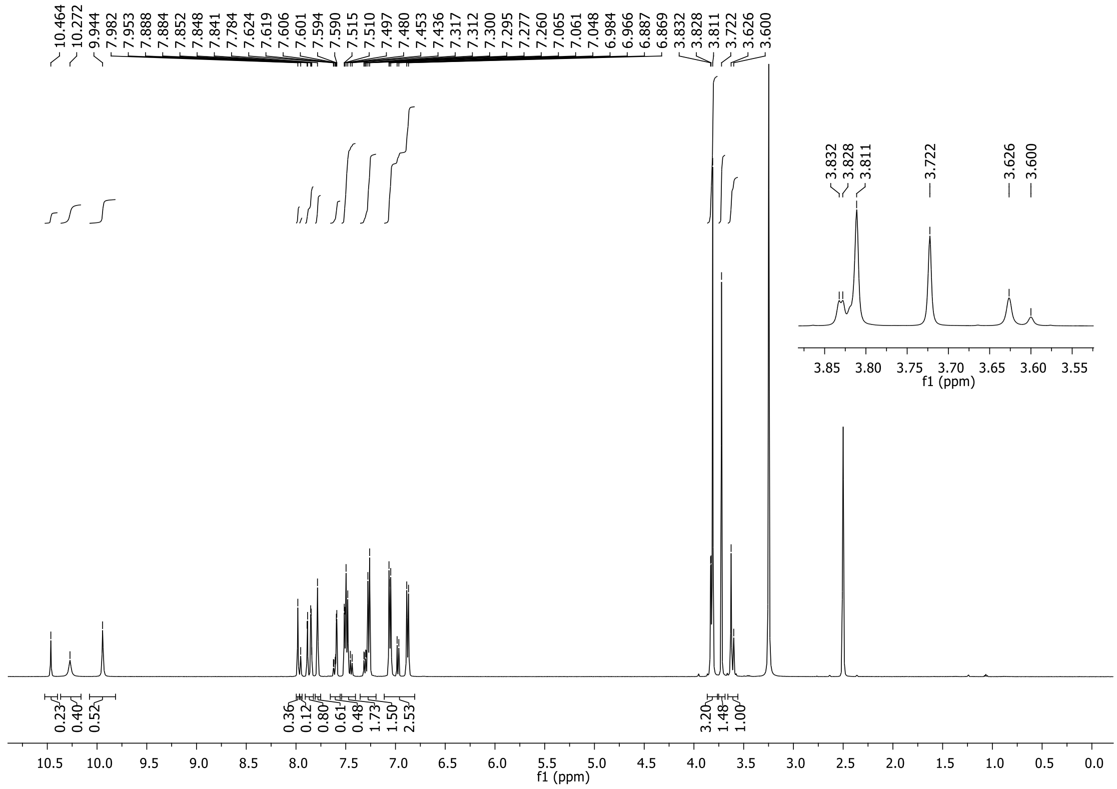

Figure S107. ^{1}H NMR (DMSO-d$_6$, 500 MHz) of **4ac/4ac'**

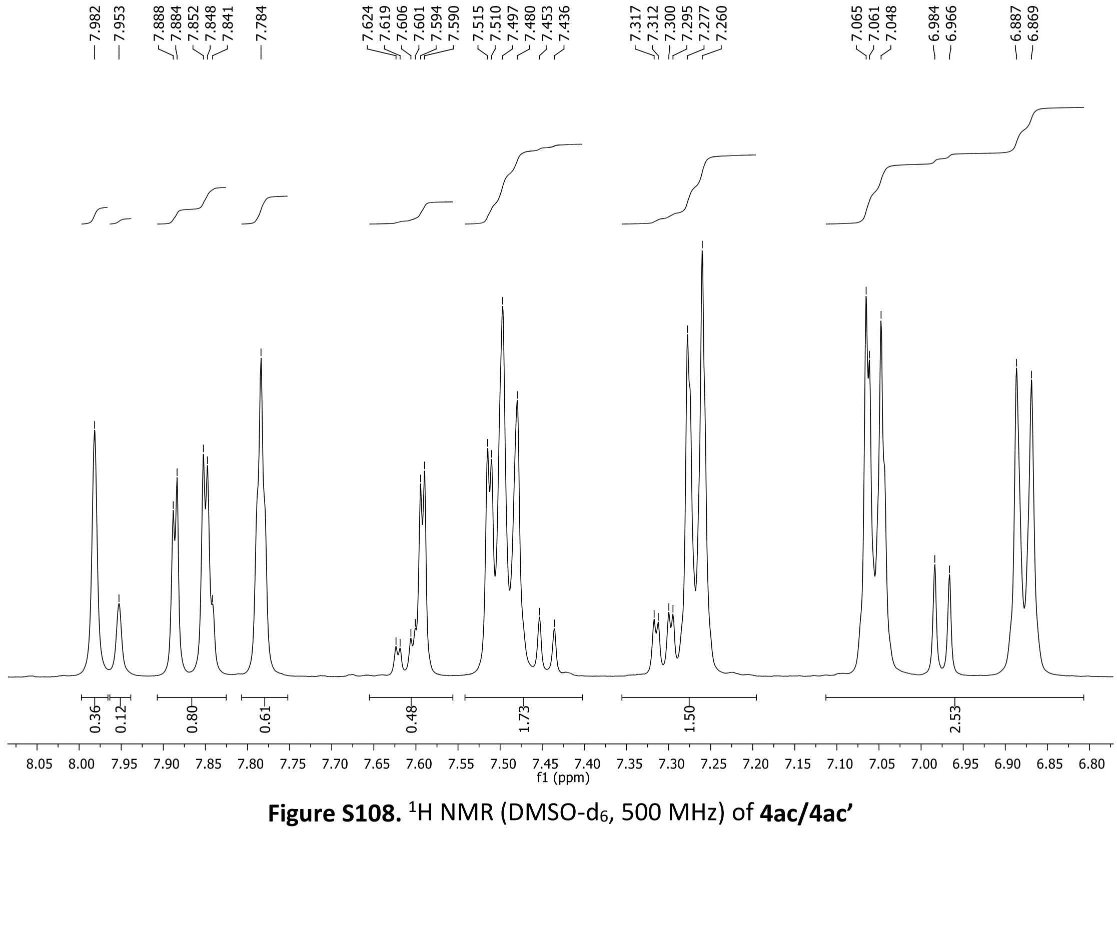

Figure S108. ^{1}H NMR (DMSO-d$_6$, 500 MHz) of **4ac/4ac'**

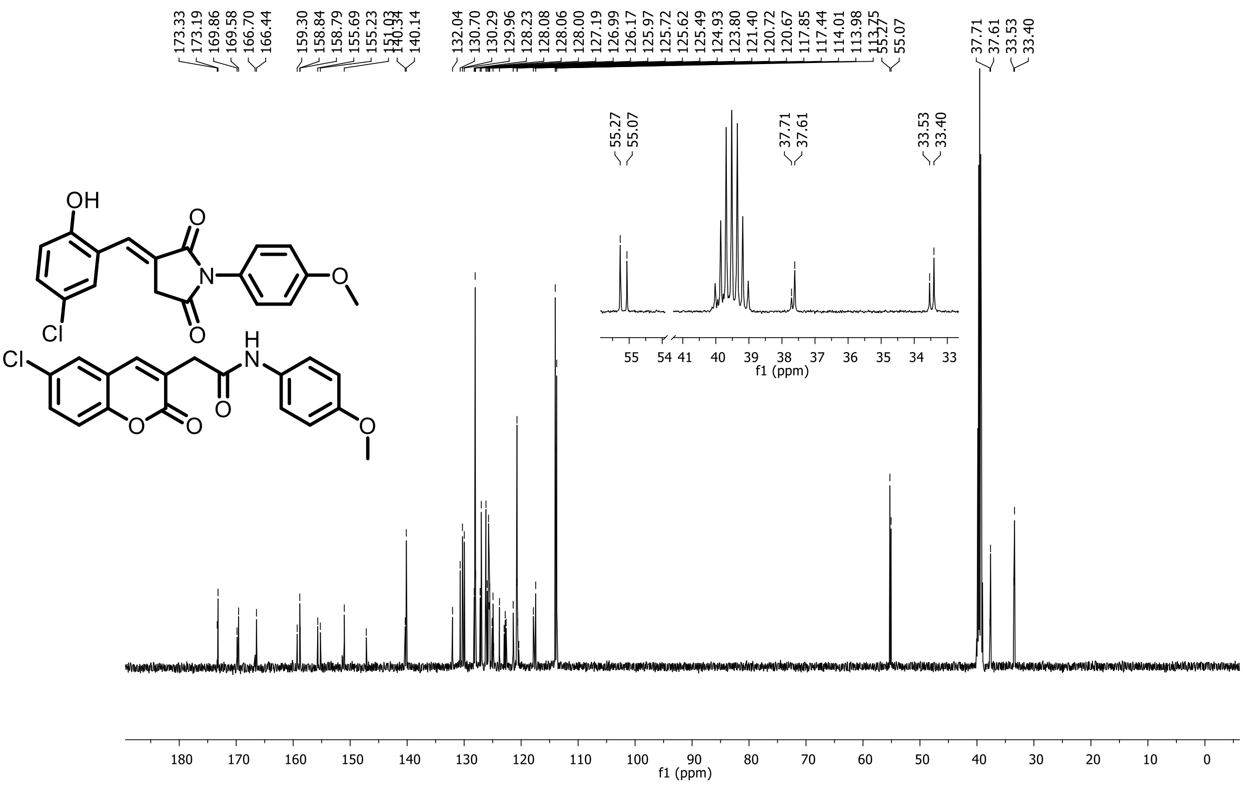

Figure S109. $^{13}C\{^{1}H\}$ NMR (DMSO-d$_6$, 125 MHz) of **4ac/4ac'**

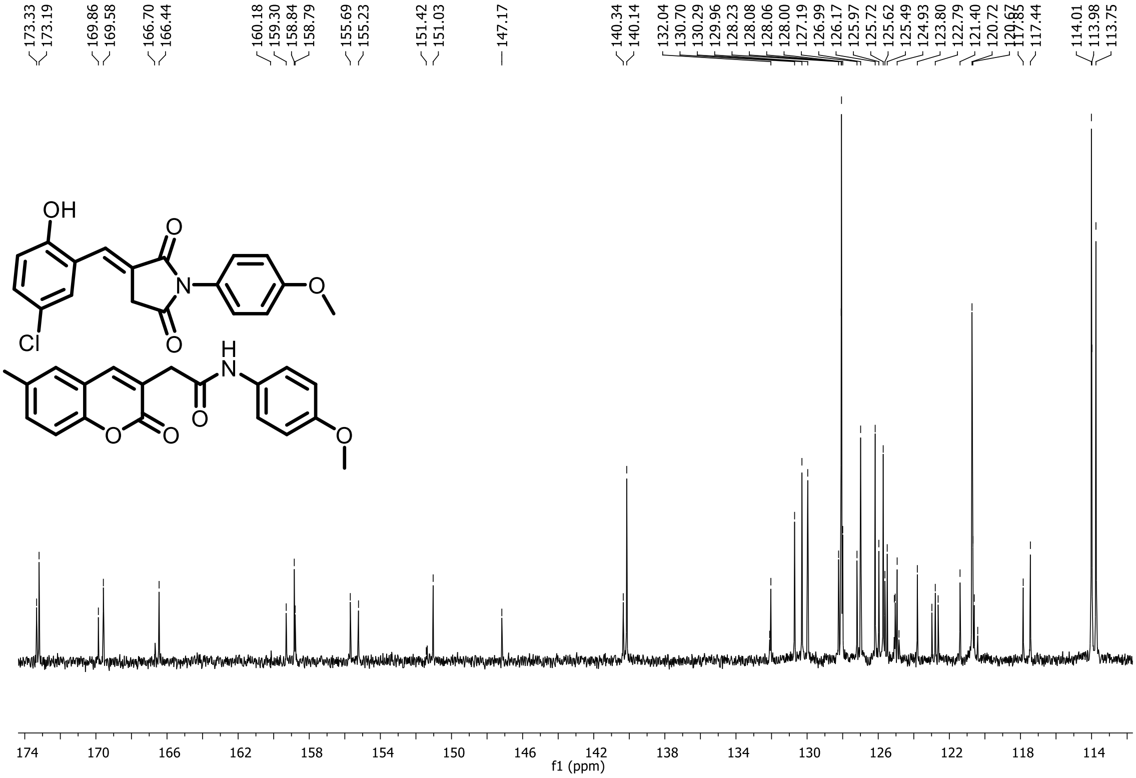

Figure S110. $^{13}C\{^{1}H\}$ NMR (DMSO-d$_6$, 125 MHz) of **4ac/4ac'**

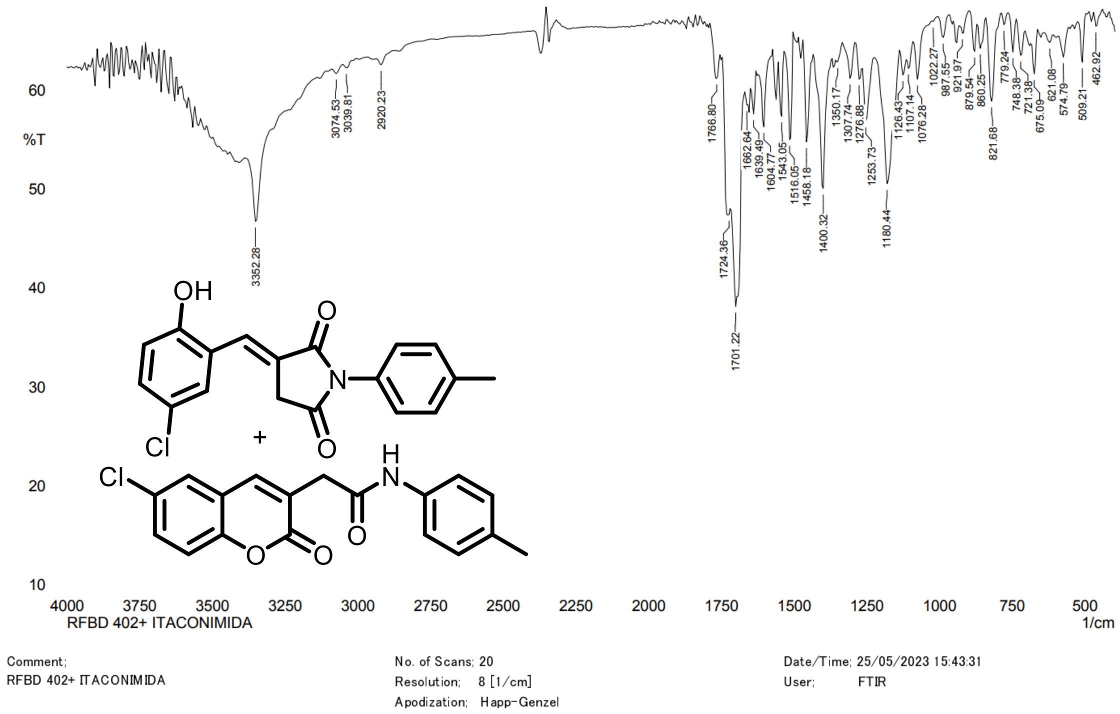

Figure S111. IR (KBr) of **4ad/4ad'**

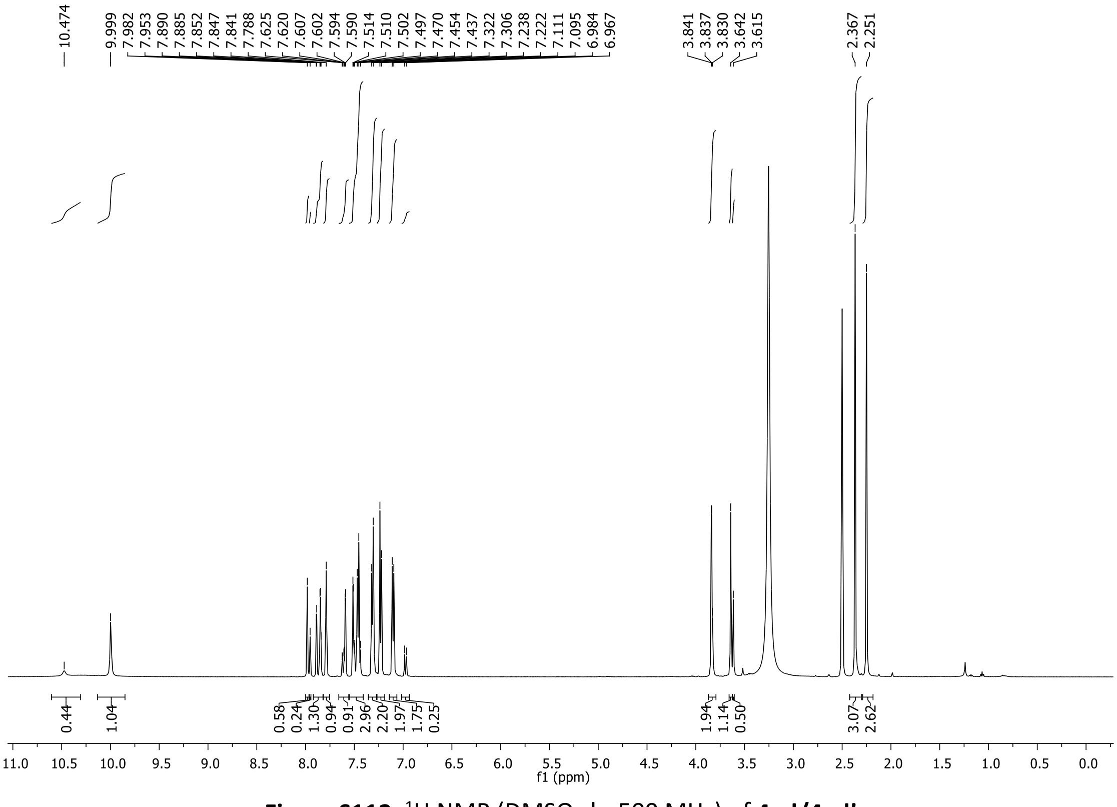

Figure S112. ^{1}H NMR (DMSO-d$_6$, 500 MHz) of **4ad/4ad'**

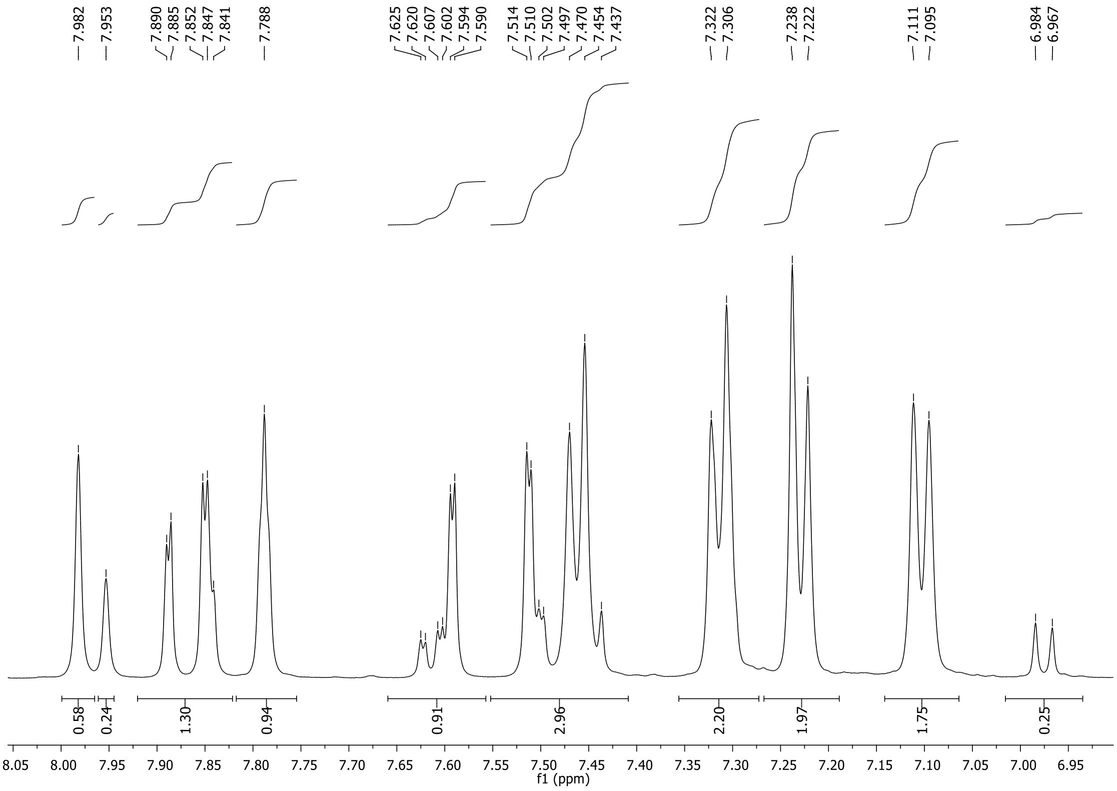

Figure S113. ^{1}H NMR (DMSO-d$_6$, 500 MHz) of **4ad/4ad'**

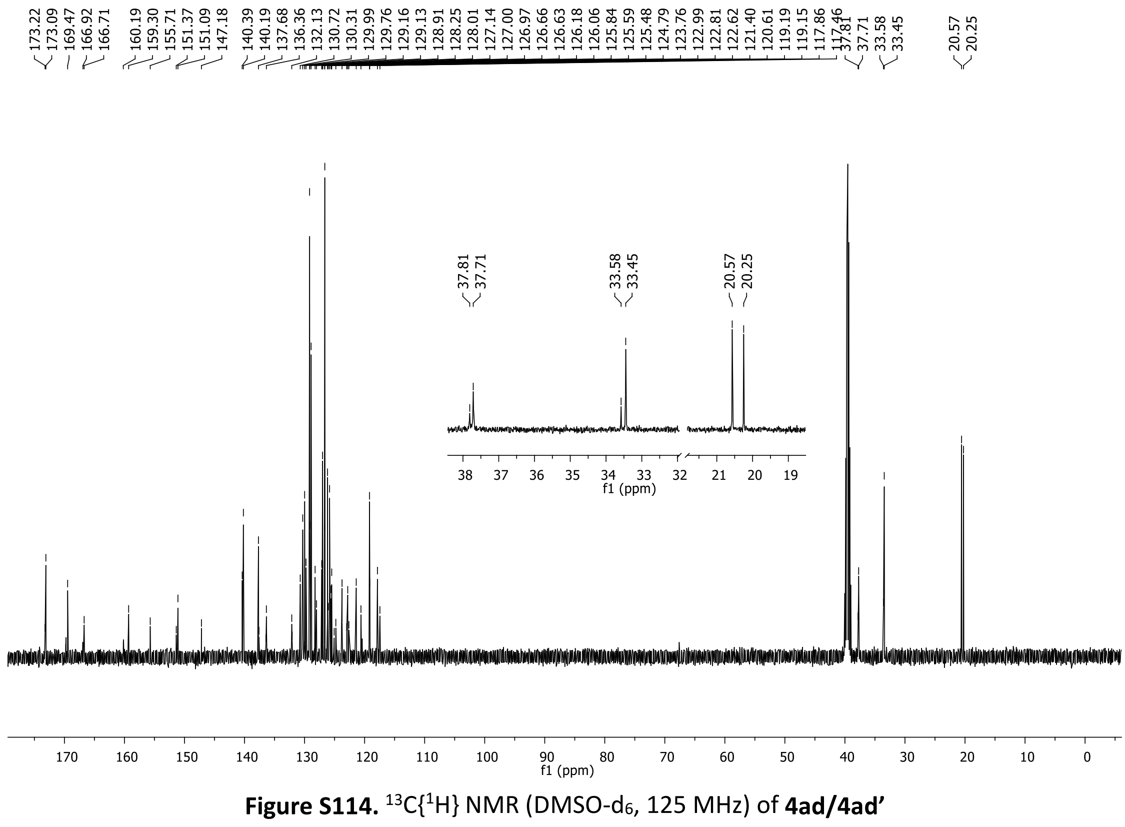

Figure S114. $^{13}C\{^{1}H\}$ NMR (DMSO-d$_6$, 125 MHz) of **4ad/4ad'**

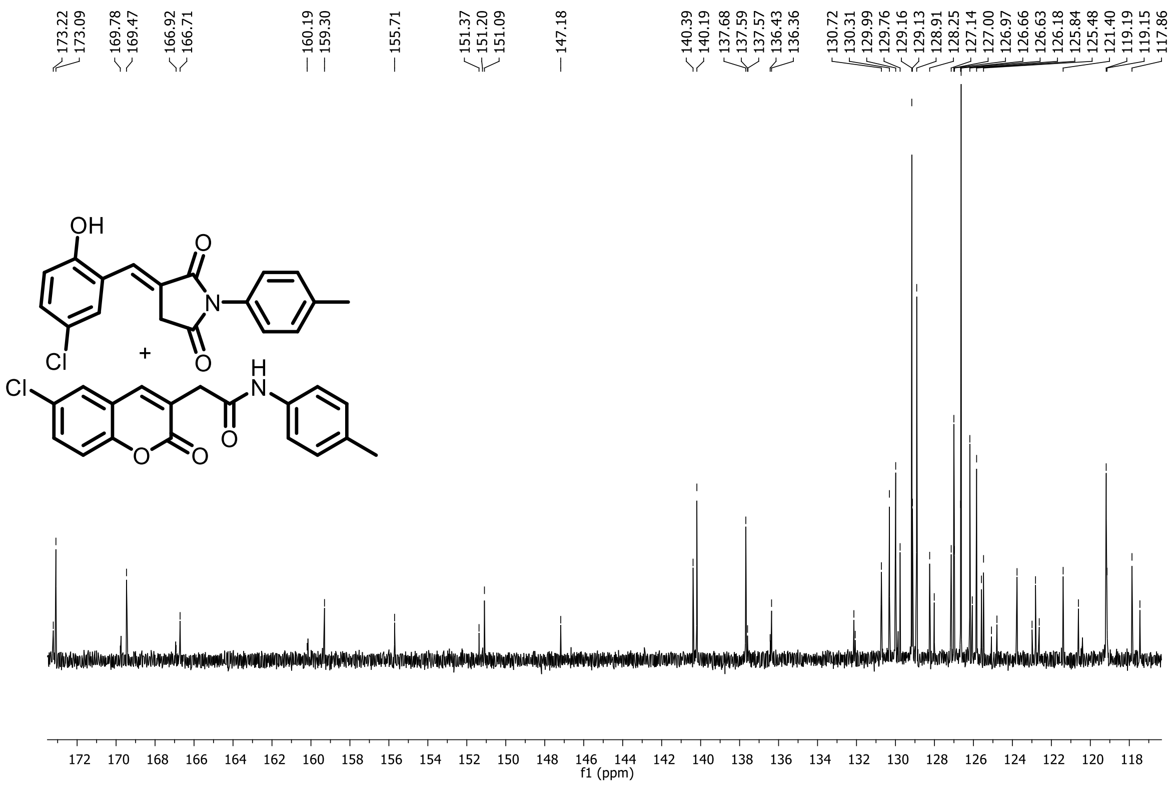

Figure S115. $^{13}C\{^{1}H\}$ NMR (DMSO-d$_6$, 125 MHz) of **4ad/4ad'**

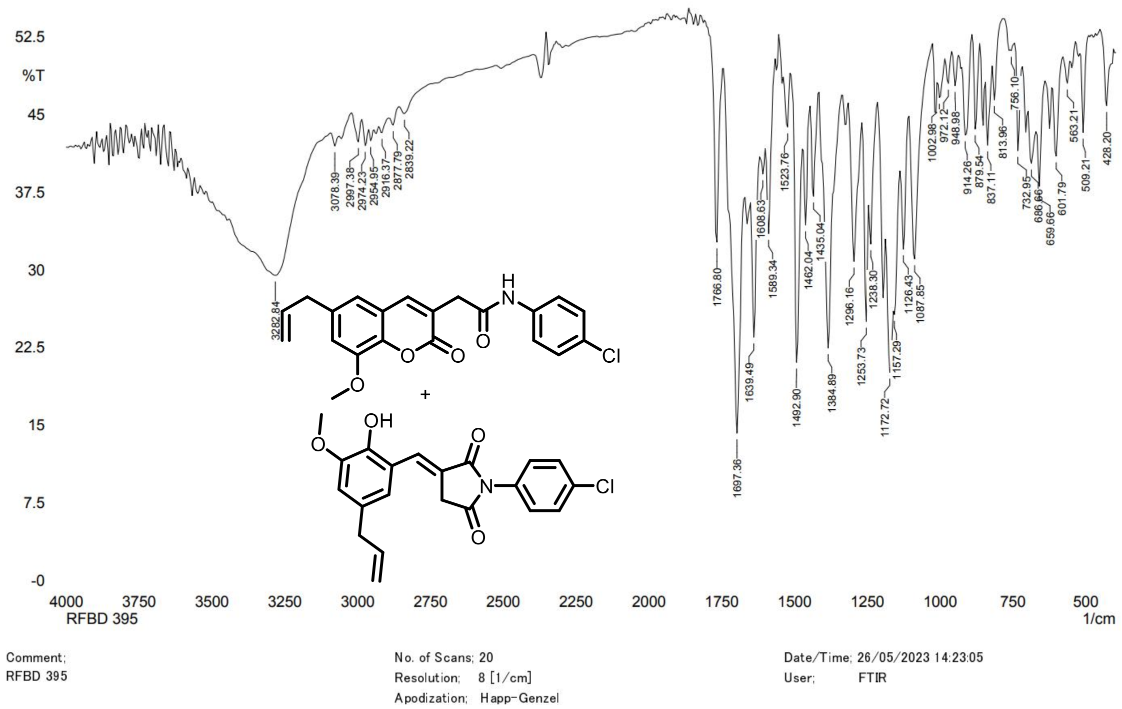

Figure S116. IR (KBr) of **4ae/4ae'**

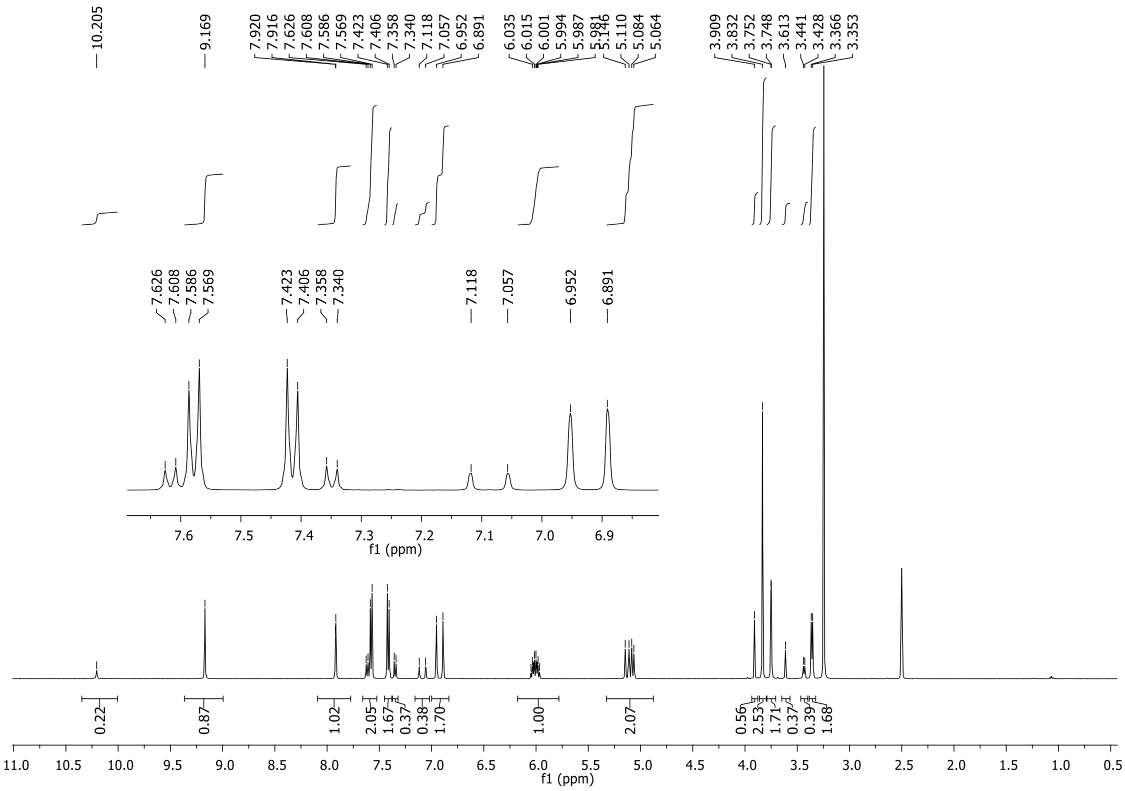

Figure S117. ^{1}H NMR (DMSO-d_6, 500 MHz) of **4ae/4ae'**

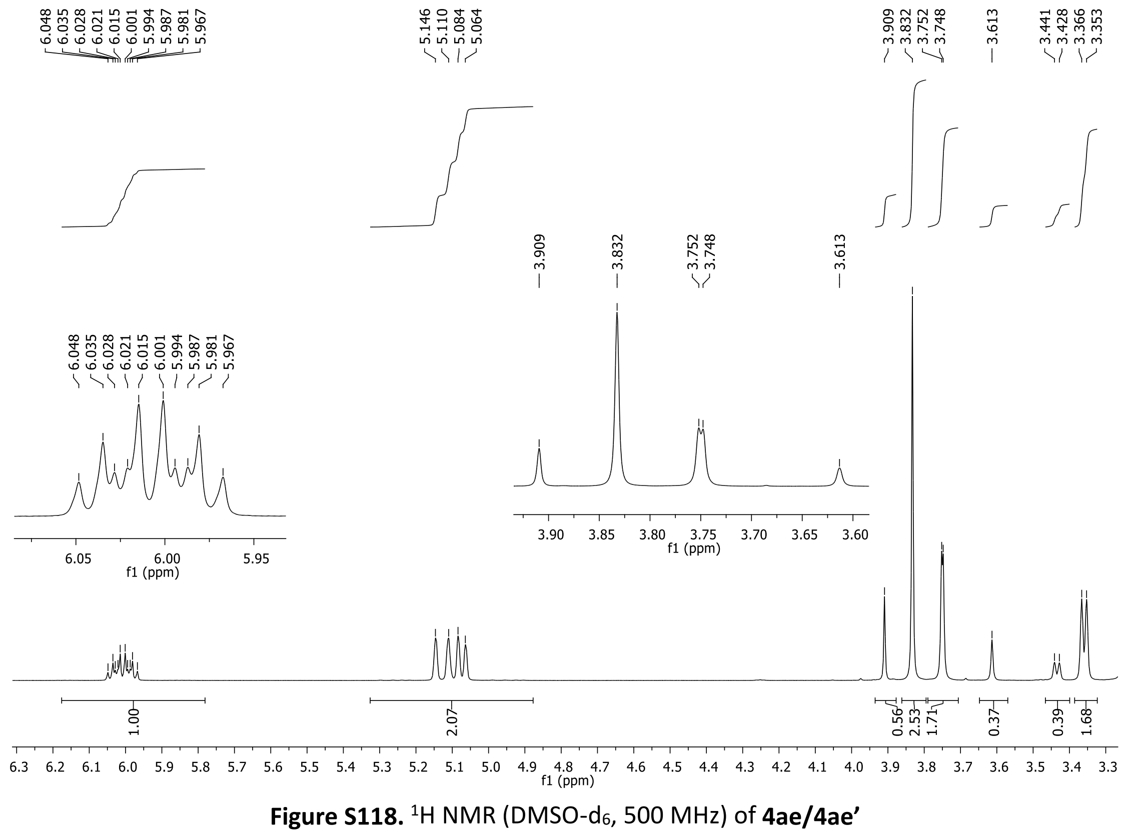

Figure S118. ^{1}H NMR (DMSO-d_6, 500 MHz) of **4ae/4ae'**

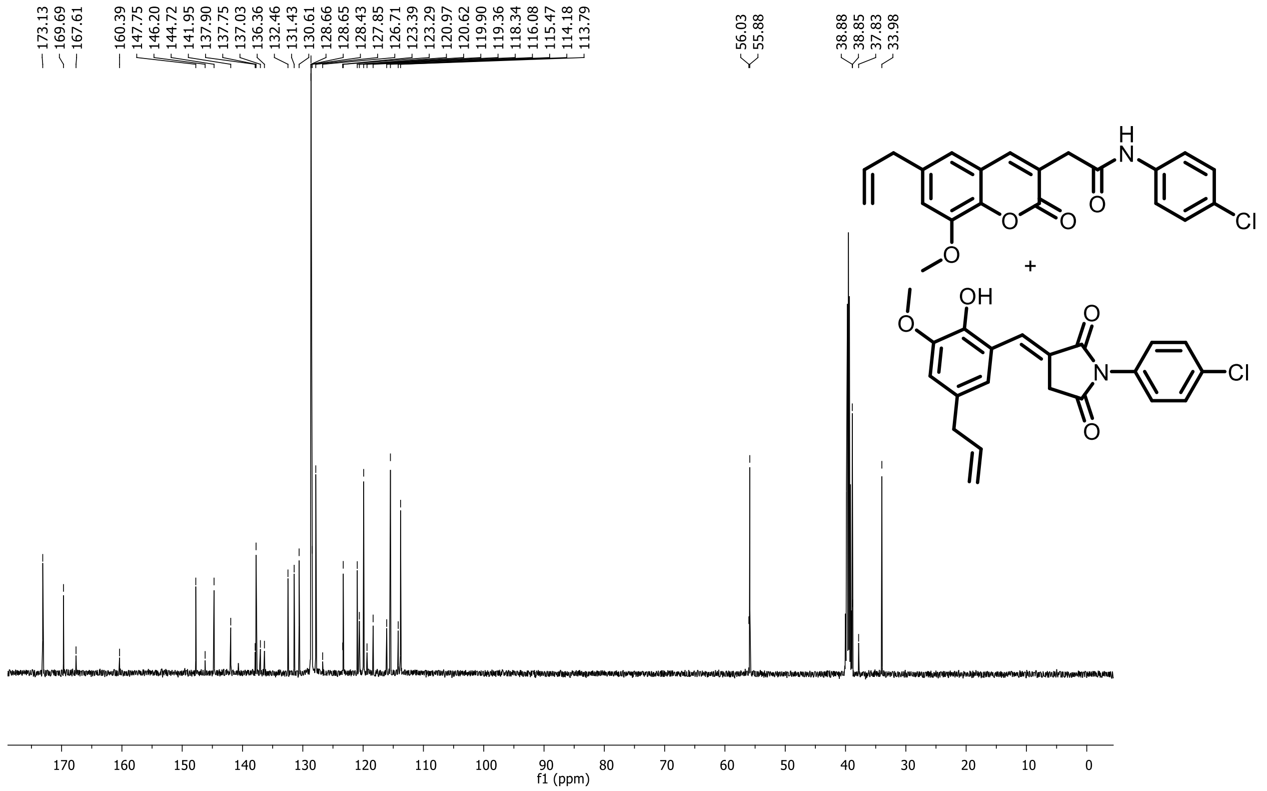

Figure S119. $^{13}C\{^{1}H\}$ NMR (DMSO-d$_6$, 125 MHz) of **4ae/4ae'**

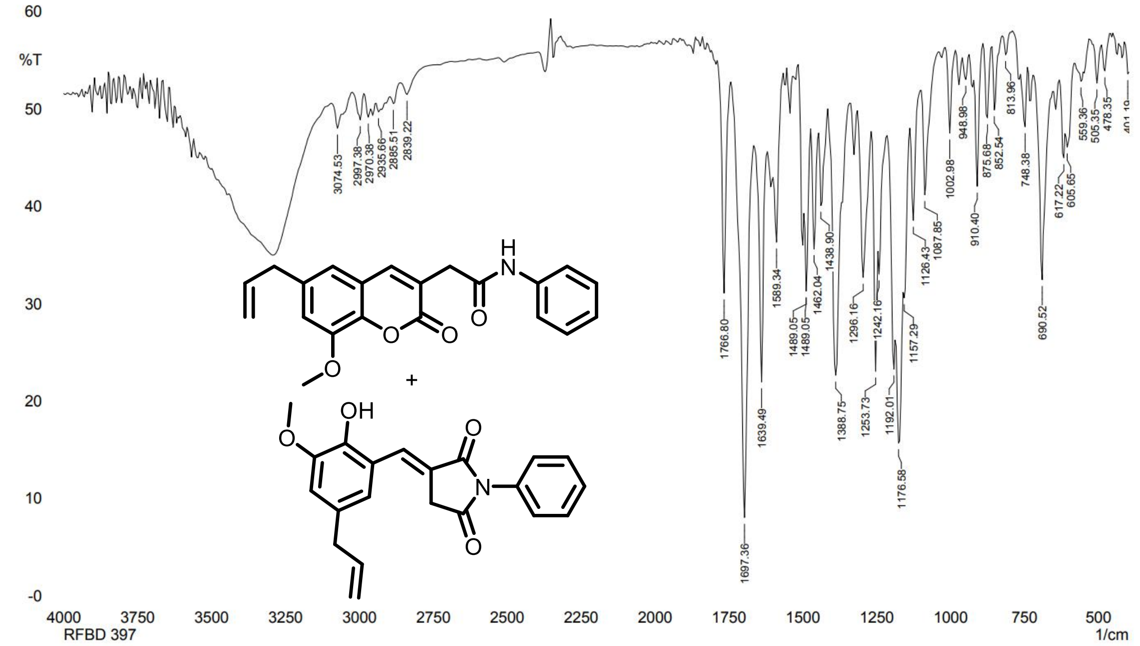

Comment;
RFBD 397

No. of Scans; 20
Resolution; 8 [1/cm]
Apodization; Happ-Genzel

Date/Time; 25/05/2023 17:23:25
User; FTIR

Figure S120. IR (KBr) of **4af/4af'**

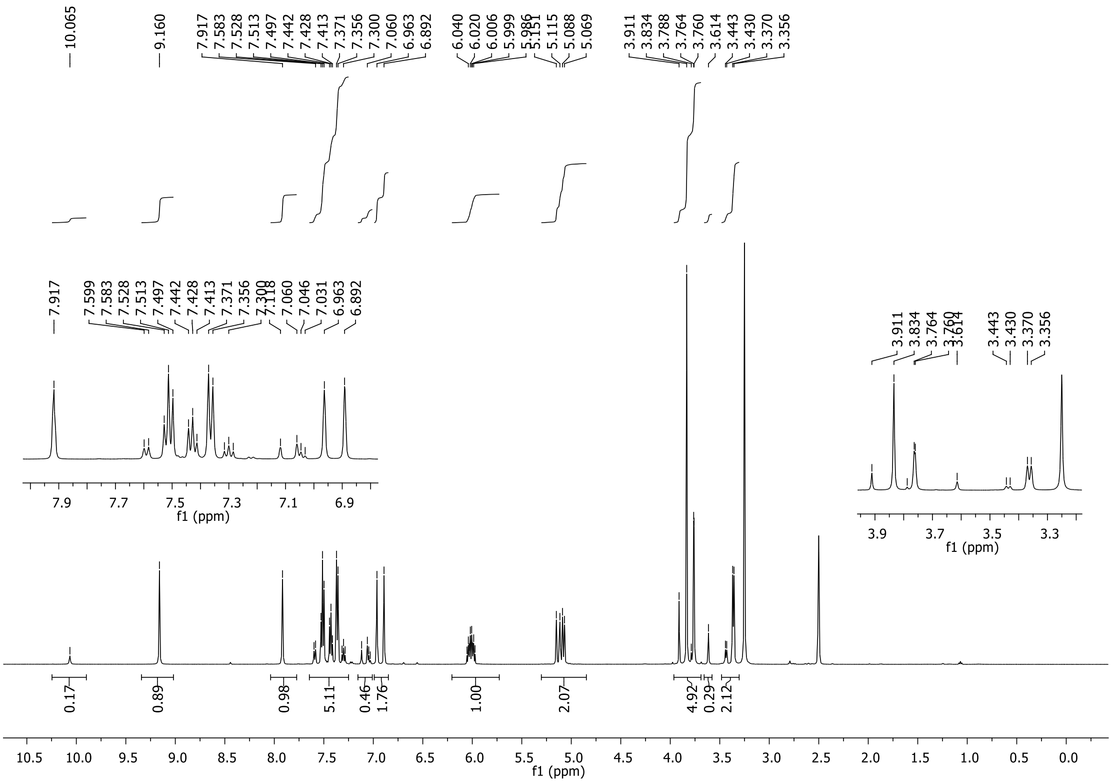

Figure S121. ^{1}H NMR (DMSO-d$_6$, 500 MHz) of **4af/4af'**

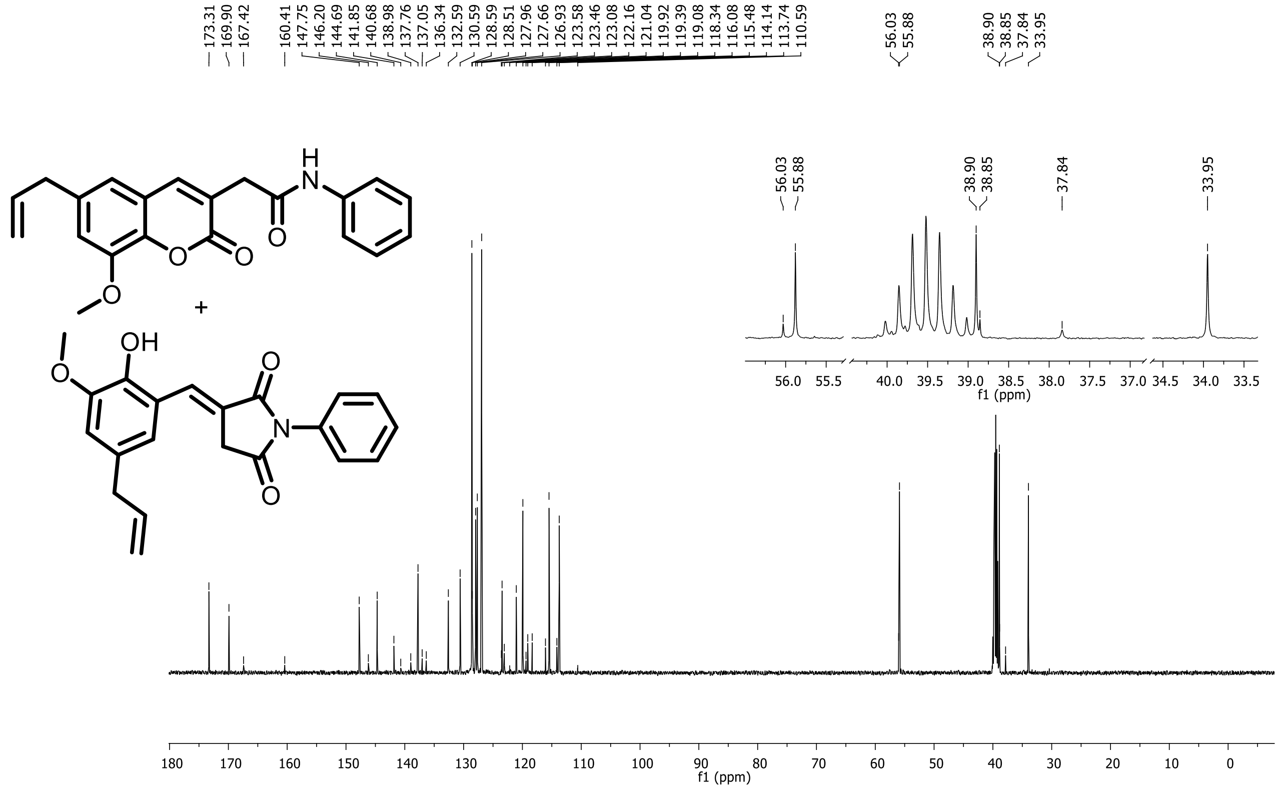

Figure S122. ^{13}C{^{1}H} NMR (DMSO-d$_6$, 125 MHz) of **4af/4af'**

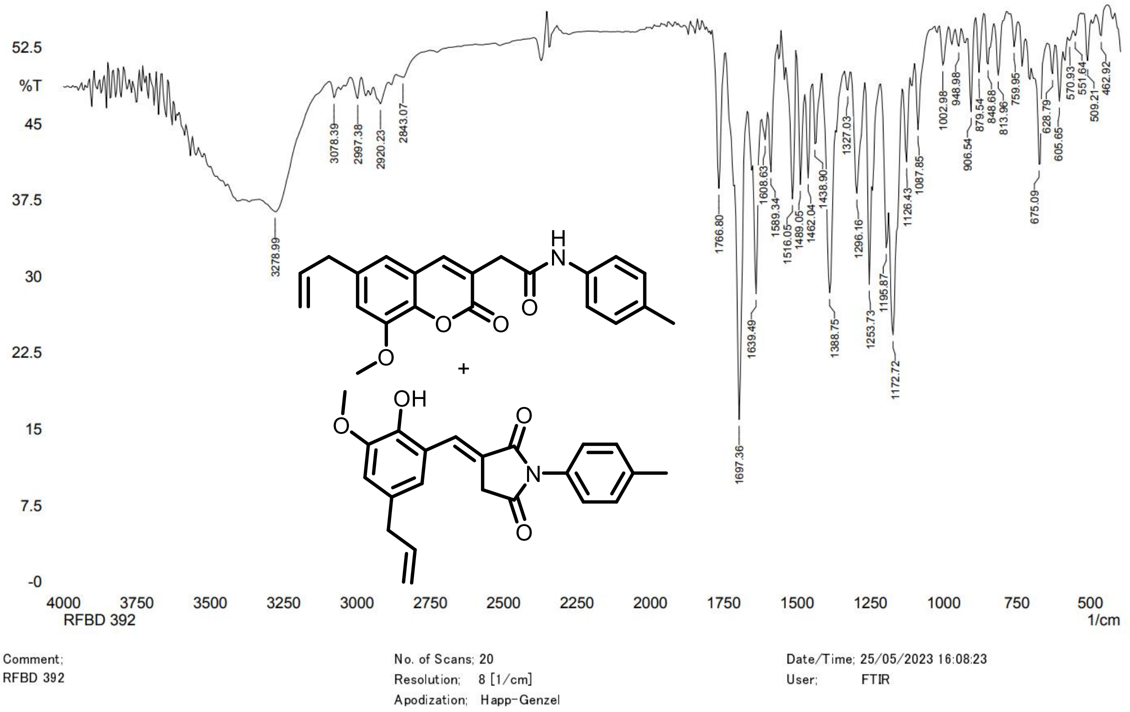

Figure S123. IR (KBr) of **4ag/4ag’**

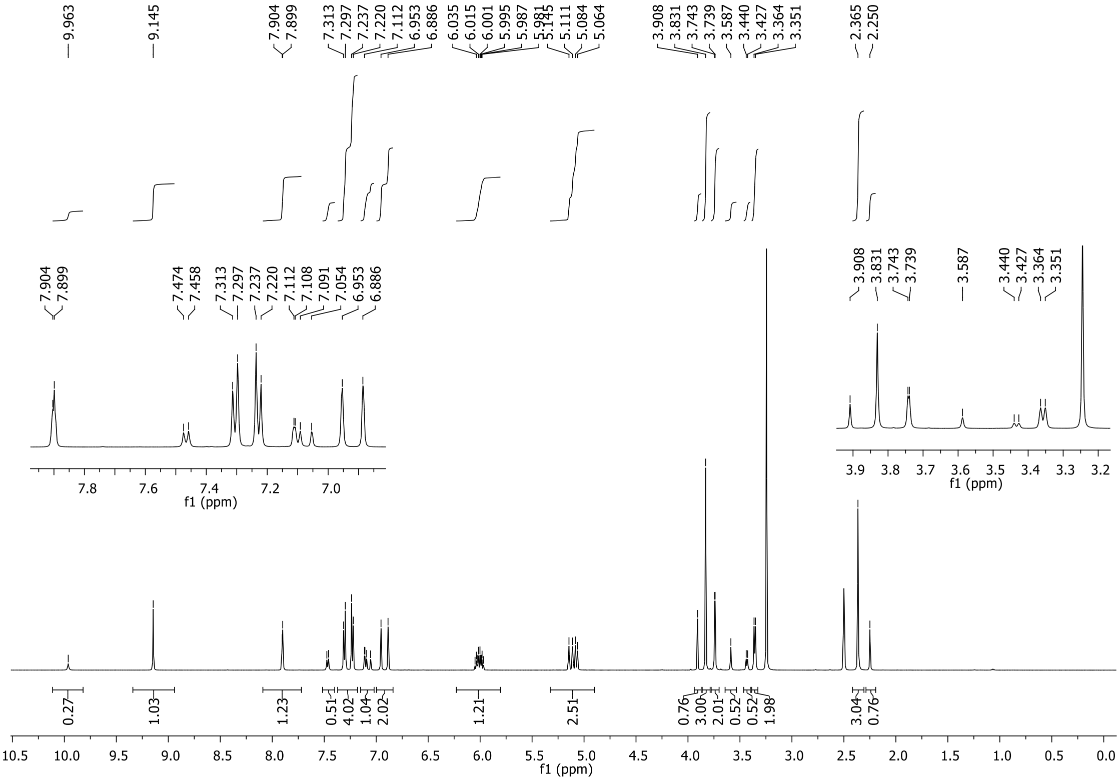

Figure S124. ^{1}H NMR (DMSO-d_6, 500 MHz) of **4ag/4ag’**

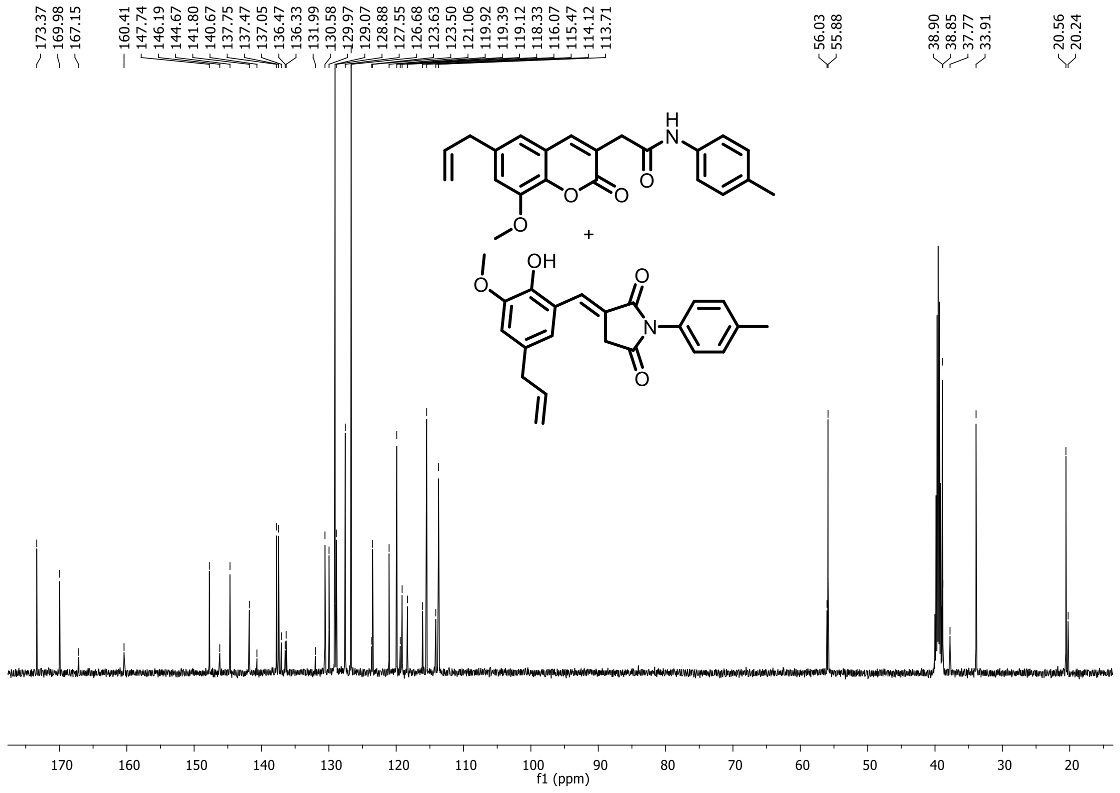

Figure S125. $^{13}C\{^{1}H\}$ NMR (DMSO-d$_6$, 125 MHz) of **4ag/4ag'**

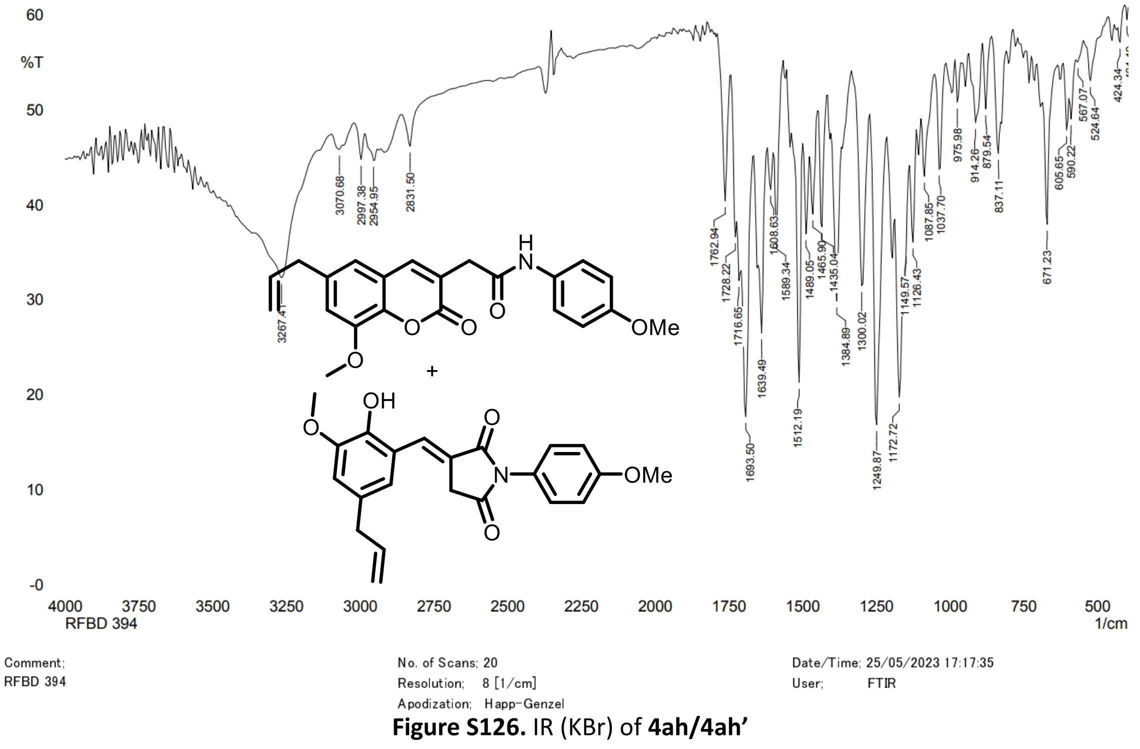

Figure S126. IR (KBr) of **4ah/4ah'**

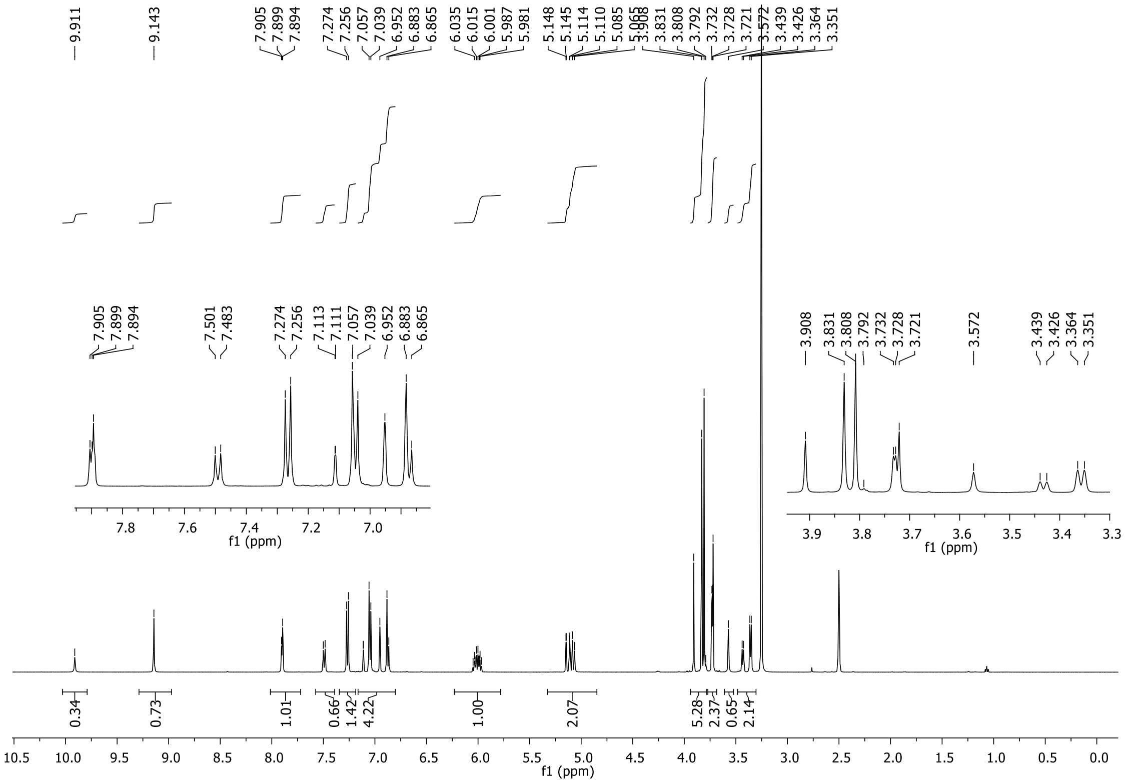

Figure S127. ^{1}H NMR (DMSO-d$_6$, 500 MHz) of **4ah/4ah’**

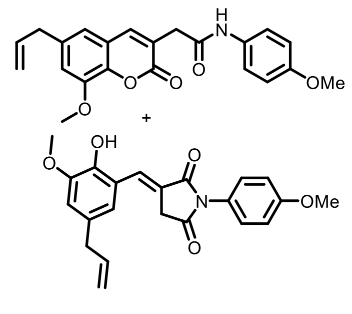

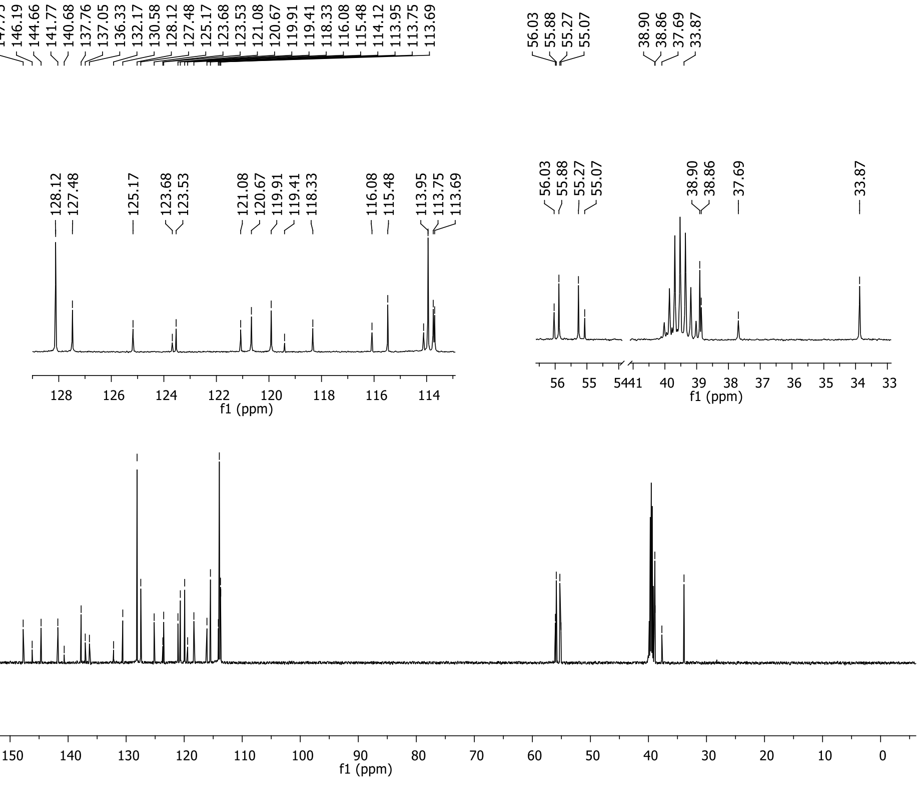

Figure S128. ^{13}C{^{1}H} NMR (DMSO-d$_6$, 125 MHz) of **4ah/4ah’**

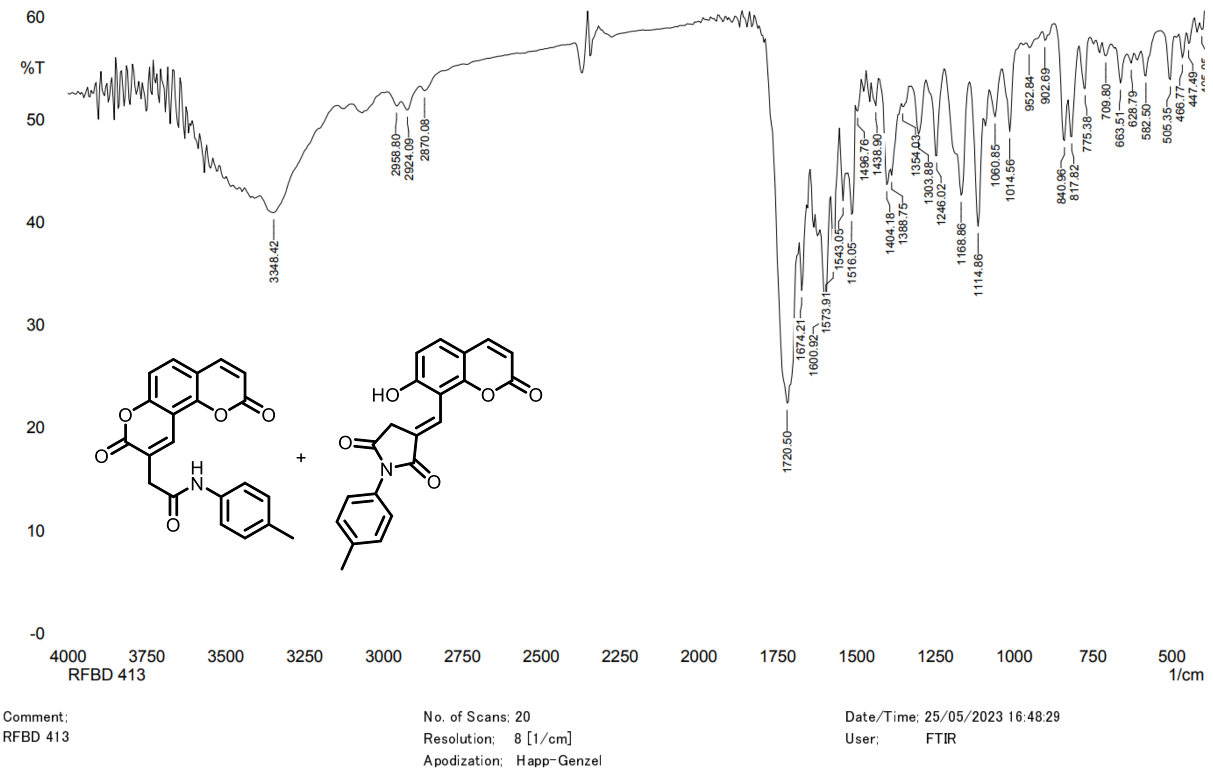

Figure S129. IR spectrum (KBr) of **4ai/4ai’**

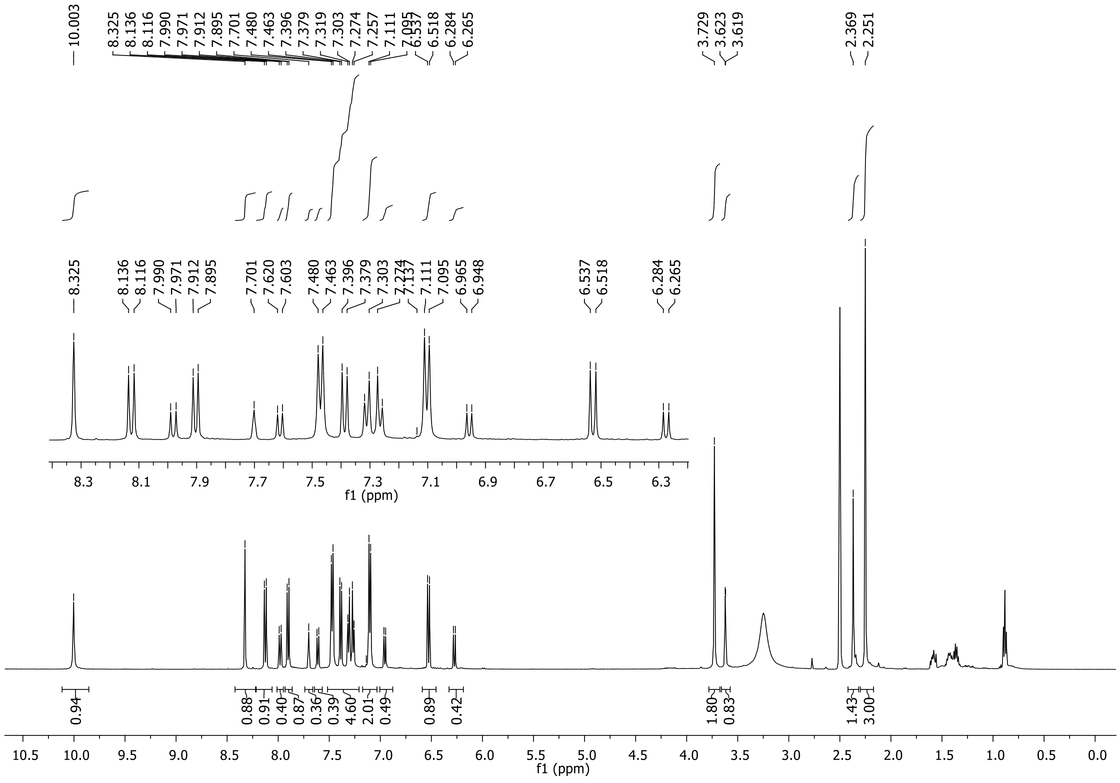

Figure S130. ^{1}H NMR (DMSO-d$_6$, 500 MHz) of **4ai/4ai’**

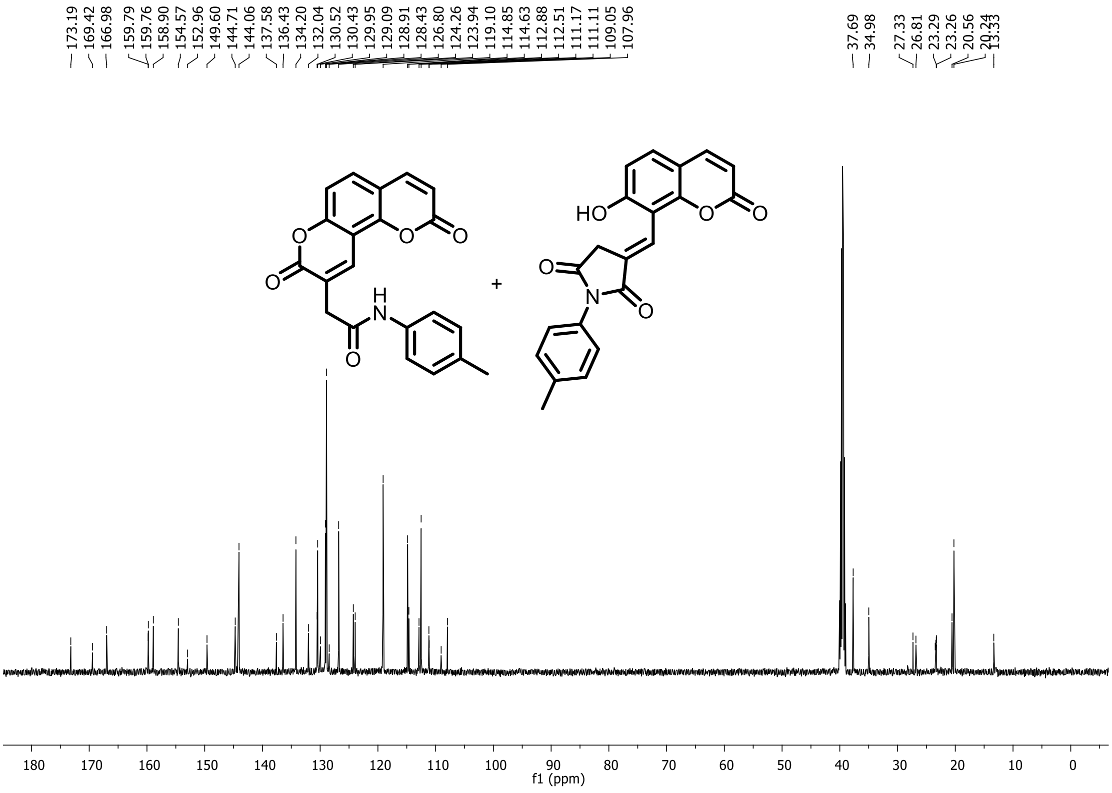

Figure S131. $^{13}C\{^{1}H\}$ NMR (DMSO-d$_6$, 125 MHz) of **4ai/4ai’**

6 Computational data

6.1. Energy profile (ΔG) of the evaluated pathway

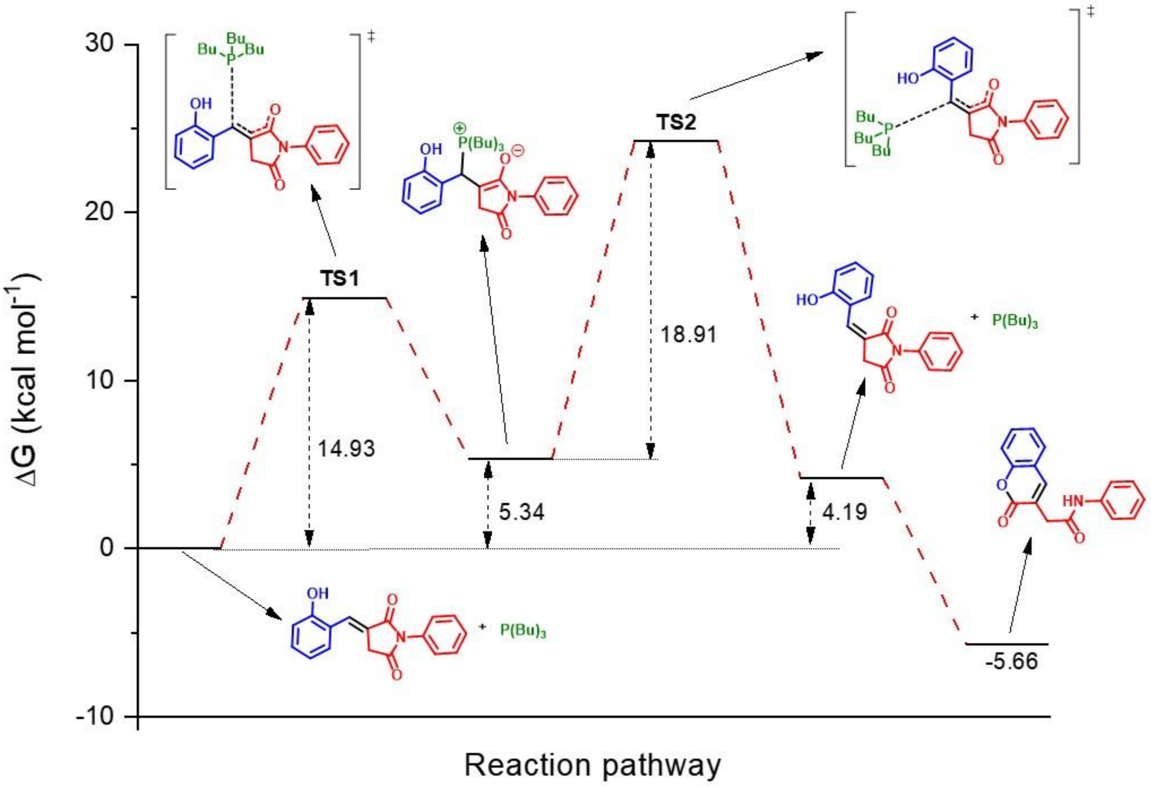

Figure S141. Reaction profile for itaconimide isomerizarion and coumarin formation.

6.2. Electronic energies (E), enthalpies (H) and Gibbs free energies (G) of all optimized structures

Table S2. Electronic energies (ΔE), enthalpies (ΔH) and Gibbs free energies (ΔG) variation for each investigated step involved in the formation of the coumarin product. Values in Hartree

Proposal		E	H	G
Step 1	*E*-Itaconimide + tributylphosphine	-1750.362068	-1749.64977	-1749.839881
	Transition state	-1750.339059	-1749.628445	-1749.816087
	Isomerization intermediate	-1750.354276	-1749.641107	-1749.82565
Step 2	Isomerization intermediate	-1750.359921	-1749.647039	-1749.831373
	Transition state	-1750.327653	-1749.616943	-1749.801241
	Z-Itaconimide + tributylphosphine	-1750.356766	-1749.644635	-1749.833202
Step 3	Z-Itaconimide	-935.7254492	-935.4255952	-935.5313782
	Coumarin	-935.7434982	-935.4423392	-935.5470702

6.3. Electronic energies (ΔE), enthalpies (ΔH) and Gibbs free energies (ΔG) variation along the reaction pathway

Table S3. Electronic energies (ΔE), enthalpies (ΔH) and Gibbs free energies (ΔG) for each investigated step involved in the formation of the coumarin product. Values in kcal mol^{-1}

Proposal		E	H	G
Step 1	*E*-Itaconimide + tributylphosphine	0.00	0.00	0.00
	Transition state	14.44	13.38	14.93
	Isomerization intermediate	4.89	5.44	8.93
Step 2	Isomerization intermediate	0.00	0.00	0.00
	Transition state	20.25	18.89	18.91
	Z-Itaconimide + tributylphosphine	1.98	1.51	-1.15
Step 3	Z-Itaconimide	0.00	0.00	0.00
	Coumarin	-11.33	-10.51	-9.85

6.4. Intrinsic reaction coordinates (IRC)

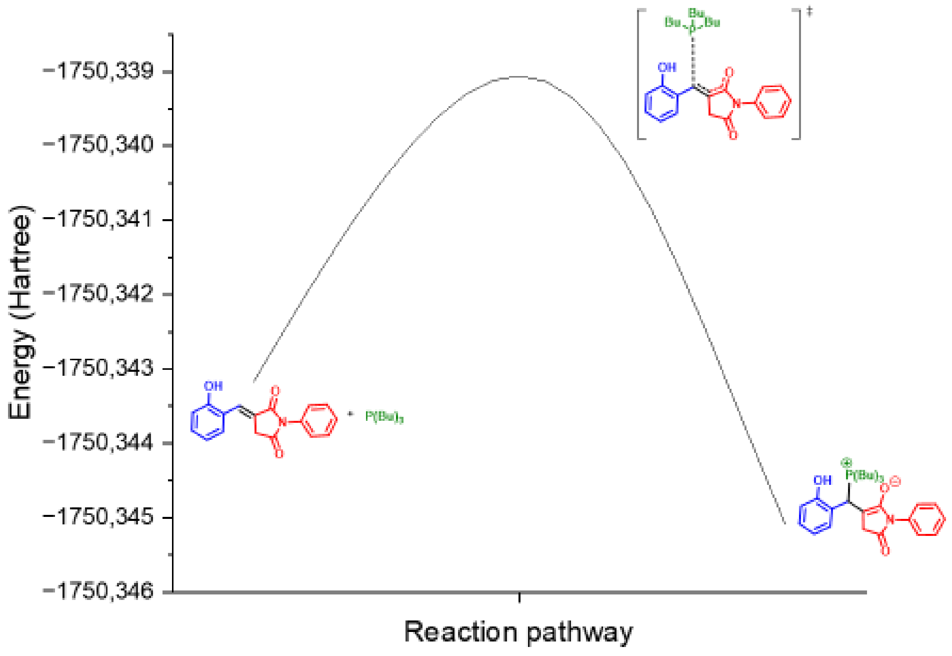

Figure S142. Step 1 - Intrinsic reaction coordinates (IRC)

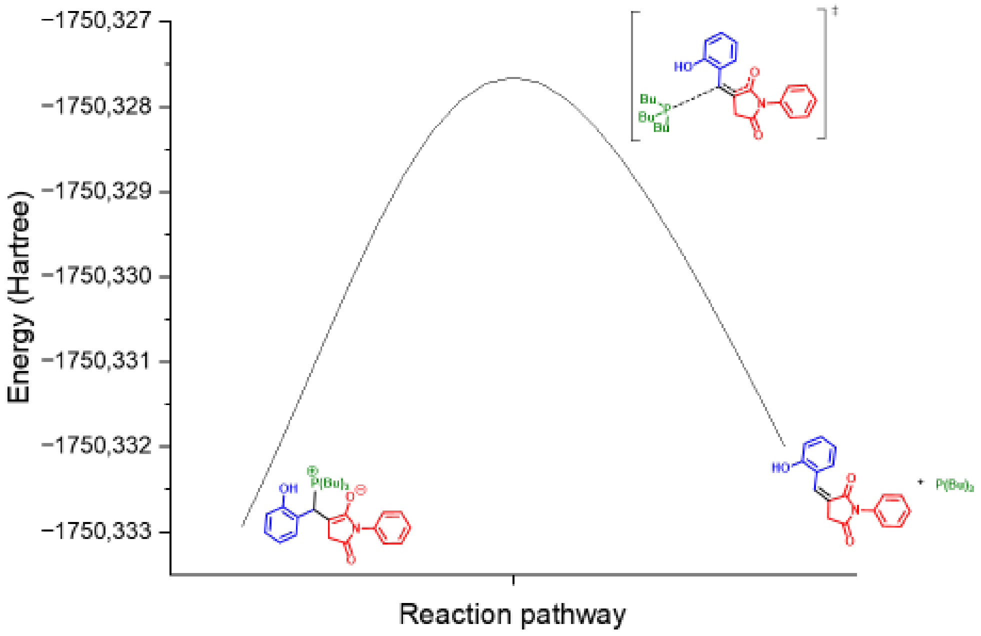

Figure S143. Step 2 - Intrinsic reaction coordinates (IRC)

6.5. Images of all optimized structures

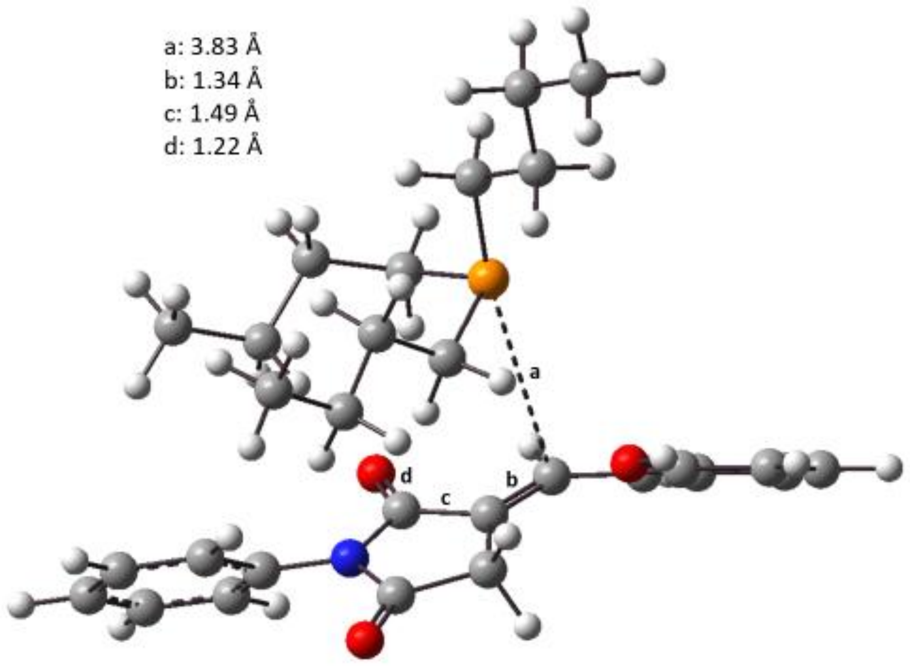

Figure S144. Step 1 - Molecular complex (E-Itaconimide + tributylphosphine)

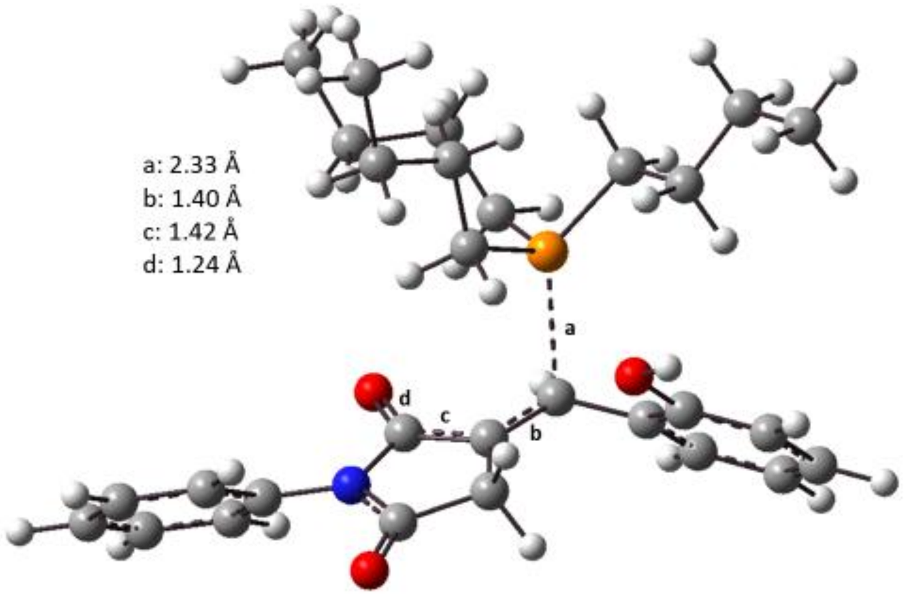

Figure S145. Step 1 - Transition state

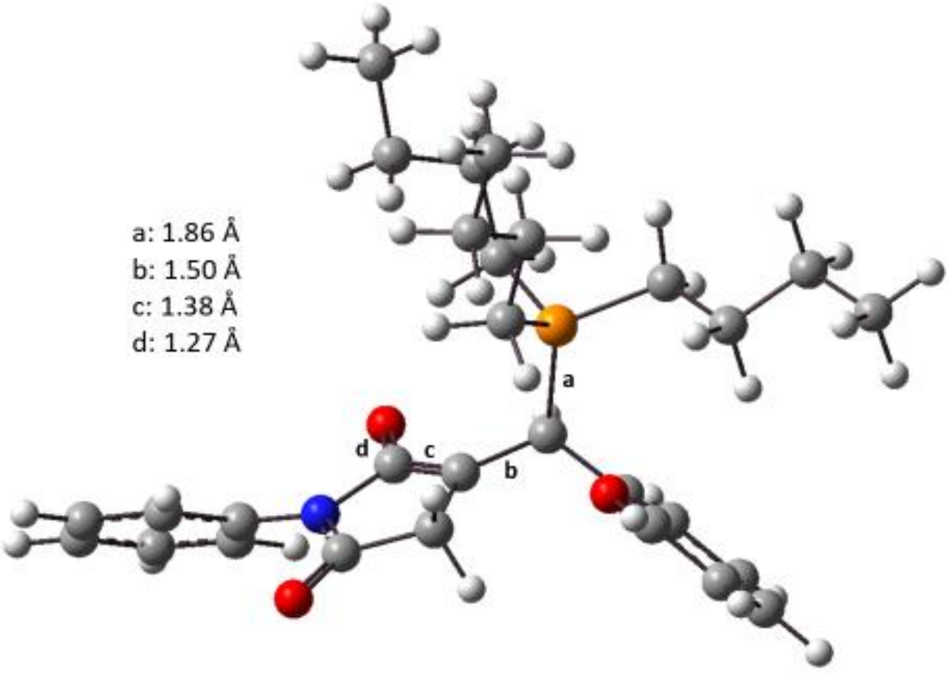

Figure S146. Step 1 - Isomerization intermediate

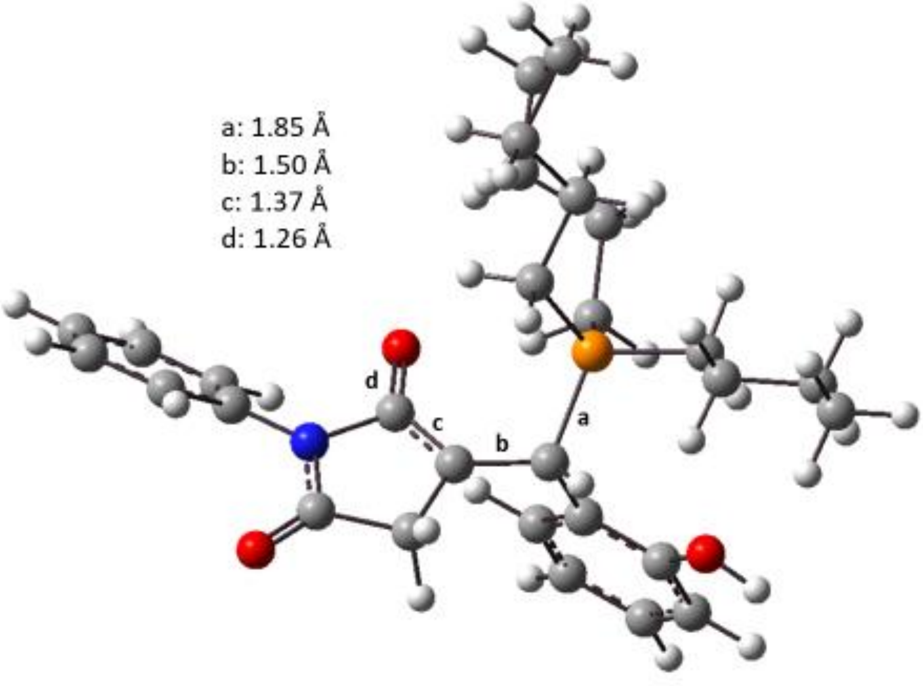

Figure S147. Step 2 - Isomerization intermediate

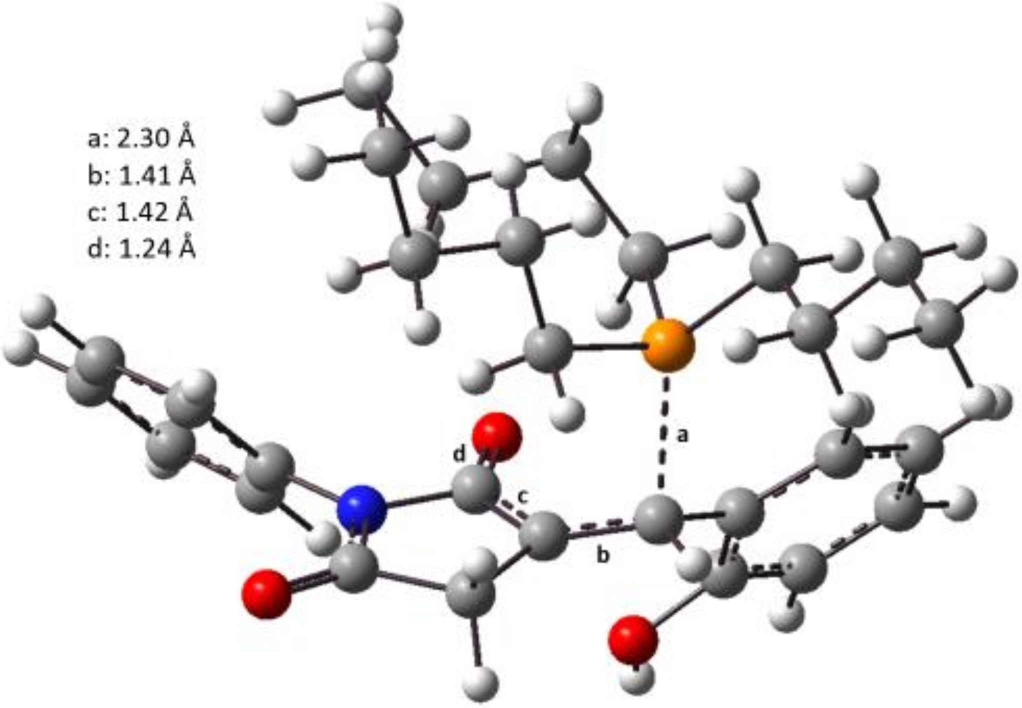

Figure S148. Step 2 - Transition state

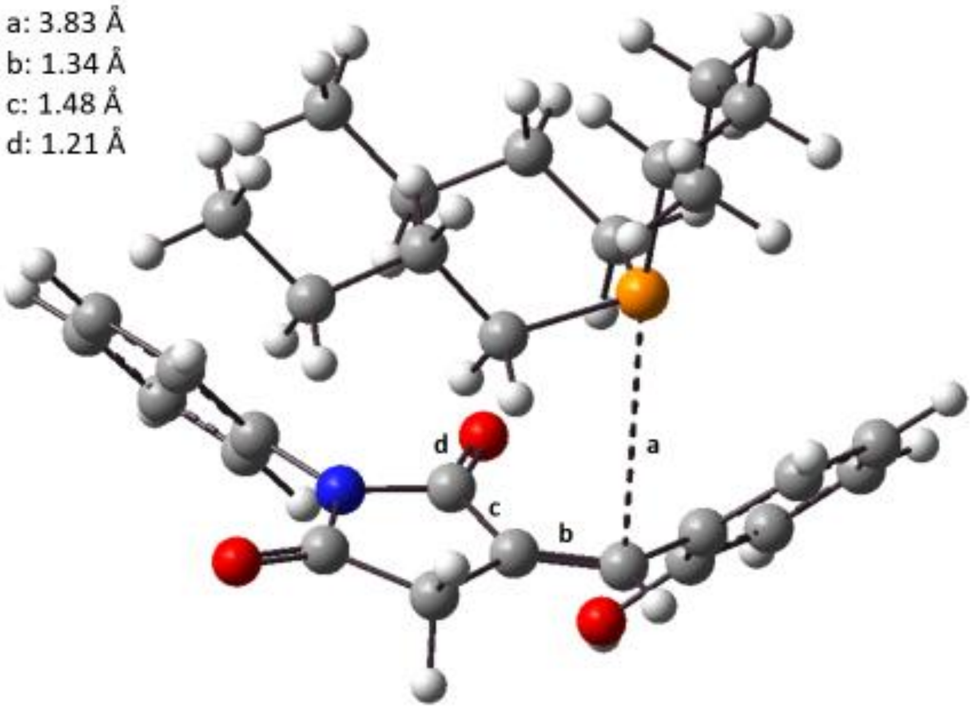

Figure S149. Step 2 - Molecular complex (Z-Itaconimide + tributylphosphine)

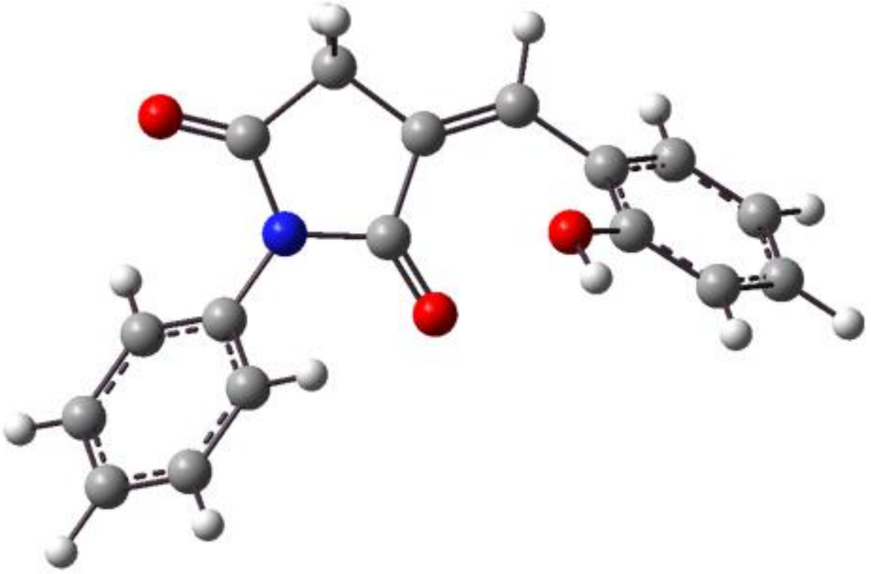

Figure S150. Z-Itaconimide

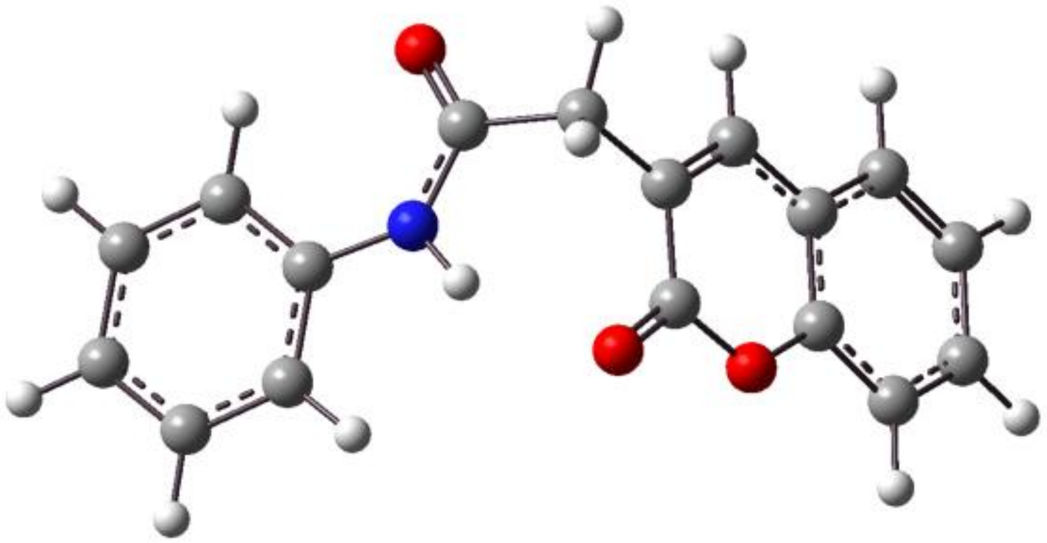

Figure S151. Coumarin

6.6. Coordinates of optimized structures

Step 1

- Molecular complex (*E*-Itaconimide + tributylphosphine)

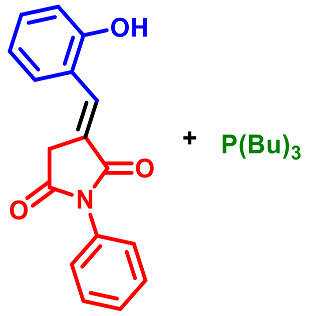

Symbolic Z-matrix:

Charge = 0 Multiplicity = 1

C	4.52056	-4.43721	0.27964
C	3.76588	-4.89124	-0.80325
C	2.55912	-4.2718	-1.09396
C	2.05825	-3.21843	-0.31047
C	2.85173	-2.75676	0.75621
C	4.07131	-3.37155	1.04837
H	5.47085	-4.90447	0.51991
H	4.12019	-5.71334	-1.41617
H	1.96494	-4.6094	-1.93935
H	4.66337	-2.99227	1.8776
O	2.4222	-1.67886	1.45677
H	3.081	-1.44171	2.1321
C	0.75873	-2.65471	-0.68378
H	0.53255	-2.71236	-1.74932
C	-0.23007	-2.15926	0.07838
C	-0.42421	-2.02166	1.55939
H	0.27206	-1.32431	2.03206
H	-0.34869	-2.97487	2.09465
N	-2.37023	-1.3167	0.41873
C	-1.82975	-1.47837	1.6966
C	-1.48302	-1.7181	-0.58715
O	-2.41526	-1.21241	2.72351
O	-1.74149	-1.69459	-1.77558
C	-3.69333	-0.84828	0.17082
C	-4.53508	-1.57719	-0.66898
C	-4.13084	0.32858	0.77706

C	-5.82585	-1.11423	-0.90723
H	-4.18127	-2.49577	-1.12492
C	-5.4297	0.7731	0.54325
H	-3.46015	0.88937	1.42079
C	-6.27654	0.05694	-0.30063
H	-6.48261	-1.67719	-1.56318
H	-5.77459	1.6872	1.01699
H	-7.28646	0.41036	-0.48456
P	1.83098	1.00674	-1.03797
C	0.50192	1.02938	-2.34579
H	1.07182	1.06061	-3.28378
H	0.02724	0.03885	-2.32623
C	-0.57902	2.11989	-2.35142
H	-0.16703	3.07203	-1.99211
H	-0.89001	2.30665	-3.38827
C	-1.83239	1.76132	-1.54837
H	-1.55868	1.41294	-0.54289
H	-2.33535	0.91428	-2.03319
C	-2.79259	2.94107	-1.43794
H	-3.70935	2.66921	-0.90446
H	-2.32623	3.77754	-0.9031
H	-3.08032	3.30456	-2.43157
C	0.91553	1.20545	0.57799
H	0.09249	0.48086	0.52989
H	1.58452	0.83697	1.36603
C	0.36544	2.57167	0.98891
H	-0.21637	3.02358	0.17674
H	1.19448	3.2624	1.19532
C	-0.51776	2.45468	2.23176
H	-1.33589	1.75	2.0204
H	0.06563	2.00805	3.04821
C	2.5837	2.69224	-1.24927
H	2.93985	2.76085	-2.28561
H	1.83166	3.48029	-1.11589
C	3.75253	2.91378	-0.28612
H	3.42754	2.72613	0.74749
H	4.54248	2.18035	-0.49695
C	4.33183	4.32499	-0.37409
H	3.54454	5.05059	-0.13046
H	4.63043	4.52792	-1.41091
C	-1.09397	3.79396	2.6797
H	-1.71105	4.23963	1.89105

H	-1.71956	3.68322	3.57125
H	-0.2948	4.50484	2.91816
C	5.52475	4.528	0.5557
H	5.91553	5.54862	0.49115
H	5.24546	4.3419	1.59907
H	6.34021	3.84095	0.30301

- Transition state

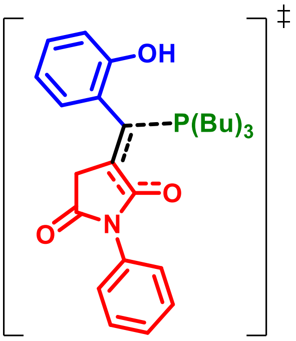

Symbolic Z-matrix:

Charge = 0 Multiplicity = 1

C	4.32105	-3.53895	-0.07565
C	3.72982	-3.63729	-1.34506
C	2.53691	-2.97636	-1.61317
C	1.87689	-2.20982	-0.62547
C	2.50235	-2.11822	0.63723
C	3.71592	-2.77746	0.91555
H	5.26134	-4.05895	0.12925
H	4.21178	-4.23168	-2.12268
H	2.08979	-3.05562	-2.6106
H	4.1682	-2.68129	1.90442
O	1.90514	-1.34556	1.59342
H	2.4568	-1.28855	2.4385
C	0.61527	-1.57218	-1.01146
H	0.56691	-1.27187	-2.07694
C	-0.53559	-1.56061	-0.28689
C	-0.78455	-2.06381	1.10402
H	-0.1246	-1.56662	1.86017
H	-0.56627	-3.15119	1.20939
N	-2.7859	-1.10751	0.23765
C	-2.23746	-1.77012	1.3892
C	-1.73731	-0.9867	-0.77974
O	-2.88745	-2.03019	2.38101
O	-1.98981	-0.40997	-1.91712
C	-4.14334	-0.63029	0.12994

C	-4.8354	-0.77615	-1.0838
C	-4.75393	-0.02303	1.23993
C	-6.14598	-0.30399	-1.17875
H	-4.35871	-1.24882	-1.94445
C	-6.06816	0.43427	1.12621
H	-4.21339	0.0926	2.18102
C	-6.76446	0.29748	-0.07858
H	-6.69023	-0.40952	-2.121
H	-6.55207	0.90407	1.98653
H	-7.79096	0.65985	-0.15956
P	1.2377	0.7387	-0.83141
C	0.16715	1.59297	-2.16681
H	0.47395	1.1443	-3.13777
H	-0.88251	1.26335	-2.0168
C	0.2387	3.11322	-2.28968
H	1.24939	3.50729	-2.06352
H	0.08245	3.37944	-3.35872
C	-0.80948	3.84382	-1.43884
H	-0.5788	3.71242	-0.35463
H	-1.80755	3.38559	-1.58302
C	-0.87182	5.3317	-1.76439
H	-1.60886	5.84752	-1.13548
H	0.09179	5.831	-1.60482
H	-1.15834	5.51653	-2.80712
C	0.49371	1.19968	0.86985
H	-0.60507	1.06581	0.80551
H	0.841	0.42214	1.58686
C	0.83443	2.58583	1.40251
H	0.4516	3.37996	0.71569
H	1.93022	2.74464	1.45739
C	0.22542	2.80273	2.79881
H	-0.87579	2.68515	2.75496
H	0.57575	2.01258	3.49224
C	2.92264	1.62943	-0.93269
H	3.25323	1.61899	-1.99141
H	2.81328	2.70007	-0.67262
C	3.95218	0.94919	-0.03593
H	3.61495	0.91628	1.02083
H	4.10882	-0.10948	-0.32951
C	5.30087	1.68843	-0.10489
H	5.16494	2.75021	0.18234
H	5.66409	1.71531	-1.15143

C	0.57611	4.17548	3.36161
H	0.18633	4.98964	2.73841
H	0.1576	4.31607	4.36655
H	1.65967	4.32593	3.44338
C	6.3496	1.04085	0.79174
H	7.30866	1.5714	0.73348
H	6.0496	1.04071	1.84702
H	6.54475	-0.00289	0.51628

- Isomerization intermediate

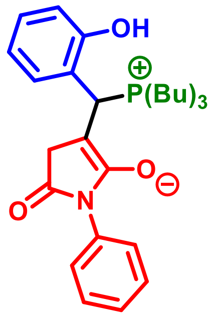

Symbolic Z-matrix:

Charge = 0 Multiplicity = 1

C	3.83915	-4.15332	-0.37646
C	3.13492	-4.11505	-1.57975
C	2.11486	-3.17257	-1.7632
C	1.79245	-2.25136	-0.76173
C	2.46918	-2.36039	0.46137
C	3.48678	-3.28503	0.66243
H	4.64878	-4.86359	-0.22856
H	3.39285	-4.80625	-2.37878
H	1.58746	-3.13239	-2.71821
H	4.01119	-3.30358	1.60924
O	2.08491	-1.4571	1.41305
H	2.68152	-1.51374	2.17416
C	0.77944	-1.08184	-0.94858
H	0.49047	-1.19882	-2.05535
C	-0.56175	-1.43168	-0.20419
C	-0.78302	-2.179	1.09463
H	-0.24892	-1.78565	1.98049
H	-0.55366	-3.25929	1.07872
N	-2.79891	-1.311	0.35215
C	-2.27555	-2.02731	1.36901
C	-1.73947	-0.95343	-0.63723
O	-2.89232	-2.48171	2.33943

O	-2.08936	-0.23454	-1.63238
C	-4.1578	-0.91425	0.25615
C	-4.86516	-1.10204	-0.92696
C	-4.79981	-0.32908	1.35466
C	-6.19613	-0.70489	-1.0181
H	-4.36459	-1.5533	-1.78141
C	-6.13569	0.04964	1.26796
H	-4.24481	-0.18291	2.28374
C	-6.83599	-0.12776	0.07752
H	-6.73618	-0.84566	-1.94805
H	-6.61657	0.50097	2.12496
H	-7.87628	0.17456	0.0041
P	1.25704	0.55926	-0.77118
C	0.7378	1.31377	-2.3645
H	1.4796	0.96583	-3.08981
H	-0.21652	0.84592	-2.61333
C	0.59385	2.8545	-2.42677
H	1.39996	3.36007	-1.88316
H	0.72663	3.14146	-3.47576
C	-0.77648	3.35578	-1.95734
H	-1.00036	2.96938	-0.95393
H	-1.53972	2.93513	-2.6165
C	-0.86989	4.8754	-1.94446
H	-1.86484	5.20248	-1.62604
H	-0.14508	5.3175	-1.25084
H	-0.68384	5.29048	-2.93933
C	0.42293	1.28786	0.65194
H	-0.64665	1.27076	0.41072
H	0.56756	0.57922	1.4722
C	0.86978	2.70239	1.07393
H	0.88971	3.39507	0.22145
H	1.88835	2.67426	1.48362
C	-0.09469	3.25746	2.13718
H	-1.10617	3.30079	1.70143
H	-0.14834	2.54373	2.97017
C	3.07222	1.06397	-0.69739
H	3.54215	0.74599	-1.63683
H	2.91753	2.15782	-0.76504
C	3.92962	0.70856	0.53177
H	3.36478	0.883	1.45304
H	4.16888	-0.36714	0.4965
C	5.23524	1.50256	0.58991

H	5.02202	2.57655	0.70831
H	5.77142	1.40203	-0.36713
C	0.30616	4.63498	2.64617
H	0.33442	5.36056	1.83383
H	-0.39391	4.99583	3.40635
H	1.30551	4.61011	3.10495
C	6.12105	1.0137	1.73468
H	7.06176	1.56316	1.77895
H	5.61399	1.1187	2.69411
H	6.36691	-0.05344	1.6028

Step 2

- Isomerization intermediate

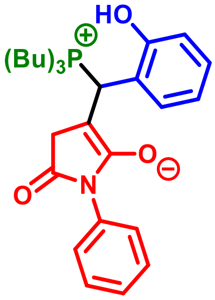

Symbolic Z-matrix:

Charge = 0 Multiplicity = 1

C	1.74367	5.1328	1.1414
C	2.67863	4.11258	1.17746
C	2.40402	2.92408	0.51427
C	1.2369	2.6748	-0.2145
C	0.32411	3.76453	-0.31616
C	0.58646	4.95732	0.38721
H	1.90732	6.07414	1.66561
H	3.61623	4.22989	1.71826
H	3.15755	2.14288	0.56137
H	-0.15662	5.75833	0.33055
O	-0.7846	3.72664	-1.09554
H	-1.24613	4.58255	-1.0064
C	1.12697	1.19517	-0.79356
H	1.70919	1.25993	-1.75383
C	-0.33418	0.86448	-1.23721
C	-0.70718	0.18788	-2.54853
H	-0.08333	-0.66503	-2.85814
H	-0.75632	0.86979	-3.4184

N	-2.53308	0.19161	-1.1274
C	-2.11778	-0.33229	-2.29821
C	-1.42151	0.95065	-0.44926
O	-2.76734	-1.07008	-3.04343
O	-1.70704	1.48533	0.66947
C	-3.73566	-0.13462	-0.47585
C	-4.56294	0.87357	0.01903
C	-4.10117	-1.47385	-0.29389
C	-5.75038	0.5461	0.67778
H	-4.27168	1.90902	-0.12281
C	-5.28704	-1.80079	0.35448
H	-3.44052	-2.25555	-0.66653
C	-6.11714	-0.78846	0.83364
H	-6.38305	1.33835	1.04981
H	-5.56515	-2.8424	0.4742
H	-7.04619	-1.03891	1.34674
P	1.62896	-0.07193	0.17487
C	0.91526	0.24377	1.83237
H	1.70441	0.7796	2.36716
H	0.08401	0.92892	1.66765
C	0.4395	-0.97395	2.64898
H	1.20621	-1.76469	2.6786
H	0.32747	-0.62343	3.67766
C	-0.90752	-1.513	2.17342
H	-0.86412	-1.79604	1.11255
H	-1.6445	-0.71184	2.2323
C	-1.37025	-2.71774	2.98406
H	-2.35052	-3.07023	2.63624
H	-0.66913	-3.55603	2.89236
H	-1.45856	-2.47378	4.04897
C	1.12295	-1.61432	-0.66886
H	0.04277	-1.54501	-0.81102
H	1.59473	-1.57022	-1.65819
C	1.48572	-2.96146	0.01171
H	1.17243	-2.97256	1.05821
H	2.56936	-3.11836	-0.00555
C	0.78001	-4.10521	-0.72248
H	-0.30569	-3.94056	-0.68233
H	1.06144	-4.08002	-1.78031
C	3.44696	-0.41333	0.5627
H	3.85171	0.40858	1.15592
H	3.27134	-1.25318	1.25903

C	4.38281	-0.82454	-0.57858
H	3.93512	-1.63746	-1.17275
H	4.52618	0.03056	-1.25859
C	5.76752	-1.29256	-0.07575
H	5.63687	-2.2034	0.52755
H	6.18355	-0.52692	0.59527
C	1.10997	-5.47563	-0.13007
H	0.81625	-5.53832	0.92108
H	0.59719	-6.27541	-0.67376
H	2.18647	-5.68456	-0.18773
C	6.73013	-1.55673	-1.22963
H	7.70849	-1.88243	-0.87061
H	6.33065	-2.33076	-1.90468
H	6.88057	-0.64823	-1.82677

- Transition state

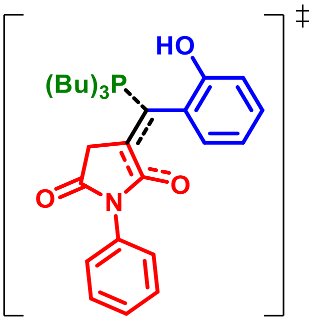

Symbolic Z-matrix:

Charge = 0 Multiplicity = 1

C	1.67186	5.16542	1.1379
C	2.59696	4.12435	1.1496
C	2.30986	2.95722	0.45306
C	1.12059	2.77031	-0.26394
C	0.22633	3.86048	-0.30993
C	0.50147	5.0321	0.40561
H	1.86199	6.08323	1.68624
H	3.52936	4.21506	1.69727
H	3.04241	2.15449	0.46251
H	-0.22025	5.84523	0.36666
O	-0.88741	3.7939	-1.0852
H	-1.37079	4.63358	-1.01178
C	0.88714	1.5143	-1.06814
H	1.58837	1.42618	-1.90007
C	-0.38876	0.99856	-1.35754
C	-0.72853	0.19917	-2.58786

H	-0.05203	-0.63193	-2.81611
H	-0.79962	0.80995	-3.49835
N	-2.51448	0.15882	-1.08668
C	-2.10746	-0.3549	-2.29705
C	-1.5063	0.99325	-0.48016
O	-2.76483	-1.10502	-3.00186
O	-1.7145	1.52162	0.61627
C	-3.73886	-0.16662	-0.44829
C	-4.56878	0.85207	0.02096
C	-4.09452	-1.50569	-0.27544
C	-5.75903	0.52517	0.66484
H	-4.27754	1.8874	-0.12172
C	-5.29331	-1.82206	0.35773
H	-3.43186	-2.28772	-0.63358
C	-6.12608	-0.80927	0.83119
H	-6.40382	1.31777	1.03239
H	-5.57162	-2.86358	0.48775
H	-7.05751	-1.05924	1.33011
P	1.81997	-0.14052	0.22968
C	0.98051	0.17139	1.84725
H	1.73728	0.72321	2.41907
H	0.16915	0.87733	1.64556
C	0.44344	-1.00291	2.67397
H	1.18337	-1.81057	2.73468
H	0.30326	-0.64776	3.70347
C	-0.89809	-1.53587	2.16764
H	-0.82741	-1.80337	1.10579
H	-1.6393	-0.72854	2.22582
C	-1.37968	-2.74663	2.95993
H	-2.35367	-3.09566	2.60116
H	-0.67125	-3.57935	2.87357
H	-1.48118	-2.50612	4.02444
C	1.19209	-1.65709	-0.60986
H	0.11157	-1.53613	-0.73622
H	1.62784	-1.62635	-1.61745
C	1.50243	-3.013	0.02926
H	1.16813	-3.03996	1.07213
H	2.58759	-3.17979	0.04296
C	0.82457	-4.15065	-0.73532
H	-0.2624	-3.99264	-0.71954
H	1.12993	-4.11251	-1.78911
C	3.54235	-0.60534	0.71863

H	3.93964	0.22034	1.32167
H	3.49398	-1.4854	1.37534
C	4.45198	-0.8689	-0.48139
H	4.02447	-1.65903	-1.11352
H	4.50473	0.03517	-1.10391
C	5.8614	-1.27841	-0.05573
H	5.80054	-2.19414	0.54651
H	6.27994	-0.5022	0.5977
C	1.15433	-5.5197	-0.14952
H	0.83347	-5.58612	0.89632
H	0.65596	-6.32193	-0.70286
H	2.23275	-5.71143	-0.17978
C	6.78576	-1.50475	-1.24779
H	7.78768	-1.80624	-0.92617
H	6.39446	-2.29008	-1.90434
H	6.88622	-0.59128	-1.84455

- Molecular complex (*Z*-Itaconimide + tributylphosphine)

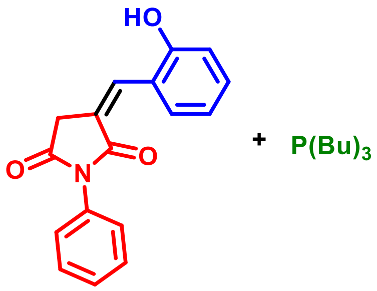

Symbolic Z-matrix:

Charge = 0 Multiplicity = 1

C	-1.15128	5.45625	1.22358
C	0.11667	5.10157	0.76463
C	0.23628	4.09317	-0.18467
C	-0.88222	3.39867	-0.66408
C	-2.15677	3.79447	-0.21537
C	-2.2814	4.8157	0.72875
H	-1.26704	6.24463	1.96149
H	1.00086	5.60886	1.13624
H	1.21715	3.81226	-0.55933
H	-3.27454	5.10428	1.06409
O	-3.24594	3.19063	-0.75372
H	-4.05141	3.56196	-0.35631
C	-0.69354	2.34459	-1.67916
H	-0.04649	2.61149	-2.51605
C	-1.21421	1.10886	-1.69086

C	-1.08091	0.08996	-2.79104
H	-0.04631	-0.14922	-3.05885
H	-1.60574	0.38666	-3.70563
N	-2.24199	-0.84439	-0.96575
C	-1.75152	-1.14713	-2.23609
C	-1.95356	0.47486	-0.57069
O	-1.86287	-2.22727	-2.77483
O	-2.23518	0.92289	0.52137
C	-2.95303	-1.76762	-0.14531
C	-4.15139	-1.3705	0.44817
C	-2.44794	-3.05165	0.05553
C	-4.8438	-2.26849	1.25451
H	-4.53352	-0.36985	0.27579
C	-3.15823	-3.9466	0.85228
H	-1.51017	-3.34578	-0.40471
C	-4.35212	-3.55726	1.45603
H	-5.77464	-1.95994	1.72028
H	-2.76848	-4.94829	1.00619
H	-4.89885	-4.25618	2.08148
P	2.38223	0.86705	0.05379
C	1.21383	0.91469	1.50604
H	1.57243	1.76668	2.09918
H	0.23584	1.2292	1.11218
C	1.04718	-0.31846	2.40807
H	1.99198	-0.87348	2.47667
H	0.82751	0.01652	3.43096
C	-0.07438	-1.26962	1.98257
H	0.00925	-1.51285	0.91437
H	-1.0386	-0.75902	2.09966
C	-0.06344	-2.5582	2.79882
H	-0.89825	-3.2147	2.53142
H	0.8682	-3.11444	2.63883
H	-0.13796	-2.34276	3.87132
C	1.87255	-0.6607	-0.89067
H	0.7755	-0.62897	-0.89975
H	2.18188	-0.51649	-1.93391
C	2.33257	-2.03819	-0.41418
H	2.0967	-2.17924	0.64811
H	3.42457	-2.12041	-0.50046
C	1.67402	-3.15302	-1.22761
H	0.5824	-3.02553	-1.18613
H	1.95301	-3.04269	-2.28394

C	3.96219	0.31863	0.86073
H	4.17524	1.0356	1.66494
H	3.84879	-0.66566	1.33194
C	5.12662	0.28981	-0.1314
H	4.86963	-0.34869	-0.98928
H	5.28674	1.2984	-0.53575
C	6.42348	-0.21751	0.49789
H	6.26432	-1.23441	0.88019
H	6.66867	0.40651	1.36728
C	2.04743	-4.54681	-0.73473
H	1.7391	-4.68862	0.30753
H	1.56693	-5.32687	-1.33404
H	3.13062	-4.7057	-0.78509
C	7.59075	-0.21365	-0.48427
H	8.51139	-0.57748	-0.01687
H	7.37943	-0.85356	-1.3485
H	7.78329	0.79781	-0.85959

Step 3

- *Z*-Itaconimide

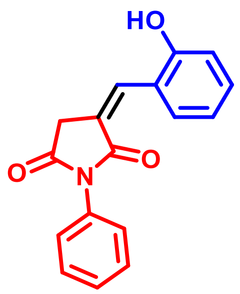

Symbolic Z-matrix:

Charge = 0 Multiplicity = 1

C	1.44413	5.40968	-1.26259
C	0.15126	5.09759	-0.84419
C	-0.03084	4.10296	0.10964
C	1.05002	3.38159	0.63279
C	2.34991	3.73227	0.224
C	2.53732	4.7398	-0.72499
H	1.60763	6.18758	-2.00254
H	-0.70373	5.62789	-1.25057
H	-1.03191	3.85407	0.45328
H	3.54925	4.99495	-1.03006
O	3.39781	3.09613	0.8055
H	4.23095	3.43487	0.43704

C	0.79596	2.34424	1.64981
H	0.14572	2.64468	2.47268
C	1.25916	1.08638	1.67707
C	1.04173	0.07943	2.7742
H	-0.00836	-0.06788	3.044
H	1.59113	0.32991	3.68866
N	2.17253	-0.92855	0.97131
C	1.61569	-1.20492	2.21956
C	1.99136	0.40962	0.57675
O	1.62324	-2.29647	2.74695
O	2.34206	0.84131	-0.50192
C	2.86466	-1.89012	0.17857
C	4.12913	-1.57756	-0.32043
C	2.27981	-3.12844	-0.08141
C	4.80833	-2.51486	-1.09271
H	4.57273	-0.61192	-0.10044
C	2.97597	-4.06459	-0.84229
H	1.28992	-3.35326	0.30277
C	4.23635	-3.75942	-1.35208
H	5.79105	-2.27256	-1.48511
H	2.5242	-5.03144	-1.04238
H	4.77235	-4.49018	-1.94985

- Coumarin

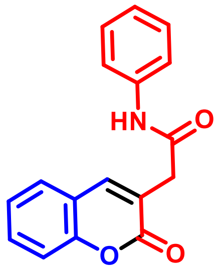

Symbolic Z-matrix:

Charge = 0 Multiplicity = 1

C	5.08203	-1.3626	-0.99041
C	5.11663	-0.08568	-1.56808
C	4.12694	0.83609	-1.27232
C	3.08288	0.49662	-0.39656
C	3.07233	-0.78568	0.16034
C	4.06058	-1.72308	-0.1229
H	5.86039	-2.08231	-1.22343
H	5.92183	0.1794	-2.24521
H	4.14017	1.83041	-1.70941

H	4.01338	-2.70567	0.33435
O	2.07632	-1.16302	1.02124
C	2.01951	1.39717	-0.02906
H	2.02299	2.4026	-0.44443
C	1.03852	1.01448	0.8173
C	-0.09583	1.91433	1.23388
H	0.18739	2.95684	1.08102
H	-0.32488	1.75828	2.2931
N	-1.99139	0.51024	0.68616
C	-1.34531	1.66879	0.39037
C	1.05349	-0.34112	1.3691
O	-1.69456	2.45797	-0.47936
O	0.21082	-0.79192	2.12614
C	-3.11299	-0.05802	0.04843
C	-3.39196	-1.39702	0.35162
C	-3.93358	0.63544	-0.8497
C	-4.47869	-2.03582	-0.2317
H	-2.74197	-1.9302	1.04092
C	-5.01619	-0.02258	-1.43088
H	-3.72296	1.66767	-1.09027
C	-5.29987	-1.35134	-1.12673
H	-4.6819	-3.07402	0.01417
H	-5.64818	0.52049	-2.12759
H	-6.14865	-1.85018	-1.58393
H	-1.54944	-0.06916	1.39717
